# Loci and pathways associated with uterine capacity for pregnancy and fertility in beef cattle

**DOI:** 10.1371/journal.pone.0188997

**Published:** 2017-12-11

**Authors:** Mahesh Neupane, Thomas W. Geary, Jennifer N. Kiser, Gregory W. Burns, Peter J. Hansen, Thomas E. Spencer, Holly L. Neibergs

**Affiliations:** 1 Department Animal Sciences, Washington State University, Pullman, Washington, United States of America; 2 USDA-ARS, Fort Keogh Livestock and Range Research Laboratory, Miles City, Montana, United States of America; 3 Division of Animal Sciences, University of Missouri, Columbia, Missouri, United States of America; 4 Department of Animal Sciences, University of Florida, Gainesville, Florida, United States of America; Universite du Quebec a Trois-Rivieres, CANADA

## Abstract

Infertility and subfertility negatively impact the economics and reproductive performance of cattle. Of note, significant pregnancy loss occurs in cattle during the first month of pregnancy, yet little is known about the genetic loci influencing pregnancy success and loss in cattle. To identify quantitative trait loci (QTL) with large effects associated with early pregnancy loss, Angus crossbred heifers were classified based on day 28 pregnancy outcomes to serial embryo transfer. A genome wide association analysis (GWAA) was conducted comparing 30 high fertility heifers with 100% success in establishing pregnancy to 55 subfertile heifers with 25% or less success. A gene set enrichment analysis SNP (GSEA-SNP) was performed to identify gene sets and leading edge genes influencing pregnancy loss. The GWAA identified 22 QTL (p < 1 x 10^−5^), and GSEA-SNP identified 9 gene sets (normalized enrichment score > 3.0) with 253 leading edge genes. Network analysis identified TNF (tumor necrosis factor), estrogen, and TP53 (tumor protein 53) as the top of 671 upstream regulators (p < 0.001), whereas the SOX2 (SRY [sex determining region Y]-box 2) and OCT4 (octamer-binding transcription factor 4) complex was the top master regulator out of 773 master regulators associated with fertility (p < 0.001). Identification of QTL and genes in pathways that improve early pregnancy success provides critical information for genomic selection to increase fertility in cattle. The identified genes and regulators also provide insight into the complex biological mechanisms underlying pregnancy establishment in cattle.

## Introduction

Embryonic mortality is a major factor that negatively affects fertility, production and economic efficiency in ruminants [1–4]. Between fertilization and term, pregnancy loss in cattle is high and ranges from 40% to 56% [4, 5], and 70–80% of embryonic loss occurs within the first month of pregnancy [4–8]. Infertility and subfertility also impact the cattle embryo transfer (ET) industry [9]. Mean survival rate to calving following transfer of an *in vitro* produced (IVP) embryo is lower (30%-40%) as compared to *in vivo* derived embryos from superovulated donors (39%-60%) [3, 10, 11]. Both embryonic and maternal factors are important in survival of the conceptus (embryo/fetus and associated extraembryonic membranes) and establishment of pregnancy [12, 13].

After fertilization (day 0) in the oviduct, the morula embryo enters the uterus on days 4 to 5 and forms a blastocyst by day 7. After hatching from the zona pellucida on days 8 to 9, the blastocyst develops into an ovoid conceptus between days 12 to 14 [14]. The ovoid conceptus begins to elongate and increases in length from 2 mm on day 14 to 20 cm or more by day 19 [15]. Maternal recognition of pregnancy is initiated on days 16 to 17 followed by implantation and placentation [16]. The actions of progesterone on the endometrium of the uterus is critical for conceptus growth and elongation [17–19]. Between days 7 and 13, dynamic changes in endometrial gene expression occur in response to progesterone from the ovary that are crucial to support blastocyst growth and conceptus elongation [18–21]. Although much knowledge of how the embryo develops to a blastocyst has been gained from *in vitro* systems [22–25], our understanding of conceptus survival, elongation and implantation for the establishment of pregnancy in cattle is very limited [26].

One of the major impediments to understanding maternal contributions to pregnancy loss in cattle has been a lack of animals with defined high and low rates of early pregnancy loss. McMillan and Donnison [27] summarized a unique approach for experimentally identifying high and low fertility in dairy heifers based on serial transfer of *in vitro* produced (IVP) embryos. That approach identified animals with high (76%) and low (11%) aggregate pregnancy rates, and a failure in the mechanism involved in conceptus elongation and thus maternal recognition of pregnancy was suggested to be the cause of early pregnancy loss in the low fertility-classified heifers [27, 28]. We used a similar approach to select beef heifers with intrinsic differences in pregnancy loss [13]. Serial transfer of a single IVP embryo was used to classify animals as high fertile (HF; 100% pregnancy success) or subfertile (SF; < 25% pregnancy success) based on day 28 pregnancy rates. In those heifers, no difference in day 14 pregnancy rates were observed after transfer of a single *in vivo* derived embryo on day 7 post-estrus [13]. Thus, the observed difference in uterine capacity for pregnancy in those fertility-classified heifers appears to manifest after day 14 and before day 28 of pregnancy, which encompasses conceptus elongation, pregnancy recognition, and implantation for the establishment of pregnancy.

The objective here was to use HF and SF classified heifers to identify loci, genes, gene pathways and regulators associated with uterine capacity for pregnancy using genome-wide association analysis (GWAA), gene set enrichment analysis-SNP (GSEA-SNP), and gene network analysis of SNP data. This approach identified genetic factors associated with early pregnancy loss in beef cattle and provides important predictors of fertility for genomic selection.

## Materials and methods

### Study population

This study was conducted with approval from Institutional Animal Care and Use Committees of the USDA-ARS Fort Keogh Livestock and Range Research Laboratory, Washington State University, and University of Missouri. The study population used heifers that were classified based on fertility as described previously [13]. Briefly, estrus was synchronized for 269 crossbred beef (Angus x Polled Hereford) heifers that were raised and maintained at the USDA-ARS Fort Keogh Livestock and Range Research Laboratory. Heifers received a single *in vitro* produced (IVP) embryo of high quality on day 7 post-estrus. Pregnancy was determined on days 28 and 42 by ultrasound and then terminated, and the process repeated three to four times for each animal. Heifers that maintained pregnancy 100% of the time were classified as high fertile (HF; n = 30), whereas heifers that maintained pregnancy 25% or less of the time were classified as subfertile (SF; n = 55).

### DNA isolation and genotyping

Whole blood (~16 ml) was collected by tail venipuncture. Red blood cells were lysed, and the white blood cells were pelleted. DNA was extracted using the Puregene DNA extraction kit (Gentra, Minneapolis, MN) according to manufacturer’s instructions. DNA quality and quantity were determined using a NanoDrop 1000 spectrophotometer (Thermo Scientific, Wilmington, DE). Genotyping was conducted by Neogen Laboratories (Lincoln, NE) using the Illumina BovineHD genotyping BeadChip (San Diego, CA), which contains 777,962 SNPs with an average distance between SNPs of 3.43 kb and a median distance of 2.68 kb. Alleles and position of SNPs within the bovine reference genome were assigned using the forward strand of the UMD 3.1 reference genome (http://bovinegenome.org).

### Genome-wide association analysis

The EMMAX-GRM general mixed model was defined as: *y* = *Xβ* + *Zμ* + *ϵ*, where y is an n × 1 vector of the observed phenotypes, X is a n × q matrix of fixed effects, β is a q × 1 vector representing the levels of the fixed effects, and Z is a n × t matrix relating the instances of the random effect to the phenotypes. Assumptions of the model are that Var(u) = σ_g_^2^K and Var(ϵ) = σ_e_^2^ resulting in Var(y) = σ_g_^2^ZKZ′ + σ_e_^2^I. In this analysis, Z is the identity matrix I and K is the matrix of pairwise genetic relationships among all samples. The mixed model equation was solved using a generalized least square solution with variance components (σ^2^_g_ and σ^2^_e_) [29–31]. Estimates of variance components were calculated using the EMMA approach as previously described [32], and stratification was controlled using the genomic relationship matrix [31] calculated from the genotypes.

Quality control filtering of the samples resulted in the removal of four SF heifers from the analyses due to a low genotyping call rate (< 0.9), and one HF heifer due to an X chromosome abnormality. Quality control filtering of SNPs removed 59,365 SNPs with a call rate < 0.9 and 106,608 SNPs with minor allele frequency < 0.01. A Hardy-Weinberg test of equilibrium was computed for the remaining 611,989 SNPs and 53 highly skewed (p < 1 × 10^−15^) SNPs were removed from the analysis. After quality control filtering, 80 heifers (29 high fertile and 51 subfertile) with 611,936 individual SNPs remained for the final analysis.

**Fig 1:**
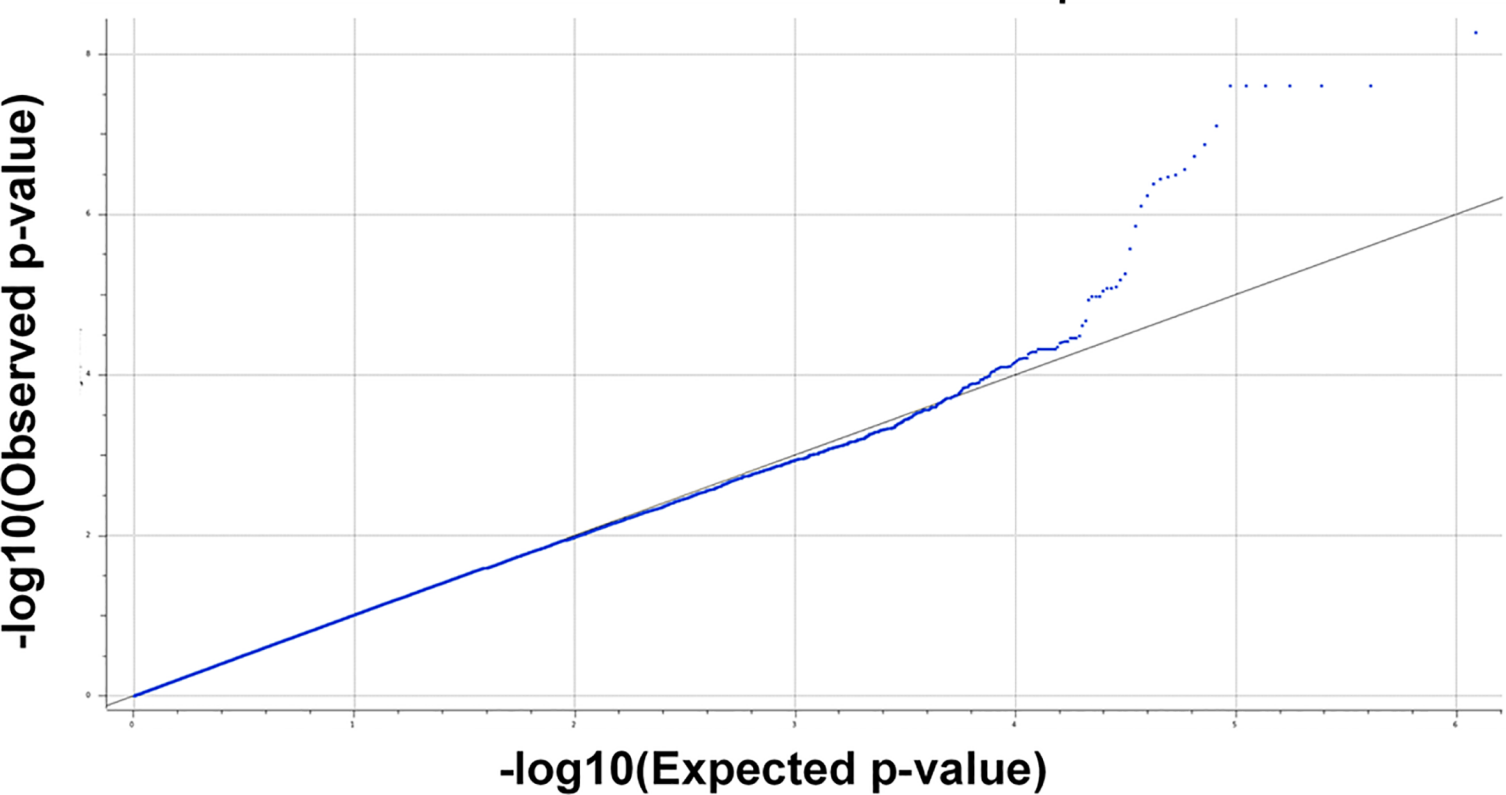
Efficient mixed-model association eXpedited with a genomic relationship matrix (EMMAX-GRM) quantile-quantile (q-q) plot of expected (X axis) versus observed (Y axis)–log_10_ transformed p values for the genome wide association analysis. The λ_GC_ = 1.01.

A quantile-quantile plot was created to compare the expected versus the observed p-values for the EMMAX-GRM mixed model (Fig 1). Based on the quantile-quantile plot and the genomic control factor (λ_GC_ = 1.01), the population stratification was adequately controlled.

The Wellcome Trust Case Control Consortium significance threshold recommendation for unadjusted p-values was used to determine if there was evidence of an association between SNPs and fertility in the GWAA [33]. A strong association was identified as SNPs with p < 5 × 10^−7^, and a moderate association was identified with p ≤ 1 × 10^−5^ and p ≥ 5 × 10^−7^. Positional candidate genes were identified around 14.2 kb of the associated SNPs based on the average haplotype block size identified in the crossbred heifers. Haplotype blocks were estimated with SNP allele correlations of > 0.9 in the SNP Variation Suite 8.3.1 software (Golden Helix, Bozeman, MT) as described [34].

### Gene set enrichment analysis (GSEA)

Gene set enrichment analysis of SNP data (GSEA-SNP) was performed using the GenGen software package [35]. SNPs were first mapped to the genes based on the UMD 3.1 assembly (Ensembl 83, *Bos taurus* UMD3.1). The most significant SNP present within the gene or surrounding the gene (within an average haplotype block size of 14.2 kb) was used as the gene proxy for 19,723 genes. The SNPs serving as gene proxies were compiled into 4,389 gene sets from Gene Ontology (GO), Reactome, Kyoto Encyclopedia of Genes and Genomes (KEGG), Biocarta and Panther. Gene sets from GO consisted of 324 cellular compartment, 447 molecular function, and 2376 biological processes specific to *Bos taurus* from level 5 of the hierarchical tree obtained from the GO2MSig database [36]. In addition to the GO gene sets, 674 gene sets were included from Reactome [37], 186 gene sets were included from KEGG [38], 217 gene sets were included from Biocarta [39], and 165 gene sets were included from Panther [40]. All SNPs were ranked by their significance of association with heifer fertility and running sum statistics and enrichment scores (ES) were computed for each gene set based on the SNP ranking.

Enrichment scores provided a measure of whether a gene set was enriched or associated with fertility. The ES increased when a ranked gene was encountered that was associated with fertility in a gene set and decreased when the gene in the gene set was not associated with fertility. The ES was calculated by using statistics similar to a weighted Kolmogorov-Smirnov-like statistic [35]. Leading edge genes were those genes that were most significant within the gene set and contributed positively to the ES prior to the peak ES for the gene set. The null distribution of ES was calculated by conducting 10,000 phenotype-based permutation tests in GenABEL in R [41]. To account for differences in the number of genes in each gene set, normalized enrichment score (NES) were computed. A gene set was defined as significant if NES was > 3.0.

### Network analysis

To identify relationships among GWAA positional candidate genes and GSEA-SNP leading edge genes, gene network analysis (upstream and master regulators analysis) was conducted using Ingenuity Pathways Analysis (IPA, Ingenuity® Systems, www.ingenuity.com). For this analysis, 262 genes (9 from GWAA and 253 from GSEA-SNP) were investigated to determine upstream and master regulators using a Fisher’s exact test with p < 0.001 as the significance threshold. The upstream regulator analysis identified regulators that controlled multiple genes through direct or indirect relationships. Master regulators are molecules that are indirectly connected to genes in the data set through upstream regulators. Details on IPA methodology have been previously described [42].

## Results and discussion

### Genome-wide association analysis (GWAA)

**Fig 2:**
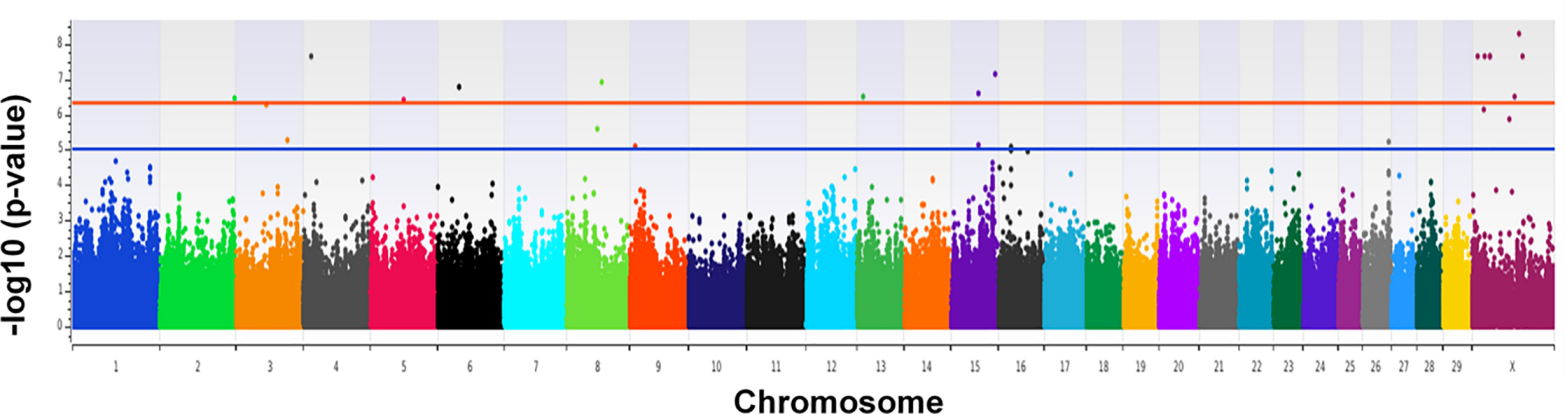
Manhattan plot of loci associated with fertility in a genome-wide association analysis using an efficient mixed-model association eXpedited with a genomic relationship matrix (EMMAX-GRM). The blue horizontal line represents the Wellcome Trust threshold for moderate association (p < 1 × 10^−5^) and the red line represents the threshold for loci that are highly associated (p < 5 × 10^−7^) with fertility. The–log_10_ p-values are indicated on the Y axis, and the bovine chromosomes are listed on the X axis.

GWAA identified 14 loci strongly associated (p < 5 × 10^−7^) and 8 loci moderately associated (5 x 10^−7^ ≥ p ≤ 1 x 10^−5^) with heifer fertility (Fig 2 and Table 1). Five loci (4 strongly and one moderately associated) have positional candidates with established physiological functions in fertility.

**Table 1:** Quantitative trait loci (QTL) associated with heifer fertility after conducting efficient mixed-model association eXpedited with a genomic relationship matrix (EMMAX-GRM) genome-wide association analysis.

Chromosome (location Mb)^1^	No. assoc. SNPs^2^	Most significant SNP^3^	p-value^4^	Positional candidate genes^5^
BTAX (85–86)	1	*rs136065255*	5.36 × 10^−9^	***KIF4A***
BTA4 (13–14)	1	*rs133805454*	2.46 × 10^−8^	*LOC101906257*
BTAX (10–11)	1	*rs133917870*	2.46 × 10^−8^	*LOC784453*
BTAX (22–23)	1	*rs137276999*	2.46 × 10^−8^	-
BTAX (32–33)	2	*rs110611558*	2.46 × 10^−8^	*LOC527384* *LOC104968419* *LOC101906593*
BTAX (91–92)	1	*rs109010586*^*§*^	2.46 × 10^−8^	*SSX5*
BTA15 (80–81)	1	*rs134238092*	7.72 × 10^−8^	-
BTA8 (63–64)	1	*rs137256869*	1.33 × 10^−7^	***TDRD7***
BTA6 (38–39)	1	*rs109381958*	1.85 × 10^−7^	*LOC104972728*
BTA15 (49–50)	2	*rs136705869*	2.70 × 10^−7^	*LOC507882* *LOC104969870* *HBG*
BTA13 (11–12)	1	*rs135282493*	3.20 × 10^−7^	*LOC526041* *LOC101906005*
BTAX (77–78)	1	*rs133484561*	3.40 × 10^−7^	-
BTA2 (135–136)	1	*rs133264693*	3.59 × 10^−7^	***PADI1***
BTA5 (60–61)	1	*rs133409493*	4.12 × 10^−7^	-
BTA3 (55–56)	1	*rs110729748*	5.77 × 10^−7^	*KYAT3*
BTAX (21–22)	1	*rs136002182*	7.72 × 10^−7^	-
BTAX (68–69)	1	*rs135028185*	1.39 × 10^−6^	-
BTA8 (55–56)	1	*rs137470071*	2.66 × 10^−6^	-
BTA3 (92–93)	1	*rs43354785*	5.39 × 10^−6^	-
BTA26 (47–48)	1	*rs135362164*	6.56 × 10^−6^	***DOCK1***
BTA9 (0–1)	2	*rs136246790*	8.29 × 10^−6^	*COL19A1*
BTA16 (21–22)	4	*rs137047555*	8.94 × 10^−6^	***SPATA17***

^1^Chromosome location of the QTL followed by the location of SNP in megabases, as measured in the UMD 3.1 reference genome assembly (http://bovinegenome.org/?q=node/61; accessed 4 April 2017)

^2^Number of significant SNPs in this genomic region associated with heifer fertility

^3^Reference sequence (rs) SNP identification number, assigned to markers submitted to the National Center for Biotechnology Information SNP database (https://www.ncbi.nlm.nih.gov/projects/SNP/; accessed 4 April 2017) for the SNP with highest significance for the QTL

^4^p-value associated with the most significant SNP for the QTL

^5^Positional candidate genes were defined as genes located ±14.2 kb of the associated SNP(s) within a QTL. **Bolded** gene names represent genes where SNP within the QTL are located within the intron of the gene

^*§*^ flagged in new annotation UMD 3.1.1 as its location is in question

The most significant locus (*rs136065255*; p = 5.36 × 10^−9^) was on *Bos taurus* X chromosome (BTAX, Table 1) and located within an intron of the kinesin family member 4A (*KIF4A*) gene. *KIF4A* is a microtubule-based motor protein and involved in many crucial cellular processes including cell division [43]. Transcript levels of *KIF4A* and other kinesin family member genes were higher in receptive endometrium compared to non-receptive endometrium at day 7 post-estrus in Simmental heifers [44]. Similarly, *KIF4A* and other kinesins, are associated with progesterone effects on the endometrium in humans [45]. The exact role of *KIF4A* in uterine function is unknown, but *KIF4A* may play an important role in cell proliferation associated with endometrial function during the estrous cycle and pregnancy.

Another strongly associated locus (*rs137256869*; p = 1.33 × 10^−7^) was on BTA8 within an intron of the tudor domain containing 7 (*TDRD7*) gene (Table 1). *TDRD7* is a protein-coding gene that encodes a component of specific cytoplasmic RNA granules involved in post-transcriptional regulation of specific genes and probably acts by binding to specific mRNAs and regulating their translation [46, 47]. In cattle, *TDRD7* expression in the endometrium is upregulated by the conceptus in both HF and SF heifers (TE Spencer, personal communication), however its function in the endometrium is not known.

The third locus (*rs136705869)* strongly associated (p = 2.7 × 10^−7^) with fertility was located on BTA15 and resulted in a missense variant (leucine to proline) in *LOC507882* (Table 1). While *LOC507882* (olfactory receptor 51F1-like) has not been characterized in cattle, its coding region resembles olfactory receptors in humans that have diverse roles including chemosensation, chemotaxis, cell-cell communication during embryogenesis, tissue growth and regeneration, and cancer in non-olfactory tissues [48].

An additional positional candidate gene, hemoglobin gamma (*HBG*), also known as fetal hemoglobin, is located within 10 kb upstream of *rs136705869*. Humans and cattle have gamma instead of beta fetal hemoglobin, which have a higher affinity for oxygen than adult hemoglobin, thereby assisting fetal hemoglobin in extracting the oxygen from the maternal hemoglobin [49]. Since the uterine environment is hypoxic, this gene might play an important role in oxygen transport within the endometrium [50, 51]; however, the function of HBG in the uterus is unknown.

The fourth locus (*rs133264693*) strongly associated (p = 3.59 × 10^−7^) with fertility was located on BTA2 within the intron of peptidylarginine deiminase 1 (*PADI1*). This gene encodes a member of the peptidyl arginine deiminase family of enzymes, which catalyzes the post-translational deimination of proteins by converting arginine residues into citrullines in the presence of calcium ions [52]. The peptidyl arginine deiminase family members have distinct substrate specificities and tissue-specific expression patterns. The type I enzyme is involved in the late stages of epidermal differentiation in keratinocytes as well as histone modifications affecting chromatin regulation and gene expression during early embryonic development in mice [53]. Although, *PADI1* is expressed in mouse and human uterus [52, 54], its function in cattle endometrium is unknown. Expression of peptidylarginine deiminase in mouse uterus is also under the control of estrogen [54], and thus may be involved in endometrial function.

One locus moderately associated (*rs135362164*; p = 6.56 × 10^−6^) with fertility was located on BTA26 and within an intron of dedicator of cytokinesis 1 (*DOCK1*). *DOCK1* is a guanine nucleotide exchange factor for small Rho family G proteins; the encoded protein regulates the small GTPase Rac1, thereby influencing several biological processes including phagocytosis and cell migration [55, 56]. *Rac1* is involved in apoptotic clearance and epithelium development in mouse uterus [55, 57]. In mice, conditional deletion of *Rac1* resulted in implantation failure due to loss of uterine luminal epithelial integrity [57].

Together, these positional candidates have potential diverse biological roles in the endometrium of the uterus that would be important for its function and establishment of pregnancy. Accordingly, mutations in the candidate genes or the regulation of these genes could result in implantation failure and early pregnancy loss. Four of the candidates (*KIF4A*, *TDRD7*, *PADI1*, and *DOCK1*) are intronic variants, and one (olfactory receptor 51F1-like *LOC507882*) is a missense variant. These loci are excellent candidates for future research to identify the causal mutations associated with fertility and pregnancy success in cattle.

### Gene set enrichment analysis (GSEA)

GSEA-SNP identified six GO and three KEGG gene sets associated with heifer fertility (NES > 3) (Table 2). The nine gene sets included three gene sets and 177 leading edge genes involved in embryonic development, three gene sets and 64 leading edge genes involved in cell signaling, and three gene sets and 119 leading edges that did not share a common function.

**Table 2:** Gene sets associated with heifer fertility from gene set enrichment analysis–SNP.

Gene set name (Database unique identifier)^1^	# Genes^2^ (# LEGS)^3^	NES^4^	Leading edge genes^5^
Epithelial cell differentiation (GO:0030855)	121(53)	3.49	***TDRD7***, *MEF2C*, *ADIPOQ*, *IRF6*, *FOXP1*, *ELF5*, *TFAP2A*, *RAPGEF2*, *CTSB*, *ANXA7*, *BCCIP*, *CDH3*, *ST14*, *KRT17*, *STAT5A*, *EHF*, *UPK2*, *BLOC1S6*, *BMP4*, *MAFF*, *GNA11*, *ACADVL*, *SMARCA4*, *RAB13*, *TMEM231*, *NFIB*, *PDE2A*, *FRZB*, *UPK1B*, *KRT5*, *GATA3*, *AIMP2*, *INTU*, *RBPJ*, *SULT1B1*, *GJA1*, *TGFB1I1*, *TOLLIP*, *CTNNB1*, *WNT16*, *STAT5B*, *F11R*, *NME2*, *ID2*, *STK4*, *PPARG*, *PTER*, *KIT*, *TGM3*, *UPK1A*, *E2F8*, *RAC1*, *TAGLN*
Heart morphogenesis (GO:0003007)	44 (25)	3.41	*MEF2C*, *SEMA3C*, *TFAP2A*, *HEY1*, *IFT57*, *BMP4*, *HAND1*, *SMARCA4*, *PTCD2*, *HAS2*, *WNT2*, *CPE*, *GATA3*, *CAV3*, *RBPJ*, *TNNC1*, *GJA1*, *CTNNB1*, *WNT16*, *EFNA1*, *ID2*, *NPY1R*, *COL4A3BP*, *CITED2*, *S1PR1*
Ether lipid metabolism (KEGG:00565)	33 (16)	3.28	*PPAP2B*, *PLA2G2F*, *PLA2G2C*, *PLD1*, *PAFAH1B2*, *PLA2G2A*, *PLA2G5*, *ENPP6*, *PAFAH1B1*, *ENPP2*, *PLA2G3*, *PLA2G6*, *PLA2G12A*, *PLA2G12B*, *PLA2G10*, *AGPS*
Fc epsilon RI signaling pathway (KEGG:04664)	79 (35)	3.26	*PRKCA*, *VAV1*, *PLA2G2F*, *PLA2G2C*, *MAPK10*, *PLCG2*, *PLA2G2A*, *PIK3R1*, *PLA2G5*, *VAV2*, *IL5*, *AKT3*, *MAP2K4*, *MAP2K6*, *MAPK3*, *PIK3R2*, *MAPK9*, *SOS1*, *PLA2G3*, *INPP5D*, *PLA2G6*, *RAF1*, *MAP2K7*, *PLA2G12A*, *PLCG1*, *PIK3CA*, *FCER1A*, *PLA2G12B*, *MAPK11*, *MAPK12*, *PLA2G10*, *TNF*, *PRKCB*, *FYN*, *PIK3R3*
Voltage gated potassium channel complex (GO:0008076)	11 (9)	3.25	*KCNJ2*, *DPP6*, *KCNH1*, *KCNB2*, *KCNMB1*, *KCNQ3*, *KCNV1*, *KCNMA1*, *KCNIP3*
Epithelium development (GO:0060429)	216 (99)	3.21	***TDRD7***, *MEF2C*, *ADIPOQ*, *IRF6*, *FOXP1*, *ELF5*, *SEMA3C*, *TFAP2A*, *ATP2C1*, *EDA*, *TP53*, *RAPGEF2*, *CTSB*, *ANXA7*, *BCCIP*, *RDH10*, *CDH3*, *IFT57*, *ST14*, *KRT17*, *TBX6*, *GPR161*, *STAT5A*, *EHF*, *UPK2*, *BLOC1S6*, *BMP4*, *LGR4*, *KRT71*, *MAFF*, *HAND1*, *GNA11*, *CSK*, *ACADVL*, *SMARCA4*, *C15H11ORF34*, *RAB13*, *TMEM231*, *NFIB*, *PDE2A*, *FRZB*, *PHACTR4*, *IL6*, *TNF*, *UPK1B*, *BDNF*, *KRT5*, *WNT2*, *GATA3*, *AIMP2*, *PRKACA*, *INTU*, *CAV3*, *RBPJ*, *SULT1B1*, *GJA1*, *TGFB1I1*, *TOLLIP*, *CTNNB1*, *WNT16*, *ZNF750*, *STAT5B*, *F11R*, *NME2*, *PSEN2*, *ID2*, *COL4A1*, *STK4*, *FST*, *CITED2*, *PPARG*, *PTER*, *KIT*, *TGM3*, *UPK1A*, *TGM2*, *EDNRA*, *ADM*, *E2F8*, *RAC1*, *GNAS*, *TAGLN*, *AQP1*, *TRAF6*, *IGF1R*, *CTSH*, *KITLG*, *ZDHHC21*, *ERCC3*, *LIN7C*, *CALB1*, *TMEM100*, *GATA5*, *KLHL3*, *TFDP1*, *CCL11*, *LMO4*, *FGF2*, *HEYL*
Cell projection organization (GO:0030030)	203 (86)	3.20	*EXT1*, *DNM2*, *MEF2C*, *FOXP1*, *SEMA3C*, *RAPGEF2*, *TTC30A*, *ATP6V0D1*, *CAPZB*, *CAPRIN1*, *TTLL1*, *IFT57*, *C7H5ORF30*, *VDAC3*, *TBX6*, *GHRL*, *MYH10*, *BLOC1S6*, *SNAP25*, *GAP43*, *PLA2G3*, *CCDC113*, *APP*, *MEF2A*, *RAB13*, *PQBP1*, *PICK1*, *CDK5R1*, *TMEM231*, *NFIB*, *CBY1*, *UCHL1*, *SNX2*, *IL6*, *ISPD*, *CDC42EP1*, *CDC42EP2*, *FYN*, *BDNF*, *GATA3*, *APOD*, *ARPC2*, *SLC39A12*, *INTU*, *AIF1*, *FLOT1*, *ASAP1*, *SDC2*, *GJA1*, *ATL1*, *CHN1*, *SCN1B*, *POC1A*, *STMN3*, *SNX10*, *MYO10*, *STMN1*, *TTC30B*, *EFNA1*, *GSN*, *CDC42*, *PFN2*, *DPYSL2*, *CCDC151*, *PRKCSH*, *SLC9A3R1*, *KIT*, *S1PR1*, *BLOC1S3*, *SS18L1*, *WASF2*, *ARFIP2*, *IFT43*, *RAC1*, *PTPDC1*, *NME7*, *RAC2*, *TMEM17*, *EMP2*, *NCS1*, *GPM6A*, *IFT27*, *LRRC6*, *CEP41*, *S100B*, *ARF1*
Glycosaminoglycan metabolic process (GO:030203)	24 (13)	3.17	*EXT1*, *EXT2*, *DSE*, *ITIH5*, *ITIH1*, *ITIH3*, *ITIH4*, *DCN*, *SLC35D1*, *IMPAD1*, *HAS2*, *CLTC*, *LYVE1*
Basal cell carcinoma (KEGG:05217)	55 (24)	3.13	*SUFU*, *FZD3*, *FZD1*, *TP53*, *FZD9*, *WNT8B*, *WNT7A*, *HHIP*, *WNT2B*, *PTCH2*, *WNT11*, *BMP4*, *GLI2*, *DVL2*, *TCF7L2*, *GSK3B*, *APC*, *AXIN2*, *FZD10*, *WNT2*, *CTNNB1*, *FZD6*, *PTCH1*, *WNT16*

^1^Database source, GO = Gene Ontology and KEGG = Kyoto Encyclopedia of Genes and Genomes

^2^Number of genes present in the gene set

^3^Number of leading edge genes in the gene set

^4^NES is the normalized enrichment score, which is the enrichment score adjusted by the number of genes in the gene set

^5^Leading edge genes are genes that contributed positively to the enrichment score for fertility; **Bolded** gene (***TDRD7)*** was both associated in the genome wide association analysis and was a leading edge gene in the gene set enrichment analysis-SNP

The GO biological processes enriched in gene sets involved in embryonic development included: epithelium development (GO:0060429); epithelial cell differentiation (GO:0030855); and heart morphogenesis (GO:0003007) (Table 2). There were ten overlapping leading edge genes (*BMP4*, *CTNNB1*, *GATA3*, *GJA1*, *ID2*, *MEF2C*, *RBPJ*, *SMARCA4*, *TFAP2A*, *WNT16*) across the three gene sets, and many of those have an established role in uterine function and blastocyst implantation in mice. One of the shared leading edge genes, gap junction protein alpha 1 (*GJA1*), also known as connexin 43 (*CX43*), has an important role in epithelial cell integrity and communication in the uterus of mice and cattle [44, 58]. In the endometrium of heifers classified on fertility, endometrial gene expression of *GJA1* was lower in receptive endometrium compared with non-receptive endometrium in day 7 post-estrus in beef heifers [44]. Another of the shared leading edge genes, recombination signal binding protein for immunoglobulin Kappa J (*RBPJ)* is required for normal embryonic development in mice, as uterine-specific deletion of this gene resulted in embryonic loss due to abnormal placentation involving estrogen receptor signaling and matrix metalloproteinase in a Notch pathway-dependent manner [59].

The epithelium development gene set consisted of 216 genes (99 leading edge genes) that are involved in the differentiation of epithelium from its initial stages to its mature form. In the epithelial cell differentiation gene set, 121 genes (53 leading edge genes) are involved in specialization of the epithelial cells. There was significant overlap between these two gene sets as all 53 leading edge genes in epithelial cell differentiation were part of the 99 leading edge genes in the epithelium development gene set. Epithelial cells proliferate and differentiate during the estrous cycle and early pregnancy to prepare for conceptus implantation [60]. One leading edge gene found in both the epithelial cell differentiation and epithelium development gene sets was *TDRD7*, a strongly associated (p = 1.33 × 10^−7^) GWAA positional candidate gene (Table 1). Another leading edge gene in this gene set was *Rac1*, which is essential for uterine luminal epithelial integrity and involved in embryo implantation in mice [55, 57]. Depletion of uterine *Rac1* during implantation elicited defective junction remodeling and epithelial polarity resulting in implantation failure in mice [57]. Both gene and protein expression of another leading edge gene interferon regulator factor 6 (*IRF6*) was increased in endometrium of higher fertility beef heifers [44]. In sheep, *IRF6* is expressed in endometrial epithelia during early pregnancy [61]. However, function of IRF6 in the ruminant uterus has not been established.

Another leading edge gene in this gene set is insulin-like growth factor 1 receptor (*IGF1R*), which is a receptor for *IGF1* and *IGF2*, and all three are expressed in the endometrium of cattle during early pregnancy [62]. In dairy cattle, treatment with bovine somatotropin increases fertility, which is likely mediated by increases in IGF1 [63]. Of note, polymorphisms in *IGF1R* were associated with a greater pregnancy rate in Luxi and Chinese Holstein cattle [62,64]. Similarly, another leading edge gene, collagen type IV alpha 1 chain (*COL4A1*), is upregulated in day 18 endometrial caruncle tissue in pregnant cattle suggesting a role for *COL4A1* in remodeling of the uterus during pregnancy for conceptus implantation and placentation [62]. Other leading edge genes in this pathway are described in Table 2.

Heart morphogenesis, the final gene set in the embryonic development category, contains 44 genes which includes 25 leading edge genes. One of the leading edge genes in this gene set, semaphorin-3c (*SEMA3C*), is also associated with fertility in a dairy cattle genome-wide association study [65]. *SEMA3C* activates the *RAC1* mediated *NFKB* signaling pathway to promote cell survival [66] in tumor cells. Since RAC1 regulates uterine remodeling and implantation events [55, 57], *SEMA3C* might be involved during these processes.

Two KEGG (ether lipid metabolism KEGG:00565 and Fc epsilon RI signaling KEGG:04664) and one GO (glycosaminoglycan metabolic process GO:030203) gene sets enriched for heifer fertility were involved in cell signaling processes. Ether lipid metabolism contains 33 genes (including 16 leading edge genes) that are involved in chemical reactions with ether lipids. The Fc epsilon RI signaling gene set contains 79 genes (including 35 leading edge genes) that controls activation of basophils and mast cells, and contributes to IgE-mediated antigen presentation [38]. The GO glycosaminoglycan metabolic process gene set contains 24 genes (including 13 leading edge genes) that are involved in chemical reactions and pathways comprising amino sugars [67]. There were no common leading edge genes among all three gene sets involved in this category but nine leading edge genes (*PLA2G10*, *PLA2G12A*, *PLA2G12B*, *PLA2G2A*, *PLA2G2C*, *PLA2G2F*, *PLA2G3*, *PLA2G5*, and *PLA2G6*) were shared between ether lipid metabolism and Fc epsilon RI signaling gene sets (Table 2). The nine genes shared between the cell signaling gene sets are members of the phospholipase A2 (*PLA2*) enzyme gene family that provides arachidonic acid for the synthesis of prostaglandins (PG), which are essential during the preimplantation period. Prostaglandins are present in bovine uterine lumen and are important for conceptus elongation and implantation in cattle [19], sheep [68] and mice [69]. Cytoplasmic *Pla2* null mice have reduced fertility resulting in small litter sizes compared to healthy controls, likely due to the disruption of PG synthesis [69]. *PLA2G2F* was also differentially expressed (log2 fold change = 3.84, p = 0.04) between the high fertile and subfertile beef heifers described in this study, suggesting that this leading edge gene may alter fertility through differences in its gene expression [13].

The Fc epsilon RI signaling gene set contained eight mitogen activated protein kinase (MAPK)-related leading edge genes (*MAP2K4*, *MAP2K6*, *MAP2K7*, *MAPK10*, *MAPK11*, *MAPK12*, *MAPK3*, and *MAPK9*). MAPK is utilized by various cytokines and growth factors to facilitate cell proliferation, differentiation and apoptosis [70]. Additionally, MAPKs interact with intracellular signaling pathways such as steroid receptors and contribute to uterine cell proliferation [71]. Cell proliferation, differentiation and apoptosis in the endometrium is critical for normal function and pregnancy. Another gene set in this category, glycosaminoglycan metabolic process, contains leading edge genes that are members of the inter-alpha-trypsin inhibitor heavy chains (*ITIH1*, *ITIH3*, *ITIH4*) gene family. These genes function as hyaluronic binding proteins and are implicated in conceptus elongation and implantation in pigs [72].

The three gene sets enriched for fertility that remain include: basal cell carcinoma gene set (KEGG:05217), voltage gated potassium channel complex (GO:0008076), and cellular component cell projection organization (GO:0030030). There were no shared leading edge genes among these three gene sets, which is expected given their diverse biological functions. The basal cell carcinoma gene set contains 55 genes (including 24 leading edge genes) that are involved with skin cancer and associated with activation of sonic hedgehog signaling which promotes cell proliferation [38]. Many of the leading edge genes in this gene set were members of the frizzled (*FZD*) gene family (*FZD1*, *FZD3*, *FZD6*, *FZD9* and *FZD10*) or a member of the wingless-related integration sites (*WNT*) family (*WNT7A*, *WNT2*, *WNT2B*, *WNT8B*, *WNT11* and *WNT16*). Members of the *FZD* gene family encode proteins that are receptors of *WNT* signaling pathways that regulate preimplantation uterine environment in cattle [73], sheep [74], mice and humans [75, 76]. Increased expression of Dickkopf1 (*DKK1*), a WNT antagonist, was differentially expressed in the endometrium of high fertile beef heifers [77]. *DKK1* encode proteins that bind to FZD receptors and inhibit *WNT* signaling pathways [78]. In addition, *DKK1* expression was also downregulated in the endometrium of lactating dairy cows [79], and addition of DKK1 to cultured bovine embryos increased their competence to establish pregnancy [80]. Another leading edge gene, *WNT7A*, can increase blastocyst development rates *in vitro* [81]. Similarly, *WNT7A* is expressed in the endometrial epithelium of sheep and implicated in conceptus elongation and implantation [74]. In addition to involvement in the *WNT* signaling pathways, WNT proteins are also involved in cell proliferation, differentiation and migration in the endometrium during implantation [76, 82].

The voltage gated potassium channel complex gene set contains 11 genes (including 9 leading edge genes) that function in the formation of transmembrane channels where potassium ions cross in response to a change in the cell membrane potential [67]. Voltage gated potassium channels are expressed in uterine smooth muscle and play a role in modulating uterine contractility during pregnancy in mice and humans [83, 84].

Finally, the cell projection organization gene set contains 203 genes (including 86 leading edge genes) responsible for cellular assembly, disassembly, and arrangement of processes such as flagellum or axons extending from a cell (Table 2) [67]. Four leading edge genes (*GJA1*, *RAC1*, *SEMA3C*, *PLA2G3*) have roles in implantation and have been previously described. One of the leading edge genes in this gene set included interleukin 6 (*IL6*) which is a multifunctional cytokine and is associated with immune response, host defense, miscarriages and hematopoiesis [85]. *IL6* has a diverse role in the maternal immune response, uterine remodeling, angiogenesis and placental development [85].

Out of 253 unique leading edge genes in nine gene sets associated with heifer fertility, six genes (*CTNNB1*, *GJA1*, *BMP4*, *WNT2*, *WNT16*, and *GATA3*) were present in three or more gene sets (Table 3). These six genes are connected through catenin beta-1 (*CTNNB1*), also known as beta-catenin. *CTNNB1* is involved in the regulation of *GJA1*, *BMP4*, and *GATA3* whereas *CTNNB1* has a feedback loop with *WNT2* and *WNT16* in the WNT (wingless) signaling pathway. *CTNNB1* plays a crucial role in adherens junctions in cell-cell adhesions and is the central effector in the canonical *WNT* signaling pathway [82]. As previously described, *WNT* signaling is likely an important functional mediator of early pregnancy events in cattle, sheep, mice and humans [73–82].

**Table 3:** Leading edge genes present in three or more gene sets associated with heifer fertility.

Leading edge genes^1^	Gene sets enriched for heifer fertility^2^						
	Epithel-ial cell differen-tiation	Heart morphogenesis	Ether lipid meta-bolism	Fc epsilon RI signal-ing	Epithe-lium develop-ment	Cell projection organiza-tion	Basal cell carci-noma
*BMP4*	X	X			X		X
*CTNNB1*	X	X			X		X
*GATA3*	X	X			X	X	
*GJA1*	X	X			X	X	
*MEF2C*	X	X			X	X	
*WNT16*	X	X			X		X
*BLOC1S6*	X				X	X	
*FOXP1*	X				X	X	
*ID2*	X	X			X		
*IFT57*		X			X	X	
*INTU*	X				X	X	
*KIT*	X				X	X	
*NFIB*	X				X	X	
*PLA2G3*			X	X		X	
*RAB13*	X				X	X	
*RAC1*	X				X	X	
*RAPGEF2*	X				X	X	
*RBPJ*	X	X			X		
*SEMA3C*		X			X	X	
*SMARCA4*	X	X			X		
*TFAP2A*	X	X			X		
*TMEM231*	X				X	X	
*WNT2*		X			X		X

^1^Leading edge genes are genes that contributed positively to the enrichment score for fertility in ≥ three gene sets enriched for fertility (NES >3)

^2^Gene sets in which leading edge genes were identified are marked

Together, the leading-edge genes in these gene sets play critical roles in uterine control of embryo development including tissue remodeling, cell proliferation, cell migration, decidualization and attachment. Spatiotemporal changes in expression level of these genes may have important roles in maintaining receptivity of the bovine uterus. Mutations that modify any of these processes have potential to affect early embryonic attachment and implantation in cattle.

### Network analysis

Network analysis of 262 positional candidates and leading edge genes identified three top upstream regulators (tumor necrosis factor or TNF, beta estradiol, and tumor protein P53 or TP53) that are directed by the master regulator SOX2-OCT4 (sex determining region Y box 2 –octamer-binding transcription factor 4) complex (Table 4).

**Table 4:** Upstream and master regulators of positional candidates and leading edge genes associated with heifer fertility.

Upstream Regulators^1^	Molecule Type^2^	p-value^3^	Regulator Gene Targets^4^ (#)^5^
Tumor necrosis factor (TNF)	Cytokine	3.3 × 10^−17^	*ACADVL*, *ADIPOQ*, *ADM*, *APC*, *APP*, *AQP1*, *AXIN2*, *BDNF*, *BMP4*, *CCL11*, *CDC42*, *CDH3*, *CDK5R1*, *CITED2*, *COL4A3BP*, *CTNNB1*, *CTSB*, *DCN*, *EFNA1*, *EHF*, *EMP2*, *ENPP2*, *EXT1*, *F11R*, *FGF2*, *FRZB*, *FST*, *FYN*, *GHRL*, *GJA1*, *GSK3B*, *HAS2*, *IGF1R*, *IL5*, *IL6*, *INPP5D*, *KCNJ2*, *KIT*, *KITLG*, *LYVE1*, *MAFF*, *MAP2K4*, *MAP2K6*, *MEF2C*, *MYH10*, *PDE2A*, *PLA2G2A*, *PLA2G3*, *PLA2G5*, *PPARG*, *PRKCA*, *RAC1*, *SDC2*, *SEMA3C*, *STAT5A*, *STMN1*, *TAGLN*, ***TDRD7***, *TFAP2A*, *TGM2*, *TNF*, *TNNC1*, *TP53*, *TRAF6*, *WNT7A*, *ZNF750* (66)
Beta-estradiol	Hormone	1.9 × 10^−16^	*ACADVL*, *ADIPOQ*, *ADM*, *AIF1*, *APC*, *APOD*, *APP*, *AQP1*, *BDNF*, *BLOC1S6*, *BMP4*, *CALB1*, *CDC42*, *CDK5R1*, *CITED2*, *CTNNB1*, *CTSB*, *CTSH*, *DCN*, *EFNA1*, *ELF5*, *ENPP2*, *EXT2*, *FGF2*, *FLOT1*, *FST*, *GJA1*, *GLI2*, *GSK3B*, *HAND1*, *HAS2*, *ID2*, *IGF1R*, *IL6*, *IRF6*, *ITIH4*, *KIT*, *KITLG*, *KRT17*, *KRT5*, *MAP2K6*, *MEF2A*, *NPY1R*, ***PADI1***, *PIK3R1*, *PIK3R2*, *PIK3R3*, *PLA2G10*, *PPARG*, *PRKCB*, *PSEN2*, *RAC1*, *RDH10*, *S1PR1*, *SDC2*, *SEMA3C*, *SLC9A3R1*, *SNAP25*, *SOS1*, *TNF*, *TNNC1*, *TP53*, *WNT11*, *WNT7A* (64)
Tumor protein p53 (TP53)	Transcription regulator	2.1 × 10^−15^	*ACADVL*, *AKT3*, *APC*, *APP*, *AXIN2*, *CDH3*, *CITED2*, *COL4A1*, *CSK*, *CTNNB1*, *CTSB*, *CTSH*, *DNM2*, *E2F8*, *EFNA1*, *ENPP2*, *ERCC3*, *F11R*, *FGF2*, *FYN*, *GSK3B*, *GSN*, *HAS2*, *ID2*, *IGF1R*, *IL5*, *IL6*, *KCNMA1*, *KIT*, *KITLG*, *MAP2K4*, *MAP2K6*, *MAP2K7*, *MAPK12*, *MAPK3*, *MYH10*, *MYO10*, *PAFAH1B2*, *PDE2A*, *PIK3R1*, *PIK3R3*, *PPARG*, *PRKCA*, *PRKCB*, *PSEN2*, *PTCH1*, *RAF1*, *RBPJ*, *S100B*, *SEMA3C*, *ST14*, *STMN1*, *TCF7L2*, *TFDP1*, *TGFB1I1*, *TGM2*, *TNF*, *TP53*, *WNT2*, *WNT7A* (60)
**Master Regulator**^**2**^			
SOX2-OCT4 complex	Transcription regulators	1.2 × 10^−22^	*ACADVL*, *ADM*, *AIF1*, *AKT3*, *APC*, *APOD*, *APP*, *AQP1*, *ARPC2*, *AXIN2*, *BMP4*, *CAPRIN1*, *CAPZB*, *CDC42*, *CDC42EP2*, *CDH3*, *CITED2*, *CLTC*, *COL4A1*, *COL4A3BP*, *CTNNB1*, *CTSB*, *CTSH*, *DCN*, ***DOCK1***, *DPYSL2*, *DVL2*, *E2F8*, *EDA*, *EDNRA*, *EFNA1*, *EHF*, *ELF5*, *ENPP2*, *ERCC3*, *EXT2*, *F11R*, *FRZB*, *FST*, *FYN*, *FZD1*, *FZD3*, *GATA3*, *GATA5*, *GJA1*, *GLI2*, *GSK3B*, *GSN*, *HAND1*, *HAS2*, *HEY1*, *HHIP*, *ID2*, *IGF1R*, *IL5*, *IL6*, *INPP5D*, *IRF6*, *ITIH3*, *KCNMA1*, *KCNMB1*, *KCNQ3*, ***KIF4A***, *KITLG*, *KRT17*, *KRT5*, *LYVE1*, *MAP2K4*, *MAP2K6*, *MAP2K7*, *MAPK11*, *MAPK12*, *MEF2C*, *MYH10*, *NFIB*, ***PADI1***, *PAFAH1B2*, *PDE2A*, *PIK3CA*, *PIK3R1*, *PIK3R2*, *PIK3R3*, *PLA2G5*, *PLD1*, *PPARG*, *PRKACA*, *PRKCA*, *PSEN2*, *PTCH1*, *PTCH2*, *RAC1*, *RAF1*, *RBPJ*, *RDH10*, *SEMA3C*, *ST14*, *STAT5A*, *STAT5B*, *STMN1*, *STMN3*, *TBX6*, *TCF7L2*, ***TDRD7***, *TFAP2A*, *TFDP1*, *TGFB1I1*, *TGM2*, *TMEM100*, *TMEM17*, *TNF*, *TNNC1*, *TRAF6*, *UCHL1*, *WNT16*, *WNT2*, *WNT2B*, *WNT7A*, *WNT8B* (118)

^1^Upstream regulators are molecules that control multiple genes in the Ingenuity Pathway Analysis through direct or indirect relationships

^2^Molecule type of the regulator as defined by the Ingenuity Pathway Analysis

^3^P-value with Fisher’s exact test

^4^List of leading edge genes from the gene set enrichment analysis–SNP and positional candidate genes from the genome-wide association analysis (in bold) regulated by each upstream regulator

^5^Total number of genes regulated by each upstream regulator

The most significant (p = 3.3 × 10^−17^) upstream regulator, TNF, directly regulates 66 genes associated with heifer fertility. *TNF* produces a cytokine protein with systemic and local functions that are elicited in response to infection and inflammation. In the uterus, the primary source of TNF is uterine macrophages and natural killer cells. TNF also stimulates nuclear factor kappa B (NFKB) to trigger inflammation [86]. *Rac1* is an upstream regulator of P38 mitogen-activated protein kinases (*P38 MAPK)* [87] and both *Rac1* and MAP kinases are leading edge genes in this data set (Table 2). Together, TNF-RAC1-MAPK signaling events function to maintain a receptive post-implantation uterine environment.

Beta-estradiol is an upstream regulator (p = 1.9 × 10^−16^) that controls 64 genes associated with heifer fertility. Optimal circulating levels of maternal estrogen and progesterone is critical to maintain implantation in humans and mice, as an imbalance of estrogen can render the uterus unreceptive to implantation [88]. Estrogen is also essential for proliferation and differentiation of uterine epithelia during early pregnancy and involves the *WNT* signaling pathway [76]. Many *WNT* signaling genes along with beta-catenin are leading edge genes in this data set (Table 2). Previously published genome-wide association studies in dairy cattle also showed estrogen as an upstream regulator of genes associated with fertility [89, 90].

A third upstream regulator of fertility was TP53 which controls 60 genes associated with heifer fertility (p = 2.1 × 10^−15^). TP53 encodes an anti-apoptotic protein known as a tumor suppressor that regulates the cell cycle and DNA repair. However, TP53 also has a role in female fertility and implantation. Polymorphisms in intron 3 and exon 4 of TP53 were associated with endometriosis and post-*in vitro* fertilization implantation failure in women due to alterations in leukemia inhibitory factor (*LIF*) expression [91]. LIF, which is crucial for blastocyst implantation, is regulated by TP53 and expressed in endometrial glands of the mouse uterus [92]. *LIF* has a diverse role in embryo implantation including transformation of the endometrium to a receptive stage, stromal decidualization, uterine leukocyte migration and regulation of prostaglandin synthesis [93]. LIF achieves these roles through signal transduction pathways including JAK/STAT, MAPK and PI3-kinase through its binding to the LIF receptor [92].

The master regulator identified was the SOX2-OCT4 complex, which regulates 18 upstream regulators and 4 GWAA positional candidates *KIF4A*, *TDRD7*, *PADI1*, and *DOCK1* and 114 leading edge genes associated with heifer fertility (p = 1.2 × 10^−22^). Transcriptional regulators SOX2 and OCT4 are two main components of the embryonic stem cell pluripotency circuit. These factors may also be important for endometrial regeneration, repair and remodeling during pregnancy in cattle, mouse and human [94]. Regeneration of uterine glands involves stem cell proliferation and is critical for successful implantation of the bovine embryo [95]. OCT4 and SOX2 proteins were present in endometrial epithelial and stromal cells as well as myometrial cells at day 8 to 10 post-estrus in cattle. The greatest expression of *OCT4* and *SOX2* was in the stromal cells in the ipsilateral horn of the uterus [96]. Highly proliferative properties of OCT4 and SOX2 helps in the reorganization of the endometrium during embryo attachment and implantation. This control of endometrial regeneration and repair may play a crucial role in establishing and maintaining a successful pregnancy in cattle. Moreover, 668 additional upstream regulators and 772 additional master regulator molecules (p < 0.001) were identified as associated with heifer fertility and are available in S1 and S2 Tables.

## Conclusion

This study focused on the QTL and genes associated with the loss of pregnancy in cattle through failed implantation of the embryo by using *in vitro* produced embryos transferred into recipient heifers. The analysis of SNP data through GWAA, GSEA-SNP and IPA revealed QTL and candidate genes that regulate establishment of early pregnancy in cattle. These approaches facilitate our understanding of the genetics and biology of endometrial receptivity in cattle. Further validation of these results in independent populations are needed before the QTLs identified from this study can serve as fertility markers for genomic selection. Validation of these markers in independent populations will also be helpful in the identification of causal mutations, which is desirable because they provide heightened accuracy when using genomic selection, particularly in multiple breeds.

Many of the gene sets and pathways identified in this study include genes already implicated in fertility in other species. Further investigation is needed to determine the role of the key upstream and master regulators that control many of the positional candidate genes identified in this study as associated with the establishment of pregnancy. The understanding of these genes, pathways and regulators is expected to help reduce early pregnancy loss in cattle resulting in increased reproductive performance.

## Supporting information

S1 Table Upstream regulators of positional candidates and leading edge genes associated with heifer fertility. (DOCX)

S2 Table Master regulators of positional candidates and leading edge genes associated with heifer fertility. (DOCX)
