# Supplementary material for: Loci and pathways associated with uterine capacity for pregnancy and fertility in beef cattle
Source: PLoS One. 2017 Dec 11;12(12):e0188997. doi: 10.1371/journal.pone.0188997 (PMC5724891; doi:10.1371/journal.pone.0188997)
Supplement: S1 Table — (DOCX) [file pone.0188997.s001.docx]

**S1 Table. Upstream regulators of positional candidates and leading edge genes associated with heifer fertility.**

| **Upstream regulator^1^** | **Molecule type*^2^*** | **p-value^3^** | **Target molecules in dataset^4^** |
| --- | --- | --- | --- |
| *TNF* | Cytokine | 3.33 × 10^-17^ | *ACADVL, ADIPOQ, ADM, APC, APP, AQP1, AXIN2, BDNF, BMP4, CCL11, CDC42, CDH3, CDK5R1, CITED2, COL4A3BP, CTNNB1, CTSB, DCN, EFNA1, EHF, EMP2, ENPP2, EXT1, F11R, FGF2, FRZB, FST, FYN, GHRL, GJA1, GSK3B, HAS2, IGF1R, IL5, IL6, INPP5D, KCNJ2, KIT, KITLG, LYVE1, MAFF, MAP2K4, MAP2K6, MEF2C, MYH10, PDE2A, PLA2G2A, PLA2G3, PLA2G5, PPARG, PRKCA, RAC1, SDC2, SEMA3C, STAT5A, STMN1, TAGLN, TDRD7, TFAP2A, TGM2, TNF, TNNC1, TP53, TRAF6, WNT7A, ZNF750* |
| Estrogen | Chemical - endogenous mammalian | 1.9 × 10^-16^ | *ACADVL, ADIPOQ, ADM, AIF1, APC, APOD, APP, AQP1, BDNF, BLOC1S6, BMP4, CALB1, CDC42, CDK5R1, CITED2, CTNNB1, CTSB, CTSH, DCN, EFNA1, ELF5, ENPP2, EXT2, FGF2, FLOT1, FST, GJA1, GLI2, GSK3B, HAND1, HAS2, ID2, IGF1R, IL6, IRF6, ITIH4, KIT, KITLG, KRT17, KRT5, MAP2K6, MEF2A, NPY1R, PADI1, PIK3R1, PIK3R2, PIK3R3, PLA2G10, PPARG, PRKCB, PSEN2, RAC1, RDH10, S1PR1, SDC2, SEMA3C, SLC9A3R1, SNAP25, SOS1, TNF, TNNC1, TP53, WNT11, WNT7A* |
| *TP53* | Transcription regulator | 2.15 × 10^-15^ | *ACADVL, AKT3, APC, APP, AXIN2, CDH3, CITED2, COL4A1, CSK, CTNNB1, CTSB, CTSH, DNM2, E2F8, EFNA1, ENPP2, ERCC3, F11R, FGF2, FYN, GSK3B, GSN, HAS2, ID2, IGF1R, IL5, IL6, KCNMA1, KIT, KITLG, MAP2K4, MAP2K6, MAP2K7, MAPK12, MAPK3, MYH10, MYO10, PAFAH1B2, PDE2A, PIK3R1, PIK3R3, PPARG, PRKCA, PRKCB, PSEN2, PTCH1, RAF1, RBPJ, S100B, SEMA3C, ST14, STMN1, TCF7L2, TFDP1, TGFB1I1, TGM2, TNF, TP53, WNT2, WNT7A* |
| *THRB* | Ligand-dependent nuclear receptor | 4.52 × 10^-15^ | *AKT3, CTNNB1, CTSH, DCN, FRZB, FZD1, FZD6, FZD9, GSN, IGF1R, IL6, MAPK3, PLA2G2A, PPARG, STAT5B, TNF, TP53, WNT11, WNT16, WNT2, WNT2B, WNT7A, WNT8B* |
| *APP* | Other | 6.42 × 10^-15^ | *AIF1, APP, AXIN2, BDNF, BMP4, CALB1, CDC42, CDH3, CDK5R1, CITED2, CLTC, CTNNB1, CTSB, DCN, DPYSL2, ENPP2, EXT1, FGF2, FYN, FZD3, GAP43, GSK3B, HEY1, IGF1R, IL6, LMO4, MAFF, MEF2C, PAFAH1B2, PIK3R1, PLCG1, PPARG, PRKACA, PRKCA, PRKCB, PTCH2, RBPJ, S100B, S1PR1, SMARCA4, SNAP25, STMN1, TAGLN, TNF, TP53, TRAF6, UCHL1* |
| *DKK1* | Growth factor | 8.99 × 10^-15^ | *APP, AXIN2, BDNF, BMP4, CTNNB1, FGF2, GATA3, GSK3B, PPARG, TAGLN, TNNC1, TP53, WNT11, WNT7A* |
| *CTNNB1* | Transcription regulator | 6.02 × 10^-14^ | *ADIPOQ, APOD, APP, AXIN2, BMP4, CAPZB, COL4A1, CTNNB1, ENPP2, FST, FZD3, GAP43, GATA3, GJA1, GLI2, HHIP, ID2, IFT57, KIT, KRT5, PIK3R1, PPARG, PRKCSH, PTCH1, PTCH2, SDC2, SEMA3C, STAT5A, STAT5B, TCF7L2, TGM2, TNF, TP53, VAV1, WNT11, WNT16, WNT2* |
| Lipopoly-saccharide | Chemical drug | 9.84 × 10^-14^ | *ADIPOQ, ADM, ANXA7, APP, AQP1, ASAP1, ATP6V0D1, BDNF, BMP4, CCL11, CDC42EP2, CDK5R1, CITED2, COL4A1, CTNNB1, CTSB, DCN, DVL2, EHF, ENPP2, FGF2, FST, FYN, GAP43, GHRL, GJA1, GSN, HEY1, ID2, IL5, IL6, INPP5D, ITIH4, KIT, MAFF, MAP2K7, MAPK9, PIK3R1, PLA2G2A, PLA2G2F, PLA2G5, PLD1, PPARG, PRKACA, PRKCA, PTCH1, S1PR1, STAT5A, STMN1, TCF7L2, TFDP1, TGM2, TNF, TNNC1, TOLLIP, TP53, TRAF6, UCHL1, VAV1* |
| *TGFB1* | Growth factor | 1.76 × 10^-13^ | *ADIPOQ, ADM, APP, AQP1, ARPC2, BDNF, BMP4, CAPRIN1, CCL11, CDK5R1, CITED2, COL4A1, CTNNB1, CTSB, CTSH, DCN, EDNRA, EXT1, EXT2, FCER1A, FGF2, FYN, FZD1, GATA3, GJA1, GLI2, GNAS, GSN, HAS2, HEY1, ID2, IL5, IL6, INPP5D, ITIH3, KCNMB1, KCNQ3, KCNV1, KIT, KITLG, KRT17, LYVE1, MAPK3, MEF2C, MYO10, NFIB, PFN2, PPARG, PRKCA, RAC1, STAT5A, STAT5B, TAGLN, TGFB1I1, TGM2, TMEM17, TNF, TP53, WNT11* |
| *ESR1* | Ligand-dependent nuclear receptor | 2.7 × 10^-13^ | *APC, AQP1, ARFIP2, ATP6V0D1, AXIN2, BCCIP, BMP4, CALB1, CDC42, CDC42EP2, CDK5R1, CPE, CTNNB1, CTSB, DNM2, EFNA1, ENPP2, FST, GATA3, GJA1, GLI2, GNAS, GSN, IGF1R, IL5, IL6, KCNMA1, MAP2K6, MAP2K7, MAPK11, MAPK12, MYH10, NFIB, PFN2, PTCH1, PTCH2, SCN1B, SEMA3C, SLC9A3R1, SOS1, STAT5A, STAT5B, STK4, SUFU, TCF7L2, TGM2, TNF, TP53, WASF2, WNT11* |
| *SOX2* | Transcription regulator | 9.3 × 10^-13^ | *APC, AXIN2, BMP4, CITED2, CTNNB1, EXT2, FRZB, FST, GATA3, GJA1, GLI2, GSK3B, HAND1, HEY1, ID2, IRF6, KITLG, KRT17, MEF2C, PIK3R1, PPARG, RBPJ, TBX6, WNT8B* |
| Tretinoin | Chemical - endogenous mammalian | 1.05 × 10^-12^ | *ADM, APOD, APP, AQP1, BMP4, CALB1, CDC42, CDK5R1, CITED2, CLTC, COL4A1, CPE, CTNNB1, CTSB, DCN, ENPP2, ERCC3, FGF2, FST, FZD10, GAP43, GJA1, GLI2, GNAS, HAS2, HEY1, ID2, IGF1R, IL5, IL6, KCNJ2, KIT, KITLG, KRT5, MAPK10, MAPK9, MEF2C, NME2, PIK3R1, PLD1, PPARG, PRKCA, PRKCB, PTCH1, RBPJ, S100B, SMARCA4, TGM2, TNF, TP53, VAV1, VAV2, WNT2B, WNT8B* |
| U0126 | Chemical - kinase inhibitor | 1.28 × 10^-11^ | *ADIPOQ, AQP1, AXIN2, BDNF, CCL11, CDK5R1, COL4A1, CTNNB1, EHF, ENPP2, FGF2, GJA1, GSK3B, GSN, IL5, IL6, MAPK3, PLD1, PPARG, PRKCB, RAC1, RAC2, SCN1B, TCF7L2, TGM2, TNF, TP53, UPK2* |
| *CNR1* | G-protein coupled receptor | 1.81 × 10^-15^ | *ADIPOQ, BDNF, BMP4, FGF2, HEYL, IGF1R, IL6, NME2, PAFAH1B1, PLD1, RAC1, S100B, SUFU, TNF, UPK1B, UPK2* |
| *HRAS* | Enzyme | 2.93 × 10^-15^ | *ADM, APP, ARF1, ASAP1, BMP4, CAV3, CDH3, COL4A1, CTNNB1, CTSB, FGF2, FZD1, GJA1, GSN, HAS2, HEY1, ID2, IGF1R, IL6, MAPK12, MAPK3, MYH10, PRKCA, PRKCB, RAF1, TAGLN, TGFB1I1, TGM2, TNF, TP53* |
| *EBF1* | Transcription regulator | 6.9 × 10^-15^ | *ADIPOQ, AKT3, GATA3, GSK3B, IL6, INPP5D, MAPK10, PIK3CA, PIK3R1, PIK3R2, PIK3R3, PPARG, PRKACA, RAF1, SOS1* |
| PD98059 | Chemical - kinase inhibitor | 1.71 × 10^-10^ | *ADIPOQ, BDNF, CAV3, CCL11, CDC42EP1, CDH3, CDK5R1, CHN1, COL4A1, CTNNB1, CTSB, CTSH, FGF2, GAP43, GHRL, GJA1, GSK3B, HAS2, IGF1R, IL5, IL6, MAPK3, PIK3R1, PPARG, STAT5A, TNF, TP53, UPK2* |
| Budesonide | Chemical drug | 2.04 × 10^-10^ | *CCL11, CDK5R1, FYN, GNA11, IL5, IL6, MAP2K4, MAP2K6, MAP2K7, PRKACA, PRKCA, TNF* |
| *MBD3* | Enzyme | 2.37 × 10^-10^ | *APC, CTNNB1, DVL2, FZD1, FZD3, GSK3B, MEF2A, SLC9A3R1, WNT16, WNT2B, WNT7A, WNT8B* |
| *WNT3A* | Cytokine | 2.62 × 10^-10^ | *APP, AXIN2, BDNF, BMP4, CTNNB1, DCN, DVL2, FZD1, GAP43, GSK3B, HAS2, IL6, KIT, KITLG, MEF2C, PLD1, PPARG, TNF, WNT11* |
| *IFNG* | Cytokine | 3.99 × 10^-10^ | *ADIPOQ, ADM, AIF1, APP, AQP1, BDNF, CALB1, CCL11, CDK5R1, CSK, CTNNB1, CTSB, CTSH, EDNRA, EHF, F11R, FGF2, FZD1, GATA3, GJA1, GNAS, HAS2, IGF1R, IL5, IL6, KCNMA1, KITLG, KRT17, MAFF, MYH10, PLA2G2A, PLA2G5, PLCG1, PLD1, PPARG, PRKCA, PSEN2, RAC2, SNAP25, TCF7L2, TFDP1, TNF, TP53, TRAF6* |
| Nocodazole | Chemical reagent | 4.91 × 10^-10^ | *CSK, FYN, GSK3B, MAP2K4, MAP2K6, MAP2K7, MAPK12, MAPK3, PRKCB, RAF1, STK4, TP53* |
| *NFKB* complex (*NFKB1,*  *NFKB2, RELA, RELB*) | Complex | 1.9 × 10^-9^ | *APP, BDNF, CALB1, CAV3, CCL11, CTNNB1, EFNA1, EHF, FGF2, FST, GAP43, GATA3, GLI2, GNAS, HAS2, IL5, IL6, KIT, KRT17, PLD1, PPARG, PRKACA, RBPJ, STAT5A, TGM2, TNF, TOLLIP, TP53* |
| *IL1B* | Cytokine | 1.91 × 10^-9^ | *ADM, AIF1, APP, BMP4, CALB1, CCL11, CTNNB1, CTSB, DCN, EFNA1, EHF, ENPP2, FGF2, FST, GATA3, GJA1, GNAS, GSK3B, HAS2, IL6, MAP2K6, MEF2C, PLA2G2A, PLA2G3, PLA2G5, PLD1, PPARG, RAC2, S100B, SNAP25, STAT5A, TGM2, TNF, TRAF6* |
| *HDAC4* | Transcription regulator | 2.13 × 10^-9^ | *BDNF, CALB1, CHN1, FST, GPM6A, IL6, MAPK10, MEF2C, PRKCA, PRKCB, SNAP25, TAGLN, TNF* |
| Dihydro-testosterone | Chemical - endogenous mammalian | 2.3 × 10^-9^ | *ACADVL, ADIPOQ, ADM, APOD, APP, AQP1, AXIN2, COL4A1, CTNNB1, E2F8, EFNA1, FGF2, FST, FZD6, GAP43, GSN, ID2, IGF1R, IL6, KRT5, MAFF, PPARG, STAT5A, TGM2, TNF, TP53* |
| *APOE* | Transporter | 3.59 × 10^-9^ | *ADIPOQ, APOD, APP, BMP4, CTNNB1, CTSB, GATA3, IGF1R, IL5, IL6, MAP2K7, PLPP3, PPARG, PRKCB, TNF, TRAF6, WNT2* |
| *KLF2* | Transcription regulator | 4.94 × 10^-9^ | *ADM, BMP4, COL4A1, EFNA1, IL6, KITLG, MAPK9, MYH10, PLPP3, PPARG, S1PR1, TNF, TRAF6, VAV1* |
| *AGT* | Growth factor | 5.67 × 10^-9^ | *ADIPOQ, ADM, BDNF, CAV3, COL4A1, EDNRA, FGF2, GAP43, GJA1, GSK3B, HAS2, HEY1, IGF1R, IL6, KITLG, MAP2K7, MYH10, PIK3R1, PLA2G10, PPARG, RAC1, TNF, TP53* |
| Lithium chloride | Chemical drug | 5.87 × 10^-9^ | *APP, AXIN2, BMP4, CTNNB1, DCN, GATA3, GSK3B, HEYL, MAPK10, PLD1, PPARG, TP53* |
| *VCAN* | Other | 7.73 × 10^-9^ | *ADM, BDNF, CPE, DCN, ENPP2, FST, HAS2, MEF2C, MYH10, PLA2G2A, RBPJ, TCF7L2, TP53* |
| *PKC*(s)  *(PRKCA,*  *PRKCB,*  *PRKCD,*  *PRKCE,*  *PRKCG,*  *PRKCH,*  *PRKCI,*  *PRKCQ,*  *PRKCZ,*  *PRKD1,*  *PRKD3)* | Group | 8.31 × 10^-9^ | *ADM, APP, BDNF, CTNNB1, CTSB, FGF2, GAP43, ID2, IL6, IRF6, KCNJ2, MAPK3, PLA2G6, PPARG, PRKCA, TNF* |
| D-glucose | Chemical - endogenous mammalian | 1.79 × 10^-8^ | *ADIPOQ, AQP1, BMP4, CALB1, CDK5R1, COL4A1, CTSB, DCN, FGF2, FST, GHRL, GJA1, IGF1R, IL6, NPY1R, PICK1, PLA2G6, PLCG1, PPARG, PRKCB, RDH10, SNAP25, TGM2, TNF, TP53* |
| *PLG* | Peptidase | 2.17 × 10^-8^ | *ADIPOQ, BDNF, DOCK1, FYN, IL6, PLA2G5, RAC1, RAC2, TNF, VAV1* |
| *EDN1* | Cytokine | 2.19 × 10^-8^ | *ADIPOQ, CDC42, COL4A1, CTNNB1, EDNRA, FGF2, FST, GJA1, HAND1, HEY1, IL6, PRKCA, PRKCB, TGM2, TP53* |
| *IRS1* | Enzyme | 3.56 × 10^-8^ | *ADIPOQ, COL4A1, DVL2, GATA3, GJA1, ID2, MAPK12, MEF2C, PIK3R1, PLPP3, PPARG, TNNC1* |
| *STAT3* | Transcription regulator | 4.19 × 10^-8^ | *ADIPOQ, ADM, CCL11, CTSB, FGF2, FST, GAP43, GATA3, GJA1, HAS2, HEY1, ID2, IGF1R, IL5, IL6, KRT17, PLA2G10, PLA2G2A, RBPJ, S1PR1, TAGLN, TFDP1, TNF, TP53* |
| *CDK2AP1* | Other | 4.34 × 10^-8^ | *CTNNB1, FZD1, FZD3, GSK3B, SLC9A3R1, WNT16, WNT2B, WNT8B* |
| *HOXA10* | Transcription regulator | 4.36 × 10^-8^ | *ADIPOQ, ADM, AIMP2, AQP1, ENPP2, FST, FZD1, GJA1, ID2, MAPK12, PIK3R1, S100B, SOS1, TP53* |
| *MAPK14* | Kinase | 4.36 × 10^-8^ | *EFNA1, GJA1, GNAS, HAS2, IL5, IL6, KITLG, MAP2K6, MEF2C, PLA2G5, TAGLN, TGM2, TNF, TP53* |
| *PSEN1* | Peptidase | 5.49 × 10^-8^ | *APP, BDNF, CDK5R1, CLTC, CTNNB1, DPYSL2, ENPP2, GJA1, HAND1, PAFAH1B2, PIK3R1, PPARG, PSEN2, RBPJ, S100B, SNAP25, STMN1, TNF, TP53, UCHL1* |
| LY294002 | Chemical - kinase inhibitor | 5.94 × 10^-8^ | *BDNF, CDK5R1, CTNNB1, FGF2, GJA1, GSK3B, HAS2, IGF1R, IL6, MAP2K4, MEF2A, MEF2C, PIK3R1, PLA2G2A, PPARG, S1PR1, SEMA3C, SOS1, TAGLN, TNF, TP53, UPK2, WNT7A* |
| Riboflavin | Chemical - endogenous mammalian | 7.29 × 10^-8^ | *APC, FZD1, FZD6, PTCH1, SUFU, TNF* |
| Progesterone | Chemical - endogenous mammalian | 7.96 × 10^-8^ | *ADIPOQ, ADM, APOD, AQP1, BDNF, CITED2, EDNRA, EFNA1, ENPP2, FST, GAP43, GJA1, HEY1, IL5, IL6, KRT5, MYO10, NME2, NPY1R, PIK3R1, PLPP3, STAT5A, STAT5B, TNF, TP53* |
| L-triiodothyronine | Chemical - endogenous mammalian | 9.69 × 10^-8^ | *ADIPOQ, ADM, APP, COL4A1, CTNNB1, CTSH, EFNA1, ENPP2, GHRL, ID2, IGF1R, IL6, ITIH3, KRT17, NFIB, PLA2G2A, PPARG, TNF, TP53* |
| Dexa-methasone | Chemical drug | 9.82 × 10^-8^ | *ADIPOQ, ADM, APOD, APP, AQP1, AXIN2, BDNF, CALB1, COL4A1, CPE, CTNNB1, CTSB, DCN, EDA, EDNRA, EFNA1, EHF, FGF2, FST, GAP43, GATA3, GHRL, GJA1, ID2, IGF1R, IL5, IL6, KIF4A, KIT, KRT17, LMO4, LYVE1, MAPK3, PAFAH1B1, PIK3R1, PLD1, PPARG, PRKCA, PRKCB, SLC35D1, STAT5A, SULT1B1, TAGLN, TGM2, TNF, TP53, ZNF750* |
| *SMARCA4* | Transcription regulator | 9.95 × 10^-8^ | *ADIPOQ, BMP4, CTSB, CTSH, EDNRA, EHF, GPM6A, HEY1, HHIP, IL6, IRF6, KCNJ2, KCNQ3, KIT, MAFF, MEF2C, PLPP3, PPARG, PTCH1, RAC2, SDC2, SMARCA4, TAGLN, TP53, WNT7A* |
| SB203580 | Chemical - kinase inhibitor | 1.01 × 10^-7^ | *ADIPOQ, AQP1, BMP4, CAV3, CCL11, CDK5R1, EFNA1, EHF, EMP2, GJA1, IL5, IL6, MAP2K6, MAPK12, PPARG, TAGLN, TGM2, TNF, TP53* |
| Norepinephrine | Chemical - endogenous mammalian | 1.06 × 10^-7^ | *APP, BDNF, DNM2, FCER1A, FGF2, FST, HHIP, IGF1R, IL6, NPY1R, PPARG, PTCH1, TNF* |
| *MAPT* | Other | 1.15 × 10^-7^ | *AIF1, APP, BDNF, CDK5R1, CLTC, DPYSL2, FYN, IL6, MAPK11, MAPK3, PAFAH1B2, S100B, SNAP25, STMN1, TNF, UCHL1* |
| Butyric acid | Chemical - endogenous mammalian | 1.16 × 10^-7^ | *AQP1, CALB1, CDC42, COL4A1, DPP6, FYN, GATA3, GATA5, GSN, IL6, INPP5D, KIT, PLCG1, PPARG, PRKACA, PRKCB, RAF1, STAT5B, TGM2, TNF, TP53* |
| *LGALS3* | Other | 1.18 × 10^-7^ | *ADIPOQ, COL4A1, CTSH, ENPP2, IL5, IL6, PPARG, PRKCSH, RAC1, TNF* |
| Phorbol myristate acetate | Chemical drug | 1.35 × 10^-7^ | *ADM, APP, BDNF, CDC42, CDK5R1, CPE, CTNNB1, CTSB, FGF2, FOXP1, FST, FZD1, GAP43, GATA3, GJA1, HAS2, ID2, IL5, IL6, KIT, KRT71, LYVE1, MAPK9, NME2, PLD1, PPARG, PRKACA, PRKCA, PRKCB, RBPJ, SNAP25, STAT5B, TFDP1, TNF, TP53* |
| *SMAD3* | Transcription regulator | 1.58 × 10^-7^ | *AXIN2, CTNNB1, FST, GATA3, GLI2, HAS2, HEY1, ID2, IL5, IL6, INPP5D, RAC1, S1PR1, TAGLN, TNF* |
| *IL1* | Group | 1.70 × 10^-7^ | *APP, BDNF, CTSB, DCN, FST, GJA1, IGF1R, IL5, IL6, KIT, KITLG, MAP2K6, PLA2G2A, PLD1, PPARG, TGM2, TNF, TP53* |
| *PRNP* | Other | 1.74 × 10^-7^ | *APP, FST, GJA1, ID2, IGF1R, IL6, PLPP3, SNAP25, TP53* |
| *TP63* | Transcription regulator | 1.98 × 10^-7^ | *ADM, APC, CDC42, CITED2, COL4A1, CTNNB1, DNM2, FST, IL6, MAFF, PIK3CA, PIK3R3, RAC2, SUFU, TAGLN, TP53, UPK1A, UPK1B, UPK2* |
| *DCN* | Other | 2.05 × 10^-7^ | *FGF2, FST, IGF1R, IL6, KIT, S1PR1, TNF, TP53* |
| *NR4A1* | Ligand-dependent nuclear receptor | 2.28 × 10^-7^ | *ADIPOQ, ADM, APOD, AXIN2, CAV3, CTNNB1, EHF, IL5, IL6, KCNIP3, KITLG, PPARG, TNF* |
| *MAPKAPK2* | Kinase | 2.39 × 10^-7^ | *CCL11, CITED2, IL5, IL6, LMO4, STAT5A, TNF, TP53* |
| *EGF* | Growth factor | 2.42 × 10^-7^ | *CDC42EP1, DCN, DVL2, FGF2, FST, GJA1, GSK3B, HAS2, ID2, IGF1R, IL6, KRT5, MAPK11, MAPK3, PLPP3, PPARG, RAC1, SNAP25, TFAP2A, TFDP1, TGM2, TP53* |
| Paroxetine | Chemical drug | 2.60 × 10^-7^ | *ADM, APP, BDNF, ID2, TNF, TP53* |
| Bucladesine | Chemical toxicant | 2.83 × 10^-7^ | *BDNF, CDK5R1, DNM2, FCER1A, FST, GAP43, HHIP, IGF1R, IL5, IL6, KIT, KITLG, PTCH1, S100B, SLC9A3R1, SNAP25, TNF* |
| *HMGA1* | Transcription regulator | 2.97 × 10^-7^ | *COL4A1, CPE, CSK, CTSH, GSN, HAND1, IL6, KIT, KITLG, PPARG, PSEN2, TAGLN* |
| Methotrexate | Chemical drug | 3.22 × 10^-7^ | *ACADVL, CCL11, COL4A1, GAP43, GSN, IL6, ITIH4, KRT71, MAP2K6, PFN2, PRKCB, RAC1, STAT5B, TNF, TP53* |
| *GLI2* | Transcription regulator | 3.28 × 10^-7^ | *BMP4, CCL11, GATA3, GLI2, HHIP, IL6, KRT17, MEF2C, PTCH1, TNF* |
| *NFKBIA* | Transcription regulator | 4.09 × 10^-7^ | *BCCIP, CCL11, CDH3, CSK, CTNNB1, CTSB, DCN, ENPP2, FGF2, HEY1, IL5, IL6, PIK3R1, RAC1, SEMA3C, TFDP1, TGFB1I1, TNF, TOLLIP, TP53* |
| *WNT1* | Cytokine | 4.50 × 10^-7^ | *AXIN2, BMP4, CTNNB1, ENPP2, IGF1R, KIT, KRT5, PPARG, SEMA3C, STAT5A, TP53* |
| *ERBB2* | Kinase | 4.69 × 10^-7^ | *CCL11, CDC42, CDC42EP2, CDH3, COL4A1, CTNNB1, CTSB, E2F8, EHF, GATA3, GJA1, GSK3B, HAS2, HEYL, ID2, IGF1R, IL6, IRF6, KIT, MYO10, NFIB, PFN2, PPARG, STMN3, TAGLN, TNF, TP53* |
| *SPHK2* | Kinase | 4.70 × 10^-7^ | *APP, FYN, IL6, TNF, TP53* |
| *HDAC1* | Transcription regulator | 5.13 × 10^-7^ | *ADIPOQ, AXIN2, BDNF, GNAS, GSK3B, IL5, IL6, MEF2C, PPARG, SLC35D1, TAGLN, TBX6, TGM2, TNF, TP53* |
| *HTT* | Transcription regulator | 5.58 × 10^-7^ | *APP, AQP1, ATP2C1, BDNF, CITED2, COL4A1, CTNNB1, DCN, ENPP2, ERCC3, GAP43, GSN, KCNJ2, NCS1, NFIB, PFN2, PIK3CA, PPARG, PRKCB, SNAP25, STMN1, TAGLN, TBX6, TGM2, TP53, UCHL1* |
| *PSMD10* | Transcription regulator | 5.76 × 10^-7^ | *GNAS, IL6, PIK3CA, PIK3R1, PLD1, TP53* |
| Cyanoco-balamin | Chemical - endogenous mammalian | 5.76 × 10^-7^ | *APC, APP, FZD1, FZD6, PTCH1, TNF* |
| Cycloheximide | Chemical reagent | 5.98 × 10^-7^ | *ADM, APP, BDNF, CCL11, CITED2, CTNNB1, FGF2, FST, GAP43, GJA1, GNAS, IL5, IL6, PLA2G2A, PPARG, PSEN2, TNF, TP53* |
| *ENPP2* | Enzyme | 6.99 × 10^-7^ | *ADIPOQ, ENPP2, IL6, PPARG, TNF* |
| Azoxymethane | Chemical toxicant | 8.09 × 10^-7^ | *CTNNB1, GSK3B, IGF1R, IL6, PRKCA, PRKCB, TP53* |
| Sphingosine-1-phosphate | Chemical - endogenous mammalian | 8.48 × 10^-7^ | *ENPP2, FGF2, GAP43, HAS2, IL6, PLA2G2A, S1PR1, TAGLN, TNF* |
| *FOS* | Transcription regulator | 8.81 × 10^-7^ | *AGPS, ARF1, ASAP1, ATP2C1, BDNF, CTSB, CTSH, DOCK1, FGF2, FRZB, FZD3, FZD6, GJA1, GSK3B, HAS2, IL6, KITLG, PLD1, STMN1, TNF, TP53, WNT11* |
| Morphine | Chemical drug | 9.63 × 10^-7^ | *ADM, AIF1, BDNF, CALB1, GATA3, GNAS, IL6, MAPK3, TNF, TP53, TRAF6* |
| *BMP4* | Growth factor | 1.03 × 10^-6^ | *BMP4, CCL11, GJA1, HEY1, ID2, IL6, KIT, PTCH1, TAGLN, TGM2, TP53* |
| Tamoxifen | Chemical drug | 1.11 × 10^-6^ | *ADM, AQP1, BDNF, BMP4, CPE, FLOT1, GAP43, HAS2, IL6, MAFF, SCN1B, SLC9A3R1, TAGLN, TNF, TP53* |
| *RET* | Kinase | 1.14 × 10^-6^ | *CALB1, CTSB, DCN, DPYSL2, GJA1, IL5, IL6, TNF, TP53, WNT11* |
| *GLI1* | Transcription regulator | 1.25 × 10^-6^ | *ACADVL, GATA5, GLI2, HHIP, ID2, IL6, MAPK11, MEF2C, PTCH1, PTCH2, TMEM100, TP53, WNT2B* |
| *RB1* | Transcription regulator | 1.29 × 10^-6^ | *CDK5R1, CITED2, COL4A3BP, EDNRA, FGF2, FST, IGF1R, IL6, KIT, MAP2K7, MAPK3, MEF2C, PPARG, PSEN2, TFDP1, TNF, TNNC1, TP53* |
| *CSF3* | Cytokine | 1.30 × 10^-6^ | *ENPP2, GJA1, IL6, KIT, KITLG, NME2, PPARG, PRKCA, PRKCB, TFDP1, TNF, TP53* |
| Calcitriol | Chemical drug | 1.34 × 10^-6^ | *APP, CALB1, CDK5R1, COL4A1, EHF, GATA3, ID2, IGF1R, IL5, IL6, KCNH1, PADI1, PLCG1, PLD1, PPARG, PRKCA, PRKCB, STMN1, TMEM100, TNF, TP53, WNT11* |
| *EREG* | Growth factor | 1.41 × 10^-6^ | *CALB1, FGF2, HAS2, IL6, TNF* |
| Transforming growth factors | Group | 1.49 × 10^-6^ | *CCL11, CTNNB1, DCN, FGF2, FST, GATA3, GLI2, HEY1, IL5, IL6, INPP5D, PLPP3, TAGLN, TNF* |
| *NOTCH1* | Transcription regulator | 1.65 × 10^-6^ | *CTNNB1, FGF2, GATA3, GLI2, HEY1, HEYL, ID2, IGF1R, IL6, LYVE1, PPARG, TAGLN, TNF, TP53* |
| *BRCA1* | Transcription regulator | 1.66 × 10^-6^ | *CDH3, CTNNB1, ENPP2, ERCC3, IGF1R, KIT, KRT17, KRT5, STAT5A, STAT5B, TNF, TP53* |
| *INS1* | Other | 1.67 × 10^-6^ | *ADIPOQ, ARF1, BDNF, FGF2, GATA3, IGF1R, IL6, MAPK3, PIK3R1, PIK3R2, PPARG, PRKCA, PRKCB, SNAP25, STAT5A, TNF* |
| 1-methyl-4-phenyl-1,2,3,6-tetrahydro-pyridine | Chemical toxicant | 1.70 × 10^-6^ | *APP, BDNF, CALB1, FGF2, IL6, MAP2K4, TNF, TP53* |
| *RELA* | Transcription regulator | 1.99 × 10^-6^ | *APP, CCL11, CITED2, COL4A3BP, CTSB, EHF, FGF2, GLI2, HAS2, IGF1R, IL6, KIT, PLD1, PPARG, STAT5A, TGM2, TNF, TP53* |
| *FGF2* | Growth factor | 2.07 × 10^-6^ | *BDNF, BMP4, CALB1, CTNNB1, DCN, ENPP2, FGF2, GAP43, GJA1, HAS2, IGF1R, IL6, LYVE1, PPARG, TAGLN, TNF, TP53* |
| *CG*  *(CGA, CGB1/CGB2, CGB3, CGB7)* | Complex | 2.15 × 10^-6^ | *ADM, BDNF, ENPP2, FST, FYN, FZD1, GHRL, GJA1, HAS2, IGF1R, IL6, MAPK3, PLPP3, PPARG, SNAP25, TNF, TP53* |
| *PI3K* (complex)  *(ATM,*  *FGFR1,*  *FGFR2,*  *FGFR3,*  *FGFR4,*  *FRS2,*  *GAB1,*  *GRB2,*  *IRS1, IRS2,*  *KL, KLB,*  *P110, PI3K,*  *PIK3C2A,*  *PIK3C2B,*  *PIK3C2G,*  *PIK3C3,*  *PIK3CA,*  *PIK3CB,*  *PIK3CD,*  *PIK3CG,*  *PIK3R1,*  *PIK3R2,*  *PIK3R3,*  *PIK3R4,*  *PIK3R5,*  *PIK3R6,*  *PTPN11,*  *TLR9)* | Complex | 2.39 × 10^-6^ | *CDK5R1, GAP43, GATA3, GJA1, GLI2, IGF1R, IL6, MAP2K4, MEF2C, PLA2G5, STMN1, TAGLN, TGM2, TNF, TP53* |
| Mifepristone | Chemical drug | 2.39 × 10^-6^ | *ADM, AQP1, CCL11, FGF2, FST, GJA1, HAS2, IL6, KIT, NME2, NPY1R, SNAP25, STAT5A, TNF, TP53* |
| *NR3C1* | Ligand-dependent nuclear receptor | 2.45 × 10^-6^ | *ADM, APP, AQP1, ARPC2, BDNF, EFNA1, GAP43, GATA3, IGF1R, IL6, KRT17, KRT5, LMO4, MAPK3, MAPK9, PIK3R1, PIK3R3, PPARG, PRKCB, RAF1, SEMA3C, STAT5A, TNF, TP53* |
| *MAPK8IP1* | Other | 2.60 × 10^-6^ | *APP, BDNF, IL6, MAP2K7, TNF* |
| *GH1* | Growth factor | 2.64 × 10^-6^ | *APC, BMP4, CALB1, CTNNB1, ERCC3, GHRL, ID2, IGF1R, IL6, PPARG, SMARCA4, STAT5A, TFAP2A* |
| *CDC42* | Enzyme | 3.10 × 10^-6^ | *CTSB, CTSH, FGF2, GSK3B, STMN1, TNF* |
| Benzo(a)-pyrene | Chemical toxicant | 3.57 × 10^-6^ | *CDK5R1, FST, FYN, FZD1, GNA11, HAS2, MAFF, MAP2K4, MAP2K6, MAP2K7, PRKACA, PRKCA, TP53* |
| *IGFBP3* | Other | 3.70 × 10^-6^ | *ADIPOQ, CCL11, IL5, LYVE1, SDC2, TNF* |
| *NOTCH2* | Transcription regulator | 3.70 × 10^-6^ | *HEY1, IL6, SDC2, TAGLN, TNF, TP53* |
| *VEGF*  *(VEGFA-VEGFD, PDF, PDGFC, PROKQ1)* | Group | 3.72 × 10^-6^ | *AXIN2, CALB1, CDC42, CTNNB1, EDA, EFNA1, EHF, EMP2, ENPP2, FGF2, FST, IL6, KITLG, LYVE1, MEF2C, PADI1, PLPP3, PRKCB, RAC1, TNF* |
| *ERK1/2* | Group | 3.80 × 10^-6^ | *ADIPOQ, BDNF, CAV3, CCL11, CTSB, FGF2, FZD1, GJA1, HAS2, IGF1R, IL6, KRT17, MAPK3, RAC1, TNF* |
| Aldosterone | Chemical - endogenous mammalian | 3.86 × 10^-6^ | *ADIPOQ, ADM, BDNF, HAS2, IGF1R, IL6, KCNMA1, KCNMB1, PPARG, TNF* |
| *Notch* | Group | 3.99 × 10^-6^ | *ADM, GATA3, HEY1, HEYL, ID2, IL6, TAGLN, TNF* |
| *TCR* | Complex | 4.12 × 10^-6^ | *ADM, AKT3, APOD, CTSB, FYN, GATA3, IL5, IL6, MAPK9, PIK3R1, PIK3R3, PLCG1, STAT5A, TNF, VAV1* |
| Rosiglitazone | Chemical drug | 4.36 × 10^-6^ | *ACADVL, ADIPOQ, ATL1, BDNF, CCL11, COL4A3BP, CTNNB1, EDNRA, GATA3, HAS2, IL6, PIK3R1, PLPP3, PPARG, SEMA3C, TNF, UPK1B, UPK2* |
| *CCL2* | Cytokine | 4.50 × 10^-6^ | *ADIPOQ, CCL11, CTSB, IL5, IL6, PPARG, TNF* |
| *GSK3B* | Kinase | 4.73 × 10^-6^ | *APP, CTNNB1, IFT27, IGF1R, IL6, RAC1, TCF7L2, TNF, TP53* |
| *SIRT6* | Enzyme | 5.09 × 10^-6^ | *AKT3, GSK3B, IGF1R, IL6, MAPK3, PLPP3, TNF* |
| *IL2* | Cytokine | 5.13 × 10^-6^ | *BCCIP, CCL11, CDK5R1, ENPP2, FGF2, FYN, GATA3, IL5, IL6, KIT, MAP2K6, PIK3R3, PLA2G10, PPARG, PTCH1, RAF1, SOS1, STAT5B, TNF, TP53, TRAF6* |
| *MAPK1* | Kinase | 5.20 × 10^-6^ | *BDNF, CAV3, CDK5R1, CTNNB1, CTSB, DSE, GATA3, IL5, IL6, KRT17, MAPK3, PLA2G5, PLD1, TDRD7, TNF, TP53* |
| Bleomycin | Chemical drug | 5.27 × 10^-6^ | *CTNNB1, FRZB, FZD1, FZD6, HEY1, IL5, IL6, KITLG, TNF, TNNC1, TP53* |
| *ADCY*  *(ADCY1-10)* | Group | 5.71 × 10^-6^ | *FGF2, GJA1, IL6, TNF, TP53* |
| *GATA4* | Transcription regulator | 5.93 × 10^-6^ | *FGF2, GATA3, GJA1, IL5, IRF6, KCNJ2, KRT17, MEF2C, PLA2G2A, TAGLN, TNNC1* |
| *SATB1* | Transcription regulator | 5.93 × 10^-6^ | *APC, GATA3, GATA5, IL5, KITLG, MEF2A, PRKCB, S1PR1, SS18L1, TBX6, TNF* |
| Deferoxamine | Chemical drug | 5.98 × 10^-6^ | *ADM, APP, BDNF, CITED2, CTNNB1, IL6, KIT, PLA2G12B, PLA2G3, PPARG, PRKCA, TP53* |
| Forskolin | Chemical toxicant | 6.43 × 10^-6^ | *ADIPOQ, APP, ATP6V0D1, BDNF, CCL11, FCER1A, FST, GAP43, GJA1, GNAS, HAS2, HHIP, ID2, IGF1R, IL5, IL6, KITLG, MAPK3, NPY1R, PRKACA, SNAP25, TNF, TP53* |
| *Mek* | Group | 6.62 × 10^-6^ | *CDK5R1, CTNNB1, GAP43, HAND1, HAS2, ID2, IL6, MAFF, PLPP3, SEMA3C, TNF, TP53* |
| *IgG*  *(IGG1, IGG3, IGHG1-4)* | Complex | 6.65 × 10^-6^ | *ADM, APP, ATP6V0D1, CCL11, FST, GNAS, IL5, IL6, S1PR1, ST14, TNF* |
| *BMP2* | Growth factor | 6.97 × 10^-6^ | *BMP4, CALB1, FGF2, FST, FZD1, HAS2, HEY1, ID2, MEF2C, PPARG, TAGLN, TGM2* |
| Androgen | Chemical drug | 7.04 × 10^-6^ | *APOD, AXIN2, BDNF, BMP4, CTNNB1, CTSB, FGF2, GJA1, GSN, PIK3R1, PRKCB* |
| *BBS12* | Other | 7.07 × 10^-6^ | *ADIPOQ, IL6, TNF* |
| *CTDNEP1* | Phosphatase | 7.07 × 10^-6^ | *AXIN2, CTNNB1, DVL2* |
| *EZH1* | Enzyme | 7.07 × 10^-6^ | *IL6, TNF, TOLLIP* |
| *CYP11B2* | Enzyme | 7.07 × 10^-6^ | *IGF1R, KCNMA1, KCNMB1* |
| *PPP1CC* | Phosphatase | 7.07 × 10^-6^ | *BDNF, IL6, TNF* |
| Cromolyn | Chemical drug | 7.07 × 10^-6^ | *APP, IL6, TNF* |
| Maslinic acid | Chemical - endogenous non-mammalian | 7.08 × 10^-6^ | *DOCK1, GSK3B, MAP2K6, PIK3R1, PRKACA, PRKCB, SOS1, TP53* |
| *PRKAA2* | Kinase | 7.54 × 10^-6^ | *GATA3, IL6, ITIH5, LMO4, NFIB, PPARG, RAF1, TGM2, TP53* |
| Resiquimod | Chemical drug | 7.89 × 10^-6^ | *AIF1, CCL11, FGF2, GATA3, HEY1, IL5, IL6, KCNMA1, STMN1, TNF, TRAF6* |
| *NFATC3* | Transcription regulator | 8.13 × 10^-6^ | *AIF1, GAP43, GJA1, IL5, IL6, PPARG, TNF* |
| Corticosteroid | Chemical drug | 8.32 × 10^-6^ | *AQP1, GAP43, IL5, IL6, PPARG, TNF* |
| Valsartan | Chemical drug | 8.32 × 10^-6^ | *ADIPOQ, APP, AQP1, FGF2, GJA1, KIT* |
| Cyclosporin A | Biologic drug | 8.52 × 10^-6^ | *APOD, APP, BDNF, CALB1, CCL11, EDNRA, FST, FYN, GJA1, IL5, IL6, LYVE1, MYH10, PRKCA, STAT5A, TAGLN, TNF, TP53* |
| Hydrogen peroxide | Chemical - endogenous mammalian | 8.79 × 10^-6^ | *ADIPOQ, APP, CTNNB1, CTSB, ENPP2, FGF2, IGF1R, IL6, KIT, KITLG, LGR4, PPARG, PRKCSH, RAC1, STMN1, TAGLN, TGM2, TNF, TP53* |
| Calpain | Complex | 9.04 × 10^-6^ | *APP, CTNNB1, GJA1, IL6, TP53* |
| *NAMPT* | Cytokine | 9.04 × 10^-6^ | *BDNF, IL6, NPY1R, TNF, TP53* |
| *UCN* | Other | 9.04 × 10^-6^ | *GNAS, IL6, MAPK3, PLA2G6, TNF* |
| *HSPD1* | Enzyme | 9.04 × 10^-6^ | *GATA3, IGF1R, IL5, IL6, TNF* |
| *TAZ* | Enzyme | 9.09 × 10^-6^ | *BDNF, ENPP2, KIT, KRT5, S1PR1, SEMA3C, TAGLN* |
| *P38 MAPK* | Group | 9.55 × 10^-6^ | *ADIPOQ, BDNF, CAV3, CCL11, FGF2, FST, GJA1, IL5, IL6, IRF6, MEF2C, PPARG, SDC2, TNF, TNNC1, TP53* |
| *MAPK10* | Kinase | 9.83 × 10^-6^ | *APP, IL6, TNF, WNT7A* |
| *GATA6* | Transcription regulator | 9.84 × 10^-6^ | *ATP6V0D1, COL4A1, FGF2, IRF6, KRT17, LYVE1, MEF2C, SEMA3C, TAGLN, TNNC1, WNT2* |
| *Y27632* | Chemical drug | 1.01 × 10^-5^ | *BMP4, DPYSL2, HAS2, IL6, PPARG, TAGLN, TP53* |
| Rimonabant | Chemical drug | 1.12 × 10^-5^ | *ADIPOQ, FGF2, IL6, TNF, TP53* |
| Alpha-tocopherol | Chemical drug | 1.12 × 10^-5^ | *ADIPOQ, IL6, PPARG, TNF, TP53* |
| *GATA3* | Transcription regulator | 1.20 × 10^-5^ | *ADM, GATA3, IL5, IL6, IRF6, KIT, KRT17, MYO10, PPARG, STAT5A, TNF, WNT11* |
| *EHF* | Transcription regulator | 1.20 × 10^-5^ | *BMP4, CDH3, EHF, FCER1A, IL6, KIT, RBPJ, STAT5B* |
| *IL15* | Cytokine | 1.25 × 10^-5^ | *CCL11, FYN, GATA3, GNAS, IL5, IL6, KIT, MAPK3, PLD1, PRKACA, RAC2, RBPJ, S1PR1, SOS1, TNF, VDAC3* |
| *PSEN2* | Peptidase | 1.30 × 10^-5^ | *APP, CTNNB1, ENPP2, GJA1, HAND1, IGF1R, PSEN2, TP53* |
| Diethylstil-bestrol | Chemical drug | 1.38 × 10^-5^ | *ADIPOQ, CITED2, CPE, FRZB, GAP43, IGF1R, ITIH4, KIF4A, LMO4, MAPK3, STAT5A, WNT7A* |
| *NFATC2* | Transcription regulator | 1.40 × 10^-5^ | *CITED2, ENPP2, GATA3, IL5, LMO4, PLA2G2A, PLD1, PPARG, STAT5A, TNF* |
| *NFKB1* | Transcription regulator | 1.52 × 10^-5^ | *APP, CCL11, CTSB, EHF, ENPP2, GATA3, GJA1, HAS2, IL5, IL6, PRKACA, TNF, TP53* |
| *FOXM1* | Transcription regulator | 1.54 × 10^-5^ | *AXIN2, CCL11, CTNNB1, FZD1, IL6, PTCH1, PTCH2, STMN1, TP53* |
| *RAF*  *(RAF1, ARAF, BRAF)* | Group | 1.54 × 10^-5^ | *HAS2, ID2, IGF1R, MAFF, PLPP3, SEMA3C, TAGLN* |
| *KRAS* | Enzyme | 1.59 × 10^-5^ | *AXIN2, BMP4, CDH3, COL4A1, CTNNB1, EFNA1, GJA1, GLI2, GSN, IGF1R, IL6, MAPK12, NME2, PLD1, PPARG, STAT5A, TNF, TP53* |
| Trichostatin A | Chemical drug | 1.62 × 10^-5^ | *APP, ARFIP2, BMP4, ENPP2, FRZB, GATA3, GSN, ID2, IL5, IL6, KIT, PAFAH1B1, PPARG, SDC2, SEMA3C, SMARCA4, STMN1, TAGLN, TGM2, TNF, TP53* |
| Dimethyl-nitrosamine | Chemical toxicant | 1.68 × 10^-5^ | *COL4A1, IL6, MAPK3, RAB13, TGM2, TNF* |
| *F3* | Transmem-brane receptor | 1.70 × 10^-5^ | *CDC42, GJA1, IL6, PLPP3, RAC1, TGM2, TNF* |
| *RSPO3* | Kinase | 1.75 × 10^-5^ | *AXIN2, IL6, TNF* |
| *SGMS1* | Enzyme | 1.75 × 10^-5^ | *APP, IL6, TNF* |
| *IgG2b* | Complex | 1.75 × 10^-5^ | *APP, IL6, TNF* |
| Ca^2+^ | Chemical - endogenous mammalian | 1.80 × 10^-5^ | *APP, BDNF, CDC42, FGF2, IGF1R, IL6, MEF2A, PADI1, PLA2G2F, PLCG1, PPARG, TAGLN, TNF* |
| *TGFBR2* | Kinase | 1.84 × 10^-5^ | *BDNF, CTNNB1, EXT2, FST, GJA1, GLI2, IL5, IL6, NFIB, TAGLN, TNF* |
| *MAPK9* | Kinase | 1.87 × 10^-5^ | *AQP1, BMP4, IL5, IL6, NME2, PPARG, PTCH1, RAC1, TNF, TP53* |
| *APC* | Enzyme | 1.88 × 10^-5^ | *AXIN2, CTNNB1, GHRL, ID2, IL6, PLA2G10, PRKCA, PRKCB, TNF* |
| *SMAD4* | Transcription regulator | 1.89 × 10^-5^ | *BMP4, CDH3, CITED2, CTNNB1, FST, GJA1, HAS2, ID2, INPP5D, RAC1, TBX6, TNF* |
| *Histone H3* | Group | 1.91 × 10^-5^ | *ADIPOQ, BDNF, BMP4, CTNNB1, EFNA1, GATA3, HAND1, ID2, IGF1R, IL5, IL6, PPARG, PTCD2, PTCH1, TAGLN, TBX6, WNT8B* |
| Vincristine | Chemical drug | 1.91 × 10^-5^ | *GAP43, MAP2K4, MAP2K7, MAPK9, PRKCA, TP53* |
| Gamma-tocotrienol | Chemical drug | 1.95 × 10^-5^ | *CTNNB1, IL6, TNF, TRAF6* |
| *TCF3* | Transcription regulator | 1.98 × 10^-5^ | *ADIPOQ, AXIN2, DOCK1, GATA3, HAND1, ID2, KIF4A, KIT, LMO4, MEF2C, PLCG2, TNF* |
| *LIPE* | Enzyme | 2.01 × 10^-5^ | *ADIPOQ, AQP1, CITED2, GJA1, ID2, KITLG, MEF2C, PPARG, TNF* |
| *JAG1* | Growth factor | 2.01 × 10^-5^ | *GLI2, HEY1, HEYL, TAGLN, TP53* |
| Apomorphine | Chemical drug | 2.01 × 10^-5^ | *APC, BDNF, FGF2, GJA1, IGF1R* |
| *GSK3*  *(GSK3A, GSK3B, H2BFM)* | Group | 2.07 × 10^-5^ | *CTNNB1, IGF1R, IL6, PPARG, TBX6, TNF, TP53* |
| *BMP7* | Growth factor | 2.14 × 10^-5^ | *ADIPOQ, BMP4, GJA1, ID2, IGF1R, IL6, MAPK3, PIK3R1, PPARG* |
| *IL25* | Cytokine | 2.17 × 10^-5^ | *CCL11, FGF2, GATA3, IL5, IL6, TNF* |
| *AKT*  *(AKT1-3)* | Group | 2.20 × 10^-5^ | *ADIPOQ, ADM, CAV3, CTNNB1, GLI2, GSK3B, IGF1R, IL5, IL6, PPARG, RDH10, TNF, TP53* |
| *MIR-199a-5p* (and other miRNAs w/seed CCAGUGU*)* | Mature microRNA | 2.28 × 10^-5^ | *COL4A1, DCN, KRT17, LIN7C, TAGLN, TGFB1I1, WNT7A* |
| *KITLG* | Growth factor | 2.36 × 10^-5^ | *AIF1, GJA1, ID2, IGF1R, IL5, IL6, KIT, MAPK11, MEF2C, PRKCA, PRKCB, TNF* |
| Cigarette smoke | Chemical toxicant | 2.36 × 10^-5^ | *ADM, CCL11, GATA3, IL5, IL6, KITLG, MAFF, MAP2K6, PPARG, PRKCA, TNF, TP53* |
| Cisplatin | Chemical drug | 2.45 × 10^-5^ | *ACADVL, AQP1, BMP4, CTNNB1, CTSB, DOCK1, FYN, GAP43, HEYL, ID2, IGF1R, IL6, INPP5D, LMO4, MAPK9, PIK3CA, PRKCA, RAC1, STMN1, TFAP2A, TNF, TP53* |
| *SELENOS* | Other | 2.63 × 10^-5^ | *ADIPOQ, IL6, PPARG, TNF* |
| *MAPK8IP3* | Other | 2.63 × 10^-5^ | *GSK3B, MAP2K4, MAP2K7, MAPK10* |
| *SPINT1* | Other | 2.63 × 10^-5^ | *CTNNB1, FZD3, FZD6, WNT11* |
| L-685,458 | Chemical - protease inhibitor | 2.63 × 10^-5^ | *APP, HEY1, TNF, TP53* |
| *LEP* | Growth factor | 2.64 × 10^-5^ | *ACADVL, ADIPOQ, APP, BDNF, COL4A1, GAP43, GHRL, GNAS, GSK3B, IFT27, IL6, NPY1R, PPARG, RAC1, SNAP25, TNF, TP53* |
| Enterotoxin B | Biologic drug | 2.74 × 10^-5^ | *BDNF, CCL11, IL5, IL6, PFN2, TNF, UCHL1* |
| *FGFR2* | Kinase | 2.76 × 10^-5^ | *BMP4, COL4A1, CPE, CTSH, FST, GJA1, HHIP, ID2, IL6* |
| *MAPK7* | Kinase | 2.77 × 10^-5^ | *BDNF, IL6, MEF2A, MEF2C, TNF, TP53* |
| *GFI1* | Transcription regulator | 2.85 × 10^-5^ | *GATA3, GJA1, ID2, IL6, MAPK3, RAF1, TNF, TOLLIP* |
| *ERG* | Transcription regulator | 2.85 × 10^-5^ | *AXIN2, CTNNB1, DOCK1, EXT1, FYN, MYO10, PLPP3, TGFB1I1, WNT11, WNT2, WNT2B* |
| Lactacystin | Chemical - protease inhibitor | 2.85 × 10^-5^ | *APP, CAV3, GATA3, GJA1, IL6, NCS1, PRKCA, RAC1, STMN1, TNF, TP53* |
| *IgE*  *(IGH)* | Complex | 3.11 × 10^-5^ | *CCL11, FCER1A, IL5, IL6, PPARG, TNF* |
| *MIR-15* | MicroRNA | 3.11 × 10^-5^ | *APP, CDC42, FGF2, MAPK3, RAF1, TP53* |
| *EZH2* | Transcription regulator | 3.12 × 10^-5^ | *AXIN2, BMP4, COL4A1, CTNNB1, EFNA1, FRZB, FZD1, GATA3, GATA5, IL5, IL6, PPARG, TNF, TP53* |
| *FSH* | Complex | 3.16 × 10^-5^ | *ADM, AXIN2, BDNF, CPE, EFNA1, FGF2, FST, GNAS, IGF1R, IL6, KITLG, NPY1R, PLA2G10, SMARCA4, SNAP25, TP53* |
| Genistein | Chemical drug | 3.16 × 10^-5^ | *ADM, ATP6V0D1, BDNF, CITED2, CTSB, CTSH, EXT1, ID2, IGF1R, IL6, KCNJ2, NME7, PIK3R1, PPARG, TNF, TP53* |
| *CDH1* | Other | 3.28 × 10^-5^ | *BMP4, CDC42, CDH3, CTNNB1, IL5, PIK3R1, TNF* |
| *CYP19A1* | Enzyme | 3.28 × 10^-5^ | *ACADVL, BDNF, GSK3B, IL6, PPARG, TNF, TP53* |
| *JUN* | Transcription regulator | 3.33 × 10^-5^ | *AKT3, APP, BDNF, CDK5R1, FGF2, FRZB, GAP43, GJA1, GSK3B, ID2, IGF1R, IL6, MAPK3, STMN1, TNF, TP53, WNT16* |
| *NFATC4* | Transcription regulator | 3.37 × 10^-5^ | *ADIPOQ, APP, BDNF, PPARG, TNF* |
| *CYBB* | Enzyme | 3.37 × 10^-5^ | *IL5, IL6, KIT, PPARG, TNF* |
| Methyl-prednisolone | Chemical drug | 3.40 × 10^-5^ | *BDNF, CDH3, CITED2, CTSH, EDA, EFNA1, ENPP2, IL6, LGR4, LYVE1, MAPK9, NFIB, PRKACA, PTER, RAB13, RAF1, SULT1B1, TNF, TP53* |
| *ERK*  *(MAPK1,3,4,7,12,15)* | Group | 3.46 × 10^-5^ | *ARPC2, CDK5R1, CTNNB1, DPYSL2, FGF2, FST, HEY1, IL6, MAFF, TGM2, TNF, TP53* |
| *PHLPP2* | Enzyme | 3.47 × 10^-5^ | *PRKCA, PRKCB, TNF* |
| *DUSP10* | Phosphatase | 3.47 × 10^-5^ | *IL6, TNF, TP53* |
| *CDH13* | Other | 3.47 × 10^-5^ | *ADIPOQ, AKT3, CTNNB1* |
| Propyl gallate | Chemical toxicant | 3.47 × 10^-5^ | *PLA2G2A, TNF, TP53* |
| Scopoletin | Chemical - endogenous non-mammalian | 3.47 × 10^-5^ | *FGF2, IL5, IL6* |
| *DSP* | Other | 3.48 × 10^-5^ | *ADIPOQ, AXIN2, CTNNB1, PPARG* |
| Cyclopiazonic acid | Chemical - endogenous non-mammalian | 3.48 × 10^-5^ | *IL5, IL6, S100B, TNF* |
| *EPHB4* | Kinase | 3.49 × 10^-5^ | *BMP4, GATA3, KIT, KITLG, STAT5B, WNT11* |
| Salicylic acid | Chemical drug | 3.49 × 10^-5^ | *BDNF, CAV3, IL6, KITLG, TNF, TP53* |
| Estrogen receptor  *(ESR1, ESR2)* | Group | 3.76 × 10^-5^ | *ADIPOQ, AKT3, BDNF, CALB1, CDH3, COL4A1, F11R, GATA3, IL6, MAPK12, SLC9A3R1, TP53* |
| *POU5F1* | Transcription regulator | 3.84 × 10^-5^ | *CTNNB1, EXT2, FRZB, HAND1, IGF1R, IL6, IRF6, KRT17, MEF2A, MEF2C, TDRD7, TNF, TP53, WNT8B* |
| *IGF1* | Growth factor | 3.90 × 10^-5^ | *ADIPOQ, ADM, APP, BDNF, BMP4, CTNNB1, GAP43, ID2, IGF1R, IL6, MAPK3, PLA2G2A, PPARG, S100B, TNF, TP53* |
| Chloroquine | Chemical drug | 3.91 × 10^-5^ | *APP, GJA1, IL6, S1PR1, TNF, TRAF6* |
| *CASP1* | Peptidase | 3.96 × 10^-5^ | *CCL11, IL5, IL6, PPARG, TNF* |
| *SFRP1* | Transmembrane receptor | 3.96 × 10^-5^ | *APP, CTNNB1, GATA3, SDC2, TP53* |
| Immuno-globulin | Complex | 4.08 × 10^-5^ | *ADM, AIF1, APP, DPYSL2, EXT1, GATA3, IL5, IL6, MAFF, MEF2C, TCF7L2, TNF* |
| *NFE2L2* | Transcription regulator | 4.14 × 10^-5^ | *ARF1, BDNF, CALB1, GNA11, IL5, IL6, KCNB2, MAFF, MAPK10, MEF2C, PAFAH1B1, PFN2, PPARG, PRKCB, TNF, TP53* |
| *PDGF, BB*  *(PDGF, PDGBB)* | Complex | 4.25 × 10^-5^ | *ADM, BMP4, DCN, FRZB, FZD1, GJA1, IGF1R, IL6, PFN2, PLCG2, PPARG, TAGLN, TGM2, TP53* |
| Fingolimod | Chemical drug | 4.36 × 10^-5^ | *ADIPOQ, BDNF, CTNNB1, IL6, S1PR1, TNF* |
| *LGR4* | Transmembrane receptor | 4.51 × 10^-5^ | *AXIN2, IL6, TNF, WNT7A* |
| *HNRNPA1* | Enzyme | 4.51 × 10^-5^ | *ANXA7, APP, IL6, TNF* |
| Thalidomide | Chemical drug | 4.62 × 10^-5^ | *APP, FGF2, IL5, IL6, TNF* |
| Andro-grapholide | Chemical drug | 4.62 × 10^-5^ | *CCL11, GATA3, IL5, IL6, TNF* |
| *Salmonella enterica* serotype *abortus equi* lipopoly-saccharide | Chemical toxicant | 4.63 × 10^-5^ | *ATL1, HEY1, ID2, IL6, KCNJ2, MAFF, NME7, PLD1, RAPGEF2, TNF* |
| H89 | Chemical - kinase inhibitor | 4.72 × 10^-5^ | *ADIPOQ, APP, FGF2, GJA1, IL5, IL6, PLD1, SNAP25, TNF* |
| *SP1* | Transcription regulator | 5.05 × 10^-5^ | *BDNF, BMP4, CITED2, EDA, FGF2, GJA1, GNAS, HAS2, IGF1R, KCNQ3, KIT, PADI1, PRKCA, PRKCB, SNAP25, TAGLN, TNF, TNNC1, TP53* |
| *CHUK* | Kinase | 5.14 × 10^-5^ | *CCL11, CTSB, DCN, ENPP2, HEY1, IL5, IL6, SEMA3C, SNX10, TNF, TP53* |
| Histone deacetylase inhibitor | Chemical drug | 5.36 × 10^-5^ | *FGF2, GSN, RAF1, SLC9A3R1, TP53* |
| Green tea polyphenol | Chemical drug | 5.36 × 10^-5^ | *ADIPOQ, IL6, PIK3R1, PPARG, TP53* |
| *SHH* | Peptidase | 5.36 × 10^-5^ | *BMP4, GLI2, HEY1, HHIP, IL6, MEF2C, PTCH1, PTCH2, STMN3, TFDP1* |
| Telmisartan | Chemical drug | 5.40 × 10^-5^ | *ADIPOQ, BDNF, IL6, PLA2G5, PPARG, TNF* |
| Resveratrol | Chemical drug | 5.43 × 10^-5^ | *ADIPOQ, AIF1, APP, BDNF, CAV3, CTNNB1, HEY1, IL6, PPARG, PSEN2, PTCH1, TNF, TP53* |
| *ITGA6* | Trans-membrane receptor | 5.74 × 10^-5^ | *CTNNB1, DVL2, ENPP2, TP53* |
| Sertraline | Chemical drug | 5.74 × 10^-5^ | *BDNF, IL6, TNF, TP53* |
| Thiazo-lidinedione | Chemical drug | 5.74 × 10^-5^ | *ADIPOQ, CTNNB1, PIK3R1, TNF* |
| *GMNN* | Transcription regulator | 5.89 × 10^-5^ | *CTNNB1, EXT2, FGF2, FRZB, HAND1, TP53, WNT8B* |
| *DLG1* | Kinase | 6.02 × 10^-5^ | *APC, KCNJ2, TNF* |
| *SERPINC1* | Enzyme | 6.02 × 10^-5^ | *IL6, MAPK3, TNF* |
| *WNT2* | Cytokine | 6.02 × 10^-5^ | *CTNNB1, DVL2, GSK3B* |
| *MGLL* | Enzyme | 6.02 × 10^-5^ | *APP, IL6, TNF* |
| *ROR2* | Kinase | 6.02 × 10^-5^ | *AXIN2, CTNNB1, PPARG* |
| *PSENEN* | Peptidase | 6.02 × 10^-5^ | *APP, PSEN2, TP53* |
| Latrunculin A | Chemical toxicant | 6.02 × 10^-5^ | *HAS2, IL6, TNF* |
| Phorbol 12,13-dibutyrate | Chemical - endogenous non-mammalian | 6.19 × 10^-5^ | *APP, IGF1R, PRKCA, PRKCB, TNF* |
| *SYK* | Kinase | 6.38 × 10^-5^ | *CITED2, FOXP1, FST, GATA3, IL6, PIK3CA, TNF* |
| *MEF2C* | Transcription regulator | 6.48 × 10^-5^ | *BDNF, FRZB, GJA1, KCNJ2, MEF2A, MEF2C, PLA2G2A, TNNC1* |
| 8-bromo-camp | Chemical reagent | 6.50 × 10^-5^ | *ADM, APP, CDC42, DCN, FGF2, GJA1, IGF1R, IL6, KITLG, TNF* |
| *KLF4* | Transcription regulator | 6.52 × 10^-5^ | *CTNNB1, EXT2, FRZB, HAND1, HEY1, IL6, IRF6, KRT17, MEF2C, TAGLN, TP53, WNT8B* |
| *MAP2K4* | Kinase | 6.62 × 10^-5^ | *IL6, MAPK9, MEF2C, PPARG, TNF, TP53* |
| *TAC1* | Other | 6.89 × 10^-5^ | *FCER1A, IL5, IL6, KITLG, PRKACA, PTCH1, TNF* |
| *ILK* | Kinase | 7.12 × 10^-5^ | *CTNNB1, GSK3B, IL6, MAPK3, TNF* |
| Cep-1347 | Chemical drug | 7.20 × 10^-5^ | *BDNF, IL6, MAP2K4, TNF* |
| Alpha-tocopherol succinate | Chemical drug | 7.20 × 10^-5^ | *FGF2, MAPK3, TNF, TP53* |
| *CYR61* | Other | 7.44 × 10^-5^ | *CTNNB1, DVL2, IL6, TNF, TOLLIP, TP53, WNT7A* |
| *PRKCA* | Kinase | 7.83 × 10^-5^ | *APP, ARPC2, CTSB, ID2, PLA2G6, PLD1, TNF, TP53* |
| *MYOCD* | Transcription regulator | 8.03 × 10^-5^ | *GJA1, HAND1, KCNMB1, MEF2C, PLA2G2A, TAGLN, TNNC1* |
| Lithium | Chemical drug | 8.03 × 10^-5^ | *APP, BDNF, CTNNB1, IL6, NCS1, TCF7L2, TP53* |
| Ibuprofen | Chemical drug | 8.15 × 10^-5^ | *APP, IL6, PPARG, RAC1, TNF* |
| Anandamide | Chemical - endogenous mammalian | 8.15 × 10^-5^ | *FGF2, IL6, PPARG, TNF, TP53* |
| Cyclic AMP | Chemical - endogenous mammalian | 8.47 × 10^-5^ | *APP, BDNF, CDC42, FGF2, GAP43, GATA3, GJA1, IL5, IL6, PTCH1, TNF, TP53* |
| *NGF* | Growth factor | 8.59 × 10^-5^ | *APP, BDNF, CDK5R1, GAP43, GSK3B, PPARG, RAC1, SNAP25, TFAP2A, TNF* |
| *ERBB4* | Kinase | 8.65 × 10^-5^ | *BDNF, CTNNB1, GAP43, GJA1, TP53, WNT2, WNT7A* |
| Ns-398 | Chemical reagent | 8.65 × 10^-5^ | *DNM2, FGF2, IL5, IL6, PPARG, TNF, TP53* |
| Haloperidol | Chemical drug | 8.65 × 10^-5^ | *BDNF, CTNNB1, DPP6, FGF2, GSK3B, PRKCA, SCN1B* |
| Benzyloxycarbonyl-Leu-Leu-Leu aldehyde | Chemical - protease inhibitor | 8.80 × 10^-5^ | *APP, CAV3, CTNNB1, CTSB, DCN, FGF2, HAS2, IL6, PPARG, RAC1, STMN1, TNF, TP53* |
| *LEF1* | Transcription regulator | 8.86 × 10^-5^ | *AXIN2, GATA3, HAS2, IL5, PTCH1, TP53* |
| *LRPAP1* | Other | 8.91 × 10^-5^ | *APP, IL6, MAPK3, TNF* |
| Dipyridamole | Chemical drug | 8.91 × 10^-5^ | *BDNF, IL6, TNF, TP53* |
| Plerixafor | Chemical drug | 8.91 × 10^-5^ | *IL5, IL6, KIT, KITLG* |
| Thyroid hormone | Chemical - endogenous mammalian | 8.99 × 10^-5^ | *BDNF, CPE, FZD3, FZD6, MAPK3, PLA2G2A, PLA2G5, ST14, STAT5B, TFDP1* |
| Rasagiline | Chemical drug | 9.29 × 10^-5^ | *APP, BDNF, PIK3R1, PRKCA, SOS1* |
| *IL33* | Cytokine | 9.31 × 10^-5^ | *APP, CCL11, GATA3, IL5, IL6, PLCG1, TNF* |
| 12(S)-hydroxyeicosatetraenoic acid | Chemical - endogenous non-mammalian | 9.54 × 10^-5^ | *IL6, PPARG, TNF* |
| 2',3'-dialdehyde ATP | Chemical reagent | 9.54 × 10^-5^ | *IL6, PLA2G5, TNF* |
| Ibudilast | Chemical drug | 9.54 × 10^-5^ | *IL6, NCS1, TNF* |
| *PFKFB3* | Kinase | 9.54 × 10^-5^ | *ADIPOQ, IL6, TNF* |
| *RGS19* | Other | 9.54 × 10^-5^ | *AXIN2, BMP4, MEF2C* |
| *IL18R1* | Transmembrane receptor | 9.54 × 10^-5^ | *IL5, IL6, TNF* |
| *DHH* | Peptidase | 9.54 × 10^-5^ | *GJA1, HHIP, PTCH1* |
| Magnesium sulfate | Chemical drug | 9.54 × 10^-5^ | *ADM, IL6, TNF* |
| *E. Coli* serotype 0127b8 lipopoly-saccharide | Chemical - endogenous non-mammalian | 9.83 × 10^-5^ | *ADIPOQ, AQP1, BDNF, EDNRA, FGF2, FZD1, GJA1, HAS2, IL6, TNF* |
| N-nitro-L-arginine methyl ester | Chemical drug | 1.00 × 10^-4^ | *ADIPOQ, ADM, CCL11, GJA1, IL6, TNF, TP53* |
| Minocycline | Chemical drug | 1.07 × 10^-4^ | *BDNF, GATA3, IL5, IL6, TNF, TP53* |
| Curcumin | Chemical drug | 1.07 × 10^-4^ | *ADIPOQ, APP, AQP1, BDNF, CTNNB1, FGF2, FYN, GNA11, GSK3B, IL6, MAPK3, PPARG, TNF, TP53* |
| *MYOD1* | Transcription regulator | 1.07 × 10^-4^ | *COL4A1, CTNNB1, ENPP2, FYN, ID2, MEF2A, MEF2C, PRKACA, SDC2, TNNC1* |
| *VEGFA* | Growth factor | 1.09 × 10^-4^ | *BMP4, CTSB, FGF2, GJA1, IL6, INPP5D, MEF2C, NME2, PLPP3, PRKCA, TNF, TP53* |
| *NTN1* | Other | 1.09 × 10^-4^ | *CCL11, CTSB, IL6, TNF* |
| Cannabidiol | Chemical drug | 1.09 × 10^-4^ | *BDNF, ID2, S100B, TNF* |
| *MYB* | Transcription regulator | 1.12 × 10^-4^ | *COL4A1, GATA3, IGF1R, IL5, KIT, KITLG, PDE2A, VAV1* |
| *TAF4* | Transcription regulator | 1.15 × 10^-4^ | *BDNF, CDH3, DCN, EHF, ID2, MAFF, TGM3* |
| Calphostin C | Chemical - kinase inhibitor | 1.17 × 10^-4^ | *IGF1R, IL6, KIT, PPARG, TNF, TP53* |
| *IL10RA* | Transmembrane receptor | 1.18 × 10^-4^ | *AKT3, BMP4, DCN, EHF, EMP2, IL6, KITLG, LMO4, PDE2A, S1PR1, TGM2, TGM3, TNF* |
| Panobinostat | Chemical drug | 1.19 × 10^-4^ | *CDC42, CITED2, KIT, RAC1, SLC9A3R1* |
| *SPHK1* | Kinase | 1.19 × 10^-4^ | *APP, IL6, MAP2K6, TNF, TP53* |
| *HGF* | Growth factor | 1.20 × 10^-4^ | *AIMP2, COL4A1, CTNNB1, EDA, EHF, EMP2, IL5, IL6, KITLG, KRT17, LYVE1, PLPP3, RAC1, SLC9A3R1, ST14, TNF, TP53* |
| 25-hydroxycholesterol | Chemical reagent | 1.27 × 10^-4^ | *APOD, FCER1A, IL6, MAFF, PIK3R3, PTCH1* |
| Actinomycin D | Chemical drug | 1.31 × 10^-4^ | *ADM, APP, BDNF, CCL11, CTNNB1, ID2, IGF1R, IL6, PPARG, TNF, TP53* |
| *MAPK13* | Kinase | 1.32 × 10^-4^ | *CITED2, IL6, TNF, TP53* |
| Sucrose | Chemical - endogenous mammalian | 1.32 × 10^-4^ | *APP, AQP1, IL6, SNAP25* |
| *THRA* | Ligand-dependent nuclear receptor | 1.33 × 10^-4^ | *APP, CTSH, FRZB, FZD9, IL6, PPARG, TNF, TP53* |
| *MIR-146* | MicroRNA | 1.35 × 10^-4^ | *IL6, KIT, TNF, TRAF6, WASF2* |
| *GHR* | Trans-membrane receptor | 1.35 × 10^-4^ | *ADIPOQ, APC, IL6, TNF, TP53* |
| *TCF7* | Transcription regulator | 1.35 × 10^-4^ | *AXIN2, CTNNB1, GATA3, PPARG, SDC2* |
| D-galactosamine | Chemical - endogenous mammalian | 1.35 × 10^-4^ | *CTSB, EDNRA, IL6, MAPK9, TNF* |
| *CCR2* | G-protein coupled receptor | 1.39 × 10^-4^ | *ADIPOQ, APP, CCL11, IL5, IL6, TNF* |
| Romidepsin | Biologic drug | 1.39 × 10^-4^ | *CDC42, FGF2, GSN, RAC1, RAF1, TP53* |
| *CXCL12* | Cytokine | 1.39 × 10^-4^ | *AIF1, CTNNB1, FYN, IL6, IRF6, KIT, MAPK12, TNF, TNNC1, TP53* |
| Tacrolimus | Chemical drug | 1.39 × 10^-4^ | *APOD, APP, CALB1, GAP43, IL5, MYH10, STAT5A, TMEM100, TNF, WNT7A* |
| *PRL* | Cytokine | 1.39 × 10^-4^ | *APP, CTSB, CTSH, DCN, ID2, IL6, KRT5, LYVE1, STAT5A, TDRD7, TNF, TP53* |
| *PTEN* | Phosphatase | 1.41 × 10^-4^ | *ADIPOQ, ADM, CITED2, CTNNB1, CTSB, IFT27, IGF1R, IL6, PIK3R1, PPARG, PRKCB, SMARCA4, STAT5A, STAT5B, TNF, TP53* |
| *APH1A* | Peptidase | 1.42 × 10^-4^ | *APP, PSEN2, TP53* |
| *UNC5B* | Transmembrane receptor | 1.42 × 10^-4^ | *CCL11, IL6, TNF* |
| *RALBP1* | Enzyme | 1.42 × 10^-4^ | *COL4A1, IL6, TNF* |
| *IGHG2b* | Other | 1.42 × 10^-4^ | *APP, IL6, TNF* |
| *CD48* | Other | 1.42 × 10^-4^ | *CCL11, IL5, TNF* |
| *SLC30A3* | Transporter | 1.42 × 10^-4^ | *APP, BDNF, SNAP25* |
| *BQ 123* | Chemical drug | 1.42 × 10^-4^ | *GJA1, IL6, TNF* |
| Daidzein | Chemical drug | 1.42 × 10^-4^ | *ADIPOQ, AIF1, BDNF, ID2, IL6, PIK3R1, TP53* |
| *CEBPB* | Transcription regulator | 1.43 × 10^-4^ | *ADIPOQ, BDNF, CDH3, CTNNB1, DCN, FZD1, GNAS, GSK3B, HAS2, IL5, IL6, PPARG, RAC2, TNF, TP53* |
| *ADNP* | Transcription regulator | 1.48 × 10^-4^ | *TNF, TP53* |
| *DHCR24* | Enzyme | 1.48 × 10^-4^ | *APP, TNF* |
| *MIR-504* | MicroRNA | 1.48 × 10^-4^ | *FOXP1, TP53* |
| *IL1RAPL1* | Trans-membrane receptor | 1.48 × 10^-4^ | *IL6, TNF* |
| *CASP10* | Peptidase | 1.48 × 10^-4^ | *IL6, TNF* |
| Polysaccharide ta-1 | Chemical reagent | 1.48 × 10^-4^ | *IL6, TNF* |
| *12(S)-HPETE* | Chemical - endogenous mammalian | 1.48 × 10^-4^ | *IL6, TNF* |
| Bis-pom-pmea | Chemical drug | 1.48 × 10^-4^ | *IL6, TNF* |
| *PML* | Transcription regulator | 1.49 × 10^-4^ | *ADIPOQ, CPE, ID2, MAFF, MAPK11, PRKCA, STMN1, TP53* |
| *PLA2G10* | Enzyme | 1.51 × 10^-4^ | *BMP4, GATA3, IL5, IL6, KRT71, TNF* |
| *CD3E* | Transmembrane receptor | 1.51 × 10^-4^ | *IL5, IL6, MAPK11, PIK3R1, STK4, TNF* |
| *GDNF* | Growth factor | 1.51 × 10^-4^ | *BDNF, CTSB, DPYSL2, KIT, PRKACA, TNF* |
| *SOX9* | Transcription regulator | 1.51 × 10^-4^ | *CTNNB1, KIT, MAPK3, PPARG, PRKCA, TCF7L2* |
| Galactosyl-ceramide-alpha | Chemical reagent | 1.51 × 10^-4^ | *CCL11, GATA3, IL5, IL6, TNF* |
| *GLI3* | Transcription regulator | 1.51 × 10^-4^ | *BMP4, GLI2, HHIP, PTCH1, WNT2B* |
| *AKT2* | Kinase | 1.51 × 10^-4^ | *CTNNB1, IGF1R, PPARG, RAC1, TNF* |
| 4-hydroxy-nonenal | Chemical toxicant | 1.51 × 10^-4^ | *APP, CTSB, IL6, SUFU, TNF* |
| Desipramine | Chemical drug | 1.58 × 10^-4^ | *BDNF, FGF2, IL6, TNF* |
| Calcifediol | Chemical - endogenous mammalian | 1.58 × 10^-4^ | *CCL11, IL6, TNF, TP53* |
| *REL* | Transcription regulator | 1.58 × 10^-4^ | *AGPS, APP, IL5, IL6, IRF6, PIK3CA, RDH10, STAT5B, TNF, TTC30B, TTLL1* |
| *CRP* | Other | 1.70 × 10^-4^ | *FCER1A, IL6, PRKCA, PRKCB, TNF* |
| *MGEA5* | Enzyme | 1.75 × 10^-4^ | *CALB1, CPE, CTNNB1, FZD10, FZD6, GSN, MYO10, PIK3R1, PLCG1, PLPP3, ST14, TFDP1, TP53, WNT11* |
| Indomethacin | Chemical drug | 1.77 × 10^-4^ | *APP, BDNF, CTNNB1, FGF2, HAS2, IL5, IL6, PPARG, PRKCA, TNF, TRAF6* |
| *PPP3CA* | Phosphatase | 1.79 × 10^-4^ | *APP, IGF1R, PLD1, PLPP3, S100B, TNF* |
| *IL18* | Cytokine | 1.79 × 10^-4^ | *ADIPOQ, CCL11, ENPP2, GJA1, IL5, IL6, INPP5D, KIT, TNF* |
| *PTGS2* | Enzyme | 1.79 × 10^-4^ | *AQP1, EDNRA, IL6, MAPK9, PPARG, RAC1, TGM2, TNF, TP53* |
| *MTOR* | Kinase | 1.82 × 10^-4^ | *ADM, CITED2, CLTC, EFNA1, FST, GAP43, GATA3, IL6, PPARG, S1PR1, TNF, TP53* |
| *PRKAA1* | Kinase | 1.85 × 10^-4^ | *IL6, ITIH5, LMO4, NFIB, TGM2, TNF, TP53* |
| *STAT5A* | Transcription regulator | 1.88 × 10^-4^ | *ADIPOQ, AXIN2, ID2, IL5, IL6, KIT, PPARG, STAT5A, STAT5B, TNF, TNNC1, TP53* |
| *IHH* | Enzyme | 1.88 × 10^-4^ | *BMP4, CTNNB1, MEF2C, PTCH1* |
| *CX3CL1* | Cytokine | 1.88 × 10^-4^ | *FGF2, IL6, PPARG, TNF* |
| *ITGA1* | Other | 1.88 × 10^-4^ | *DCN, PPARG, RAC1, TNF* |
| *ITGB2* | Trans-membrane receptor | 1.89 × 10^-4^ | *GATA3, IL6, RAC1, RAC2, TNF* |
| *ABCA1* | Transporter | 1.89 × 10^-4^ | *ADIPOQ, APP, IL6, PPARG, TNF* |
| *ANXA7* | Ion channel | 1.94 × 10^-4^ | *APC, CTSB, FST, FYN, GATA3, TFDP1* |
| Gemfibrozil | Chemical drug | 1.94 × 10^-4^ | *ACADVL, ANXA7, APP, IL6, PPARG, TNF* |
| *PDGF* complex  *(PDGF, PDGF-AA,AB,BB,CC)* | Complex | 1.97 × 10^-4^ | *COL4A1, FGF2, GJA1, HEY1, PIK3R3, PPARG, TP53* |
| *TNFSF10* | Cytokine | 1.97 × 10^-4^ | *CTNNB1, CTSB, IGF1R, IL5, IL6, TNF, TP53* |
| *CMKLR1* | G-protein coupled receptor | 2.01 × 10^-4^ | *ADIPOQ, IL6, TNF* |
| *MEOX1* | Transcription regulator | 2.01 × 10^-4^ | *BMP4, GLI2, MEF2C* |
| *PAWR* | Transcription regulator | 2.01 × 10^-4^ | *APP, CCL11, MAPK3* |
| *RELN* | Peptidase | 2.01 × 10^-4^ | *APP, BDNF, LYVE1* |
| Montelukast | Chemical drug | 2.01 × 10^-4^ | *CCL11, IL5, IL6* |
| *IC87114* | Chemical - kinase inhibitor | 2.01 × 10^-4^ | *CCL11, IL5, TNF* |
| Lidocaine | Chemical drug | 2.01 × 10^-4^ | *APP, GATA3, IL6* |
| *E64d* | Chemical - protease inhibitor | 2.01 × 10^-4^ | *APP, FGF2, TP53* |
| *APOA1* | Transporter | 2.11 × 10^-4^ | *APP, CDC42, IL6, RAC1, TNF* |
| *PRKACA* | Kinase | 2.11 × 10^-4^ | *ADIPOQ, BDNF, ID2, IL5, MYH10* |
| *MIR-16-5p* (and other miRNAs w/seed AGCAGCA) | Mature microRNA | 2.18 × 10^-4^ | *BDNF, CAPRIN1, FGF2, IGF1R, IL6, KITLG, MAP2K4, MAPK3, PAFAH1B2, RAF1* |
| *BMP*  *(BMP1-8, BMP10, BMP15)* | Group | 2.22 × 10^-4^ | *DPYSL2, HAND1, ID2, IL6* |
| *NOS1* | Enzyme | 2.22 × 10^-4^ | *ADIPOQ, BDNF, IL6, TNF* |
| Amitriptyline | Chemical drug | 2.22 × 10^-4^ | *BDNF, FGF2, TAGLN, TP53* |
| Acetylcholine | Chemical - endogenous mammalian | 2.22 × 10^-4^ | *IL6, KCNQ3, RAC1, TNF* |
| *FOXP3* | Transcription regulator | 2.23 × 10^-4^ | *AKT3, ID2, IL5, IL6, MAPK9, TNF, TRAF6* |
| *AGER* | Trans-membrane receptor | 2.27 × 10^-4^ | *APP, COL4A1, CTNNB1, IL6, TNF, TP53* |
| Troglitazone | Chemical drug | 2.31 × 10^-4^ | *ADIPOQ, APC, AXIN2, COL4A1, CTNNB1, DCN, IL5, IL6, PPARG, TNF, TP53, UPK1A, UPK1B, UPK2* |
| Epicatechin | Chemical drug | 2.34 × 10^-4^ | *DSE, HEY1, MAPK12, SNAP25, TNF* |
| *LEPR* | Trans-membrane receptor | 2.35 × 10^-4^ | *ADIPOQ, COL4A1, GAP43, ID2, IL6, PPARG, SNAP25, TCF7L2, TNF* |
| *MKNK1* | Kinase | 2.37 × 10^-4^ | *APC, GAP43, PAFAH1B1, SNAP25, TAGLN, TFDP1, TNF* |
| *MIR-155* | MicroRNA | 2.37 × 10^-4^ | *DOCK1, IL5, IL6, INPP5D, S1PR1, TNF, TP53* |
| Hydrocortisone | Chemical - endogenous mammalian | 2.41 × 10^-4^ | *ADM, FGF2, GAP43, GNAS, IL6, PPARG, STAT5A, TP53* |
| Bisindolylmaleimide I | Chemical - kinase inhibitor | 2.52 × 10^-4^ | *CTNNB1, GAP43, IL6, MAPK3, PLD1, PRKCA, TNF* |
| *IL-1R*  *(IL18RAP,*  *IL1R1,*  *IL1R2,*  *IL1RAP,*  *IL1RAPL1,*  *IL1RAPL2,*  *IL1RL1,*  *IL1RL2)* | Group | 2.60 × 10^-4^ | *APP, IL6, S100B, TNF* |
| *LPIN1* | Phosphatase | 2.60 × 10^-4^ | *ACADVL, IL6, PPARG, TNF* |
| *DLL4* | Other | 2.60 × 10^-4^ | *GATA3, HEY1, IL5, LYVE1* |
| *RCAN1* | Transcription regulator | 2.60 × 10^-4^ | *BDNF, GSK3B, IL6, TNF* |
| *LTF* | Peptidase | 2.60 × 10^-4^ | *IL5, IL6, TNF, TP53* |
| Folic acid | Chemical - endogenous mammalian | 2.60 × 10^-4^ | *APC, APP, FZD1, FZD6, TP53* |
| *IL13* | Cytokine | 2.64 × 10^-4^ | *ACADVL, BDNF, CCL11, CTSB, CTSH, ENPP2, FLOT1, GATA3, GSN, IL6, KITLG, PPARG, TGM2, TNF* |
| PP2/AG1879 tyrosine kinase inhibitor | Chemical - kinase inhibitor | 2.64 × 10^-4^ | *FGF2, IGF1R, IL6, RAC1, TP53, VAV1* |
| *ATF3* | Transcription regulator | 2.64 × 10^-4^ | *ADIPOQ, CTNNB1, GSN, IL6, TNF, TP53* |
| (+)-MK-801 | Chemical drug | 2.64 × 10^-4^ | *APP, BDNF, FGF2, GJA1, RAC1, TP53* |
| *PKA*  *(PKA* catalytic subunits) | Complex | 2.66 × 10^-4^ | *GHRL, GJA1, HAS2, IGF1R, IL5, IL6, PLD1, TNF* |
| *SRF* | Transcription regulator | 2.67 × 10^-4^ | *CALB1, CAPRIN1, CTNNB1, FST, GPM6A, GSN, MAPK10, PAFAH1B1, PDE2A, RAF1, SNX2, TAGLN, TNNC1* |
| Ionomycin | Chemical reagent | 2.67 × 10^-4^ | *BDNF, GATA3, IL5, IL6, KCNJ2, PPARG, RBPJ, STAT5B, TNF* |
| Rp-8-Br-camps | Chemical - kinase inhibitor | 2.74 × 10^-4^ | *BDNF, PRKACA, TNF* |
| (E)-4-hydroxy-3-methyl-but-2-enyl pyrophosphate | Chemical - endogenous non-mammalian | 2.74 × 10^-4^ | *IL5, IL6, TNF* |
| *TRIM38* | Other | 2.74 × 10^-4^ | *IL6, TNF, TRAF6* |
| *CSK* | Kinase | 2.74 × 10^-4^ | *FYN, IL6, TNF* |
| *IL17RB* | Trans-membrane receptor | 2.74 × 10^-4^ | *CCL11, IL5, IL6* |
| *MZF1* | Transcription regulator | 2.74 × 10^-4^ | *FGF2, PADI1, PRKCA* |
| Rosmarinic acid | Chemical - endogenous non-mammalian | 2.74 × 10^-4^ | *BDNF, IL6, TNF* |
| Albuterol | Chemical drug | 2.74 × 10^-4^ | *GNAS, IL6, TNF* |
| Streptozocin | Chemical drug | 2.74 × 10^-4^ | *CALB1, COL4A1, GHRL, GNAS, IGF1R, IL6, KIT, PPARG, S100B, TNF* |
| *YY1* | Transcription regulator | 2.79 × 10^-4^ | *BDNF, BMP4, COL19A1, FCER1A, HAS2, PFN2, TAGLN, TGM2, TNNC1, TP53, VAV1* |
| Methylselenic acid | Chemical reagent | 2.79 × 10^-4^ | *APOD, BMP4, CDC42, DPYSL2, ID2, IGF1R, MAPK3, MAPK9, PRKCA, TFDP1, WNT7A* |
| *TNFSF12* | Cytokine | 2.84 × 10^-4^ | *CCL11, HEY1, HEYL, IL6, MEF2A, MEF2C, TNF* |
| *INHA* | Growth factor | 2.84 × 10^-4^ | *COL4A3BP, F11R, FST, HAS2, KIT, KITLG, PIK3R1* |
| *CD36* | Trans-membrane receptor | 2.87 × 10^-4^ | *ADIPOQ, IGF1R, IL6, PPARG, TNF* |
| N-3 fatty acids | Chemical drug | 2.87 × 10^-4^ | *ADIPOQ, IL6, PIK3R1, PPARG, TNF* |
| *RAF1* | Kinase | 2.96 × 10^-4^ | *CDC42EP1, CHN1, CTNNB1, CTSH, IGF1R, IL6, PLD1, RAC1, TGM2, TNF* |
| *RAC1* | Enzyme | 3.01 × 10^-4^ | *CTNNB1, DPYSL2, FGF2, GSN, IL5, IL6, RAC1* |
| *USP7* | Peptidase | 3.02 × 10^-4^ | *IL6, PPARG, TNF, TP53* |
| *CTSB* | Peptidase | 3.02 × 10^-4^ | *APP, CTSB, RAC1, TNF* |
| *F2* | Peptidase | 3.09 × 10^-4^ | *CDC42EP1, COL4A1, FGF2, HAS2, IGF1R, IL6, MAPK3, RAC1, RAC2, S100B, TNF* |
| Methapyrilene | Chemical drug | 3.09 × 10^-4^ | *CTSB, ENPP2, GSK3B, ITIH4, NFIB, PRKCB, STMN1, TP53* |
| *ADM* | Other | 3.17 × 10^-4^ | *ADM, FGF2, IL6, PPARG, TNF* |
| *NR3C2* | Ligand-dependent nuclear receptor | 3.19 × 10^-4^ | *ARPC2, FGF2, IGF1R, IL6, SEMA3C, TNF, TP53* |
| Apigenin | Chemical - endogenous non-mammalian | 3.29 × 10^-4^ | *CTNNB1, DVL2, IL6, TAGLN, TNF, TP53* |
| Prostaglandin E2 | Chemical - endogenous mammalian | 3.31 × 10^-4^ | *APP, DCN, FGF2, FST, GJA1, HAS2, IGF1R, IL5, IL6, PPARG, TNF, TP53* |
| *BCL2* | Transporter | 3.38 × 10^-4^ | *AIF1, CTSH, GAP43, IL6, SNAP25, TNF, TP53* |
| Hedgehog  *(HHAT, IHH, SSH, DHH)* | Group | 3.48 × 10^-4^ | *BDNF, GATA5, KIT, PTCH1, TP53* |
| *WISP2* | Growth factor | 3.48 × 10^-4^ | *ADIPOQ, AXIN2, CTNNB1, GATA3, PPARG* |
| *NRL* | Transcription regulator | 3.48 × 10^-4^ | *BMP4, IGF1R, MEF2C, PIK3CA, PIK3R1* |
| *PGF* | Growth factor | 3.49 × 10^-4^ | *DCN, FST, IL6, TNF* |
| *MIR-25* | MicroRNA | 3.49 × 10^-4^ | *IL6, MAP2K4, TNF, TP53* |
| *MIR-92a-3p* (and other miRNAs w/seed AUUGCAC) | Mature microRNA | 3.49 × 10^-4^ | *ENPP6, IL6, MAP2K4, TNF* |
| *MEP1B* | Peptidase | 3.49 × 10^-4^ | *APP, RBPJ, TFDP1, TRAF6* |
| Bryostatin 1 | Chemical drug | 3.49 × 10^-4^ | *APP, IL6, PRKCA, TNF* |
| Suramin | Chemical drug | 3.49 × 10^-4^ | *FGF2, FST, IL6, TP53* |
| *HIF1A* | Transcription regulator | 3.52 × 10^-4^ | *ADIPOQ, ADM, ARPC2, AXIN2, CITED2, FYN, GJA1, ID2, IL6, MAFF, MEF2C, PRKCA, TNF, TP53* |
| *SMO* | G-protein coupled receptor | 3.53 × 10^-4^ | *FZD3, GLI2, HHIP, KRT5, PTCH1, PTCH2* |
| Lenalidomide | Chemical drug | 3.59 × 10^-4^ | *AGPS, CTNNB1, FZD10, NFIB, TCF7L2, TNF, WNT11, WNT2B, WNT8B* |
| *JUP* | Other | 3.62 × 10^-4^ | *AXIN2, CTNNB1, NME2* |
| *PDE3B* | Enzyme | 3.62 × 10^-4^ | *ADIPOQ, IL6, TNF* |
| *GRN* | Growth factor | 3.62 × 10^-4^ | *APP, IL6, TNF* |
| *GREM1* | Other | 3.62 × 10^-4^ | *AXIN2, TNF, WNT11* |
| Metoprolol | Chemical drug | 3.62 × 10^-4^ | *AQP1, GJA1, IL6* |
| Irinotecan | Chemical drug | 3.62 × 10^-4^ | *IL6, TNF, TP53* |
| *CREB*  *(ATF2,*  *ATF4,*  *CREB1,*  *CREB3,*  *CREB3L4,*  *CREB5,*  *CREBBP,*  *EP300)* | Group | 3.62 × 10^-4^ | *ADIPOQ, ADM, BDNF, CTSB, GJA1, GNAS, HAS2, IL6, LMO4, PPARG, PRKCA, TNF* |
| *IGF1R* | Trans-membrane receptor | 3.64 × 10^-4^ | *APP, CDC42, COL4A1, CTNNB1, ID2, IGF1R, IL6, KRT5, RAC1, TFDP1, TP53* |
| *RXRA* | Ligand-dependent nuclear receptor | 3.76 × 10^-4^ | *APP, CALB1, CTSH, HAS2, IL5, IL6, PLA2G2A, PLD1, PPARG, TFAP2A, TNF* |
| *MIR-30c-5p* (and other miRNAs w/seed GUAAACA) | Mature microRNA | 3.79 × 10^-4^ | *BDNF, IL6, MYO10, PAFAH1B2, PPARG, TNF, TP53* |
| *PIK3R1* | Kinase | 3.79 × 10^-4^ | *IL6, PIK3CA, PIK3R1, PIK3R2, PPARG, TNF* |
| Spironolactone | Chemical drug | 3.79 × 10^-4^ | *FGF2, IL6, KCNMA1, KCNMB1, PPARG, TNF* |
| *DGCR8* | Enzyme | 3.82 × 10^-4^ | *BDNF, GJA1, ID2, TAGLN, TCF7L2* |
| *MSTN* | Growth factor | 3.82 × 10^-4^ | *CAV3, IGF1R, IL6, MEF2A, MEF2C* |
| N-methyl-D-aspartate | Chemical reagent | 3.82 × 10^-4^ | *APP, BDNF, CALB1, FGF2, TNF* |
| *INS* | Other | 3.92 × 10^-4^ | *ACADVL, GHRL, IGF1R, IL6, PPARG, RDH10, TNF, TP53* |
| Kainic acid | Chemical toxicant | 3.92 × 10^-4^ | *APP, BDNF, CLTC, FGF2, GAP43, KCNQ3, TNF, TP53* |
| *LRP6* | Trans-membrane receptor | 4.01 × 10^-4^ | *APP, CTNNB1, GATA3, IL6* |
| *ZEB2* | Transcription regulator | 4.01 × 10^-4^ | *FCER1A, INPP5D, PLCG1, PLCG2* |
| *ALOX15* | Enzyme | 4.01 × 10^-4^ | *CTNNB1, IL6, PPARG, TNF* |
| *B2M* | Trans-membrane receptor | 4.01 × 10^-4^ | *IGF1R, IL6, MEF2A, MEF2C* |
| Fulvestrant | Chemical drug | 4.01 × 10^-4^ | *BMP4, CTNNB1, GJA1, ID2, IGF1R, PPARG, RAC1, SLC9A3R1, STMN1, TNF, TP53* |
| *MIR-34a-5p* (and other miRNAs w/seed GGCAGUG) | Mature microRNA | 4.19 × 10^-4^ | *AXIN2, FOXP1, PPARG, TAGLN, TP53* |
| *NOTCH3* | Transcription regulator | 4.19 × 10^-4^ | *APOD, HEY1, SDC2, TAGLN, TP53* |
| *RTN4* | Other | 4.19 × 10^-4^ | *APP, BDNF, DPYSL2, GAP43, IL6* |
| *BAX* | Transporter | 4.19E-04 | *CTSB, CTSH, IL6, TGM2, TNF* |
| *IL1A* | Cytokine | 4.25 × 10^-4^ | *APOD, APP, CCL11, FGF2, IL5, IL6, KIT, KITLG, PPARG, TNF* |
| *IKBKB* | Kinase | 4.27 × 10^-4^ | *CCL11, CTNNB1, CTSB, DCN, ENPP2, FYN, IL5, IL6, SEMA3C, TNF, TP53* |
| Fluoxetine | Chemical drug | 4.34 × 10^-4^ | *BDNF, FGF2, GNAS, IL6, NME2, TAGLN* |
| *IL10* | Cytokine | 4.39 × 10^-4^ | *ADM, CCL11, CTSB, DCN, EHF, IL5, IL6, INPP5D, KITLG, PLA2G2A, S100B, TNF, TRAF6* |
| Arundic acid | Chemical drug | 4.39 × 10^-4^ | *APP, S100B* |
| *PTCHD4* | Other | 4.39 × 10^-4^ | *PTCH1, PTCH2* |
| *C1QTNF1* | Other | 4.39 × 10^-4^ | *IL6, TNF* |
| *SPON2* | Other | 4.39 × 10^-4^ | *IL6, TNF* |
| *SCARB2* | Other | 4.39 × 10^-4^ | *IL6, TNF* |
| Satratoxin G | Chemical toxicant | 4.39 × 10^-4^ | *IL6, TNF* |
| *DHX15* | Enzyme | 4.39 × 10^-4^ | *IL6, TNF* |
| *GCA* | Other | 4.39 × 10^-4^ | *IL6, TNF* |
| *RAB10* | Enzyme | 4.39 × 10^-4^ | *IL6, TNF* |
| *DSG1* | Other | 4.39 × 10^-4^ | *IL5, TNF* |
| *STK38* | Kinase | 4.39 × 10^-4^ | *IL6, TNF* |
| *NLRC3* | Other | 4.39 × 10^-4^ | *IL6, TNF* |
| *TRIM30a/ TRIM30d* | Other | 4.39 × 10^-4^ | *IL6, TNF* |
| *ARHGEF2* | Other | 4.39 × 10^-4^ | *IL6, TNF* |
| *RPS7* | Other | 4.39 × 10^-4^ | *PIK3R1, TP53* |
| *DHCR7* | Enzyme | 4.39 × 10^-4^ | *IL6, TNF* |
| *PTPN12* | Phosphatase | 4.39 × 10^-4^ | *IL6, TNF* |
| *SNRNP70* | Other | 4.39 × 10^-4^ | *APP, PSEN2* |
| 14-oxodha | Chemical - endogenous mammalian | 4.39 × 10^-4^ | *IL6, TNF* |
| Gabexate | Chemical drug | 4.39 × 10^-4^ | *IL6, TNF* |
| Oseltamivir | Chemical drug | 4.39 × 10^-4^ | *IL6, TNF* |
| Ketotifen | Chemical drug | 4.39 × 10^-4^ | *IL6, TNF* |
| Diacerein | Chemical drug | 4.39 × 10^-4^ | *IL6, TNF* |
| Magnesium chloride | Chemical drug | 4.39 × 10^-4^ | *IL6, TNF* |
| Aripiprazole | Chemical drug | 4.39 × 10^-4^ | *BDNF, TNF* |
| Sr 31747 | Chemical drug | 4.39 × 10^-4^ | *IL6, TNF* |
| *SRC* | Kinase | 4.46 × 10^-4^ | *CTNNB1, CTSB, GJA1, HAS2, IGF1R, IL6, PRKCA* |
| *IRF4* | Transcription regulator | 4.50 × 10^-4^ | *GATA3, IL6, INPP5D, MAPK9, PRKCA, RAC1, SMARCA4, TNF* |
| Epigallo-catechingallate | Chemical drug | 4.56 × 10^-4^ | *APC, APP, CTNNB1, GJA1, IGF1R, IL6, PRKCA, TNF, TOLLIP, TP53* |
| *NOS2* | Enzyme | 4.57 × 10^-4^ | *ADIPOQ, APP, CAV3, COL4A1, GJA1, IL6, ITIH4, TNF, TNNC1* |
| Nicotine | Chemical drug | 4.57 × 10^-4^ | *BDNF, FGF2, GATA3, GHRL, GJA1, HAND1, IL6, TNF, TP53* |
| Prostaglandin d2 | Chemical - endogenous mammalian | 4.58 × 10^-4^ | *HAS2, IL5, IL6, TNF* |
| 3-methyladenine | Chemical toxicant | 4.58 × 10^-4^ | *ADIPOQ, IL6, TNF, TP53* |
| *MIR124 (a and b)* | Group | 4.58 × 10^-4^ | *IL6, TNF, TP53, TRAF6* |
| *ARRB2* | Other | 4.58 × 10^-4^ | *CTNNB1, GSK3B, IL6, TNF* |
| *PRKCQ* | Kinase | 4.58 × 10^-4^ | *CAV3, IL5, IL6, TNF* |
| *CLU* | Other | 4.58 × 10^-4^ | *APP, IL6, TNF, TP53* |
| *IL17RA* | Trans-membrane receptor | 4.58 × 10^-4^ | *CCL11, IL5, IL6, TNF* |
| R-win 55,212 | Chemical reagent | 4.58 × 10^-4^ | *IL6, TFDP1, TNF, TP53* |
| Proteasome inhibitor PSI | Chemical - protease inhibitor | 4.58 × 10^-4^ | *CAV3, IL6, PRKCA, TP53* |
| Glycyrrhizic acid | Chemical drug | 4.58 × 10^-4^ | *AIF1, GJA1, TNF, TP53* |
| *LYN* | Kinase | 4.64 × 10^-4^ | *BDNF, FYN, IL6, STAT5A, STAT5B, TNF* |
| *FZD8* | G-protein coupled receptor | 4.66 × 10^-4^ | *BMP4, CTNNB1, TP53* |
| *PLA2G4A* | Enzyme | 4.66 × 10^-4^ | *IL6, PLA2G2A, PLA2G5* |
| *IL1RAP* | Trans-membrane receptor | 4.66 × 10^-4^ | *IL5, IL6, TNF* |
| *TIMP3* | Other | 4.66 × 10^-4^ | *APP, IL6, KIT* |
| *SERPINB5* | Other | 4.66 × 10^-4^ | *CDC42, IL5, RAC1* |
| *PON1* | Phosphatase | 4.66 × 10^-4^ | *IL6, PPARG, TNF* |
| *HAVCR1* | Other | 4.66 × 10^-4^ | *GATA3, IL5, TNF* |
| *CCL3L3* | Cytokine | 4.66 × 10^-4^ | *IL5, IL6, TNF* |
| 1,2-dimethyl-hydrazine | Chemical toxicant | 4.66 × 10^-4^ | *GJA1, TNF, TP53* |
| To-901317 | Chemical reagent | 4.89 × 10^-4^ | *ADM, APOD, APP, IL5, IL6, ITIH1, ITIH4, PPARG, TGM2, TNF* |
| *BCL6* | Transcription regulator | 4.92 × 10^-4^ | *FGF2, GATA3, GLI2, IL5, IL6, PLA2G2A, TNF, TP53* |
| Sirolimus | Chemical drug | 4.96 × 10^-4^ | *ACADVL, ADM, APP, ARF1, DPYSL2, GAP43, GATA3, ID2, IGF1R, IL6, MAPK12, PIK3R1, PPARG, PRKCA, STMN1, TNF* |
| *HDAC5* | Transcription regulator | 5.00 × 10^-4^ | *CTNNB1, MEF2C, PPARG, TAGLN, TNF* |
| *IL6* | Cytokine | 5.05 × 10^-4^ | *APP, BDNF, CAPRIN1, CCL11, CTNNB1, DCN, ENPP2, FGF2, GAP43, ID2, IL5, IL6, KIT, PLA2G2A, PPARG, SNX10, TGM2, TNF, TP53, TRAF6* |
| Prednisolone | Chemical drug | 5.06 × 10^-4^ | *APC, APP, CDK5R1, CTSB, EDNRA, IL6, SDC2, TGM2, TNF, TP53* |
| HISTONE H4  *(HIST1H4J)* | Group | 5.14 × 10^-4^ | *ADIPOQ, HAS2, IL6, MEF2C, PPARG, TAGLN, TBX6, TNF* |
| *PLP1* | Other | 5.21 × 10^-4^ | *ENPP2, IL5, PTCH1, TNF* |
| *ABCG1* | Transporter | 5.21 × 10^-4^ | *APP, IL5, IL6, TNF* |
| Rosuvastatin | Chemical drug | 5.21 × 10^-4^ | *IL6, KIT, RAC1, TNF* |
| *MIR-10* | MicroRNA | 5.29 × 10^-4^ | *APC, GJA1, IGF1R, IL6, TNF, TP53* |
| Pravastatin | Chemical drug | 5.44 × 10^-4^ | *ADIPOQ, GJA1, IL6, TNF, TP53* |
| *PPARG* | Ligand-dependent nuclear receptor | 5.51 × 10^-4^ | *ADIPOQ, APP, ASAP1, CTNNB1, F11R, FST, FZD1, IL6, PLA2G2A, PLA2G3, PPARG, TAGLN, TNF, TP53, TRAF6* |
| *AMPK*  *(PRKAA,*  *PRKAA1,*  *PRKAA2,*  *PRKAB,*  *PRKAB1,*  *PRKAB2,*  *PRKAG,*  *PRKAG1,*  *PRKAG2)* | Complex | 5.51 × 10^-4^ | *ADIPOQ, CTNNB1, GJA1, IL6, PPARG, TNF, TP53* |
| *AG490* | Chemical - kinase inhibitor | 5.64 × 10^-4^ | *FGF2, IL6, MYH10, PLA2G2A, TAGLN, TNF* |
| *WT1* | Transcription regulator | 5.79 × 10^-4^ | *APC, CAPRIN1, COL4A1, CTNNB1, EFNA1, EXT2, IGF1R, RBPJ, SOS1, TFAP2A* |
| *PGR* | Ligand-dependent nuclear receptor | 5.82 × 10^-4^ | *DPYSL2, EDNRA, ELF5, GATA3, MYO10, PFN2, SLC9A3R1, SNAP25, STAT5A, STAT5B, TNF* |
| *SPRY1* | Other | 5.88E × 10^-4^ | *AXIN2, ID2, LMO4* |
| *SMPD1* | Enzyme | 5.88E × 10^-4^ | *CTSB, IL6, TNF* |
| Salmeterol | Chemical drug | 5.88E × 10^-4^ | *IL5, IL6, TNF* |
| Beta-1,3-glucan | Chemical drug | 5.88E × 10^-4^ | *FGF2, IL6, TNF* |
| Na^+^ | Chemical - endogenous mammalian | 5.88E × 10^-4^ | *CTNNB1, TNF, TP53* |
| *GAB2* | Other | 5.90 × 10^-4^ | *APP, GSN, IL6, TNF* |
| *MMP14* | Peptidase | 5.90 × 10^-4^ | *ANXA7, CTNNB1, GAP43, IL6* |
| *TRB* | Trans-membrane receptor | 5.90 × 10^-4^ | *CCL11, GATA3, IL5, TNF* |
| *IL4R* | Trans-membrane receptor | 5.90 × 10^-4^ | *GATA3, IL5, IL6, PPARG* |
| *PRKCB* | Kinase | 5.92 × 10^-4^ | *APP, CTNNB1, FGF2, IL6, PRKCB* |
| *MIR-124-3p* (and other miRNAs w/seed AAGGCAC) | Mature microRNA | 5.99 × 10^-4^ | *BDNF, BLOC1S6, DNM2, DVL2, F11R, GSK3B, GSN, MYO10, RDH10, TRAF6* |
| *PITX2* | Transcription regulator | 6.01 × 10^-4^ | *FZD3, FZD9, IRF6, TBX6, WNT2, WNT2B* |
| *ADCYAP1* | Other | 6.40 × 10^-4^ | *BDNF, ENPP2, FST, GAP43, GATA3, IL6, MAP2K7, NPY1R, SNAP25, TNF* |
| *IL32* | Cytokine | 6.42 × 10^-4^ | *CTNNB1, IL6, TNF, TP53, TRAF6* |
| Estrogen | Chemical drug | 6.56 × 10^-4^ | *BDNF, CALB1, GAP43, GJA1, IGF1R, IL6, KCNH1, RAF1, S1PR1, TNF, TP53* |
| Ethanol | Chemical - endogenous mammalian | 6.63 × 10^-4^ | *ADIPOQ, ADM, BDNF, BMP4, GNAS, GPM6A, HEY1, IL6, MAP2K4, PPARG, STAT5B, TNF, TP53* |
| *HSD11B1* | Enzyme | 6.65 × 10^-4^ | *ADIPOQ, IL6, INPP5D, TNF* |
| *CAMK4* | Kinase | 6.65 × 10^-4^ | *BDNF, IL6, TNF, TP53* |
| *TNC* | Other | 6.65 × 10^-4^ | *EDNRA, ID2, IL6, TNF* |
| *FOXP1* | Other | 6.65 × 10^-4^ | *GAP43, GJA1, IL6, MEF2C* |
| *HDAC*  *(HDAC1-11)* | Group | 6.66 × 10^-4^ | *BDNF, CTNNB1, EDNRA, GATA3, GLI2, IL6, SEMA3C, TNF* |
| *FAS* | Trans-membrane receptor | 6.79 × 10^-4^ | *CDC42EP2, CDK5R1, COL4A1, EFNA1, IL6, INPP5D, MAP2K4, MEF2C, PIK3CA, PLD1, PRKCB, TGM3, TNF* |
| Imatinib | Chemical drug | 6.80 × 10^-4^ | *APP, IL6, KIT, RAF1, STAT5A, TNF* |
| *NTRK2* | Kinase | 6.96 × 10^-4^ | *APP, BDNF, DNM2, SNAP25, TNF* |
| *MIR-155-5p* (miRNAs w/seed UAAUGCU) | Mature microRNA | 7.24 × 10^-4^ | *ARFIP2, CTNNB1, IL6, INPP5D, MYO10, PIK3R1, TCF7L2, TNF* |
| 15(s)-hete | Chemical - endogenous mammalian | 7.28 × 10^-4^ | *FGF2, MAPK3, PPARG* |
| Monophos-phoryl lipid A | Chemical reagent | 7.28 × 10^-4^ | *APP, IL6, TNF* |
| 2-(4-acetoxyphenyl)-2-chloro-N-methylethyl-amine | Chemical reagent | 7.28 × 10^-4^ | *IL5, IL6, TNF* |
| *UCP2* | Transporter | 7.28 × 10^-4^ | *ADIPOQ, IL6, TNF* |
| *FCER1A* | Trans-membrane receptor | 7.28 × 10^-4^ | *IL6, PLCG2, TNF* |
| 10-nitrooleate | Chemical - endogenous mammalian | 7.28 × 10^-4^ | *IL6, PPARG, TNF* |
| *ITIH1* | Other | 7.28 × 10^-4^ | *ITIH1, PPARG, TNF* |
| *ITGAL* | Trans-membrane receptor | 7.28 × 10^-4^ | *GATA3, IL5, TNF* |
| Pyridoxine | Chemical - endogenous mammalian | 7.28 × 10^-4^ | *APC, FZD1, FZD6* |
| Cobalt | Chemical toxicant | 7.28 × 10^-4^ | *FGF2, IL6, TNF* |
| Dextran sulfate | Chemical drug | 7.28 × 10^-4^ | *CCL11, CTNNB1, E2F8, IGF1R, IL5, IL6, S1PR1, STMN1, TGM2, TNF* |
| *AHR* | Ligand-dependent nuclear receptor | 7.30 × 10^-4^ | *ADM, APP, COL4A1, CTNNB1, DCN, E2F8, IL6, KIT, PLA2G12A, PPARG, TNF, TP53* |
| *TREM1* | Trans-membrane receptor | 7.44 × 10^-4^ | *IL5, IL6, KCNJ2, MAFF, NME7, PLD1, PLPP3, PPARG, TNF* |
| Lovastatin | Chemical drug | 7.45 × 10^-4^ | *ADIPOQ, GAP43, GATA3, IL5, IL6, PIK3R3, TNF* |
| *ACVRL1* | Kinase | 7.46 × 10^-4^ | *EFNA1, ID2, LYVE1, TAGLN* |
| Sildenafil | Chemical drug | 7.46 × 10^-4^ | *AIF1, APP, BDNF, TNF* |
| Diclofenac | Chemical drug | 7.46 × 10^-4^ | *APP, IL6, TMEM100, TNF* |
| *MAPK8* | Kinase | 7.55 × 10^-4^ | *APOD, AQP1, BMP4, IL5, IL6, PTCH1, TNF, TP53* |
| *COL18A1* | Other | 7.67 × 10^-4^ | *APC, CTNNB1, EFNA1, FGF2, IL6, TNF* |
| *EP300* | Transcription regulator | 7.77 × 10^-4^ | *ADIPOQ, AXIN2, CAV3, GATA3, GSK3B, ID2, IGF1R, IL6, IRF6, KCNIP3, MEF2C, TNF, TP53, WNT8B* |
| Docosa-hexaenoic acid | Chemical drug | 7.86 × 10^-4^ | *ADIPOQ, APP, BDNF, DPYSL2, IL6, PIK3R1, RAF1, TNF* |
| *TP73* | Transcription regulator | 7.90 × 10^-4^ | *CDC42EP2, CTNNB1, EDA, ERCC3, FGF2, FST, ID2, IL6, KIT, PIK3R3, STMN1, TP53* |
| *SIRT1* | Transcription regulator | 8.02 × 10^-4^ | *ADIPOQ, BDNF, CTNNB1, GATA5, GLI2, IGF1R, IL6, MEF2C, TNF, TP53* |
| *MMP9* | Peptidase | 8.13 × 10^-4^ | *CCL11, CTNNB1, CTSB, IL6, TNF* |
| Lysophos-phatidic acid | Chemical - other | 8.14 × 10^-4^ | *CCL11, IL6, PPARG, TAGLN, TNF, TP53* |
| *N-COR*  *(NCOR1, NCOR2)* | Group | 8.14 × 10^-4^ | *ADIPOQ, ASAP1, AXIN2, FZD1, PLA2G3, TBX6* |
| *LDLR* | Transporter | 8.14 × 10^-4^ | *AXIN2, GJA1, IL6, PPARG, TNF, WNT7A* |
| Gw9662 | Chemical reagent | 8.14 × 10^-4^ | *ADIPOQ, APP, IL6, PPARG, TNF, UPK2* |
| Phorbol esters | Chemical - other | 8.14 × 10^-4^ | *APP, IL5, IL6, PRKCA, PRKCB, TNF* |
| *EPAS1* | Transcription regulator | 8.28 × 10^-4^ | *ADM, CITED2, GJA1, IL6, ITIH5, MAFF, PIK3CA, PPARG, PRKCA* |
| *MSX2* | Transcription regulator | 8.35 × 10^-4^ | *BMP4, CTNNB1, PPARG, WNT7A* |
| *CD80* | Trans-membrane receptor | 8.35 × 10^-4^ | *IGF1R, IL5, IL6, TNF* |
| *OSM* | Cytokine | 8.47 × 10^-4^ | *CCL11, CDH3, CTSH, FGF2, GAP43, ID2, IL5, IL6, KRT17, MYH10, PPARG, STK4, TNF, TNNC1, TP53, UPK1A* |
| *SNCA* | Enzyme | 8.53 × 10^-4^ | *AIF1, BDNF, CTSB, GSK3B, GSN, TNF, TP53, UCHL1* |
| Thapsigargin | Chemical toxicant | 8.53 × 10^-4^ | *ADIPOQ, CSK, IL6, MEF2C, TGM2, TMEM100, TNF, TP53* |
| *JNK*  *(MAP2K4,*  *MAPK10,*  *MAPK12,*  *MAPK8,*  *MAPK9)* | Group | 8.57 × 10^-4^ | *APP, CCL11, GAP43, GJA1, IL5, IL6, TGM2, TNF, TP53* |
| *SREBF1* | Transcription regulator | 8.57 × 10^-4^ | *ADIPOQ, CAPZB, IL6, MEF2C, PIK3R3, PLA2G3, PPARG, TNF, TP53* |
| *N-acetyl-L-cysteine* | Chemical drug | 8.57 × 10^-4^ | *ADM, FGF2, IGF1R, IL5, IL6, PSEN2, RAF1, TNF, TP53* |
| *TCF*  *(HNF1A,*  *HNF1B,*  *TCF12,*  *TCF4,*  *TCF7,*  *TCF7L1,*  *TCF7L2,*  *ZEB1)* | Group | 8.62 × 10^-4^ | *APOD, BMP4, GJA1, KRT5, PTCH1, SEMA3C* |
| Treprostinil | Chemical drug | 8.71 × 10^-4^ | *IL6, TNF* |
| *KLK8* | Peptidase | 8.71 × 10^-4^ | *IL5, IL6* |
| *SNX27* | Other | 8.71 × 10^-4^ | *APP, PPARG* |
| *TNIP3* | Other | 8.71 × 10^-4^ | *IL6, TNF* |
| *B3GNT2* | Enzyme | 8.71 × 10^-4^ | *IL6, TNF* |
| *ZFP64* | Other | 8.71 × 10^-4^ | *IL6, TNF* |
| *RNF122* | Other | 8.71 × 10^-4^ | *IL6, TNF* |
| Enecadin | Chemical drug | 8.71 × 10^-4^ | *BDNF, FGF2* |
| *DACT3* | Other | 8.71 × 10^-4^ | *AXIN2, DVL2* |
| *APH-1* | Group | 8.71 × 10^-4^ | *APP, PSEN2* |
| *UNG* | Enzyme | 8.71 × 10^-4^ | *BDNF, TP53* |
| *SERPING1* | Other | 8.71 × 10^-4^ | *IL6, TNF* |
| *GALNS* | Enzyme | 8.71 × 10^-4^ | *BDNF, GAP43* |
| *SUFU* | Transcription regulator | 8.71 × 10^-4^ | *HHIP, PTCH1* |
| *MUC2* | Other | 8.71 × 10^-4^ | *IL6, TNF* |
| *SLC40A1* | Transporter | 8.71 × 10^-4^ | *IL6, TNF* |
| *FGA* | Other | 8.71 × 10^-4^ | *IL6, TNF* |
| *HLA-C* | Other | 8.71 × 10^-4^ | *IL6, TNF* |
| *ORM1* | Other | 8.71 × 10^-4^ | *IL6, TNF* |
| *A4GALT* | Enzyme | 8.71 × 10^-4^ | *IL6, TNF* |
| *CD163* | Trans-membrane receptor | 8.71 × 10^-4^ | *IL6, TNF* |
| *SIGLEC1* | Other | 8.71 × 10^-4^ | *IL6, TNF* |
| *CFH* | Other | 8.71 × 10^-4^ | *PPARG, TNF* |
| *PRMT2* | Enzyme | 8.71 × 10^-4^ | *IL6, TNF* |
| *APLP2* | Other | 8.71 × 10^-4^ | *GSK3B, TP53* |
| *ACOD1* | Enzyme | 8.71 × 10^-4^ | *IL6, TNF* |
| *PTCH2* | Trans-membrane receptor | 8.71 × 10^-4^ | *KITLG, PTCH2* |
| Graptopetalum paraguayense methanol extract | Chemical - endogenous non-mammalian | 8.71 × 10^-4^ | *IL6, TNF* |
| *PELI2* | Kinase | 8.71 × 10^-4^ | *IL6, TNF* |
| *IFIT2* | Other | 8.71 × 10^-4^ | *IL6, TNF* |
| *SCIN* | Other | 8.71 × 10^-4^ | *GSN, RAC2* |
| *CD300LD* | Other | 8.71 × 10^-4^ | *IL6, TNF* |
| *BST2* | Other | 8.71 × 10^-4^ | *IL6, TNF* |
| *HS6ST2* | Enzyme | 8.71 × 10^-4^ | *FGF2, IL6* |
| N-hydroxy-2,2-diphenylacetamide | Chemical reagent | 8.71 × 10^-4^ | *IL6, TNF* |
| *ITF3056* | Chemical reagent | 8.71 × 10^-4^ | *IL6, TNF* |
| Neopterin | Chemical - endogenous non-mammalian | 8.71 × 10^-4^ | *IL6, TNF* |
| Verlukast | Chemical drug | 8.71 × 10^-4^ | *IL5, TNF* |
| 1,1-dimethyl-4-phenylpiperazinium iodide | Chemical drug | 8.71 × 10^-4^ | *IL6, TNF* |
| Dihydrosphingosine 1-phosphate | Chemical - endogenous mammalian | 8.71 × 10^-4^ | *IL6, TNF* |
| Microcystin-LR | Chemical toxicant | 8.71 × 10^-4^ | *IL6, TNF* |
| 1-methyl-DL-tryptophan | Chemical drug | 8.71 × 10^-4^ | *IL6, TNF* |
| Vi capsular polysaccharide | Chemical drug | 8.71 × 10^-4^ | *IL6, TNF* |
| *EPO* | Cytokine | 8.75 × 10^-4^ | *BDNF, BMP4, FGF2, GATA3, IL6, KIT, NFIB, PRKCA, PRKCB, TNF, TP53* |
| Clozapine | Chemical drug | 8.77 × 10^-4^ | *BDNF, CTNNB1, GATA3, GSK3B, IL6* |
| Atorvastatin | Chemical drug | 8.88 × 10^-4^ | *ADIPOQ, BDNF, EDNRA, GJA1, IL6, MAPK3, PPARG, S100B, TMEM17* |
| *CHD7* | Enzyme | 8.88 × 10^-4^ | *GLI2, HEY1, RBPJ* |
| *GIP* | Other | 8.88 × 10^-4^ | *ADIPOQ, IL6, TNF* |
| *TNFRSF6B* | Trans-membrane receptor | 8.88 × 10^-4^ | *IL5, IL6, TNF* |
| *TNFSF4* | Cytokine | 8.88 × 10^-4^ | *IL5, IL6, TNF* |
| *MIR-221* | MicroRNA | 8.88 × 10^-4^ | *KIT, PIK3R1, STAT5A* |
| *EPHX2* | Enzyme | 8.88 × 10^-4^ | *BDNF, HEY1, IL6* |
| *DHX9* | Enzyme | 8.88 × 10^-4^ | *ID2, IL6, TNF* |
| *ITGB4* | Trans-membrane receptor | 8.88 × 10^-4^ | *ENPP2, IL6, TP53* |
| 101.10 peptide | Chemical reagent | 8.88 × 10^-4^ | *GJA1, IL6, TNF* |
| Phenytoin | Chemical drug | 8.88 × 10^-4^ | *BDNF, MAPK3, RAF1* |
| Pilocarpine | Chemical drug | 8.88 × 10^-4^ | *APP, BDNF, KCNQ3* |
| *IKBKG* | Kinase | 9.01 × 10^-4^ | *CTSB, DCN, ENPP2, IL6, PPARG, SEMA3C, TNF* |
| *IDH1* | Enzyme | 9.30 × 10^-4^ | *KCNB2, MAPK10, PPARG, PTCH1* |
| *MIR-182-5p (and other miRNAs w/seed UUGGCAA)* | Mature microRNA | 9.30 × 10^-4^ | *IGF1R, PIK3CA, RAC1, SOS1* |
| *AKT1* | Kinase | 9.37 × 10^-4^ | *ADIPOQ, COL4A1, CTNNB1, FGF2, IGF1R, IL6, PIK3R1, PPARG, TNF, TP53* |
| Medroxy-progesterone acetate | Chemical drug | 9.44 × 10^-4^ | *BDNF, DCN, FGF2, IL5, IL6, TNF, WNT16* |
| *F2R* | G-protein coupled receptor | 9.45 × 10^-4^ | *GJA1, IL6, TAGLN, TGM2, TP53* |
| *MAP3K7* | Kinase | 9.45 × 10^-4^ | *BMP4, HAS2, IL6, TNF, WNT11* |
| Sn50 peptide | Chemical toxicant | 9.45 × 10^-4^ | *GJA1, IL6, PPARG, TNF, TP53* |

^1^Upstream regulators control multiple genes in the dataset through direct or indirect relationships

^2^Molecule type of the upstream regulator as defined by the Ingenuity Pathway Analysis

^3^P-value with fisher’s exact test

^4^List of leading edge genes and positional candidate genes regulated by each upstream regulator
