# Supplementary material for: Loci and pathways associated with uterine capacity for pregnancy and fertility in beef cattle
Source: PLoS One. 2017 Dec 11;12(12):e0188997. doi: 10.1371/journal.pone.0188997 (PMC5724891; doi:10.1371/journal.pone.0188997)
Supplement: S2 Table — (DOCX) [file pone.0188997.s002.docx]

**S2 Table. Master regulators of positional candidates and leading edge genes associated with heifer fertility.**

| **Master regulator^1^** | **Molecule type^2^** | **Participating regulators^3^** | **p-value^4^** | **Target molecules in dataset^5^** |
| --- | --- | --- | --- | --- |
| *SOX2-OCT4* complex | Transcription regulators | *ACADVL, ADIPOQ, ADM, APC, APP, AQP1, AXIN2, BDNF, BMP4, CCL11, CDC42, CDH3, CDK5R1, CITED2, COL4A3BP, CTNNB1, CTSB, DCN, EFNA1, EHF, EMP2, ENPP2, EXT1, F11R, FGF2, FRZB, FST, FYN, GHRL, GJA1, GSK3B, HAS2, IGF1R, IL5, IL6, INPP5D, KCNJ2, KIT, KITLG, LYVE1, MAFF, MAP2K4, MAP2K6, MEF2C, MYH10, PDE2A, PLA2G2A, PLA2G3, PLA2G5, PPARG, PRKCA, RAC1, SDC2, SEMA3C, STAT5A, STMN1, TAGLN, TDRD7, TFAP2A, TGM2, TNF, TNNC1, TP53, TRAF6, WNT7A, ZNF750* | 1.2 x 10^-22^ | *ACADVL, ADM, AIF1, AKT3, APC, APOD, APP, AQP1, ARPC2, AXIN2, BMP4, CAPRIN1, CAPZB, CDC42, CDC42EP2, CDH3, CITED2, CLTC, COL4A1, COL4A3BP, CTNNB1, CTSB, CTSH, DCN, DOCK1, DPYSL2, DVL2, E2F8, EDA, EDNRA, EFNA1, EHF, ELF5, ENPP2, ERCC3, EXT2, F11R, FRZB, FST, FYN, FZD1, FZD3, GATA3, GATA5, GJA1, GLI2, GSK3B, GSN, HAND1, HAS2, HEY1, HHIP, ID2, IGF1R, IL5, IL6, INPP5D, IRF6, ITIH3, KCNMA1, KCNMB1, KCNQ3, KIF4A, KITLG, KRT17, KRT5, LYVE1, MAP2K4, MAP2K6, MAP2K7, MAPK11, MAPK12, MEF2C, MYH10, NFIB, PADI1, PAFAH1B2, PDE2A, PIK3CA, PIK3R1, PIK3R2, PIK3R3, PLA2G5, PLD1, PPARG, PRKACA, PRKCA, PSEN2, PTCH1, PTCH2, RAC1, RAF1, RBPJ, RDH10, SEMA3C, ST14, STAT5A, STAT5B, STMN1, STMN3, TBX6, TCF7L2, TDRD7, TFAP2A, TFDP1, TGFB1I1, TGM2, TMEM100, TMEM17, TNF, TNNC1, TRAF6, UCHL1, WNT16, WNT2, WNT2B, WNT7A, WNT8B* |
| *AHR* | Ligand-dependent nuclear receptor | *ACADVL, ADIPOQ, ADM, AIF1, APC, APOD, APP, AQP1, BDNF, BLOC1S6, BMP4, CALB1, CDC42, CDK5R1, CITED2, CTNNB1, CTSB, CTSH, DCN, EFNA1, ELF5, ENPP2, EXT2, FGF2, FLOT1, FST, GJA1, GLI2, GSK3B, HAND1, HAS2, ID2, IGF1R, IL6, IRF6, ITIH4, KIT, KITLG, KRT17, KRT5, MAP2K6, MEF2A, NPY1R, PADI1, PIK3R1, PIK3R2, PIK3R3, PLA2G10, PPARG, PRKCB, PSEN2, RAC1, RDH10, S1PR1, SDC2, SEMA3C, SLC9A3R1, SNAP25, SOS1, TNF, TNNC1, TP53, WNT11, WNT7A* | 2.21 x 10^-22^ | *ADIPOQ, ADM, AGPS, AIMP2, AKT3, ANXA7, APC, APP, AQP1, ARF1, ARFIP2, ATP6V0D1, BCCIP, BDNF, BMP4, CAPRIN1, CCL11, CDC42, CDC42EP1, CDC42EP2, CDH3, CDK5R1, CHN1, CITED2, COL4A1, COL4A3BP, CPE, CTNNB1, CTSB, CTSH, DCN, DNM2, DPYSL2, E2F8, EDNRA, EFNA1, ELF5, ENPP2, EXT2, FLOT1, FST, FZD1, FZD3, GATA3, GATA5, GHRL, GLI2, GNAS, GSN, ID2, IL5, IL6, INPP5D, ITIH3, ITIH4, ITIH5, KCNB2, KCNMA1, KCNMB1, KIF4A, KIT, KRT17, KRT5, KYAT3, MAP2K4, MAP2K6, MAP2K7, MAPK10, MAPK11, MAPK12, MAPK9, MEF2C, MYH10, NFIB, NME2, NPY1R, PAFAH1B1, PFN2, PIK3R2, PLA2G10, PLA2G12A, PLA2G2F, PLA2G5, PLCG1, PLCG2, PLD1, PPARG, PRKCB, PSEN2, PTCH1, PTCH2, RAC1, RAF1, RDH10, S100B, S1PR1, SCN1B, SDC2, SEMA3C, SLC35D1, SLC39A12, SLC9A3R1, SNX10, SOS1, STAT5A, STAT5B, STK4, STMN1, SUFU, TAGLN, TBX6, TCF7L2, TDRD7, TFAP2A, TMEM17, TNF, TNNC1, TTC30B, TTLL1, UCHL1, UPK1A, UPK1B, UPK2, VAV1, VAV2, WASF2, WNT2B, WNT8B* |
| Palmatine | Chemical toxicant | *ACADVL, AKT3, APC, APP, AXIN2, CDH3, CITED2, COL4A1, CSK, CTNNB1, CTSB, CTSH, DNM2, E2F8, EFNA1, ENPP2, ERCC3, F11R, FGF2, FYN, GSK3B, GSN, HAS2, ID2, IGF1R, IL5, IL6, KCNMA1, KIT, KITLG, MAP2K4, MAP2K6, MAP2K7, MAPK12, MAPK3, MYH10, MYO10, PAFAH1B2, PDE2A, PIK3R1, PIK3R3, PPARG, PRKCA, PRKCB, PSEN2, PTCH1, RAF1, RBPJ, S100B, SEMA3C, ST14, STMN1, TCF7L2, TFDP1, TGFB1I1, TGM2, TNF, TP53, WNT2, WNT7A* | 2.88 x 10^-22^ | *ADIPOQ, ADM, AKT3, APC, APP, AQP1, ARFIP2, ATP6V0D1, BCCIP, BDNF, BMP4, CCL11, CDC42, CDC42EP1, CDC42EP2, CDH3, CDK5R1, CHN1, CITED2, COL4A1, COL4A3BP, CPE, CTNNB1, CTSB, CTSH, DCN, DNM2, E2F8, EDNRA, EFNA1, ENPP2, FST, FZD1, GATA3, GHRL, GLI2, GNA11, GNAS, GSN, ID2, IL5, IL6, INPP5D, KCNMA1, KIF4A, KIT, KRT17, MAP2K4, MAP2K6, MAPK11, MAPK12, MAPK9, MEF2C, MYH10, NFIB, PFN2, PLA2G12A, PLA2G5, PLCG1, PLD1, PPARG, PRKACA, PRKCA, PSEN2, PTCH1, PTCH2, RAC1, RAC2, RAF1, RDH10, S1PR1, SDC2, SEMA3C, SOS1, STAT5A, STAT5B, STK4, STMN1, SUFU, TAGLN, TCF7L2, TDRD7, TFAP2A, TNF, TNNC1, WASF2* |
| *MAP2K4* | Kinase | *AKT3, CTNNB1, CTSH, DCN, FRZB, FZD1, FZD6, FZD9, GSN, IGF1R, IL6, MAPK3, PLA2G2A, PPARG, STAT5B, TNF, TP53, WNT11, WNT16, WNT2, WNT2B, WNT7A, WNT8B* | 9.95 x 10^-22^ | *ADIPOQ, ADM, AIF1, AIMP2, AKT3, APC, APOD, APP, AQP1, ARF1, ARFIP2, ATP6V0D1, BDNF, BMP4, CALB1, CAPZB, CAV3, CDC42, CDC42EP2, CDH3, CDK5R1, CITED2, COL4A1, CPE, CSK, CTSB, CTSH, DCN, DNM2, EHF, ENPP2, EXT2, F11R, FGF2, FZD1, GAP43, GATA3, GNA11, GNAS, GSK3B, GSN, HAND1, HEY1, ID2, IGF1R, IL6, IRF6, KCNB2, KCNMA1, KIT, KITLG, MAFF, MAP2K7, MAPK10, MAPK11, MAPK12, MAPK9, MEF2A, MEF2C, MYH10, NME2, PAFAH1B1, PFN2, PIK3R1, PLD1, PPARG, PRKCB, PTCH1, PTCH2, RAC1, RBPJ, S1PR1, SDC2, SEMA3C, SNAP25, SOS1, STAT5A, STAT5B, STK4, SUFU, TCF7L2, TDRD7, TFAP2A, TGFB1I1, TGM2, TNF, TNNC1, TP53, TRAF6, WASF2, WNT16, WNT7A, WNT8B* |
| Primaquine | Chemical drug | *AIF1, APP, AXIN2, BDNF, BMP4, CALB1, CDC42, CDH3, CDK5R1, CITED2, CLTC, CTNNB1, CTSB, DCN, DPYSL2, ENPP2, EXT1, FGF2, FYN, FZD3, GAP43, GSK3B, HEY1, IGF1R, IL6, LMO4, MAFF, MEF2C, PAFAH1B2, PIK3R1, PLCG1, PPARG, PRKACA, PRKCA, PRKCB, PTCH2, RBPJ, S100B, S1PR1, SMARCA4, SNAP25, STMN1, TAGLN, TNF, TP53, TRAF6, UCHL1* | 1.07 x 10^-21^ | *ADIPOQ, ADM, AKT3, APC, APP, AQP1, ARFIP2, ATP6V0D1, BCCIP, BDNF, BMP4, CCL11, CDC42, CDC42EP1, CDC42EP2, CDH3, CHN1, CITED2, COL4A1, COL4A3BP, CPE, CTNNB1, CTSB, CTSH, DCN, DNM2, E2F8, EDNRA, EFNA1, ENPP2, FLOT1, FST, FZD1, GAP43, GATA3, GHRL, GLI2, GNA11, GNAS, GSN, ID2, IL5, IL6, INPP5D, KCNJ2, KCNMA1, KIF4A, KIT, KRT17, MAFF, MAP2K7, MAPK11, MAPK12, MAPK9, MYH10, NFIB, PFN2, PLA2G12A, PLA2G5, PLCG1, PLD1, PPARG, PRKACA, PRKCA, PSEN2, PTCH1, PTCH2, RAC1, RAC2, RAF1, RDH10, S1PR1, SCN1B, SDC2, SEMA3C, SLC9A3R1, SOS1, STAT5A, STAT5B, STK4, STMN1, SUFU, TCF7L2, TDRD7, TFAP2A, TNF, TNNC1, WASF2* |
| *CD276* | Other | *APP, AXIN2, BDNF, BMP4, CTNNB1, FGF2, GATA3, GSK3B, PPARG, TAGLN, TNNC1, TP53, WNT11, WNT7A* | 1.35 x 10^-21^ | *AIF1, AIMP2, AKT3, APC, AQP1, ARF1, ARPC2, AXIN2, BCCIP, BMP4, CALB1, CCL11, CDC42, CDC42EP1, CDC42EP2, CDK5R1, CHN1, CSK, CTSH, DCN, DSE, DVL2, E2F8, EDA, EFNA1, EHF, ENPP2, FGF2, FRZB, FST, FZD3, GAP43, GATA3, GJA1, GNAS, GSN, HAS2, HEY1, HEYL, HHIP, ID2, IFT27, IGF1R, IL5, IL6, IRF6, ITIH5, KCNJ2, KCNMB1, KCNQ3, KIF4A, KIT, KITLG, LGR4, MAFF, MAP2K4, MEF2A, MEF2C, MYH10, MYO10, NFIB, PADI1, PIK3CA, PIK3R3, PLA2G5, PLA2G6, PLCG1, PLD1, PRKACA, PRKCA, PTCH2, RAC2, RBPJ, RDH10, S100B, S1PR1, SEMA3C, SMARCA4, SNAP25, SNX10, SOS1, STAT5B, STMN3, SUFU, TAGLN, TDRD7, TFAP2A, TFDP1, TGFB1I1, TGM2, TNF, TP53, TRAF6, UPK1A, UPK1B, UPK2, WNT11, WNT16* |
| 5-hydroxy-L-tryptophan | Chemical - endogenous mammalian | *ADIPOQ, APOD, APP, AXIN2, BMP4, CAPZB, COL4A1, CTNNB1, ENPP2, FST, FZD3, GAP43, GATA3, GJA1, GLI2, HHIP, ID2, IFT57, KIT, KRT5, PIK3R1, PPARG, PRKCSH, PTCH1, PTCH2, SDC2, SEMA3C, STAT5A, STAT5B, TCF7L2, TGM2, TNF, TP53, VAV1, WNT11, WNT16, WNT2* | 1.51 x 10^-21^ | *ADIPOQ, ADM, AIF1, AKT3, APC, APP, AQP1, ARFIP2, ATP6V0D1, BCCIP, BDNF, BMP4, CCL11, CDC42, CDC42EP1, CDC42EP2, CDH3, CDK5R1, CHN1, CITED2, COL4A1, COL4A3BP, CPE, CTNNB1, CTSB, CTSH, DCN, DNM2, E2F8, EDNRA, EFNA1, ENPP2, FST, FYN, FZD1, GATA3, GHRL, GLI2, GNAS, GSN, ID2, IL5, IL6, INPP5D, KCNMA1, KIF4A, KIT, KRT17, MAP2K4, MAP2K6, MAP2K7, MAPK12, MEF2C, MYH10, NFIB, PFN2, PLA2G12A, PLA2G5, PLCG1, PLD1, PPARG, PSEN2, PTCH1, PTCH2, RAC1, RAF1, RDH10, S1PR1, SDC2, SEMA3C, SOS1, STAT5A, STAT5B, STK4, STMN1, SUFU, TAGLN, TCF7L2, TDRD7, TFAP2A, TNF, TNNC1, WASF2* |
| Tg101348 | Chemical drug | *ADIPOQ, ADM, ANXA7, APP, AQP1, ASAP1, ATP6V0D1, BDNF, BMP4, CCL11, CDC42EP2, CDK5R1, CITED2, COL4A1, CTNNB1, CTSB, DCN, DVL2, EHF, ENPP2, FGF2, FST, FYN, GAP43, GHRL, GJA1, GSN, HEY1, ID2, IL5, IL6, INPP5D, ITIH4, KIT, MAFF, MAP2K7, MAPK9, PIK3R1, PLA2G2A, PLA2G2F, PLA2G5, PLD1, PPARG, PRKACA, PRKCA, PTCH1, S1PR1, STAT5A, STMN1, TCF7L2, TFDP1, TGM2, TNF, TNNC1, TOLLIP, TP53, TRAF6, UCHL1, VAV1* | 2.43 x 10^-21^ | *AIF1, AIMP2, AKT3, APC, AQP1, ARF1, ARPC2, AXIN2, BCCIP, BMP4, CALB1, CCL11, CDC42, CDC42EP1, CDC42EP2, CDK5R1, CHN1, CSK, CTSH, DCN, DSE, DVL2, E2F8, EDA, EFNA1, EHF, ENPP2, FGF2, FRZB, FST, FYN, FZD3, GAP43, GATA3, GJA1, GNAS, GSN, HAS2, HEY1, HEYL, HHIP, ID2, IFT27, IGF1R, IL5, IL6, IRF6, ITIH5, KCNJ2, KCNMB1, KCNQ3, KIF4A, KIT, KITLG, LGR4, MAFF, MAP2K4, MEF2A, MEF2C, MYH10, MYO10, NFIB, PADI1, PIK3CA, PIK3R3, PLA2G5, PLA2G6, PLCG1, PLD1, PRKACA, PRKCA, PTCH2, RAC2, RBPJ, RDH10, S100B, S1PR1, SEMA3C, SMARCA4, SNAP25, SNX10, SOS1, STMN3, SUFU, TAGLN, TDRD7, TFAP2A, TFDP1, TGFB1I1, TGM2, TP53, TRAF6, UPK1A, UPK1B, UPK2, VAV1, WNT11, WNT16* |
| *AHR* | Ligand-dependent nuclear receptor | *ADIPOQ, ADM, APP, AQP1, ARPC2, BDNF, BMP4, CAPRIN1, CCL11, CDK5R1, CITED2, COL4A1, CTNNB1, CTSB, CTSH, DCN, EDNRA, EXT1, EXT2, FCER1A, FGF2, FYN, FZD1, GATA3, GJA1, GLI2, GNAS, GSN, HAS2, HEY1, ID2, IL5, IL6, INPP5D, ITIH3, KCNMB1, KCNQ3, KCNV1, KIT, KITLG, KRT17, LYVE1, MAPK3, MEF2C, MYO10, NFIB, PFN2, PPARG, PRKCA, RAC1, STAT5A, STAT5B, TAGLN, TGFB1I1, TGM2, TMEM17, TNF, TP53, WNT11* | 3.56 x 10^-21^ | *ADIPOQ, ADM, AKT3, APC, APP, AQP1, ARFIP2, ATP6V0D1, BCCIP, BDNF, BMP4, CCL11, CDC42, CDC42EP1, CDC42EP2, CDH3, CDK5R1, CHN1, CITED2, COL4A1, COL4A3BP, CPE, CTNNB1, CTSB, CTSH, DCN, DNM2, E2F8, EDNRA, EFNA1, ENPP2, FST, FZD1, GATA3, GHRL, GLI2, GNAS, GSN, ID2, IL5, IL6, INPP5D, KCNMA1, KIF4A, KIT, KRT17, MAP2K4, MAP2K6, MAP2K7, MAPK11, MAPK12, MEF2C, MYH10, NFIB, PFN2, PLA2G12A, PLA2G5, PLCG1, PLD1, PPARG, PSEN2, PTCH1, PTCH2, RAC1, RAF1, RDH10, S1PR1, SDC2, SEMA3C, SOS1, STAT5A, STAT5B, STK4, STMN1, SUFU, TAGLN, TCF7L2, TDRD7, TFAP2A, TNF, TNNC1, WASF2* |
| CpG odn d-19 | Chemical reagent | *APC, AQP1, ARFIP2, ATP6V0D1, AXIN2, BCCIP, BMP4, CALB1, CDC42, CDC42EP2, CDK5R1, CPE, CTNNB1, CTSB, DNM2, EFNA1, ENPP2, FST, GATA3, GJA1, GLI2, GNAS, GSN, IGF1R, IL5, IL6, KCNMA1, MAP2K6, MAP2K7, MAPK11, MAPK12, MYH10, NFIB, PFN2, PTCH1, PTCH2, SCN1B, SEMA3C, SLC9A3R1, SOS1, STAT5A, STAT5B, STK4, SUFU, TCF7L2, TGM2, TNF, TP53, WASF2, WNT11* | 4.67 x 10^-21^ | *AIF1, AIMP2, AKT3, APC, AQP1, ARF1, ARPC2, AXIN2, BCCIP, BMP4, CALB1, CCL11, CDC42, CDC42EP1, CDC42EP2, CDK5R1, CHN1, CSK, CTSH, DCN, DSE, DVL2, E2F8, EDA, EFNA1, EHF, ENPP2, FGF2, FRZB, FST, FZD3, GAP43, GATA3, GJA1, GNAS, GSN, HAS2, HEY1, HEYL, HHIP, ID2, IFT27, IGF1R, IL5, IL6, IRF6, ITIH5, KCNJ2, KCNMB1, KCNQ3, KIF4A, KIT, KITLG, LGR4, MAFF, MAP2K4, MEF2A, MEF2C, MYH10, MYO10, NFIB, PADI1, PIK3CA, PIK3R3, PLA2G5, PLA2G6, PLCG1, PLD1, PRKACA, PRKCA, PTCH2, RAC2, RBPJ, RDH10, S100B, S1PR1, SEMA3C, SMARCA4, SNAP25, SNX10, SOS1, STAT5B, STMN3, SUFU, TAGLN, TDRD7, TFAP2A, TFDP1, TGFB1I1, TGM2, TP53, TRAF6, UPK1A, UPK1B, UPK2, WNT11, WNT16* |
| *MAP3K1* | Kinase | *APC, AXIN2, BMP4, CITED2, CTNNB1, EXT2, FRZB, FST, GATA3, GJA1, GLI2, GSK3B, HAND1, HEY1, ID2, IRF6, KITLG, KRT17, MEF2C, PIK3R1, PPARG, RBPJ, TBX6, WNT8B* | 5.09 x 10^-21^ | *ADM, AIF1, AKT3, APC, APOD, APP, AQP1, ARFIP2, ARPC2, ATP6V0D1, BDNF, CALB1, CDC42, CDC42EP1, CDC42EP2, CDH3, CDK5R1, CHN1, COL4A1, CPE, CSK, CTSB, CTSH, DCN, DNM2, DPYSL2, EDNRA, EHF, ELF5, ENPP2, FCER1A, FGF2, FRZB, FST, FZD1, GAP43, GATA3, GLI2, GNAS, GSN, HAND1, HAS2, IGF1R, INPP5D, KCNMA1, KIT, KITLG, KRT17, MAFF, MAP2K4, MAP2K7, MAPK12, MAPK9, MEF2C, MYH10, MYO10, PFN2, PIK3R1, PLA2G2A, PLCG2, PLD1, PRKACA, PTCH1, PTCH2, RBPJ, S1PR1, SDC2, SEMA3C, SNAP25, SNX10, SOS1, STAT5A, STAT5B, STMN1, SUFU, TCF7L2, TDRD7, TFDP1, TGFB1I1, TGM2, TNF, TNNC1, TRAF6, VAV1, WASF2, WNT16, WNT7A* |
| *AKT2* | Kinase | *ADM, APOD, APP, AQP1, BMP4, CALB1, CDC42, CDK5R1, CITED2, CLTC, COL4A1, CPE, CTNNB1, CTSB, DCN, ENPP2, ERCC3, FGF2, FST, FZD10, GAP43, GJA1, GLI2, GNAS, HAS2, HEY1, ID2, IGF1R, IL5, IL6, KCNJ2, KIT, KITLG, KRT5, MAPK10, MAPK9, MEF2C, NME2, PIK3R1, PLD1, PPARG, PRKCA, PRKCB, PTCH1, RBPJ, S100B, SMARCA4, TGM2, TNF, TP53, VAV1, VAV2, WNT2B, WNT8B* | 1.66 x 10^-20^ | *ADIPOQ, ADM, APC, APOD, APP, AQP1, ARFIP2, ATP6V0D1, AXIN2, BDNF, BMP4, CDC42, CDC42EP2, CDH3, COL4A1, CPE, CSK, CTNNB1, CTSB, DCN, DNM2, EFNA1, ENPP2, FGF2, FST, FYN, FZD1, FZD9, GAP43, GATA3, GLI2, GNAS, GSN, HAS2, HEY1, HHIP, ID2, IGF1R, IL5, IRF6, KCNMA1, KIT, MAP2K6, MAP2K7, MAPK11, MAPK12, MAPK3, MYH10, PFN2, PIK3CA, PIK3R1, PPARG, PTCH1, PTCH2, RAC1, RDH10, SDC2, SEMA3C, SNX10, SOS1, STAT5A, STAT5B, STK4, SUFU, TBX6, TCF7L2, TFDP1, TGFB1I1, TGM2, TNF, WASF2, WNT11, WNT2* |
| *GNL2* | Enzyme | *ADIPOQ, AQP1, AXIN2, BDNF, CCL11, CDK5R1, COL4A1, CTNNB1, EHF, ENPP2, FGF2, GJA1, GSK3B, GSN, IL5, IL6, MAPK3, PLD1, PPARG, PRKCB, RAC1, RAC2, SCN1B, TCF7L2, TGM2, TNF, TP53, UPK2* | 4.11 x 10^-20^ | *ACADVL, AIF1, AKT3, APP, AXIN2, BCCIP, BDNF, CALB1, CAPZB, CAV3, CCL11, CDC42, CDC42EP1, CDC42EP2, CDH3, CDK5R1, CHN1, CLTC, COL4A1, COL4A3BP, CPE, CSK, CTSB, CTSH, DPYSL2, DVL2, EDA, EDNRA, EFNA1, EHF, ELF5, ENPP2, ERCC3, FCER1A, FGF2, FOXP1, FST, FYN, FZD3, GAP43, GATA5, GNAS, GSK3B, GSN, HAND1, HAS2, HHIP, ID2, IFT27, IGF1R, INPP5D, ITIH3, ITIH4, ITIH5, KCNJ2, KCNMA1, KCNQ3, KIT, KRT17, KRT5, LYVE1, MAP2K4, MAP2K6, MAP2K7, MAPK11, MAPK12, MAPK3, MEF2A, MEF2C, MYH10, NFIB, PADI1, PAFAH1B2, PDE2A, PIK3CA, PIK3R1, PIK3R2, PIK3R3, PLA2G12A, PLA2G3, PLCG2, PLD1, PRKCA, PSEN2, PTCH1, PTCH2, RAC2, RAF1, RBPJ, RDH10, S100B, SEMA3C, SLC9A3R1, SMARCA4, ST14, STAT5A, STAT5B, STMN1, STMN3, TCF7L2, TDRD7, TFDP1, TGFB1I1, TGM2, TMEM100, TMEM17, TNNC1, TP53, UCHL1, WNT2, WNT2B, WNT7A* |
| Candesartan cilexetil | Chemical drug | *ADIPOQ, BDNF, BMP4, FGF2, HEYL, IGF1R, IL6, NME2, PAFAH1B1, PLD1, RAC1, S100B, SUFU, TNF, UPK1B, UPK2* | 7.48 x 10^-20^ | *ACADVL, ADM, AIF1, AKT3, APC, AQP1, ARF1, ARPC2, AXIN2, BDNF, CALB1, CAPZB, CCL11, CDC42, CDC42EP1, CHN1, COL4A3BP, CTSH, DCN, DVL2, EDNRA, EFNA1, EHF, EMP2, ENPP2, FGF2, FOXP1, FRZB, FST, FZD3, GAP43, GATA3, GJA1, GNA11, GNAS, GSK3B, GSN, HAS2, HEY1, HHIP, ID2, IGF1R, IL5, IL6, IRF6, KCNJ2, KCNMB1, KIF4A, KIT, KITLG, LMO4, MAFF, MAPK11, MAPK9, MEF2A, MEF2C, NCS1, NFIB, PAFAH1B1, PDE2A, PFN2, PIK3R3, PLA2G5, PLCG1, PRKCA, PTCH2, RAC2, RBPJ, RDH10, S1PR1, SEMA3C, SMARCA4, SNAP25, SNX10, STAT5B, SUFU, TAGLN, TFDP1, TGM3, TNNC1, TP53, TRAF6, UPK1A, UPK1B, UPK2, WNT11, WNT16* |
| Bleomycin | Chemical drug | *ADM, APP, ARF1, ASAP1, BMP4, CAV3, CDH3, COL4A1, CTNNB1, CTSB, FGF2, FZD1, GJA1, GSN, HAS2, HEY1, ID2, IGF1R, IL6, MAPK12, MAPK3, MYH10, PRKCA, PRKCB, RAF1, TAGLN, TGFB1I1, TGM2, TNF, TP53* | 1.37 x 10^-19^ | *ACADVL, ADIPOQ, AKT3, APOD, APP, AQP1, ARPC2, BDNF, BMP4, CALB1, CAPRIN1, CAV3, CCL11, CDH3, CDK5R1, CITED2, COL4A1, CTSH, DCN, EDNRA, EFNA1, EHF, ENPP2, EXT1, FCER1A, FGF2, FRZB, FST, FYN, FZD1, FZD6, GAP43, GATA3, GLI2, GNAS, GSK3B, HAS2, HEY1, ID2, IL6, INPP5D, ITIH3, KCNMA1, KCNQ3, KCNV1, KIT, KITLG, KRT17, LYVE1, MAP2K6, MAP2K7, MAPK12, MAPK3, MEF2C, MYH10, MYO10, PAFAH1B2, PDE2A, PFN2, PLD1, PLPP3, PRKCA, PSEN2, PTCH1, RBPJ, RDH10, S1PR1, ST14, STAT5B, STMN1, TAGLN, TCF7L2, TFDP1, TGFB1I1, TGM2, TMEM17, TNF, TNNC1, TP53, WNT11, WNT2, WNT7A* |
| Ruxolitinib | Chemical drug | *ADIPOQ, AKT3, GATA3, GSK3B, IL6, INPP5D, MAPK10, PIK3CA, PIK3R1, PIK3R2, PIK3R3, PPARG, PRKACA, RAF1, SOS1* | 1.45 x 10^-19^ | *AIF1, AIMP2, AKT3, APC, AQP1, ARF1, ARPC2, AXIN2, BCCIP, CALB1, CAPZB, CCL11, CDC42, CDC42EP1, CDC42EP2, CHN1, CSK, CTSH, DCN, DVL2, E2F8, EDA, EFNA1, EHF, EMP2, ENPP2, FGF2, FOXP1, FRZB, FST, FZD3, GAP43, GATA3, GJA1, GNAS, GSN, HAS2, HEY1, HEYL, HHIP, ID2, IGF1R, IL5, IL6, IRF6, ITIH5, KCNJ2, KCNMB1, KCNQ3, KIF4A, KIT, KITLG, LGR4, LMO4, MAFF, MAP2K4, MEF2A, MEF2C, MYH10, MYO10, NFIB, PADI1, PAFAH1B1, PDE2A, PIK3R3, PLA2G5, PLA2G6, PLCG1, PRKACA, PRKCA, PTCH2, RAC2, RBPJ, RDH10, S100B, S1PR1, SEMA3C, SMARCA4, SNAP25, SNX10, SOS1, STAT5B, STMN3, SUFU, TAGLN, TFAP2A, TFDP1, TGFB1I1, TGM3, TP53, TRAF6, UPK1A, UPK1B, UPK2, WNT11, WNT16* |
| Azd1480 | Chemical drug | *ADIPOQ, BDNF, CAV3, CCL11, CDC42EP1, CDH3, CDK5R1, CHN1, COL4A1, CTNNB1, CTSB, CTSH, FGF2, GAP43, GHRL, GJA1, GSK3B, HAS2, IGF1R, IL5, IL6, MAPK3, PIK3R1, PPARG, STAT5A, TNF, TP53, UPK2* | 1.45 x 10^-19^ | *AIF1, AIMP2, AKT3, APC, AQP1, ARF1, ARPC2, AXIN2, BCCIP, CALB1, CAPZB, CCL11, CDC42, CDC42EP1, CDC42EP2, CHN1, CSK, CTSH, DCN, DVL2, E2F8, EDA, EFNA1, EHF, EMP2, ENPP2, FGF2, FOXP1, FRZB, FST, FZD3, GAP43, GATA3, GJA1, GNAS, GSN, HAS2, HEY1, HEYL, HHIP, ID2, IGF1R, IL5, IL6, IRF6, ITIH5, KCNJ2, KCNMB1, KCNQ3, KIF4A, KIT, KITLG, LGR4, LMO4, MAFF, MAP2K4, MEF2A, MEF2C, MYH10, MYO10, NFIB, PADI1, PAFAH1B1, PDE2A, PIK3R3, PLA2G5, PLA2G6, PLCG1, PRKACA, PRKCA, PTCH2, RAC2, RBPJ, RDH10, S100B, S1PR1, SEMA3C, SMARCA4, SNAP25, SNX10, SOS1, STAT5B, STMN3, SUFU, TAGLN, TFAP2A, TFDP1, TGFB1I1, TGM3, TP53, TRAF6, UPK1A, UPK1B, UPK2, WNT11, WNT16* |
| Cimo | Chemical reagent | *CCL11, CDK5R1, FYN, GNA11, IL5, IL6, MAP2K4, MAP2K6, MAP2K7, PRKACA, PRKCA, TNF* | 1.48 x 10^-19^ | *AIF1, AIMP2, AKT3, APC, AQP1, ARF1, ARPC2, AXIN2, BCCIP, CALB1, CAPZB, CCL11, CDC42, CDC42EP1, CDC42EP2, CHN1, CSK, CTSH, DCN, DVL2, E2F8, EDA, EFNA1, EHF, EMP2, ENPP2, FGF2, FOXP1, FRZB, FST, FZD3, GAP43, GATA3, GJA1, GNAS, GSN, HAS2, HEY1, HEYL, HHIP, ID2, IGF1R, IL5, IL6, IRF6, ITIH5, KCNJ2, KCNMB1, KCNQ3, KIF4A, KIT, KITLG, LGR4, LMO4, MAFF, MAP2K4, MEF2A, MEF2C, MYH10, MYO10, NFIB, PADI1, PAFAH1B1, PDE2A, PIK3R3, PLA2G5, PLA2G6, PLCG1, PRKACA, PRKCA, PTCH2, RAC2, RBPJ, RDH10, S100B, S1PR1, SEMA3C, SMARCA4, SNAP25, SNX10, SOS1, STAT5B, STMN3, SUFU, TAGLN, TFAP2A, TFDP1, TGFB1I1, TGM3, TP53, TRAF6, UPK1A, UPK1B, UPK2, WNT11, WNT16* |
| *TRPS1* | Transcription regulator | *APC, CTNNB1, DVL2, FZD1, FZD3, GSK3B, MEF2A, SLC9A3R1, WNT16, WNT2B, WNT7A, WNT8B* | 1.84 x 10^-19^ | *ACADVL, ADIPOQ, ADM, AIMP2, AKT3, APOD, APP, AQP1, ARF1, ARPC2, ATP6V0D1, BDNF, CALB1, CAPRIN1, CAPZB, CCL11, CDC42, CDC42EP2, CDH3, CDK5R1, CITED2, CLTC, COL4A1, COL4A3BP, CTNNB1, CTSB, CTSH, DCN, DSE, DVL2, EDA, EDNRA, EHF, ENPP2, ENPP6, EXT1, EXT2, F11R, FCER1A, FGF2, FZD1, FZD3, GATA3, GHRL, GLI2, GNAS, GSN, HAND1, HAS2, HEY1, HEYL, HHIP, ID2, IFT27, IL5, IL6, INPP5D, ITIH3, ITIH4, ITIH5, KCNJ2, KCNMA1, KCNMB1, KCNQ3, KCNV1, KITLG, KRT17, LIN7C, LYVE1, MAP2K4, MAP2K7, MAPK12, MAPK3, MAPK9, MEF2C, MYO10, NFIB, NME2, PADI1, PAFAH1B2, PDE2A, PFN2, PIK3R1, PLA2G2A, PLA2G5, PLA2G6, PLD1, PPARG, PRKACA, PSEN2, PTCH1, PTCH2, RAC1, RAC2, RBPJ, RDH10, SDC2, SEMA3C, SNAP25, SNX10, ST14, STAT5A, STAT5B, STK4, STMN3, TAGLN, TFAP2A, TGFB1I1, TGM2, TGM3, TMEM17, TNF, TNNC1, TP53, WNT11, WNT16, WNT2, WNT7A, WNT8B* |
| Lmt-28 | Chemical reagent | *APP, AXIN2, BDNF, BMP4, CTNNB1, DCN, DVL2, FZD1, GAP43, GSK3B, HAS2, IL6, KIT, KITLG, MEF2C, PLD1, PPARG, TNF, WNT11* | 2.3 x 10^-19^ | *AIF1, AIMP2, AKT3, APC, AQP1, ARF1, ARPC2, AXIN2, BCCIP, BMP4, CALB1, CAPRIN1, CCL11, CDC42, CDC42EP1, CDC42EP2, CDK5R1, CHN1, CSK, CTSH, DSE, DVL2, E2F8, EDA, EFNA1, EHF, ENPP2, FGF2, FRZB, FST, FZD3, GAP43, GATA3, GJA1, GNAS, GSN, HAS2, HEY1, HEYL, HHIP, ID2, IFT27, IGF1R, IL5, IL6, IRF6, ITIH5, KCNJ2, KCNMB1, KCNQ3, KIT, KITLG, LGR4, LYVE1, MAFF, MAP2K4, MEF2A, MEF2C, MYH10, MYO10, NFIB, PADI1, PIK3CA, PIK3R3, PLA2G2A, PLA2G5, PLA2G6, PLCG1, PLD1, PRKACA, PRKCA, PSEN2, PTCH2, RAC2, RBPJ, RDH10, S100B, S1PR1, SMARCA4, SNAP25, SNX10, SOS1, STAT5B, STMN3, SUFU, TAGLN, TFAP2A, TFDP1, TGFB1I1, TNF, TP53, TRAF6, UPK1A, UPK1B, UPK2, WNT11, WNT16* |
| *NR2F2* | Ligand-dependent nuclear receptor | *ADIPOQ, ADM, AIF1, APP, AQP1, BDNF, CALB1, CCL11, CDK5R1, CSK, CTNNB1, CTSB, CTSH, EDNRA, EHF, F11R, FGF2, FZD1, GATA3, GJA1, GNAS, HAS2, IGF1R, IL5, IL6, KCNMA1, KITLG, KRT17, MAFF, MYH10, PLA2G2A, PLA2G5, PLCG1, PLD1, PPARG, PRKCA, PSEN2, RAC2, SNAP25, TCF7L2, TFDP1, TNF, TP53, TRAF6* | 2.95 x 10^-19^ | *ACADVL, ADIPOQ, ADM, AIF1, AKT3, APOD, APP, AQP1, ARPC2, AXIN2, CALB1, CAPRIN1, CAPZB, CDC42, CDC42EP2, CDH3, CLTC, COL4A1, COL4A3BP, CTNNB1, CTSB, CTSH, DCN, DPYSL2, DVL2, E2F8, EDA, EDNRA, EFNA1, EHF, ELF5, ENPP2, ERCC3, EXT2, F11R, FRZB, FYN, FZD1, FZD3, GATA5, GJA1, GSK3B, GSN, HAND1, HHIP, ID2, IGF1R, IL5, IL6, INPP5D, IRF6, ITIH3, KCNMA1, KCNMB1, KCNQ3, KRT17, KRT5, LYVE1, MAP2K4, MAP2K6, MAP2K7, MAPK11, MAPK12, MEF2A, MEF2C, MYH10, NFIB, PADI1, PAFAH1B2, PDE2A, PFN2, PIK3CA, PIK3R2, PIK3R3, PLA2G3, PLA2G5, PLD1, PPARG, PRKACA, PRKCA, PSEN2, PTCH1, PTCH2, RAC1, RAF1, RBPJ, RDH10, SEMA3C, ST14, STAT5A, STAT5B, STMN1, STMN3, TCF7L2, TDRD7, TFAP2A, TFDP1, TGFB1I1, TGM2, TMEM100, TMEM17, TNNC1, TP53, TRAF6, WNT16, WNT2, WNT2B, WNT7A, WNT8B* |
| Ferrous sulfate | Chemical drug | *CSK, FYN, GSK3B, MAP2K4, MAP2K6, MAP2K7, MAPK12, MAPK3, PRKCB, RAF1, STK4, TP53* | 3.99 x 10^-19^ | *ADIPOQ, AKT3, APP, BCCIP, BDNF, CALB1, CAV3, CDH3, CDK5R1, CITED2, COL4A1, COL4A3BP, CSK, CTNNB1, CTSB, DCN, DVL2, EFNA1, EHF, ENPP2, FGF2, FRZB, FST, GAP43, GJA1, GLI2, GNAS, GSK3B, HAS2, HEY1, ID2, IFT27, IL5, IL6, KCNJ2, KIT, KRT17, MAPK3, PIK3CA, PIK3R1, PLD1, PRKACA, RAC1, RBPJ, SEMA3C, SNX10, STAT5A, STMN1, TBX6, TCF7L2, TFDP1, TGFB1I1, TGM2, TNF, TRAF6, WNT16* |
| Psychosine | Chemical - endogenous mammalian | *APP, BDNF, CALB1, CAV3, CCL11, CTNNB1, EFNA1, EHF, FGF2, FST, GAP43, GATA3, GLI2, GNAS, HAS2, IL5, IL6, KIT, KRT17, PLD1, PPARG, PRKACA, RBPJ, STAT5A, TGM2, TNF, TOLLIP, TP53* | 5.61 x 10^-19^ | *ADIPOQ, ADM, APOD, APP, AQP1, BDNF, BMP4, CAV3, CCL11, CITED2, COL4A1, CTNNB1, CTSB, FOXP1, FST, FYN, FZD1, GAP43, GATA3, GJA1, GLI2, HAS2, ID2, IGF1R, IL5, IL6, IRF6, KCNJ2, PIK3CA, PLA2G6, PPARG, PRKCA, PTCH1, RAC1, STAT5A, STAT5B, TGM2, TNF, TP53* |
| Sulpiride | Chemical drug | *ADM, AIF1, APP, BMP4, CALB1, CCL11, CTNNB1, CTSB, DCN, EFNA1, EHF, ENPP2, FGF2, FST, GATA3, GJA1, GNAS, GSK3B, HAS2, IL6, MAP2K6, MEF2C, PLA2G2A, PLA2G3, PLA2G5, PLD1, PPARG, RAC2, S100B, SNAP25, STAT5A, TGM2, TNF, TRAF6* | 1.42 x 10^-18^ | *ACADVL, ADIPOQ, ADM, AIF1, AIMP2, AKT3, APOD, APP, AQP1, ARF1, ARPC2, ATP6V0D1, BDNF, CAPRIN1, CAPZB, CCL11, CDC42, CDC42EP1, CDC42EP2, CDH3, CHN1, CITED2, CLTC, COL4A1, CTNNB1, CTSB, CTSH, DCN, DVL2, EDA, EDNRA, ENPP2, EXT1, EXT2, F11R, FCER1A, FGF2, FZD1, FZD3, GATA3, GHRL, GLI2, GNAS, GSN, HAND1, HAS2, HEY1, HEYL, ID2, IFT27, IL5, IL6, INPP5D, ITIH3, ITIH4, ITIH5, KCNJ2, KCNMB1, KCNQ3, KCNV1, KITLG, KRT17, KRT5, LIN7C, LYVE1, MAP2K4, MAP2K7, MAPK11, MAPK12, MAPK3, MAPK9, MEF2A, MEF2C, MYO10, NFIB, NME2, PAFAH1B2, PDE2A, PFN2, PLA2G12A, PLA2G2A, PLA2G2F, PLA2G5, PLPP3, PPARG, PSEN2, PTCH1, PTCH2, RAC1, RAC2, SDC2, SEMA3C, SNAP25, SNX10, ST14, STAT5A, STAT5B, STMN3, TAGLN, TFAP2A, TGFB1I1, TGM2, TGM3, TMEM17, TNF, TNNC1, TP53, WNT11, WNT16, WNT2, WNT7A, WNT8B* |
| *IKK* complex  (*CHUK,*  *IKBKB,*  *IKBKE,*  *IKBKG)* | Complex | *BDNF, CALB1, CHN1, FST, GPM6A, IL6, MAPK10, MEF2C, PRKCA, PRKCB, SNAP25, TAGLN, TNF* | 1.45 x 10^-18^ | *ADIPOQ, AKT3, BCCIP, BDNF, CALB1, CAV3, CDH3, CDK5R1, CITED2, COL4A1, COL4A3BP, CSK, CTNNB1, CTSB, DCN, DVL2, EFNA1, EHF, ENPP2, FGF2, FRZB, FST, GAP43, GJA1, GLI2, GNAS, GSK3B, HAS2, HEY1, ID2, IFT27, IL5, IL6, KCNJ2, KIT, KRT17, MAPK3, PIK3CA, PIK3R1, PLD1, PRKACA, RAC1, RBPJ, SEMA3C, SNX10, STAT5A, STMN1, TBX6, TCF7L2, TFDP1, TGFB1I1, TGM2, TNF, TRAF6, WNT16* |
| Tpca-1 | Chemical - kinase inhibitor | *ACADVL, ADIPOQ, ADM, APOD, APP, AQP1, AXIN2, COL4A1, CTNNB1, E2F8, EFNA1, FGF2, FST, FZD6, GAP43, GSN, ID2, IGF1R, IL6, KRT5, MAFF, PPARG, STAT5A, TGM2, TNF, TP53* | 1.48 x 10^-18^ | *ACADVL, ADM, AIF1, AQP1, ARF1, ARPC2, BCCIP, BMP4, CALB1, CCL11, CDC42, CDC42EP2, CDH3, CLTC, CSK, CTNNB1, CTSB, CTSH, DCN, DVL2, EDNRA, EHF, ENPP2, ERCC3, FGF2, FRZB, FST, FYN, FZD3, GAP43, GATA3, GATA5, GJA1, GNAS, GSK3B, HAND1, HAS2, HEY1, ID2, IFT27, IGF1R, IL5, IL6, IRF6, KCNJ2, KCNMA1, KCNMB1, KIF4A, KIT, KITLG, MAFF, MAP2K4, MAP2K6, MAPK11, MAPK12, MAPK9, MYH10, PAFAH1B2, PDE2A, PFN2, PIK3R1, PIK3R2, PIK3R3, PLA2G5, PLCG2, PLD1, PRKACA, PRKCA, RAC2, RAF1, RBPJ, RDH10, S1PR1, SEMA3C, SMARCA4, SNX10, ST14, STAT5A, SUFU, TAGLN, TDRD7, TFAP2A, TFDP1, TGFB1I1, TMEM100, TNF, TP53, UPK1A, UPK1B, UPK2, WNT11, WNT16, WNT2, WNT2B, WNT7A* |
| Benomyl | Chemical toxicant | *ADIPOQ, APOD, APP, BMP4, CTNNB1, CTSB, GATA3, IGF1R, IL5, IL6, MAP2K7, PLPP3, PPARG, PRKCB, TNF, TRAF6, WNT2* | 1.55 x 10^-18^ | *ACADVL, AIF1, APC, APOD, AQP1, ARFIP2, ARPC2, ATP6V0D1, BCCIP, BDNF, BMP4, CALB1, CDC42, CDC42EP2, CDH3, COL4A3BP, CPE, CTNNB1, CTSH, DNM2, DSE, DVL2, E2F8, EDA, EDNRA, EFNA1, EHF, ENPP2, ERCC3, FCER1A, FLOT1, FOXP1, FST, FYN, FZD3, GJA1, GNAS, GSK3B, GSN, HHIP, IFT27, IGF1R, IL6, INPP5D, IRF6, ITIH5, KCNJ2, KCNMA1, KCNQ3, KITLG, KRT5, LGR4, LMO4, MAP2K4, MAP2K6, MAP2K7, MAPK11, MAPK12, MAPK3, MEF2A, MEF2C, MYH10, PADI1, PAFAH1B2, PFN2, PLA2G12A, PLA2G5, PLA2G6, PLCG1, PLD1, PLPP3, PPARG, PRKCA, PSEN2, PTCH1, PTCH2, RAC2, RAF1, RBPJ, SCN1B, SEMA3C, SLC9A3R1, SNAP25, SOS1, ST14, STAT5A, STAT5B, STK4, SUFU, TAGLN, TCF7L2, TDRD7, TFAP2A, TGFB1I1, TGM2, TRAF6, WASF2, WNT11, WNT16, WNT2, WNT7A* |
| *TRAF2* | Enzyme | *ADM, BMP4, COL4A1, EFNA1, IL6, KITLG, MAPK9, MYH10, PLPP3, PPARG, S1PR1, TNF, TRAF6, VAV1* | 1.58 x 10^-18^ | *ACADVL, ADIPOQ, ADM, APOD, AQP1, AXIN2, BCCIP, BDNF, BMP4, CALB1, CAV3, CDH3, CDK5R1, CITED2, COL4A3BP, CSK, CTSB, DCN, EFNA1, EHF, ENPP2, FGF2, FST, FYN, GAP43, GATA3, GLI2, GNAS, GSN, HAS2, HEY1, ID2, IL6, IRF6, ITIH3, KIT, KITLG, KYAT3, MAFF, MAPK9, PFN2, PIK3R1, PLA2G12A, PLA2G3, PLA2G6, PLD1, PRKACA, PTCH1, RAC1, RBPJ, SDC2, SEMA3C, SNX10, STAT5A, TAGLN, TCF7L2, TFDP1, TGFB1I1, TGM2, TMEM17, TNF, TNNC1, TRAF6, UCHL1, WNT11, WNT7A* |
| Fc receptor  *(FCAMR,  FCAR,*  *FCER1A,*  *FCER1G,*  *FCER2,*  *FCGR1A,*  *FCGR1B,*  *FCGR2A,*  *FCGR2B,*  *FCGR2C,*  *FCGR3A/FCGR3B,*  *FCR,*  *INPP5D,*  *INPPL1)* | Group | *ADIPOQ, ADM, BDNF, CAV3, COL4A1, EDNRA, FGF2, GAP43, GJA1, GSK3B, HAS2, HEY1, IGF1R, IL6, KITLG, MAP2K7, MYH10, PIK3R1, PLA2G10, PPARG, RAC1, TNF, TP53* | 2.29 x 10^-18^ | *ADIPOQ, ADM, APP, ARPC2, BDNF, BMP4, CAV3, CCL11, CDK5R1, CITED2, CTNNB1, EFNA1, ENPP2, FGF2, FOXP1, FZD1, GAP43, GLI2, GNAS, HAS2, HEY1, ID2, IGF1R, IL5, IRF6, KCNJ2, KITLG, LMO4, MAFF, MAP2K4, MAP2K6, MAPK3, MEF2A, MEF2C, PIK3CA, PLA2G2A, PLA2G5, PLA2G6, PLCG2, PLD1, PPARG, PRKCA, S1PR1, STAT5A, STMN1, TGM2, VAV1* |
| Sc-58125 | Chemical drug | *APP, AXIN2, BMP4, CTNNB1, DCN, GATA3, GSK3B, HEYL, MAPK10, PLD1, PPARG, TP53* | 2.38 x 10^-18^ | *ACADVL, ADIPOQ, ADM, APC, APP, AQP1, AXIN2, BDNF, BMP4, CCL11, CDC42, CDH3, CDK5R1, CITED2, COL4A1, COL4A3BP, CTNNB1, CTSB, DCN, EDNRA, EFNA1, EHF, EMP2, ENPP2, EXT1, F11R, FGF2, FST, FYN, GJA1, GSK3B, HAND1, HAS2, HEY1, IGF1R, IL5, IL6, KCNJ2, KIT, KITLG, LYVE1, MAFF, MAP2K4, MAP2K6, MAPK9, MEF2C, MYH10, PDE2A, PLA2G2A, PLA2G3, PLA2G5, PRKCB, RAC1, SDC2, SEMA3C, STAT5A, STMN1, TAGLN, TDRD7, TFAP2A, TGM2, TNF, TP53, TRAF6, WNT7A, ZNF750* |
| *UBE4B* | Enzyme | *ADM, BDNF, CPE, DCN, ENPP2, FST, HAS2, MEF2C, MYH10, PLA2G2A, RBPJ, TCF7L2, TP53* | 4.37 x 10^-18^ | *ACADVL, ADM, AIF1, AKT3, APOD, APP, AQP1, ARPC2, AXIN2, CALB1, CAPRIN1, CAPZB, CDC42, CDH3, CITED2, CLTC, CTNNB1, CTSB, CTSH, DCN, DPYSL2, DSE, DVL2, E2F8, EDA, EDNRA, EFNA1, EHF, ELF5, ENPP2, F11R, FOXP1, FRZB, FST, FYN, FZD3, GATA5, GSK3B, GSN, HAND1, HAS2, HHIP, ID2, IFT27, IGF1R, IL5, IL6, IRF6, ITIH3, KCNMA1, KCNMB1, KCNQ3, KRT5, KYAT3, LMO4, LYVE1, MAP2K4, MAP2K6, MAP2K7, MAPK11, MAPK12, MYH10, NFIB, PADI1, PAFAH1B2, PDE2A, PIK3CA, PIK3R2, PIK3R3, PLA2G5, PPARG, PRKACA, PRKCA, PSEN2, PTCH1, PTCH2, RAC2, RAF1, RBPJ, S100B, SEMA3C, ST14, STMN1, STMN3, SUFU, TAGLN, TCF7L2, TDRD7, TFAP2A, TFDP1, TGFB1I1, TGM2, TMEM100, TMEM17, TNF, TNNC1, TP53, TRAF6, UCHL1, UPK1A, UPK1B, UPK2, WNT16, WNT2, WNT2B, WNT7A* |
| Parthenolide | Chemical drug | *ADM, APP, BDNF, CTNNB1, CTSB, FGF2, GAP43, ID2, IL6, IRF6, KCNJ2, MAPK3, PLA2G6, PPARG, PRKCA, TNF* | 5.31 x 10^-18^ | *ADIPOQ, AGPS, APOD, AQP1, ARF1, AXIN2, BCCIP, BMP4, CAV3, CDH3, CITED2, COL4A1, COL4A3BP, CSK, CTNNB1, CTSB, DCN, EFNA1, EHF, ENPP2, FST, GAP43, GATA3, GJA1, GLI2, GNA11, GNAS, HAS2, HEY1, ID2, IL6, IRF6, ITIH4, KCNB2, KIT, KRT17, LMO4, MAFF, MAPK10, MEF2C, PAFAH1B1, PFN2, PIK3CA, PIK3R1, PLD1, PRKACA, PRKCB, PTCH1, RBPJ, RDH10, S1PR1, SEMA3C, STAT5A, TAGLN, TFDP1, TGFB1I1, TGM2, TNF, TNNC1, TP53, TTC30B, TTLL1* |
| Argatroban | Chemical drug | *ADIPOQ, AQP1, BMP4, CALB1, CDK5R1, COL4A1, CTSB, DCN, FGF2, FST, GHRL, GJA1, IGF1R, IL6, NPY1R, PICK1, PLA2G6, PLCG1, PPARG, PRKCB, RDH10, SNAP25, TGM2, TNF, TP53* | 6.39 x 10^-18^ | *AIF1, AIMP2, AKT3, APC, APOD, ARPC2, BCCIP, CALB1, CAPRIN1, CAPZB, CDC42EP1, CDC42EP2, CDK5R1, COL4A1, COL4A3BP, CPE, CSK, CTSH, DOCK1, DPYSL2, DSE, E2F8, EDNRA, EFNA1, EHF, ELF5, ENPP2, EXT1, EXT2, FCER1A, FGF2, FOXP1, FRZB, GAP43, GJA1, GNA11, GNAS, HAND1, HAS2, HEYL, IFT27, IGF1R, IL6, INPP5D, IRF6, ITIH3, ITIH5, KCNJ2, KCNQ3, KCNV1, LIN7C, LMO4, LYVE1, MAFF, MAP2K4, MAPK11, MAPK3, MEF2C, MYH10, NFIB, NME2, PADI1, PAFAH1B1, PIK3R2, PLA2G2F, PLA2G5, PLA2G6, PLCG1, PLD1, PLPP3, PRKACA, PRKCA, RAC1, RAC2, RBPJ, S100B, S1PR1, SDC2, SEMA3C, SMARCA4, SOS1, STMN3, TAGLN, TCF7L2, TDRD7, TFDP1, TGM2, TMEM17, TNF, TNNC1, TP53, VAV1, VAV2, WNT11, WNT16, WNT2, WNT7A, WNT8B* |
| *SUZ12* | Enzyme | *ADIPOQ, BDNF, DOCK1, FYN, IL6, PLA2G5, RAC1, RAC2, TNF, VAV1* | 6.64 x 10^-18^ | *ADM, AIF1, AKT3, ARF1, ARPC2, AXIN2, BMP4, CAV3, CCL11, CDC42, CDH3, CDK5R1, CTNNB1, DCN, ENPP2, ERCC3, FGF2, FRZB, FST, FYN, FZD3, GAP43, GJA1, GSK3B, GSN, HAS2, HEY1, HHIP, ID2, IFT27, IGF1R, IL5, IRF6, KCNJ2, KCNMB1, KIF4A, KIT, KITLG, KRT17, MAFF, MAP2K4, MAPK3, MEF2A, MEF2C, PFN2, PIK3CA, PIK3R3, PLA2G5, PPARG, PRKACA, PRKCA, PTCH2, RAC2, RBPJ, RDH10, S1PR1, SEMA3C, SMARCA4, SNX10, STAT5A, STAT5B, SUFU, TAGLN, TCF7L2, TFAP2A, TFDP1, TGM2, TNF, TNNC1, TP53, UPK1A, UPK1B, UPK2, WNT11, WNT16* |
| *ZFP36* | Transcription regulator | *ADIPOQ, CDC42, COL4A1, CTNNB1, EDNRA, FGF2, FST, GJA1, HAND1, HEY1, IL6, PRKCA, PRKCB, TGM2, TP53* | 7.19 x 10^-18^ | *ACADVL, ADM, AGPS, AIMP2, APC, APOD, APP, AQP1, ARFIP2, ATP6V0D1, AXIN2, BCCIP, BDNF, BMP4, CAPZB, CCL11, CDC42EP1, CDC42EP2, CDH3, CDK5R1, CHN1, CITED2, CLTC, COL4A3BP, CPE, CSK, DCN, DNM2, DOCK1, DSE, EDNRA, EFNA1, EHF, EMP2, ENPP2, EXT1, EXT2, F11R, FGF2, FST, FYN, GATA3, GHRL, GJA1, GSK3B, HAS2, HEY1, HEYL, HHIP, ID2, IGF1R, IL5, IL6, ITIH4, KCNB2, KCNJ2, KCNMB1, KIF4A, KIT, KITLG, LGR4, LMO4, LYVE1, MAFF, MAP2K4, MAP2K6, MAPK10, MAPK9, MEF2C, MYH10, NME2, PAFAH1B1, PDE2A, PIK3R3, PLA2G12A, PLA2G2A, PLA2G3, PLA2G5, PLPP3, PPARG, PRKACA, PRKCA, PRKCB, PTCH1, RBPJ, S1PR1, SDC2, SEMA3C, SLC35D1, SMARCA4, SNX10, SOS1, STAT5A, STK4, STMN1, STMN3, SUFU, TAGLN, TDRD7, TFAP2A, TFDP1, TGM2, TNF, TRAF6, TTC30B, TTLL1, UCHL1, UPK1A, UPK1B, UPK2, VAV1, WASF2, WNT7A, WNT8B, ZNF750* |
| *NCOR1* | Transcription regulator | *ADIPOQ, COL4A1, DVL2, GATA3, GJA1, ID2, MAPK12, MEF2C, PIK3R1, PLPP3, PPARG, TNNC1* | 8.2 x 10^-18^ | *ACADVL, ADIPOQ, AKT3, APC, APOD, APP, AQP1, ARFIP2, ATP6V0D1, BCCIP, BDNF, BMP4, CALB1, CCL11, CDC42, CDC42EP2, CDH3, COL4A1, CPE, DNM2, DPYSL2, EDNRA, EFNA1, ELF5, ENPP2, F11R, FLOT1, FZD1, FZD6, GAP43, GATA3, GJA1, GNAS, GSN, HAS2, IL5, KCNJ2, KCNMA1, KIT, KYAT3, MAFF, MAP2K6, MAP2K7, MAPK11, MAPK12, MAPK3, MEF2C, MYH10, MYO10, NME2, NPY1R, PFN2, PLA2G2A, PLCG1, PPARG, PRKCA, PTCH1, PTCH2, SCN1B, SEMA3C, SOS1, STAT5A, STAT5B, STK4, SUFU, TCF7L2, TGM2, TRAF6, WASF2, WNT11, WNT16, WNT2, WNT2B, WNT7A, WNT8B* |
| *MIR-155-5p* (miRNAs w/seed UAAUGCU) | Mature microRNA | *ADIPOQ, ADM, CCL11, CTSB, FGF2, FST, GAP43, GATA3, GJA1, HAS2, HEY1, ID2, IGF1R, IL5, IL6, KRT17, PLA2G10, PLA2G2A, RBPJ, S1PR1, TAGLN, TFDP1, TNF, TP53* | 9.06 x 10^-18^ | *ACADVL, ADM, APC, APOD, AQP1, ARF1, ARFIP2, ARPC2, BCCIP, CALB1, CCL11, CDC42, CDH3, CDK5R1, COL4A3BP, CSK, CTNNB1, DCN, DSE, DVL2, F11R, FGF2, FRZB, FST, FZD3, GAP43, GATA3, GJA1, GNAS, GSK3B, HAS2, HEY1, HHIP, ID2, IGF1R, IL5, IL6, INPP5D, IRF6, KCNJ2, KCNMA1, KCNMB1, KIF4A, KIT, KITLG, MAFF, MAP2K4, MAP2K7, MAPK12, MEF2C, MYO10, PAFAH1B1, PAFAH1B2, PDE2A, PFN2, PIK3CA, PIK3R1, PIK3R3, PLA2G2A, PLA2G5, PLCG1, PLD1, PSEN2, PTCH2, RAC2, RBPJ, RDH10, S1PR1, SEMA3C, SMARCA4, SNAP25, SNX10, ST14, STAT5A, STAT5B, SUFU, TAGLN, TCF7L2, TDRD7, TFAP2A, TFDP1, TGFB1I1, TNF, TP53, TRAF6, UPK1A, UPK1B, UPK2, VAV1, WNT11, WNT16, WNT2, WNT7A* |
| *SUFU* | Transcription regulator | *CTNNB1, FZD1, FZD3, GSK3B, SLC9A3R1, WNT16, WNT2B, WNT8B* | 9.59 x 10^-18^ | *ACADVL, ADM, APP, ARPC2, BDNF, BMP4, CALB1, CCL11, CDK5R1, CITED2, CLTC, COL4A1, COL4A3BP, ENPP2, FYN, FZD1, GAP43, GATA3, GATA5, GJA1, GLI2, GSN, HAND1, HAS2, HEY1, HHIP, ID2, IL6, IRF6, KCNJ2, KCNMB1, KIT, KRT17, MAFF, MAP2K4, MAPK11, MAPK3, MEF2A, MEF2C, PIK3CA, PIK3R1, PLA2G2A, PLA2G5, PLA2G6, PLD1, PRKACA, PTCH1, PTCH2, RAC1, RDH10, S1PR1, SDC2, SEMA3C, SOS1, STAT5B, STMN1, STMN3, TMEM100, TP53, TRAF6, VAV2, WNT16, WNT2B* |
| *ERBB2* | Kinase | *ADIPOQ, ADM, AIMP2, AQP1, ENPP2, FST, FZD1, GJA1, ID2, MAPK12, PIK3R1, S100B, SOS1, TP53* | 9.66 x 10^-18^ | *ADIPOQ, AKT3, APC, APOD, APP, ARFIP2, ARPC2, ATP6V0D1, BCCIP, BDNF, CALB1, CDC42, CDC42EP2, CDH3, CDK5R1, CITED2, COL4A3BP, CPE, CTNNB1, DCN, DNM2, DSE, E2F8, EDA, EDNRA, EHF, ENPP2, FLOT1, FYN, FZD1, FZD3, GATA3, GJA1, GLI2, GNAS, GSK3B, GSN, HAS2, HEYL, HHIP, IGF1R, IL6, KCNJ2, KCNMA1, KCNQ3, KIT, LYVE1, MAFF, MAP2K4, MAP2K6, MAPK11, MAPK12, MEF2A, MEF2C, MYH10, MYO10, NFIB, PADI1, PFN2, PIK3CA, PIK3R2, PLA2G2A, PLA2G5, PLD1, PPARG, PRKACA, PSEN2, PTCH2, RAC2, RBPJ, RDH10, S1PR1, SCN1B, SDC2, SEMA3C, SLC9A3R1, SNAP25, SOS1, STAT5B, STK4, STMN1, STMN3, SUFU, TCF7L2, TDRD7, TFDP1, TGM2, TP53, TRAF6, WASF2, WNT11, WNT2, WNT7A* |
| *CHUK* | Kinase | *EFNA1, GJA1, GNAS, HAS2, IL5, IL6, KITLG, MAP2K6, MEF2C, PLA2G5, TAGLN, TGM2, TNF, TP53* | 1.1 x 10^-17^ | *ADIPOQ, ADM, APP, AXIN2, BCCIP, BDNF, BMP4, CALB1, CDC42EP2, CDH3, CITED2, CLTC, COL4A1, COL4A3BP, CSK, CTSB, DCN, DVL2, EFNA1, EHF, ENPP2, ERCC3, FGF2, FYN, FZD1, FZD3, GATA3, GJA1, GNAS, GSN, HAS2, HEY1, HEYL, HHIP, ID2, IL5, IL6, KCNJ2, KIT, KRT17, LGR4, LYVE1, MEF2C, PIK3CA, PIK3R1, PIK3R3, PLA2G2A, PLD1, PRKACA, PRKCA, PTCH1, PTCH2, RBPJ, RDH10, S1PR1, SDC2, SEMA3C, SNX10, STAT5A, STMN1, TAGLN, TDRD7, TFDP1, TGFB1I1, TGM2, TNF, TP53, TRAF6, WNT11* |
| *EDA* | Cytokine | *APP, BDNF, CDK5R1, CLTC, CTNNB1, DPYSL2, ENPP2, GJA1, HAND1, PAFAH1B2, PIK3R1, PPARG, PSEN2, RBPJ, S100B, SNAP25, STMN1, TNF, TP53, UCHL1* | 1.12 x 10^-17^ | *AGPS, AIMP2, AKT3, APC, APOD, APP, AQP1, ARF1, ARFIP2, ATP6V0D1, BCCIP, BDNF, BMP4, CALB1, CAPZB, CAV3, CDC42, CDC42EP2, CDH3, CDK5R1, CLTC, CPE, CSK, CTNNB1, CTSB, DCN, DNM2, DPYSL2, DVL2, EFNA1, EHF, ENPP2, EXT1, EXT2, FCER1A, FGF2, FOXP1, FRZB, FST, FZD3, GAP43, GJA1, GNA11, GNAS, GSK3B, HAND1, HAS2, HHIP, IGF1R, IL6, ITIH4, KCNB2, KCNJ2, KCNMB1, KCNQ3, KCNV1, KIF4A, KIT, KITLG, KRT17, KRT5, LMO4, LYVE1, MAFF, MAPK10, MAPK3, MAPK9, MEF2A, MYO10, NFIB, NME2, PADI1, PAFAH1B1, PIK3CA, PIK3R1, PLA2G12A, PLA2G3, PLA2G5, PLA2G6, PLCG1, PLCG2, PLD1, PRKACA, PRKCB, PTCH1, PTCH2, RAC1, RAF1, RBPJ, S100B, SEMA3C, SLC9A3R1, SNX10, SOS1, STAT5A, STK4, STMN1, SUFU, TDRD7, TFDP1, TGFB1I1, TGM2, TTC30B, TTLL1, UCHL1, VAV1, WASF2, WNT16, WNT8B* |
| Misoprostol | Chemical drug | *BDNF, CDK5R1, CTNNB1, FGF2, GJA1, GSK3B, HAS2, IGF1R, IL6, MAP2K4, MEF2A, MEF2C, PIK3R1, PLA2G2A, PPARG, S1PR1, SEMA3C, SOS1, TAGLN, TNF, TP53, UPK2, WNT7A* | 1.22 x 10^-17^ | *ACADVL, AIF1, APP, AQP1, ARF1, ARPC2, CAPRIN1, CCL11, CDC42, CDC42EP2, CDK5R1, CITED2, DCN, DPYSL2, DSE, E2F8, EDA, EDNRA, ELF5, ENPP2, F11R, FGF2, FRZB, FST, GAP43, GATA3, GJA1, GSN, HAS2, HEY1, HEYL, ID2, IFT27, IGF1R, IL5, IL6, INPP5D, KCNMB1, KCNQ3, KIF4A, MAFF, MAP2K4, MAP2K7, MAPK12, MAPK9, MEF2A, MEF2C, MYH10, MYO10, NFIB, PADI1, PFN2, PIK3CA, PIK3R1, PIK3R3, PLA2G10, PLA2G2F, PLA2G5, PLA2G6, PLCG1, PLPP3, PRKACA, RAC2, RBPJ, RDH10, S1PR1, SEMA3C, SLC9A3R1, SMARCA4, SNAP25, SNX10, STAT5A, STAT5B, STK4, STMN3, SUFU, TAGLN, TCF7L2, TDRD7, TFAP2A, TFDP1, TNF, TNNC1, TP53, TRAF6, UPK1A, UPK1B, UPK2, WNT16* |
| *MAP3K11* | Kinase | *APC, FZD1, FZD6, PTCH1, SUFU, TNF* | 1.79 x 10^-17^ | *AKT3, APOD, APP, AQP1, ARPC2, AXIN2, BDNF, BMP4, CAV3, CCL11, CDK5R1, CITED2, COL4A1, CTSB, DCN, DSE, EFNA1, ENPP2, FGF2, FRZB, FST, FYN, FZD3, GLI2, GNAS, GSK3B, GSN, HAS2, HHIP, ID2, IGF1R, IL6, KITLG, KRT17, MAFF, MAP2K4, MAP2K6, MAPK3, MAPK9, MEF2C, PLD1, PTCH1, PTCH2, RAC1, SDC2, SEMA3C, SNX10, STK4, STMN1, TAGLN, TDRD7, TGM2, TNNC1, WNT11, WNT16, WNT7A* |
| *LGR4* | Trans-membrane receptor | *ADIPOQ, ADM, APOD, AQP1, BDNF, CITED2, EDNRA, EFNA1, ENPP2, FST, GAP43, GJA1, HEY1, IL5, IL6, KRT5, MYO10, NME2, NPY1R, PIK3R1, PLPP3, STAT5A, STAT5B, TNF, TP53* | 1.96 x 10^-17^ | *APC, APP, AXIN2, BMP4, CITED2, CTNNB1, EXT2, FRZB, FST, GATA3, GJA1, GLI2, GSK3B, HAND1, HEY1, ID2, IL6, IRF6, KITLG, KRT17, MEF2C, PIK3R1, PPARG, RBPJ, TBX6, TNF, WNT7A, WNT8B* |
| *Map3k7* | Kinase | *ADIPOQ, ADM, APP, COL4A1, CTNNB1, CTSH, EFNA1, ENPP2, GHRL, ID2, IGF1R, IL6, ITIH3, KRT17, NFIB, PLA2G2A, PPARG, TNF, TP53* | 2.02 x 10^-17^ | *ADIPOQ, AKT3, APOD, AQP1, ARPC2, AXIN2, BCCIP, BDNF, BMP4, CALB1, CDC42, CDH3, CDK5R1, CITED2, COL4A1, COL4A3BP, CSK, CTNNB1, CTSB, DCN, EFNA1, EHF, ENPP2, FGF2, FRZB, FST, FZD1, GAP43, GLI2, GNAS, GSK3B, HAS2, INPP5D, ITIH5, KIT, KRT17, LMO4, MAFF, NFIB, PIK3R1, PLCG2, PLD1, PRKACA, PTCH1, RAF1, RBPJ, S1PR1, SDC2, SEMA3C, SNX10, STAT5A, STMN1, TAGLN, TDRD7, TFDP1, TGFB1I1, TNF, TNNC1, WNT11, WNT16, WNT2* |
| Sorbitol | Chemical - endogenous mammalian | *ADIPOQ, ADM, APOD, APP, AQP1, AXIN2, BDNF, CALB1, COL4A1, CPE, CTNNB1, CTSB, DCN, EDA, EDNRA, EFNA1, EHF, FGF2, FST, GAP43, GATA3, GHRL, GJA1, ID2, IGF1R, IL5, IL6, KIF4A, KIT, KRT17, LMO4, LYVE1, MAPK3, PAFAH1B1, PIK3R1, PLD1, PPARG, PRKCA, PRKCB, SLC35D1, STAT5A, SULT1B1, TAGLN, TGM2, TNF, TP53, ZNF750* | 2.06 x 10^-17^ | *ACADVL, ADM, AIF1, AKT3, APC, APOD, APP, AQP1, ARPC2, AXIN2, BCCIP, CALB1, CCL11, CDH3, CDK5R1, CITED2, CSK, CTSH, DCN, DPYSL2, DSE, EFNA1, EHF, FRZB, FST, FYN, FZD1, FZD3, GAP43, GJA1, GLI2, GNAS, GSN, HAS2, ID2, ITIH5, KCNMA1, KCNQ3, KIT, KITLG, MAFF, MAP2K4, MAP2K6, MAP2K7, MAPK12, MAPK9, MEF2A, MYH10, NFIB, PAFAH1B1, PAFAH1B2, PDE2A, PIK3CA, PIK3R1, PLA2G5, PLCG1, PLD1, PRKCA, PSEN2, PTCH1, PTCH2, RAC1, RAF1, RBPJ, RDH10, S100B, S1PR1, SDC2, SEMA3C, SNAP25, ST14, STK4, TCF7L2, TDRD7, TGFB1I1, TNNC1, TRAF6, WNT11, WNT16, WNT2, WNT7A* |
| Idoxifene | Chemical drug | *ADIPOQ, BMP4, CTSB, CTSH, EDNRA, EHF, GPM6A, HEY1, HHIP, IL6, IRF6, KCNJ2, KCNQ3, KIT, MAFF, MEF2C, PLPP3, PPARG, PTCH1, RAC2, SDC2, SMARCA4, TAGLN, TP53, WNT7A* | 2.28 x 10^-17^ | *APC, APOD, AQP1, ARFIP2, ATP6V0D1, AXIN2, BCCIP, BDNF, BMP4, CALB1, CDC42, CDC42EP1, CDC42EP2, CDH3, CHN1, COL4A3BP, CPE, CTNNB1, CTSH, DNM2, DSE, DVL2, E2F8, EDA, EDNRA, EFNA1, EHF, ENPP2, ERCC3, FCER1A, FLOT1, FOXP1, FST, GHRL, GJA1, GNAS, GSK3B, GSN, IGF1R, IRF6, ITIH4, KCNJ2, KCNMA1, KRT5, LMO4, MAP2K4, MAP2K6, MAP2K7, MAPK11, MAPK12, MAPK3, MEF2A, MEF2C, MYH10, NME2, PADI1, PFN2, PLA2G12A, PLA2G2A, PLA2G5, PLA2G6, PLPP3, PRKCA, PTCH1, PTCH2, RBPJ, SCN1B, SDC2, SEMA3C, SLC9A3R1, SNAP25, SOS1, STAT5A, STAT5B, STK4, SUFU, TAGLN, TCF7L2, TDRD7, TFAP2A, TFDP1, TGFB1I1, TGM2, TNF, TNNC1, TP53, WASF2* |
| *RPL23A* | Other | *ADIPOQ, AQP1, BMP4, CAV3, CCL11, CDK5R1, EFNA1, EHF, EMP2, GJA1, IL5, IL6, MAP2K6, MAPK12, PPARG, TAGLN, TGM2, TNF, TP53* | 2.64 x 10^-17^ | *ACADVL, AIF1, AKT3, APP, AXIN2, CAPRIN1, CAPZB, CDC42, CDC42EP1, CDC42EP2, CDH3, CDK5R1, CHN1, CLTC, COL4A1, COL4A3BP, CTNNB1, CTSB, CTSH, DCN, DPYSL2, DVL2, EDA, EDNRA, EFNA1, EHF, ELF5, ENPP2, ERCC3, F11R, FCER1A, FGF2, FYN, FZD1, FZD3, GATA5, GSK3B, GSN, HAND1, HAS2, HHIP, ID2, IFT27, IGF1R, IL5, INPP5D, IRF6, ITIH3, KCNJ2, KCNMA1, KCNMB1, KCNQ3, KRT17, KRT5, LYVE1, MAP2K4, MAP2K6, MAP2K7, MAPK11, MAPK12, MAPK3, MEF2A, MEF2C, MYH10, NFIB, PADI1, PAFAH1B2, PDE2A, PIK3CA, PIK3R2, PIK3R3, PLA2G5, PRKCA, PSEN2, PTCH1, PTCH2, RAC1, RAC2, RAF1, RBPJ, RDH10, SEMA3C, SMARCA4, ST14, STAT5B, STMN1, STMN3, TCF7L2, TFAP2A, TFDP1, TGFB1I1, TGM2, TMEM100, TMEM17, TNF, TNNC1, TP53, TRAF6, UCHL1, WNT2, WNT2B, WNT7A* |
| *PIN1* | Enzyme | *APP, BDNF, DNM2, FCER1A, FGF2, FST, HHIP, IGF1R, IL6, NPY1R, PPARG, PTCH1, TNF* | 2.69 x 10^-17^ | *ACADVL, ADM, AIF1, AKT3, CALB1, CAV3, CCL11, CDC42, CDC42EP1, CDH3, CDK5R1, CHN1, CITED2, CTNNB1, CTSB, CTSH, EFNA1, EHF, ENPP2, F11R, FGF2, FRZB, FZD1, GATA3, GLI2, GSN, HAS2, HEY1, ID2, IFT27, IL6, INPP5D, KCNJ2, KCNMA1, KIT, KITLG, KRT17, LGR4, MAP2K6, MAP2K7, MAPK11, MAPK3, MYH10, PAFAH1B2, PDE2A, PIK3CA, PIK3R3, PLA2G2A, PLCG2, PLD1, PRKCA, PSEN2, PTCH1, PTCH2, RAC1, RAC2, RBPJ, S1PR1, SLC9A3R1, ST14, STAT5A, SUFU, TDRD7, TFDP1, TGFB1I1, TGM2, TNNC1, UPK1A, UPK1B, UPK2, WNT16, WNT2, WNT7A* |
| *AMPK*  *(PRKAA,*  *PRKAA1,*  *PRKAA2,*  *PRKAB,*  *PRKAB1,*  *PRKAB2,*  *PRKAG,*  *PRKAG1,*  *PRKAG2)* | Complex | *AIF1, APP, BDNF, CDK5R1, CLTC, DPYSL2, FYN, IL6, MAPK11, MAPK3, PAFAH1B2, S100B, SNAP25, STMN1, TNF, UCHL1* | 2.87 x 10^-17^ | *ACADVL, ADIPOQ, ADM, AGPS, AIF1, AKT3, ANXA7, APOD, APP, ARF1, ARPC2, BMP4, CDC42EP1, CDH3, CDK5R1, CITED2, CLTC, COL4A3BP, CTNNB1, CTSB, CTSH, DPYSL2, DSE, DVL2, EDNRA, ELF5, ENPP2, FST, FYN, FZD10, FZD3, FZD9, GATA5, GLI2, GNAS, GSK3B, GSN, HHIP, IL6, IRF6, ITIH3, ITIH4, ITIH5, KCNMA1, KCNMB1, KITLG, KRT17, KRT71, KYAT3, LGR4, LIN7C, LMO4, MAFF, MAP2K4, MAP2K6, MAP2K7, MAPK11, MAPK9, MYO10, NCS1, NFIB, NPY1R, PAFAH1B2, PDE2A, PFN2, PHACTR4, PIK3CA, PLA2G10, PLA2G12A, PLA2G5, PLCG2, PLD1, PLPP3, PRKCA, PSEN2, PTCH1, PTCH2, PTPDC1, RAF1, RBPJ, S100B, S1PR1, SDC2, SLC35D1, SMARCA4, SNAP25, ST14, STAT5A, STK4, STMN1, TAGLN, TBX6, TDRD7, TFDP1, TGFB1I1, TMEM100, TMEM17, TNF, TRAF6, TTC30B, TTLL1, UCHL1, UPK1A, UPK1B, UPK2, VAV2, WNT16, WNT2, WNT2B, WNT7A* |
| *IKBKG* | Kinase | *AQP1, CALB1, CDC42, COL4A1, DPP6, FYN, GATA3, GATA5, GSN, IL6, INPP5D, KIT, PLCG1, PPARG, PRKACA, PRKCB, RAF1, STAT5B, TGM2, TNF, TP53* | 2.9 x 10^-17^ | *ADM, AKT3, APP, BCCIP, BDNF, BMP4, CALB1, CDH3, CDK5R1, CITED2, COL4A3BP, CSK, CTSB, DCN, DVL2, EFNA1, EHF, ENPP2, FRZB, FST, FYN, FZD1, GAP43, GATA3, GJA1, GLI2, GNAS, GSK3B, HAS2, HEY1, ID2, IL5, IL6, KIT, KRT17, MAFF, MAPK9, MEF2C, PIK3R1, PLA2G2A, PLCG2, PLD1, PPARG, PRKACA, RAC1, RBPJ, SDC2, SEMA3C, SNX10, STAT5A, STMN1, TDRD7, TFDP1, TGFB1I1, TGM2, TNF, TNNC1, TRAF6, WNT11, WNT16* |
| *NT5C1A* | Phosphatase | *ADIPOQ, COL4A1, CTSH, ENPP2, IL5, IL6, PPARG, PRKCSH, RAC1, TNF* | 3.02 x 10^-17^ | *ACADVL, ADIPOQ, ADM, AIF1, AKT3, APOD, APP, ARF1, ARPC2, BMP4, CDC42EP1, CDH3, CDK5R1, CITED2, CLTC, COL4A3BP, CTNNB1, CTSB, CTSH, DPYSL2, DSE, DVL2, EDNRA, ELF5, ENPP2, FST, FYN, FZD3, GLI2, GNAS, GSK3B, GSN, HHIP, IL6, IRF6, ITIH3, ITIH4, ITIH5, KCNMA1, KCNMB1, KITLG, KRT17, KYAT3, LGR4, LMO4, MAFF, MAP2K4, MAP2K6, MAP2K7, MAPK11, MAPK9, MYO10, NFIB, PAFAH1B2, PDE2A, PFN2, PIK3CA, PLA2G5, PLD1, PPARG, PRKCA, PSEN2, PTCH1, PTCH2, RAF1, RBPJ, S1PR1, SDC2, SMARCA4, SNAP25, ST14, STAT5A, STK4, STMN1, TAGLN, TDRD7, TFDP1, TGFB1I1, TMEM17, TNF, TRAF6, UCHL1, WNT16, WNT2, WNT7A* |
| *UCHL3* | Peptidase | *ADM, APP, BDNF, CDC42, CDK5R1, CPE, CTNNB1, CTSB, FGF2, FOXP1, FST, FZD1, GAP43, GATA3, GJA1, HAS2, ID2, IL5, IL6, KIT, KRT71, LYVE1, MAPK9, NME2, PLD1, PPARG, PRKACA, PRKCA, PRKCB, RBPJ, SNAP25, STAT5B, TFDP1, TNF, TP53* | 3.02 x 10^-17^ | *ACADVL, ADIPOQ, ADM, AIF1, AKT3, APOD, APP, ARF1, ARPC2, BMP4, CDC42EP1, CDH3, CDK5R1, CITED2, CLTC, COL4A3BP, CTNNB1, CTSB, CTSH, DPYSL2, DSE, DVL2, EDNRA, ELF5, ENPP2, FST, FYN, FZD3, GLI2, GNAS, GSK3B, GSN, HHIP, IL6, IRF6, ITIH3, ITIH4, ITIH5, KCNMA1, KCNMB1, KITLG, KRT17, KYAT3, LGR4, LMO4, MAFF, MAP2K4, MAP2K6, MAP2K7, MAPK11, MAPK9, MYO10, NFIB, PAFAH1B2, PDE2A, PFN2, PIK3CA, PLA2G5, PLD1, PRKCA, PSEN2, PTCH1, PTCH2, RAF1, RBPJ, S1PR1, SDC2, SMARCA4, SNAP25, ST14, STAT5A, STK4, STMN1, TAGLN, TDRD7, TFDP1, TGFB1I1, TMEM17, TNF, TP53, TRAF6, UCHL1, WNT16, WNT2, WNT7A* |
| Warfarin | Chemical drug | *AXIN2, CTNNB1, FST, GATA3, GLI2, HAS2, HEY1, ID2, IL5, IL6, INPP5D, RAC1, S1PR1, TAGLN, TNF* | 3.24 x 10^-17^ | *AIF1, AKT3, APOD, AQP1, ARF1, ARPC2, BCCIP, CALB1, CCL11, CDC42, CDC42EP2, CDH3, CDK5R1, CSK, CTSH, DCN, DSE, EFNA1, EHF, ENPP2, ERCC3, FGF2, FRZB, FST, FZD3, GAP43, GATA3, GJA1, GSK3B, GSN, HAS2, HEY1, HHIP, ID2, IFT27, IGF1R, IL5, IL6, IRF6, KCNJ2, KCNMB1, KIF4A, KITLG, KRT5, LYVE1, MAFF, MAP2K4, MEF2C, NCS1, NFIB, PFN2, PIK3CA, PIK3R1, PIK3R2, PLA2G5, PLCG1, PLD1, PRKCA, PTCH2, RAC2, RBPJ, RDH10, S1PR1, SEMA3C, SMARCA4, SNX10, STAT5A, STAT5B, SUFU, TAGLN, TCF7L2, TFDP1, TGFB1I1, TGM2, TNF, TP53, TRAF6, UPK1A, UPK1B, UPK2, VAV1, VAV2, WNT11* |
| Acetate | Chemical - endogenous mammalian | *APP, BDNF, CTSB, DCN, FST, GJA1, IGF1R, IL5, IL6, KIT, KITLG, MAP2K6, PLA2G2A, PLD1, PPARG, TGM2, TNF, TP53* | 3.3 x 10^-17^ | *ACADVL, ADIPOQ, ADM, AIF1, AKT3, APOD, APP, ARF1, ARPC2, BMP4, CDC42EP1, CDH3, CDK5R1, CITED2, CLTC, COL4A3BP, CTNNB1, CTSB, CTSH, DPYSL2, DSE, DVL2, EDNRA, ELF5, ENPP2, FST, FYN, FZD3, GLI2, GNAS, GSK3B, GSN, HHIP, IL6, IRF6, ITIH3, ITIH4, ITIH5, KCNMA1, KCNMB1, KITLG, KRT17, KYAT3, LGR4, LMO4, MAFF, MAP2K4, MAP2K6, MAP2K7, MAPK11, MAPK9, MYO10, NFIB, PAFAH1B2, PDE2A, PFN2, PIK3CA, PLA2G5, PLD1, PRKCA, PSEN2, PTCH1, PTCH2, RAF1, RBPJ, S1PR1, SDC2, SMARCA4, SNAP25, ST14, STAT5A, STK4, STMN1, TAGLN, TDRD7, TFDP1, TGFB1I1, TMEM17, TNF, TP53, TRAF6, UCHL1, WNT16, WNT2, WNT7A* |
| Octanoic acid | Chemical - endogenous mammalian | *APP, FST, GJA1, ID2, IGF1R, IL6, PLPP3, SNAP25, TP53* | 3.38 x 10^-17^ | *ACADVL, ADIPOQ, ADM, AIF1, AKT3, APOD, APP, ARF1, ARPC2, BMP4, CDC42EP1, CDH3, CDK5R1, CITED2, CLTC, COL4A3BP, CTNNB1, CTSB, CTSH, DPYSL2, DSE, DVL2, EDNRA, ELF5, ENPP2, FST, FYN, FZD3, GLI2, GNAS, GSK3B, GSN, HHIP, IL6, IRF6, ITIH3, ITIH4, ITIH5, KCNMA1, KCNMB1, KITLG, KRT17, KYAT3, LGR4, LMO4, MAFF, MAP2K4, MAP2K6, MAP2K7, MAPK11, MAPK9, MYO10, NFIB, PAFAH1B2, PDE2A, PFN2, PIK3CA, PLA2G5, PLD1, PPARG, PRKCA, PSEN2, PTCH1, PTCH2, RAF1, RBPJ, S1PR1, SDC2, SMARCA4, SNAP25, ST14, STAT5A, STK4, STMN1, TAGLN, TDRD7, TFDP1, TGFB1I1, TMEM17, TNF, TRAF6, UCHL1, WNT16, WNT2, WNT7A* |
| *PELP1* | Other | *ADM, APC, CDC42, CITED2, COL4A1, CTNNB1, DNM2, FST, IL6, MAFF, PIK3CA, PIK3R3, RAC2, SUFU, TAGLN, TP53, UPK1A, UPK1B, UPK2* | 3.62 x 10^-17^ | *ADM, AKT3, APC, APOD, AQP1, ARF1, ARFIP2, ARPC2, ATP6V0D1, BCCIP, BMP4, CALB1, CDC42, CDC42EP2, CDH3, COL4A1, COL4A3BP, CPE, CTNNB1, CTSH, DNM2, DVL2, E2F8, EDA, EDNRA, EFNA1, EHF, ENPP2, ERCC3, F11R, FCER1A, FLOT1, FOXP1, FRZB, FST, FYN, GJA1, GNA11, GNAS, GSK3B, GSN, HEY1, IGF1R, INPP5D, IRF6, KCNJ2, KCNMA1, KCNQ3, KITLG, KRT5, MAP2K4, MAP2K6, MAP2K7, MAPK11, MAPK12, MEF2A, MEF2C, MYH10, NFIB, PADI1, PAFAH1B2, PDE2A, PFN2, PLA2G12A, PLA2G2A, PLA2G5, PLA2G6, PLCG1, PLPP3, PRKCA, PSEN2, PTCH1, PTCH2, RBPJ, RDH10, SCN1B, SDC2, SEMA3C, SLC9A3R1, SNAP25, SOS1, ST14, STAT5A, STAT5B, STK4, SUFU, TAGLN, TCF7L2, TFDP1, TGFB1I1, TGM2, TNF, TNNC1, TP53, WASF2, WNT16, WNT2, WNT7A* |
| *HBP1* | Transcription regulator | *FGF2, FST, IGF1R, IL6, KIT, S1PR1, TNF, TP53* | 3.73 x 10^-17^ | *ACADVL, AKT3, APC, APP, ARF1, ARFIP2, ATP6V0D1, AXIN2, BDNF, BMP4, CALB1, CAV3, CCL11, CDC42EP1, CDC42EP2, CDH3, CHN1, COL4A1, CPE, CTSB, CTSH, DNM2, DPYSL2, DSE, EDA, EDNRA, EFNA1, EHF, ELF5, ENPP2, FCER1A, FLOT1, FOXP1, FRZB, FST, FYN, FZD3, FZD6, GAP43, GATA3, GATA5, GJA1, GNA11, GNAS, GSK3B, GSN, HAS2, HHIP, ID2, IGF1R, INPP5D, ITIH3, ITIH4, ITIH5, KCNB2, KCNMA1, KCNQ3, KIF4A, KIT, KRT17, KRT5, LGR4, LYVE1, MAP2K6, MAP2K7, MAPK10, MAPK12, MAPK3, MAPK9, MEF2A, MYH10, NFIB, PADI1, PAFAH1B1, PAFAH1B2, PDE2A, PIK3CA, PIK3R2, PIK3R3, PLA2G12A, PLA2G3, PLA2G6, PLCG2, PLD1, PPARG, PRKACA, PRKCA, PRKCB, PSEN2, PTCH1, PTCH2, RAC2, RBPJ, S100B, SCN1B, SLC9A3R1, SNX10, SOS1, ST14, STAT5A, STMN1, STMN3, SUFU, TCF7L2, TFDP1, TGFB1I1, TGM2, TMEM100, TMEM17, UCHL1, VAV1, WASF2, WNT2, WNT2B, WNT7A, ZNF750* |
| Az-960 | Chemical reagent | *ADIPOQ, ADM, APOD, AXIN2, CAV3, CTNNB1, EHF, IL5, IL6, KCNIP3, KITLG, PPARG, TNF* | 4.27 x 10^-17^ | *AIF1, AIMP2, AKT3, APC, AQP1, ARF1, ARPC2, AXIN2, BCCIP, BMP4, CALB1, CCL11, CDC42, CDC42EP1, CDC42EP2, CDK5R1, CHN1, CSK, CTSH, DCN, DSE, DVL2, E2F8, EDA, EHF, ENPP2, FGF2, FRZB, FST, FZD3, GAP43, GJA1, GSN, HAS2, HEY1, HEYL, HHIP, IFT27, IGF1R, IL5, IL6, IRF6, ITIH5, KCNJ2, KCNMB1, KCNQ3, KIF4A, KIT, LGR4, MAFF, MAP2K4, MAP2K6, MEF2A, MYH10, MYO10, NFIB, PADI1, PIK3CA, PIK3R3, PLA2G5, PLA2G6, PLCG1, PLD1, PRKACA, PRKCA, PTCH2, RAC2, RBPJ, RDH10, S100B, S1PR1, SEMA3C, SMARCA4, SNAP25, SNX10, SOS1, STAT5B, STMN3, SUFU, TAGLN, TDRD7, TFAP2A, TFDP1, TGFB1I1, TP53, TRAF6, UPK1A, UPK1B, UPK2, WNT11, WNT16* |
| *ULK1* | Kinase | *CCL11, CITED2, IL5, IL6, LMO4, STAT5A, TNF, TP53* | 4.61 x 10^-17^ | *ACADVL, ADM, AKT3, APOD, AQP1, ARPC2, AXIN2, BDNF, BMP4, CALB1, CAV3, CCL11, CDC42, CDC42EP2, CDH3, CDK5R1, COL4A1, CTSH, DCN, E2F8, EFNA1, EHF, ENPP2, FGF2, FRZB, FST, GAP43, GLI2, GNAS, GSN, HEYL, ID2, IL5, IL6, KCNMA1, KIT, KRT17, MAFF, MAP2K4, MAP2K6, MAP2K7, MAPK12, MAPK3, MEF2C, MYH10, MYO10, NFIB, PAFAH1B2, PDE2A, PFN2, PLA2G5, PLD1, PRKACA, PRKCA, PSEN2, PTCH1, RBPJ, RDH10, S1PR1, SDC2, SEMA3C, SNX10, ST14, STAT5A, STMN3, TAGLN, TCF7L2, TGFB1I1, TGM2, TNF, TNNC1, WNT11, WNT16, WNT2, WNT7A* |
| *PHB2* | Transcription regulator | *CDC42EP1, DCN, DVL2, FGF2, FST, GJA1, GSK3B, HAS2, ID2, IGF1R, IL6, KRT5, MAPK11, MAPK3, PLPP3, PPARG, RAC1, SNAP25, TFAP2A, TFDP1, TGM2, TP53* | 4.88 x 10^-17^ | *ADM, AKT3, APC, APOD, AQP1, ARF1, ARFIP2, ARPC2, ATP6V0D1, BCCIP, BMP4, CALB1, CDC42, CDC42EP2, CDH3, COL4A1, COL4A3BP, CPE, CTNNB1, CTSH, DNM2, DVL2, E2F8, EDA, EDNRA, EFNA1, EHF, ENPP2, ERCC3, F11R, FCER1A, FLOT1, FOXP1, FRZB, FST, FYN, GJA1, GNA11, GNAS, GSK3B, GSN, HEY1, IGF1R, INPP5D, IRF6, KCNJ2, KCNMA1, KCNQ3, KITLG, KRT5, MAP2K4, MAP2K6, MAP2K7, MAPK11, MAPK12, MEF2A, MEF2C, MYH10, NFIB, PADI1, PAFAH1B2, PDE2A, PFN2, PLA2G12A, PLA2G2A, PLA2G5, PLA2G6, PLCG1, PLPP3, PRKCA, PSEN2, PTCH1, PTCH2, RBPJ, RDH10, SCN1B, SDC2, SEMA3C, SLC9A3R1, SNAP25, SOS1, ST14, STAT5A, STAT5B, STK4, SUFU, TCF7L2, TFDP1, TGFB1I1, TGM2, TNF, TNNC1, TP53, WASF2, WNT16, WNT2, WNT7A* |
| *WNT5A* | Cytokine | *ADM, APP, BDNF, ID2, TNF, TP53* | 5.26 x 10^-17^ | *ADIPOQ, ADM, APOD, APP, AQP1, ARPC2, AXIN2, BDNF, BMP4, CALB1, CAV3, CCL11, CDK5R1, CITED2, COL4A1, CTSB, EFNA1, EHF, ENPP2, FST, FZD3, GLI2, GNAS, GSK3B, GSN, HAS2, HEY1, HHIP, ID2, IGF1R, IL6, IRF6, KCNJ2, KIT, KITLG, KRT17, LMO4, MAFF, MAP2K6, MAPK3, MEF2C, PLA2G2A, PLA2G6, PLD1, PRKCA, PTCH1, PTCH2, RAC1, RBPJ, RDH10, SDC2, TAGLN, TGM2, TNF, TNNC1, WNT11* |
| *SOX2* | Transcription regulator | *BDNF, CDK5R1, DNM2, FCER1A, FST, GAP43, HHIP, IGF1R, IL5, IL6, KIT, KITLG, PTCH1, S100B, SLC9A3R1, SNAP25, TNF* | 5.37 x 10^-17^ | *ACADVL, AKT3, APC, APP, AXIN2, BMP4, CDH3, CITED2, COL4A1, CTNNB1, CTSB, CTSH, EFNA1, ENPP2, EXT2, FRZB, FST, FYN, GATA3, GJA1, GLI2, GSK3B, GSN, HAND1, HAS2, HEY1, ID2, IGF1R, IL5, IL6, IRF6, KCNMA1, KITLG, KRT17, MAP2K6, MAP2K7, MAPK12, MEF2C, MYH10, PAFAH1B2, PDE2A, PIK3R1, PPARG, PRKCA, PSEN2, PTCH1, RBPJ, ST14, STMN1, TBX6, TCF7L2, TFDP1, TGFB1I1, TGM2, TNF, WNT2, WNT7A, WNT8B* |
| 2-aminopurine | Chemical reagent | *COL4A1, CPE, CSK, CTSH, GSN, HAND1, IL6, KIT, KITLG, PPARG, PSEN2, TAGLN* | 5.85 x 10^-17^ | *ACADVL, ADIPOQ, ADM, AIF1, AIMP2, AKT3, APC, APP, ARF1, ARFIP2, ARPC2, ATP6V0D1, AXIN2, BDNF, CALB1, CAV3, CCL11, CDC42EP1, CDH3, COL4A1, CPE, CSK, CTSB, CTSH, DNM2, DPYSL2, DSE, EDA, EFNA1, EHF, ELF5, EMP2, ENPP2, EXT2, FCER1A, FST, FYN, FZD3, GAP43, GATA5, GLI2, GNA11, GNAS, GSK3B, GSN, HAS2, HHIP, ID2, IL6, ITIH3, ITIH4, ITIH5, KCNB2, KCNJ2, KCNMA1, KIT, KRT17, KRT5, KYAT3, LGR4, LMO4, LYVE1, MAP2K4, MAP2K6, MAP2K7, MAPK10, MAPK12, MAPK9, MEF2A, MYH10, NME2, PAFAH1B1, PAFAH1B2, PDE2A, PIK3R1, PIK3R2, PLA2G10, PLA2G12A, PLA2G3, PLCG1, PLCG2, PLD1, PLPP3, PRKACA, PRKCA, PRKCB, PSEN2, PTCH1, PTCH2, RAC2, RAF1, RBPJ, RDH10, S100B, SEMA3C, SLC9A3R1, SMARCA4, SNX10, SOS1, ST14, STAT5A, STK4, STMN1, STMN3, SUFU, TBX6, TCF7L2, TFDP1, TGFB1I1, TGM2, TMEM100, TMEM17, TNF, TOLLIP, UCHL1, WASF2, WNT2, WNT2B, WNT7A, WNT8B* |
| Resiquimod | Chemical drug | *ACADVL, CCL11, COL4A1, GAP43, GSN, IL6, ITIH4, KRT71, MAP2K6, PFN2, PRKCB, RAC1, STAT5B, TNF, TP53* | 6.02 x 10^-17^ | *ADIPOQ, ADM, AIF1, AKT3, APOD, APP, AQP1, ARPC2, BCCIP, BDNF, BMP4, CALB1, CCL11, CDH3, CITED2, COL4A3BP, CSK, DCN, EFNA1, ENPP2, FRZB, FST, FYN, FZD1, GAP43, GATA3, GLI2, GNAS, GSK3B, HAS2, HEY1, ID2, IL5, IL6, INPP5D, KCNH1, KCNMA1, KIT, KRT17, MAFF, MAP2K6, MEF2C, PIK3R1, PLA2G5, PLD1, PTCH1, RAC1, RBPJ, RDH10, S1PR1, SDC2, SEMA3C, SNX10, STAT5A, STMN1, TFDP1, TGFB1I1, TGM2, TNF, TNNC1, TRAF6, VAV2, WNT11, WNT16* |
| At-9283 | Chemical drug | *BMP4, CCL11, GATA3, GLI2, HHIP, IL6, KRT17, MEF2C, PTCH1, TNF* | 6.07 x 10^-17^ | *ACADVL, ADM, AIMP2, AKT3, ANXA7, AQP1, ARF1, ARPC2, AXIN2, BCCIP, BMP4, CAPZB, CCL11, CDH3, CDK5R1, COL4A3BP, CPE, CSK, CTNNB1, CTSH, DOCK1, DVL2, EFNA1, ERCC3, FGF2, FST, FYN, FZD3, GAP43, GATA3, GJA1, GNAS, GSN, HAS2, HEY1, HHIP, ID2, IGF1R, IL5, IL6, ITIH5, KCNJ2, KCNMA1, KCNMB1, KIF4A, KIT, KITLG, MAFF, MAP2K4, MAP2K6, MAP2K7, MAPK11, MAPK12, MEF2A, MEF2C, MYH10, NME2, PAFAH1B2, PDE2A, PFN2, PIK3CA, PIK3R1, PIK3R3, PLA2G5, PSEN2, PTCH2, RAC2, RBPJ, RDH10, S1PR1, SMARCA4, SNX10, ST14, STAT5B, SUFU, TAGLN, TFAP2A, TFDP1, TGFB1I1, TNF, TNNC1, TP53, UPK1A, UPK1B, UPK2, WNT11, WNT16, WNT2, WNT7A* |
| Ep300/Pcaf  (*EP300,*  *KAT2B*) | Complex | *BCCIP, CCL11, CDH3, CSK, CTNNB1, CTSB, DCN, ENPP2, FGF2, HEY1, IL5, IL6, PIK3R1, RAC1, SEMA3C, TFDP1, TGFB1I1, TNF, TOLLIP, TP53* | 6.27 x 10^-17^ | *ACADVL, AIF1, AKT3, APC, APOD, APP, ARF1, ARFIP2, ARPC2, ATP6V0D1, AXIN2, BCCIP, CALB1, CAPRIN1, CAPZB, CDC42EP2, CDH3, CLTC, COL4A1, COL4A3BP, CPE, CTNNB1, CTSB, CTSH, DCN, DNM2, DPYSL2, DVL2, E2F8, EDA, EDNRA, EFNA1, ELF5, ENPP2, ERCC3, FYN, FZD1, FZD3, GATA5, GNA11, GSK3B, GSN, HAND1, HAS2, HHIP, ID2, IGF1R, IL5, IL6, KCNB2, KCNJ2, KCNMA1, KCNMB1, KCNQ3, LYVE1, MAP2K4, MAP2K6, MAP2K7, MAPK10, MAPK12, MAPK9, MEF2A, MYH10, NFIB, PADI1, PAFAH1B1, PAFAH1B2, PDE2A, PIK3CA, PIK3R2, PIK3R3, PLA2G5, PLD1, PPARG, PRKACA, PRKCA, PRKCB, PSEN2, PTCH1, PTCH2, RAC1, RAF1, RBPJ, RDH10, SEMA3C, SLC9A3R1, SMARCA4, SOS1, ST14, STAT5A, STAT5B, STK4, STMN1, SUFU, TCF7L2, TDRD7, TFAP2A, TFDP1, TGFB1I1, TGM2, TMEM100, TNF, TNNC1, TP53, WASF2, WNT2, WNT2B, WNT7A* |
| *SLC6A4* | Transporter | *AXIN2, BMP4, CTNNB1, ENPP2, IGF1R, KIT, KRT5, PPARG, SEMA3C, STAT5A, TP53* | 6.34 x 10^-17^ | *ACADVL, AIF1, AKT3, APOD, AQP1, ARF1, AXIN2, BCCIP, BDNF, CCL11, CDC42, CDH3, CLTC, CSK, CTSH, EFNA1, FOXP1, FRZB, FST, FZD3, GAP43, GATA3, GHRL, GJA1, GNAS, GSN, HAND1, HAS2, HEY1, HHIP, ID2, IFT27, IGF1R, IL5, IL6, IRF6, KCNJ2, KCNMB1, KIF4A, KRT17, KRT5, LMO4, LYVE1, MAP2K4, MAP2K6, MAPK3, NFIB, PFN2, PIK3CA, PIK3R1, PIK3R3, PLA2G2A, PLA2G5, PLCG1, PLD1, PRKACA, PRKCA, PTCH2, RAC2, RBPJ, S1PR1, SMARCA4, SNX10, SUFU, TAGLN, TCF7L2, TDRD7, TFAP2A, TFDP1, TGFB1I1, TNF, TP53, UPK1A, UPK1B, UPK2, WNT11, WNT16* |
| Fonda-parinux | Chemical drug | *CCL11, CDC42, CDC42EP2, CDH3, COL4A1, CTNNB1, CTSB, E2F8, EHF, GATA3, GJA1, GSK3B, HAS2, HEYL, ID2, IGF1R, IL6, IRF6, KIT, MYO10, NFIB, PFN2, PPARG, STMN3, TAGLN, TNF, TP53* | 6.39 x 10^-17^ | *ADM, AIF1, AKT3, APOD, AQP1, ARF1, ARPC2, AXIN2, BCCIP, CALB1, CCL11, CDC42, CDC42EP1, CDH3, CSK, DSE, EDA, EDNRA, EFNA1, EMP2, FGF2, FRZB, FST, FZD3, GAP43, GATA3, GJA1, GNAS, GSK3B, GSN, HAS2, HEY1, HHIP, ID2, IFT27, IGF1R, IL5, IL6, IRF6, ITIH4, KCNJ2, KCNMB1, KIF4A, KIT, LYVE1, MAFF, MAP2K4, MAP2K6, MAPK3, PFN2, PIK3CA, PIK3R1, PIK3R3, PLA2G5, PLD1, PRKCA, PTCH2, RBPJ, RDH10, S100B, S1PR1, SMARCA4, SNX10, STAT5A, SUFU, TAGLN, TCF7L2, TDRD7, TFAP2A, TFDP1, TGFB1I1, TNF, TNNC1, TP53, UPK1A, UPK1B, UPK2, WNT11, WNT16* |
| *OLR1* | Trans-membrane receptor | *APP, FYN, IL6, TNF, TP53* | 8.42 x 10^-17^ | *ADIPOQ, ADM, APOD, APP, AQP1, BCCIP, BDNF, BMP4, CALB1, CDH3, CITED2, COL4A3BP, CSK, DCN, EFNA1, EHF, ENPP2, FGF2, FYN, FZD1, GAP43, GJA1, GLI2, GNAS, HAS2, ID2, IL6, IRF6, KCNJ2, KIT, KITLG, KRT17, MAP2K6, MAPK3, MEF2A, MEF2C, PIK3R1, PLA2G6, PLD1, PRKCA, PTCH1, RAC1, RBPJ, S1PR1, SDC2, SEMA3C, STAT5A, TAGLN, TFDP1, TGFB1I1, TGM2, TNF, TNNC1* |
| *RIPK1* | Kinase | *ADIPOQ, AXIN2, BDNF, GNAS, GSK3B, IL5, IL6, MEF2C, PPARG, SLC35D1, TAGLN, TBX6, TGM2, TNF, TP53* | 8.46 x 10^-17^ | *ACADVL, ADIPOQ, ADM, AKT3, APOD, APP, AQP1, BDNF, BMP4, CALB1, CAV3, CCL11, CDK5R1, CITED2, COL4A1, EFNA1, EHF, FGF2, FRZB, FST, FYN, FZD1, GAP43, GLI2, GNAS, GSK3B, HAS2, IL6, KIT, KITLG, KRT17, KYAT3, MAP2K6, MAPK3, MEF2C, PLA2G12A, PLA2G6, PLCG2, PLD1, PRKACA, PTCH1, RAC1, RBPJ, RDH10, SDC2, STAT5A, STMN1, TAGLN, TDRD7, TGM2, TNF, TNNC1, TP53, WNT11, WNT16, WNT7A* |
| *BTRC* | Enzyme | *APP, AQP1, ATP2C1, BDNF, CITED2, COL4A1, CTNNB1, DCN, ENPP2, ERCC3, GAP43, GSN, KCNJ2, NCS1, NFIB, PFN2, PIK3CA, PPARG, PRKCB, SNAP25, STMN1, TAGLN, TBX6, TGM2, TP53, UCHL1* | 9.53 x 10^-17^ | *ACADVL, ADIPOQ, ADM, AKT3, APOD, AXIN2, BCCIP, BDNF, BMP4, CALB1, CDC42, CITED2, CLTC, COL4A3BP, CSK, CTNNB1, CTSB, CTSH, DCN, EFNA1, EHF, FGF2, GAP43, GJA1, GNAS, GSK3B, HEY1, HHIP, ID2, IL6, INPP5D, KCNJ2, KCNMA1, KIT, KRT17, MAP2K6, MAP2K7, MAPK12, MYH10, NME2, PAFAH1B2, PDE2A, PIK3CA, PIK3R1, PIK3R3, PLD1, PPARG, PRKACA, PRKCB, PSEN2, RAC1, RAC2, RBPJ, RDH10, S1PR1, SEMA3C, ST14, STAT5A, STMN1, SUFU, TCF7L2, TFDP1, TGFB1I1, TGM2, TRAF6, UPK1A, UPK1B, UPK2, WNT2, WNT7A* |
| *AMPK*  *(PRKAA,*  *PRKAA1,*  *PRKAA2,*  *PRKAB,*  *PRKAB1,*  *PRKAB2,*  *PRKAG,*  *PRKAG1,*  *PRKAG2)* | Complex | *GNAS, IL6, PIK3CA, PIK3R1, PLD1, TP53* | 9.88 x 10^-17^ | *ACADVL, ADIPOQ, ADM, AIF1, AKT3, APOD, APP, ARF1, ARPC2, BMP4, CDC42EP1, CDH3, CDK5R1, CITED2, CLTC, COL4A3BP, CTNNB1, CTSB, CTSH, DPYSL2, DSE, DVL2, EDNRA, ELF5, ENPP2, FST, FYN, FZD3, GLI2, GNAS, GSK3B, GSN, HHIP, IL6, IRF6, ITIH3, ITIH4, ITIH5, KCNMA1, KCNMB1, KITLG, KRT17, KYAT3, LGR4, LMO4, MAFF, MAP2K4, MAP2K6, MAP2K7, MAPK11, MAPK9, MYO10, NFIB, PAFAH1B2, PDE2A, PFN2, PIK3CA, PLA2G5, PLD1, PRKCA, PSEN2, PTCH1, PTCH2, RAF1, RBPJ, S1PR1, SDC2, SMARCA4, SNAP25, ST14, STAT5A, STK4, STMN1, TAGLN, TDRD7, TFDP1, TGFB1I1, TMEM17, TNF, TRAF6, UCHL1, WNT16, WNT2, WNT7A* |
| HDAC1/2  *(HDAC1,*  *HDAC1L,*  *HDAC2)* | Group | *APC, APP, FZD1, FZD6, PTCH1, TNF* | 1.08 x 10^-16^ | *ACADVL, ADM, AIF1, AKT3, APOD, APP, ARPC2, AXIN2, BCCIP, CAPRIN1, CAPZB, CCL11, CDC42, CDH3, CITED2, CLTC, COL4A3BP, CSK, CTNNB1, CTSB, CTSH, DPYSL2, DSE, DVL2, E2F8, EDA, EDNRA, EFNA1, ELF5, ENPP2, FGF2, FRZB, FST, FYN, FZD3, GATA5, GLI2, GSK3B, GSN, HAND1, HAS2, HHIP, ID2, IFT27, IRF6, ITIH3, KCNJ2, KCNMA1, KCNMB1, KCNQ3, KRT5, LMO4, LYVE1, MAP2K4, MAP2K6, MAP2K7, MAPK11, MAPK12, MEF2C, MYH10, NFIB, PADI1, PAFAH1B2, PDE2A, PIK3CA, PIK3R1, PIK3R2, PIK3R3, PLA2G12A, PLA2G5, PLCG1, PLD1, PPARG, PRKACA, PRKCA, PSEN2, PTCH1, PTCH2, RAC2, RAF1, RBPJ, S100B, SLC35D1, ST14, STAT5A, STAT5B, STMN1, STMN3, SUFU, TAGLN, TCF7L2, TDRD7, TFAP2A, TFDP1, TGFB1I1, TMEM100, TMEM17, TNNC1, TP53, UCHL1, UPK1A,UPK1B, UPK2, WNT16, WNT2, WNT2B, WNT7A* |
| *NCOA3* | Transcription regulator | *ADM, APP, BDNF, CCL11, CITED2, CTNNB1, FGF2, FST, GAP43, GJA1, GNAS, IL5, IL6, PLA2G2A, PPARG, PSEN2, TNF, TP53* | 1.26 x 10^-16^ | *AKT3, APC, ARFIP2, BCCIP, BDNF, CALB1, CAV3, CCL11, CDC42, CDH3, CITED2, COL4A1, CPE, CTSB, CTSH, DNM2, DPYSL2, E2F8, EDNRA, EFNA1, EHF, ELF5, ENPP2, EXT1, F11R, FGF2, FLOT1, FZD1, FZD6, GAP43, GLI2, GNAS, GSK3B, GSN, HEYL, IGF1R, IL5, KCNMA1, KRT17, MAFF, MAP2K6, MAP2K7, MAPK11, MAPK12, MAPK3, MEF2C, MYH10, MYO10, NFIB, NME7, PFN2, PLA2G2A, PLCG1, PPARG, PRKCA, PTCH1, PTCH2, RDH10, SCN1B, SEMA3C, SLC9A3R1, SNAP25, SOS1, STAT5A, STAT5B, STK4, STMN3, SUFU, TAGLN, TCF7L2, TGM2, TRAF6, WASF2, WNT11, WNT16, WNT2, WNT2B, WNT7A, WNT8B* |
| *ESR2* | Ligand-dependent nuclear receptor | *ADIPOQ, ENPP2, IL6, PPARG, TNF* | 1.45 x 10^-16^ | *ADM, AKT3, APC, APOD, APP, AQP1, ARFIP2, ARPC2, ATP6V0D1, BCCIP, BMP4, CALB1, CAV3, CDC42, CDC42EP2, CITED2, CLTC, COL4A3BP, CPE, CTNNB1, DNM2, DSE, EDA, EDNRA, EHF, ENPP2, F11R, FRZB, FZD1, GATA3, GLI2, GNAS, GSK3B, GSN, HEY1, IGF1R, IL5, KCNJ2, KCNMA1, KCNQ3, KIT, KRT17, MAFF, MAP2K6, MAP2K7, MAPK11, MAPK12, MAPK3, MYH10, NFIB, PADI1, PFN2, PLA2G2A, PLCG1, PLD1, PTCH1, PTCH2, RAC1, RBPJ, RDH10, S1PR1, SEMA3C, SNAP25, SOS1, STAT5A, STAT5B, STK4, STMN1, SUFU, TAGLN, TCF7L2, TDRD7, TFDP1, TGM2, TNF, TNNC1, TRAF6, WASF2, WNT16* |
| *CD2* | Trans-membrane receptor | *CTNNB1, GSK3B, IGF1R, IL6, PRKCA, PRKCB, TP53* | 1.64 x 10^-16^ | *ADIPOQ, AIF1, APOD, APP, AQP1, ARPC2, BMP4, CCL11, COL4A3BP, CTNNB1, EDA, EDNRA, EFNA1, ELF5, ENPP2, FGF2, FST, GATA3, GJA1, GNAS, HEY1, ID2, IGF1R, IL5, KCNQ3, KIT, KITLG, LMO4, MAFF, MAP2K6, MAP2K7, MAPK3, MAPK9, MEF2C, PADI1, PLA2G12A, PLA2G2A, PLA2G5, PLCG1, PLD1, PSEN2, PTCH1, RAC1, RAC2, SNAP25, STAT5A, TAGLN, TNNC1, TP53* |
| *SPEN* | Transcription regulator | *ENPP2, FGF2, GAP43, HAS2, IL6, PLA2G2A, S1PR1, TAGLN, TNF* | 1.65 x 10^-16^ | *ACADVL, ADIPOQ, ADM, AKT3, APC, APOD, AQP1, ARFIP2, ARPC2, ATP6V0D1, BCCIP, BMP4, CDC42, CDC42EP2, CLTC, COL4A3BP, CPE, CTNNB1, DNM2, DVL2, E2F8, EDA, EDNRA, EFNA1, EMP2, ENPP2, ERCC3, F11R, FCER1A, FGF2, FLOT1, FOXP1, FST, GATA3, GJA1, GLI2, GNAS, GSK3B, GSN, HEY1, HEYL, ID2, IGF1R, IL6, INPP5D, IRF6, KCNMA1, KCNQ3, KIF4A, KRT5, KYAT3, LYVE1, MAP2K6, MAP2K7, MAPK11, MAPK12, MEF2A, MEF2C, MYH10, NFIB, PFN2, PLA2G12A, PLA2G2A, PLA2G6, PLCG1, PLPP3, PPARG, PRKCA, PRKCB, PTCH1, PTCH2, RAF1, RDH10, SCN1B, SDC2, SEMA3C, SLC9A3R1, SNAP25, SOS1, STAT5A, STAT5B, STK4, STMN1, SUFU, TAGLN, TCF7L2, TFAP2A, TFDP1, TGFB1I1, TGM2, TNNC1, TP53, TRAF6, WASF2* |
| *IFNGR2* | Trans-membrane receptor | *AGPS, ARF1, ASAP1, ATP2C1, BDNF, CTSB, CTSH, DOCK1, FGF2, FRZB, FZD3, FZD6, GJA1, GSK3B, HAS2, IL6, KITLG, PLD1, STMN1, TNF, TP53, WNT11* | 1.7 x 10^-16^ | *AIF1, AIMP2, APC, AQP1, ARF1, ARPC2, BCCIP, CAPZB, CCL11, CDC42, CDC42EP1, CDC42EP2, CHN1, CSK, CTSH, DCN, DVL2, E2F8, EDA, EMP2, ENPP2, FOXP1, FST, FZD3, GAP43, GATA3, GJA1, GSN, HAS2, HEY1, HEYL, HHIP, ID2, IGF1R, IL5, IL6, INPP5D, IRF6, ITIH4, ITIH5, KCNJ2, KCNMB1, KCNQ3, KIF4A, KITLG, LGR4, LMO4, MAFF, MAP2K4, MEF2A, MEF2C, MYH10, MYO10, NFIB, PADI1, PAFAH1B1, PDE2A, PIK3R3, PLA2G5, PLA2G6, PLCG1, PPARG, PRKACA, PRKCA, PTCH2, RAC2, RBPJ, RDH10, S100B, S1PR1, SEMA3C, SMARCA4, SNAP25, SNX10, SOS1, STAT5B, STMN3, SUFU, TAGLN, TFAP2A, TFDP1, TGFB1I1, TGM3, TRAF6, UPK1A, UPK1B, UPK2, WNT11* |
| *E. coli* B4 lipopolysaccharide | Chemical toxicant | *ADM, AIF1, BDNF, CALB1, GATA3, GNAS, IL6, MAPK3, TNF, TP53, TRAF6* | 1.72 x 10^-16^ | *ADIPOQ, ADM, AIF1, AKT3, APOD, APP, AQP1, ARPC2, BCCIP, BDNF, CALB1, CCL11, CDH3, CDK5R1, CITED2, COL4A1, COL4A3BP, CSK, CTSB, CTSH, DCN, DSE, EFNA1, EHF, ENPP2, FOXP1, FRZB, FST, FYN, GAP43, GATA3, GLI2, GNAS, GSK3B, GSN, HAS2, IL5, IL6, INPP5D, KIT, KITLG, MAP2K6, MEF2C, PIK3CA, PIK3R1, PLCG1, PLCG2, PLD1, RAC1, RBPJ, RDH10, SDC2, SEMA3C, SNAP25, SNX10, STAT5A, STMN1, TAGLN, TFDP1, TGFB1I1, TGM2, TNF, TNNC1, TOLLIP, TP53, TRAF6, WNT16* |
| *JMY-P300*  *(P300, JMY)* | Complex | *BMP4, CCL11, GJA1, HEY1, ID2, IL6, KIT, PTCH1, TAGLN, TGM2, TP53* | 2.05 x 10^-16^ | *ACADVL, AIF1, AKT3, APC, APOD, APP, ARF1, ARFIP2, ARPC2, ATP6V0D1, AXIN2, BCCIP, CALB1, CAPRIN1, CAPZB, CDC42EP2, CDH3, CLTC, COL4A1, COL4A3BP, CPE, CTNNB1, CTSB, CTSH, DCN, DNM2, DPYSL2, DVL2, E2F8, EDA, EDNRA, EFNA1, ELF5, ENPP2, ERCC3, FYN, FZD1, FZD3, GATA5, GNA11, GSK3B, GSN, HAND1, HAS2, HHIP, ID2, IGF1R, IL5, IL6, INPP5D, KCNB2, KCNJ2, KCNMA1, KCNMB1, KCNQ3, LYVE1, MAP2K4, MAP2K6, MAP2K7, MAPK10, MAPK12, MEF2A, MYH10, NFIB, PADI1, PAFAH1B1, PAFAH1B2, PDE2A, PIK3CA, PIK3R2, PIK3R3, PLA2G5, PLD1, PPARG, PRKACA, PRKCA, PRKCB, PSEN2, PTCH1, PTCH2, RAC1, RAF1, RBPJ, RDH10, SEMA3C, SLC9A3R1, SOS1, ST14, STAT5A, STAT5B, STK4, STMN1, STMN3, SUFU, TCF7L2, TFAP2A, TFDP1, TGFB1I1, TGM2, TMEM100, TNF, TNNC1, TP53, WASF2, WNT2, WNT2B, WNT7A* |
| *TIRAP* | Other | *ADM, AQP1, BDNF, BMP4, CPE, FLOT1, GAP43, HAS2, IL6, MAFF, SCN1B, SLC9A3R1, TAGLN, TNF, TP53* | 2.21 x 10^-16^ | *ADM, AKT3, APOD, APP, AQP1, ARPC2, BCCIP, BDNF, BMP4, CALB1, CDH3, CDK5R1, CITED2, COL4A3BP, CSK, CTNNB1, CTSB, DCN, EFNA1, EHF, ENPP2, FGF2, FRZB, FST, GAP43, GATA3, GLI2, GNAS, GSK3B, HAS2, HEY1, IL6, KIT, KITLG, KRT17, LMO4, MAFF, MAP2K6, MEF2C, PIK3R1, PLCG2, PLD1, PRKCA, PTCH1, RAC1, RBPJ, RDH10, SDC2, SEMA3C, STMN1, TAGLN, TFDP1, TGFB1I1, TGM2, TNF, TNNC1, TRAF6, WNT16* |
| Sb1578 | Chemical drug | *CALB1, CTSB, DCN, DPYSL2, GJA1, IL5, IL6, TNF, TP53, WNT11* | 2.46 x 10^-16^ | *AIMP2, AKT3, ANXA7, APC, AQP1, ARF1, ARPC2, AXIN2, BCCIP, BDNF, CALB1, CDC42, CDC42EP1, CDC42EP2, CDK5R1, CHN1, CITED2, CSK, CTSB, CTSH, DCN, DSE, DVL2, E2F8, EDA, EFNA1, EHF, ENPP2, FGF2, FRZB, FYN, FZD3, GLI2, GNAS, GSN, HEYL, HHIP, IGF1R, IRF6, ITIH5, KCNQ3, KIT, LGR4, MAFF, MAP2K4, MAPK11, MEF2A, MEF2C, MYH10, MYO10, NFIB, NME2, PADI1, PFN2, PIK3R2, PLA2G5, PLA2G6, PLCG1, PLD1, PRKACA, PTCH1, PTCH2, RAC1, RBPJ, RDH10, S100B, S1PR1, SDC2, SEMA3C, SNAP25, SOS1, STAT5B, STMN3, TAGLN, TCF7L2, TDRD7, TFDP1, TGFB1I1, TGM2, VAV1, WNT11, WNT16* |
| *PTPN4* | Phosphatase | *ACADVL, GATA5, GLI2, HHIP, ID2, IL6, MAPK11, MEF2C, PTCH1, PTCH2, TMEM100, TP53, WNT2B* | 2.73 x 10^-16^ | *ACADVL, ADIPOQ, AIF1, AKT3, APC, APP, AXIN2, BDNF, CALB1, CAPZB, CAV3, CDH3, CDK5R1, CITED2, CTNNB1, CTSH, DSE, EFNA1, ELF5, ENPP2, F11R, FOXP1, FST, FYN, FZD1, GAP43, GATA3, GNA11, GSK3B, GSN, HAS2, ID2, IGF1R, KCNB2, KCNJ2, KCNMA1, KITLG, KRT17, MAFF, MAP2K6, MAP2K7, MAPK10, MAPK11, MAPK3, MEF2C, MYH10, PAFAH1B2, PDE2A, PFN2, PIK3CA, PLA2G12A, PLA2G2A, PLA2G5, PLD1, PRKCA, PRKCB, PSEN2, PTCH1, RAC1, RBPJ, SDC2, SLC9A3R1, SNAP25, ST14, STAT5A, STMN1, TAGLN, TCF7L2, TDRD7, TGFB1I1, TGM2, TNF, TNNC1, VAV1, WNT2, WNT7A* |
| *F2R* | G-protein coupled receptor | *CDK5R1, CITED2, COL4A3BP, EDNRA, FGF2, FST, IGF1R, IL6, KIT, MAP2K7, MAPK3, MEF2C, PPARG, PSEN2, TFDP1, TNF, TNNC1, TP53* | 2.73 x 10^-16^ | *APOD, APP, AQP1, ARPC2, BDNF, CALB1, CAPRIN1, CCL11, CDC42, CDC42EP2, CDH3, CDK5R1, COL4A1, COL4A3BP, CTSH, DCN, E2F8, EDNRA, EHF, EXT1, FCER1A, FST, FZD1, GAP43, GJA1, GNAS, GSK3B, HEYL, IL6, INPP5D, IRF6, ITIH3, KCNJ2, KCNQ3, KCNV1, KITLG, KRT17, LYVE1, MAFF, MAP2K4, MAPK3, MEF2C, MYO10, NFIB, PIK3CA, PIK3R1, PIK3R2, PLA2G5, PLA2G6, PLCG1, PLD1, PLPP3, PRKCA, PTCH1, RBPJ, RDH10, S1PR1, SDC2, SOS1, STAT5A, STAT5B, STMN1, STMN3, TAGLN, TFDP1, TGFB1I1, TGM2, TMEM17, TNNC1, TP53, VAV2, WNT11, WNT7A* |
| Okadaic acid | Chemical toxicant | *ENPP2, GJA1, IL6, KIT, KITLG, NME2, PPARG, PRKCA, PRKCB, TFDP1, TNF, TP53* | 3.36 x 10^-16^ | *ACADVL, ADM, AIF1, AKT3, APOD, APP, AQP1, ARPC2, BCCIP, BDNF, CALB1, CAPZB, CDH3, CDK5R1, CITED2, COL4A3BP, CSK, DCN, DPYSL2, DSE, DVL2, EDA, EDNRA, EFNA1, EHF, ERCC3, FGF2, FRZB, FST, FYN, FZD3, FZD6, GLI2, GNAS, HAND1, HHIP, IFT27, IL6, IRF6, KCNJ2, KCNMA1, KITLG, LMO4, MAFF, MAP2K4, MAP2K6, MAP2K7, MAPK11, MAPK12, MAPK9, MYH10, NFIB, PADI1, PAFAH1B2, PDE2A, PIK3R1, PIK3R2, PLA2G2A, PLA2G5, PLA2G6, PLCG1, PRKCA, PSEN2, PTCH1, PTCH2, RAF1, RBPJ, RDH10, S100B, SDC2, SEMA3C, SLC35D1, SMARCA4, ST14, STAT5B, STK4, TDRD7, TGFB1I1, TGM2, TNF, TNNC1, TRAF6, WNT16, WNT2, WNT2B, WNT7A, WNT8B* |
| Muromonab-CD3 | Biologic drug | *APP, CALB1, CDK5R1, COL4A1, EHF, GATA3, ID2, IGF1R, IL5, IL6, KCNH1, PADI1, PLCG1, PLD1, PPARG, PRKCA, PRKCB, STMN1, TMEM100, TNF, TP53, WNT11* | 4.04 x 10^-16^ | *ADM, AIF1, AKT3, AQP1, ARPC2, BCCIP, BMP4, CALB1, CDC42EP1, CDH3, CHN1, COL4A1, COL4A3BP, CSK, CTSH, DCN, DOCK1, DSE, EFNA1, EHF, ELF5, ENPP2, FOXP1, FRZB, FYN, FZD1, GNAS, GSK3B, GSN, HAND1, HEY1, IL5, IL6, IRF6, KCNJ2, KCNQ3, KIF4A, KIT, KITLG, MAFF, MAP2K6, MAP2K7, MEF2A, MYH10, PIK3CA, PIK3R1, PLA2G12A, PLA2G6, PLCG1, PRKACA, PRKCB, RAC1, RAC2, RBPJ, RDH10, S100B, S1PR1, SDC2, SEMA3C, STAT5A, STMN1, TAGLN, TDRD7, TFDP1, TGFB1I1, TNF, TNNC1, VAV1, WNT16* |
| *MAP3K2* | Kinase | *CALB1, FGF2, HAS2, IL6, TNF* | 4.11 x 10^-16^ | *ADIPOQ, AKT3, APOD, APP, AQP1, ARPC2, BCCIP, CALB1, CDH3, CDK5R1, CITED2, CSK, CTSB, DCN, DSE, EFNA1, EHF, ENPP2, FGF2, FRZB, FST, FYN, FZD1, GAP43, GATA3, GLI2, GNAS, GSK3B, HAND1, HAS2, IGF1R, IL6, KIT, KITLG, LMO4, MAFF, MAP2K4, MAP2K6, MAPK9, MEF2A, MEF2C, PIK3R1, PLD1, PTCH1, RAC1, RBPJ, SDC2, SEMA3C, SNX10, STMN1, TAGLN, TDRD7, TFDP1, TGFB1I1, TGM2, TNF, TNNC1, WNT16, WNT7A* |
| Bapta-am | Chemical reagent | *CCL11, CTNNB1, DCN, FGF2, FST, GATA3, GLI2, HEY1, IL5, IL6, INPP5D, PLPP3, TAGLN, TNF* | 4.97 x 10^-16^ | *ADM, AGPS, AIF1, AKT3, APC, APOD, APP, AQP1, ARF1, ARFIP2, ARPC2, ATP6V0D1, BDNF, BMP4, CALB1, CAPZB, CDC42, CDC42EP2, CDH3, CDK5R1, CITED2, COL4A1, COL4A3BP, CPE, CSK, DCN, DNM2, EHF, ENPP2, FRZB, FST, FYN, FZD1, GAP43, GATA3, GHRL, GNAS, GSN, HEY1, ID2, IL6, IRF6, KIT, KRT17, LMO4, MAFF, MAP2K4, MAP2K6, MAP2K7, MAPK12, MEF2A, MEF2C, MYH10, NFIB, PFN2, PIK3CA, PIK3R1, PLA2G2A, PLA2G3, PLCG1, PLCG2, PLD1, PLPP3, PRKACA, PRKCA, PTCH1, PTCH2, RBPJ, RDH10, S100B, SDC2, SEMA3C, SOS1, STK4, SUFU, TAGLN, TCF7L2, TDRD7, TFDP1, TGFB1I1, TNF, TNNC1, TTC30B, TTLL1, WASF2, WNT16* |
| *FCER1*  *(FCER1A, FCER1G, MS4A2)* | Complex | *CTNNB1, FGF2, GATA3, GLI2, HEY1, HEYL, ID2, IGF1R, IL6, LYVE1, PPARG, TAGLN, TNF, TP53* | 5.89 x 10^-16^ | *ADIPOQ, ADM, AIF1, AKT3, APOD, AQP1, ARPC2, BCCIP, BDNF, CALB1, CDH3, CDK5R1, CITED2, COL4A1, CSK, DCN, DSE, EFNA1, EHF, ENPP2, FGF2, FOXP1, FRZB, FYN, FZD1, GAP43, GLI2, GNAS, GSK3B, GSN, HEY1, ID2, IL5, IL6, KCNJ2, KIT, KITLG, MAFF, MAP2K4, MEF2C, PIK3CA, PIK3R1, PLA2G2A, PLA2G5, PLA2G6, PLCG1, PLCG2, PLD1, PRKCA, PTCH1, RBPJ, RDH10, SDC2, SEMA3C, STAT5A, STAT5B, STK4, TDRD7, TFDP1, TGFB1I1, TGM2, TNF, TNNC1, VAV1, WNT16* |
| Pyrrolidine dithiocarba-mate | Chemical reagent | *CDH3, CTNNB1, ENPP2, ERCC3, IGF1R, KIT, KRT17, KRT5, STAT5A, STAT5B, TNF, TP53* | 7.09 x 10^-16^ | *ADIPOQ, ADM, AIF1, AKT3, AQP1, ARPC2, AXIN2, BCCIP, CALB1, CAV3, CDH3, CITED2, COL4A1, COL4A3BP, CSK, CTSB, DCN, EDNRA, EHF, ENPP2, FRZB, FYN, GATA3, GSK3B, ID2, IFT27, IGF1R, IL6, ITIH4, KIT, KITLG, KRT17, MAFF, MAP2K6, MAPK11, MAPK3, MAPK9, MEF2A, MEF2C, PIK3R1, PLA2G3, PLD1, PPARG, PRKACA, PTCH1, RAC1, RBPJ, RDH10, S1PR1, SDC2, SEMA3C, STAT5A, STMN1, TAGLN, TCF7L2, TFDP1, TGFB1I1, TNF, VAV1, WNT16* |
| *MIR-34* | MicroRNA | *ADIPOQ, ARF1, BDNF, FGF2, GATA3, IGF1R, IL6, MAPK3, PIK3R1, PIK3R2, PPARG, PRKCA, PRKCB, SNAP25, STAT5A, TNF* | 7.88 x 10^-16^ | *ACADVL, ADIPOQ, AIF1, AKT3, APC, APP, BMP4, CDC42, CDH3, CITED2, COL4A1, CTNNB1, CTSB, EFNA1, ENPP2, EXT2, FRZB, FST, FYN, GAP43, GATA3, GJA1, GLI2, GSN, HAND1, HAS2, HEY1, IL5, IL6, IRF6, KCNMA1, KITLG, MAP2K6, MAP2K7, MAPK12, MEF2C, MYH10, NME2, PAFAH1B2, PDE2A, PIK3R1, PLCG1, PRKCA, PSEN2, PTCH1, RBPJ, SDC2, SNAP25, ST14, STMN1, TAGLN, TBX6, TCF7L2, TFDP1, TGFB1I1, TGM2, TP53, UCHL1, WNT2, WNT7A, WNT8B* |
| Diethylstilbe-strol | Chemical drug | *APP, BDNF, CALB1, FGF2, IL6, MAP2K4, TNF, TP53* | 8.97 x 10^-16^ | *ADIPOQ, AKT3, APC, APOD, APP, AQP1, ARFIP2, ARPC2, ATP6V0D1, AXIN2, BCCIP, BDNF, BMP4, CAV3, CCL11, CDH3, CDK5R1, CITED2, CPE, CTSB, DNM2, E2F8, EFNA1, EHF, ENPP2, F11R, FGF2, FRZB, FST, FZD1, GAP43, GATA3, GJA1, GNAS, GSN, HEY1, HEYL, ID2, IL5, INPP5D, ITIH4, KCNMA1, KIF4A, KRT5, MAFF, MAP2K6, MAP2K7, MAPK11, MAPK12, MAPK3, MEF2C, MYH10, MYO10, NFIB, PFN2, PLCG1, PTCH1, PTCH2, S1PR1, SEMA3C, SLC9A3R1, SMARCA4, SOS1, STAT5A, STAT5B, STK4, STMN3, SUFU, TCF7L2, TGM2, WASF2, WNT11, WNT7A* |
| *DICER1* | Enzyme | *APP, CCL11, CITED2, COL4A3BP, CTSB, EHF, FGF2, GLI2, HAS2, IGF1R, IL6, KIT, PLD1, PPARG, STAT5A, TGM2, TNF, TP53* | 1.02 x 10^-15^ | *ACADVL, ADIPOQ, ADM, AIF1, AKT3, APOD, AQP1, ARF1, AXIN2, BDNF, CAV3, CDC42, CDC42EP2, CDK5R1, CITED2, COL4A1, CTSB, CTSH, DPYSL2, E2F8, EDNRA, EFNA1, EHF, ELF5, ENPP2, FCER1A, FGF2, FYN, GAP43, GLI2, HEYL, ID2, KCNJ2, KCNMA1, KIF4A, KIT, KITLG, KRT17, MAP2K6, MAP2K7, MAPK11, MAPK12, MEF2C, MYH10, MYO10, PAFAH1B2, PDE2A, PFN2, PIK3R1, PLCG1, PRKCA, PSEN2, RAC1, RAF1, RBPJ, RDH10, SEMA3C, SLC9A3R1, SNAP25, ST14, STAT5A, STAT5B, STMN1, STMN3, TCF7L2, TDRD7, TFAP2A, TFDP1, TGFB1I1, TGM2, TNF, TP53, VAV1, WNT2, WNT7A* |
| Imidapril | Chemical drug | *BDNF, BMP4, CALB1, CTNNB1, DCN, ENPP2, FGF2, GAP43, GJA1, HAS2, IGF1R, IL6, LYVE1, PPARG, TAGLN, TNF, TP53* | 1.15 x 10^-15^ | *AIMP2, AKT3, APC, APOD, AQP1, ARF1, ARFIP2, ARPC2, ATP6V0D1, BCCIP, BMP4, CALB1, CCL11, CDC42, CDC42EP1, CDC42EP2, CDH3, CHN1, COL4A3BP, CPE, DCN, DNM2, DPYSL2, DSE, DVL2, E2F8, EDNRA, EHF, EMP2, EXT2, FGF2, FOXP1, FRZB, FST, FYN, GAP43, GATA3, GJA1, GNA11, GNAS, GSN, HAND1, HAS2, HEY1, ID2, IGF1R, IL6, INPP5D, ITIH3, ITIH5, KCNB2, KCNJ2, KCNMA1, KCNMB1, KIF4A, KIT, KITLG, KRT5, LMO4, MAFF, MAP2K4, MAP2K7, MAPK10, MEF2A, MEF2C, NFIB, NME2, PAFAH1B1, PAFAH1B2, PDE2A, PIK3R3, PLA2G2A, PLA2G5, PLCG1, PLCG2, PPARG, PRKCB, PSEN2, PTCH1, RAC1, RAC2, RAF1, RBPJ, S100B, S1PR1, SMARCA4, SNX10, SOS1, ST14, STAT5B, STK4, TAGLN, TDRD7, TFDP1, TGFB1I1, TGM3, TMEM17, TP53, TRAF6, UCHL1, UPK1A, UPK1B, UPK2, WASF2, WNT16, WNT2, WNT7A, WNT8B* |
| *FAM20C* | Enzyme | *ADM, BDNF, ENPP2, FST, FYN, FZD1, GHRL, GJA1, HAS2, IGF1R, IL6, MAPK3, PLPP3, PPARG, SNAP25, TNF, TP53* | 1.16 x 10^-15^ | *ADIPOQ, ADM, AIF1, AKT3, APP, ARF1, AXIN2, CCL11, CDC42, CITED2, COL4A3BP, CPE, CTNNB1, CTSB, DCN, DPYSL2, FZD3, GAP43, GATA3, GJA1, GLI2, GSK3B, HAS2, HEY1, ID2, IGF1R, ITIH1, KITLG, MAFF, MAPK3, MEF2C, MYH10, PIK3R1, PLA2G10, PLA2G2A, PLCG2, PLD1, PPARG, PRKACA, PRKCA, PTCH2, RAF1, RBPJ, S100B, S1PR1, TCF7L2, TDRD7, TGM2, TP53, TRAF6* |
| *ALP*  *(AKP1,*  *AKP4, ALPI, ALPL, ALPP,ALPPL2,*  *JEVR)* | Group | *CDK5R1, GAP43, GATA3, GJA1, GLI2, IGF1R, IL6, MAP2K4, MEF2C, PLA2G5, STMN1, TAGLN, TGM2, TNF, TP53* | 1.27 x 10^-15^ | *ACADVL, ADIPOQ, APP, ARF1, ARPC2, AXIN2, BCCIP, BDNF, BMP4, CALB1, CAPRIN1, CCL11, CDC42EP2, CLTC, CSK, CTSH, DCN, DPYSL2, DSE, E2F8, EDA, EDNRA, EHF, ELF5, ENPP2, ERCC3, EXT1, F11R, FCER1A, FOXP1, FZD1, FZD3, GJA1, GNA11, GNAS, HEYL, HHIP, ID2, IL6, INPP5D, ITIH3, KCNB2, KCNMA1, KCNV1, KRT5, LIN7C, LMO4, LYVE1, MAFF, MAP2K7, MAPK10, MAPK12, MEF2C, MYH10, MYO10, NFIB, PADI1, PAFAH1B1, PAFAH1B2, PDE2A, PIK3CA, PIK3R1, PLA2G12A, PLA2G2A, PLA2G6, PLCG1, PLD1, PLPP3, PPARG, PRKCA, PRKCB, PSEN2, PTCH2, RAC2, RAF1, RBPJ, RDH10, S100B, S1PR1, SCN1B, SDC2, SMARCA4, SNAP25, ST14, STAT5A, STAT5B, STMN3, TCF7L2, TDRD7, TMEM17, TNF, TNNC1, TP53, TRAF6, VAV1, WNT2, WNT7A* |
| Sulindac | Chemical drug | *ADM, AQP1, CCL11, FGF2, FST, GJA1, HAS2, IL6, KIT, NME2, NPY1R, SNAP25, STAT5A, TNF, TP53* | 1.35 x 10^-15^ | *ACADVL, ADIPOQ, ADM, APP, AQP1, AXIN2, BCCIP, BDNF, BMP4, CALB1, CAV3, CDH3, COL4A1, CSK, CTNNB1, CTSH, DCN, E2F8, EDNRA, EFNA1, EHF, ENPP2, F11R, FGF2, FST, FYN, FZD3, GATA3, GJA1, GLI2, GNAS, HAS2, HEY1, HHIP, ID2, IFT27, IGF1R, KRT17, KYAT3, MAPK9, NFIB, PIK3R1, PLA2G12A, PLCG1, PLD1, PTCH1, PTCH2, RBPJ, SDC2, SEMA3C, SNX10, STAT5A, TAGLN, TCF7L2, TFDP1, TGFB1I1, TGM2, TRAF6, WNT11* |
| *AMOT* | Other | *ADM, APP, AQP1, ARPC2, BDNF, EFNA1, GAP43, GATA3, IGF1R, IL6, KRT17, KRT5, LMO4, MAPK3, MAPK9, PIK3R1, PIK3R3, PPARG, PRKCB, RAF1, SEMA3C, STAT5A, TNF, TP53* | 1.43 x 10^-15^ | *ACADVL, ADM, APOD, AQP1, CALB1, CDC42, CDC42EP1, CDC42EP2, CDH3, CDK5R1, CHN1, COL4A3BP, DCN, E2F8, EDNRA, EHF, ELF5, ENPP2, ERCC3, FRZB, FYN, GATA3, GATA5, GNAS, GSK3B, GSN, HEYL, HHIP, IL5, IL6, KCNMA1, KIT, KRT17, LMO4, LYVE1, MAP2K6, MAP2K7, MAPK11, MAPK12, MAPK9, MEF2C, MYH10, PAFAH1B2, PDE2A, PIK3CA, PIK3R3, PLA2G12A, PLA2G5, PLD1, PRKCA, PSEN2, PTCH2, RAC2, SDC2, ST14, STAT5A, SUFU, TAGLN, TCF7L2, TFDP1, TGFB1I1, TMEM100, TNF, TNNC1, UPK1A, UPK1B, UPK2, WNT16, WNT2, WNT2B, WNT7A* |
| *CD226* | Other | *APP, BDNF, IL6, MAP2K7, TNF* | 1.46 x 10^-15^ | *AIF1, AKT3, APOD, APP, AQP1, ARF1, ARPC2, BCCIP, BDNF, CALB1, CDH3, CDK5R1, CITED2, COL4A1, CSK, CTNNB1, CTSH, DCN, DSE, EFNA1, EHF, ENPP2, FGF2, FOXP1, FRZB, GLI2, GNAS, GSN, HAS2, ID2, IL6, IRF6, KCNJ2, KIT, KITLG, LMO4, MAFF, MAP2K4, MAP2K6, MAPK12, MEF2A, MEF2C, MYH10, PIK3CA, PIK3R1, PLA2G2A, PLA2G5, PLA2G6, PLCG1, PLD1, PRKACA, PRKCA, PRKCB, PTCH1, RBPJ, RDH10, S1PR1, SDC2, SEMA3C, SNX10, TFDP1, TGFB1I1, TGM2, TNF, TNNC1, TRAF6, WNT16* |
| Lapatinib | Chemical drug | *APC, BMP4, CALB1, CTNNB1, ERCC3, GHRL, ID2, IGF1R, IL6, PPARG, SMARCA4, STAT5A, TFAP2A* | 1.46 x 10^-15^ | *ADIPOQ, ADM, APC, AQP1, ARFIP2, ARPC2, ATP6V0D1, BCCIP, BMP4, CAV3, CDC42, CDH3, CDK5R1, CPE, DNM2, E2F8, EFNA1, EHF, ENPP2, FGF2, FST, GAP43, GATA3, GJA1, GLI2, GNAS, GSK3B, GSN, HAND1, HAS2, HEY1, HEYL, IL5, IL6, KCNMA1, KRT17, MAFF, MAP2K4, MAP2K6, MAP2K7, MAPK11, MAPK12, MEF2C, MYH10, MYO10, NFIB, PFN2, PIK3CA, PLA2G5, PLCG1, PPARG, PTCH1, PTCH2, RDH10, SEMA3C, SOS1, STAT5A, STAT5B, STK4, STMN1, STMN3, SUFU, TCF7L2, TGM2, WASF2* |
| Oxymatrine | Chemical drug | *CTSB, CTSH, FGF2, GSK3B, STMN1, TNF* | 1.74 x 10^-15^ | *AIMP2, AKT3, APC, APOD, APP, AQP1, ARF1, ARPC2, AXIN2, BCCIP, BMP4, CAPRIN1, CCL11, CDC42, CDC42EP2, CHN1, CLTC, CPE, CSK, CTNNB1, DCN, DOCK1, DPYSL2, DVL2, E2F8, EDA, EMP2, ENPP2, ERCC3, EXT1, FCER1A, FGF2, FOXP1, FRZB, FST, FYN, GAP43, GATA3, GJA1, GLI2, GNA11, HAND1, HAS2, HEY1, HEYL, ID2, IGF1R, INPP5D, IRF6, ITIH5, KCNB2, KCNJ2, KCNMA1, KCNMB1, KCNV1, KIF4A, KRT5, KYAT3, LGR4, LMO4, MAP2K4, MAP2K6, MAP2K7, MAPK10, MAPK11, MEF2A, MYO10, NFIB, PADI1, PAFAH1B1, PAFAH1B2, PDE2A, PIK3CA, PIK3R2, PIK3R3, PLA2G2A, PLA2G3, PLA2G5, PLCG1, PPARG, PRKACA, PRKCB, PSEN2, RAC1, RAC2, RBPJ, RDH10, S100B, S1PR1, SEMA3C, SMARCA4, SNX10, SOS1, ST14, STMN3, SUFU, TFAP2A, TFDP1, TNF, TP53, UCHL1, UPK1A, UPK1B, UPK2, VAV1, WNT2, WNT7A* |
| *ITCH* | Enzyme | *CDK5R1, FST, FYN, FZD1, GNA11, HAS2, MAFF, MAP2K4, MAP2K6, MAP2K7, PRKACA, PRKCA, TP53* | 1.76 x 10^-15^ | *ACADVL, ADIPOQ, ADM, AKT3, APOD, AQP1, AXIN2, BMP4, CALB1, CDC42, CDC42EP2, CDK5R1, COL4A1, COL4A3BP, CTSH, DCN, EFNA1, EHF, ELF5, ERCC3, FRZB, FZD1, GATA3, GATA5, GNAS, GSK3B, HEYL, HHIP, IL5, IL6, KIT, KRT17, LMO4, LYVE1, MAPK11, MAPK3, MAPK9, MEF2C, PIK3CA, PIK3R3, PLA2G12A, PLA2G5, PLD1, PTCH2, RAC2, RBPJ, SDC2, STAT5A, SUFU, TMEM100, TNF, TNNC1, UPK1A, UPK1B, UPK2, WNT16, WNT2, WNT2B, WNT7A* |
| *SPHK1* | Kinase | *ADIPOQ, CCL11, IL5, LYVE1, SDC2, TNF* | 1.78 x 10^-15^ | *ADIPOQ, AKT3, APOD, APP, AQP1, ARF1, ARPC2, AXIN2, BCCIP, CALB1, CDH3, COL4A1, CSK, DCN, DSE, EFNA1, EHF, FRZB, FST, FYN, FZD3, GLI2, GNAS, GSK3B, GSN, HAS2, HHIP, ID2, IFT27, IL6, INPP5D, IRF6, KCNJ2, KIT, MAFF, MAP2K6, MAPK12, MEF2A, MYH10, PADI1, PIK3R1, PLA2G2A, PLA2G2F, PLA2G6, PLCG1, PLD1, PRKCA, PTCH1, PTCH2, RBPJ, RDH10, SEMA3C, SNX10, STAT5A, STMN1, TCF7L2, TDRD7, TFDP1, TGFB1I1, TGM2, TNNC1, TP53, WNT11, WNT16* |
| Uric acid | Chemical - endogenous mammalian | *HEY1, IL6, SDC2, TAGLN, TNF, TP53* | 1.88 x 10^-15^ | *ADIPOQ, AIF1, AKT3, APP, ARPC2, BCCIP, BDNF, BMP4, CALB1, CDH3, CDK5R1, CITED2, CSK, CTSB, DCN, EFNA1, EHF, ENPP2, FGF2, FOXP1, FRZB, FYN, FZD1, GAP43, GATA3, GJA1, GNAS, GSK3B, HAS2, HEY1, IL5, IL6, KIT, KRT17, MAFF, MAPK11, PIK3CA, PIK3R1, PLD1, RAC1, RBPJ, SDC2, SEMA3C, STAT5A, STMN1, TFDP1, TGFB1I1, TGM2, TNF, TNNC1, TP53, VAV1, WNT16* |
| *TCRD* | Other | *AXIN2, CALB1, CDC42, CTNNB1, EDA, EFNA1, EHF, EMP2, ENPP2, FGF2, FST, IL6, KITLG, LYVE1, MEF2C, PADI1, PLPP3, PRKCB, RAC1, TNF* | 1.93 x 10^-15^ | *ACADVL, ADM, AIF1, AIMP2, AKT3, APC, APOD, APP, ARFIP2, ARPC2, ATP6V0D1, AXIN2, BCCIP, CAPZB, CCL11, CDC42, CDC42EP1, CDH3, CHN1, CITED2, COL4A1, CPE, CTNNB1, CTSH, DCN, DNM2, DSE, DVL2, E2F8, EHF, ENPP2, EXT2, FOXP1, FRZB, FYN, FZD1, GATA3, GLI2, GNAS, GSN, HAND1, HEYL, ID2, IFT27, IGF1R, IL5, IL6, ITIH5, KCNMA1, KIT, KRT5, LMO4, MAFF, MAP2K4, MEF2A, MYO10, NFIB, NME2, PAFAH1B2, PDE2A, PFN2, PIK3R1, PIK3R2, PLA2G5, PLCG1, PLD1, PRKACA, PRKCA, PTCH2, RAC1, RAC2, RAF1, RBPJ, RDH10, S1PR1, SDC2, SNAP25, SOS1, ST14, STAT5A, STAT5B, STMN3, SUFU, TAGLN, TDRD7, TGFB1I1, TNF, TP53, VAV1, WASF2, WNT16, WNT2, WNT7A, WNT8B* |
| *CX3CL1* | Cytokine | *ADIPOQ, BDNF, CAV3, CCL11, CTSB, FGF2, FZD1, GJA1, HAS2, IGF1R, IL6, KRT17, MAPK3, RAC1, TNF* | 1.95 x 10^-15^ | *ADM, AIF1, APOD, APP, AQP1, ARPC2, CDK5R1, CITED2, COL4A1, CTNNB1, CTSB, CTSH, DCN, DSE, EFNA1, ENPP2, FGF2, FOXP1, FYN, FZD1, GAP43, GLI2, GNAS, GSK3B, GSN, HAS2, ID2, IL6, KITLG, KRT17, MAFF, MAP2K4, MAP2K6, MAPK11, MEF2C, PIK3CA, PLA2G5, PLD1, PPARG, PTCH1, RAC1, RDH10, SDC2, SEMA3C, SNX10, STAT5B, TAGLN, TDRD7, TGM2, TNF, TNNC1* |
| *SELE* | Trans-membrane receptor | *ADIPOQ, ADM, BDNF, HAS2, IGF1R, IL6, KCNMA1, KCNMB1, PPARG, TNF* | 1.95 x 10^-15^ | *ADIPOQ, APP, ARPC2, BDNF, CCL11, CDK5R1, CTNNB1, CTSB, EFNA1, FGF2, FST, FYN, FZD1, GATA3, GLI2, GNAS, HAS2, HEY1, IGF1R, IL5, IL6, KITLG, MAFF, MAP2K4, MAP2K6, MAPK3, MEF2C, PLA2G2A, PLA2G5, PLD1, PPARG, RAC2, SDC2, STAT5A, STAT5B, STMN1, TAGLN, TGM2, TNF, TNNC1, TP53* |
| *PRKCI* | Kinase | *ADM, GATA3, HEY1, HEYL, ID2, IL6, TAGLN, TNF* | 1.96 x 10^-15^ | *ACADVL, ADIPOQ, APC, APP, AXIN2, BDNF, BMP4, CALB1, CCL11, CDC42, CDK5R1, COL4A3BP, CTSB, EFNA1, EHF, EXT2, FRZB, FZD1, GAP43, GATA3, GATA5, GLI2, GNAS, GSK3B, GSN, HAND1, HAS2, HEY1, HHIP, IL6, IRF6, KIT, KITLG, KRT17, MAPK11, MAPK3, MEF2C, PIK3R1, PLD1, PPARG, PTCH1, PTCH2, RAC1, STAT5A, TBX6, TGM2, TMEM100, TNF, WNT2B, WNT8B* |
| *CDK5* | Kinase | *ADM, AKT3, APOD, CTSB, FYN, GATA3, IL5, IL6, MAPK9, PIK3R1, PIK3R3, PLCG1, STAT5A, TNF, VAV1* | 1.98 x 10^-15^ | *ACADVL, ADM, AIF1, AQP1, BDNF, BMP4, CAV3, CCL11, CDC42, CDC42EP2, CDH3, CDK5R1, CLTC, COL4A1, CTSH, DCN, DPYSL2, DSE, E2F8, EHF, ENPP2, F11R, FRZB, FST, FYN, FZD1, FZD3, GLI2, GSK3B, HEY1, HEYL, HHIP, KCNMA1, KRT17, LGR4, MAFF, MAP2K4, MAP2K6, MAP2K7, MAPK11, MAPK12, MEF2C, MYH10, MYO10, NFIB, NPY1R, PAFAH1B2, PDE2A, PFN2, PLA2G2A, PLA2G5, PLD1, PLPP3, PSEN2, PTCH1, PTCH2, RAC1, RAF1, RBPJ, RDH10, S100B, S1PR1, SDC2, SEMA3C, SNAP25, ST14, STAT5B, STMN1, STMN3, TAGLN, TBX6, TCF7L2, TDRD7, TFDP1, TGFB1I1, TGM2, TNNC1, WNT11, WNT16, WNT2, WNT7A* |
| *RPSA* | Translation regulator | *ACADVL, ADIPOQ, ATL1, BDNF, CCL11, COL4A3BP, CTNNB1, EDNRA, GATA3, HAS2, IL6, PIK3R1, PLPP3, PPARG, SEMA3C, TNF, UPK1B, UPK2* | 2.02 x 10^-15^ | *ADIPOQ, AKT3, APOD, APP, AQP1, ARPC2, BCCIP, BDNF, BMP4, CDH3, CDK5R1, CSK, CTNNB1, CTSB, DCN, EFNA1, ENPP2, FGF2, FRZB, FST, FZD1, GAP43, GNAS, GSK3B, HAS2, HEY1, IGF1R, IL6, KITLG, MAFF, MAP2K6, MEF2C, PIK3R1, PPARG, PTCH1, RAC1, SEMA3C, STMN1, TAGLN, TFDP1, TGFB1I1, TGM2, TNF, TNNC1, TOLLIP, WNT16* |
| Cannabidiol | Chemical drug | *ADIPOQ, CCL11, CTSB, IL5, IL6, PPARG, TNF* | 2.05 x 10^-15^ | *ACADVL, ADIPOQ, ADM, ARPC2, AXIN2, BCCIP, BDNF, BMP4, CALB1, CDC42, COL4A1, CSK, CTSH, DCN, DOCK1, DSE, DVL2, EFNA1, EHF, ENPP2, F11R, FGF2, FRZB, FZD1, GLI2, GNA11, GSK3B, GSN, HEYL, IFT27, IGF1R, KCNJ2, KCNMA1, KIT, KITLG, KRT17, MAFF, MAP2K4, MAP2K6, MAP2K7, MAPK11, MAPK12, MAPK9, MEF2C, MYH10, NME2, PAFAH1B1, PAFAH1B2, PDE2A, PIK3R1, PLA2G2A, PLA2G5, PRKACA, PRKCB, PSEN2, PTCH1, RAC1, RAC2, RDH10, S100B, SDC2, SEMA3C, SNX10, ST14, STAT5A, STMN1, SUFU, TCF7L2, TDRD7, TMEM100, TNF, TNNC1, TP53, TRAF6, UPK1B, UPK2, VAV1, WNT16, WNT2, WNT7A* |
| *MIR-145-5p* (and other miRNAs w/seed UCCAGUU) | Mature microRNA | *APP, CTNNB1, IFT27, IGF1R, IL6, RAC1, TCF7L2, TNF, TP53* | 2.29 x 10^-15^ | *ADIPOQ, ADM, APP, ARPC2, BDNF, CAPRIN1, CCL11, CDK5R1, CITED2, COL4A1, CTNNB1, CTSB, CTSH, DCN, EDNRA, EXT1, F11R, FCER1A, FGF2, FZD1, GATA3, GLI2, GNAS, GSN, HAS2, HEY1, ID2, IGF1R, IL5, IL6, INPP5D, ITIH3, KCNQ3, KCNV1, KITLG, KRT17, LYVE1, MAPK3, MEF2C, MYO10, PADI1, PFN2, PPARG, RAC1, STAT5A, STAT5B, TAGLN, TGFB1I1, TGM2, TMEM17, TNF, TP53, WNT11* |
| *DKK1* | Growth factor | *AKT3, GSK3B, IGF1R, IL6, MAPK3, PLPP3, TNF* | 2.64 x 10^-15^ | *ADIPOQ, AIF1, APC, APP, ARFIP2, ATP6V0D1, AXIN2, BDNF, BMP4, CAV3, CDC42EP2, CHN1, CITED2, COL4A1, CPE, CSK, CTNNB1, DNM2, DSE, ELF5, EMP2, EXT1, F11R, FCER1A, FGF2, FYN, FZD3, GAP43, GATA3, GATA5, GHRL, GJA1, GNA11, GSK3B, HAS2, HEYL, HHIP, ID2, IFT27, IL5, IL6, ITIH5, KCNB2, KIF4A, KYAT3, LGR4, LIN7C, LYVE1, MAP2K4, MAPK10, MAPK11, MAPK3, MYO10, PAFAH1B1, PHACTR4, PIK3R1, PIK3R2, PIK3R3, PLA2G10, PLA2G2A, PLA2G3, PLA2G5, PLA2G6, PLCG2, PLPP3, PPARG, PRKCSH, PTCH1, PTCH2, PTPDC1, RAC1, RAC2, RAF1, RDH10, S100B, SDC2, SLC35D1, SLC9A3R1, SMARCA4, SNX10, SOS1, STAT5A, STAT5B, STK4, SUFU, TBX6, TCF7L2, TFAP2A, TNF, TNNC1, TP53, VAV1, WASF2, WNT11, WNT7A* |
| *RAB7B* | Peptidase | *BCCIP, CCL11, CDK5R1, ENPP2, FGF2, FYN, GATA3, IL5, IL6, KIT, MAP2K6, PIK3R3, PLA2G10, PPARG, PTCH1, RAF1, SOS1, STAT5B, TNF, TP53, TRAF6* | 2.77 x 10^-15^ | *ADIPOQ, AGPS, AIF1, AIMP2, APOD, ARF1, ARFIP2, ATP6V0D1, BCCIP, BDNF, CALB1, CAPRIN1, CDC42, CDC42EP1, CDH3, CHN1, CLTC, CPE, CSK, CTNNB1, CTSB, DCN, DNM2, DPYSL2, DSE, DVL2, EDA, EFNA1, EHF, ELF5, EMP2, ENPP2, ERCC3, EXT1, EXT2, FCER1A, FGF2, FOXP1, FST, FZD1, FZD3, GAP43, GHRL, GJA1, GLI2, GNAS, GSN, HAND1, HAS2, HEYL, HHIP, IFT27, IFT57, IGF1R, IL5, IL6, INPP5D, ITIH3, ITIH4, ITIH5, KCNB2, KCNMB1, KCNQ3, KIF4A, KIT, KRT17, KYAT3, LYVE1, MAP2K4, MAP2K7, MAPK10, MAPK3, MAPK9, MEF2A, MYO10, NFIB, NME2, NPY1R, PADI1, PAFAH1B1, PAFAH1B2, PDE2A, PIK3R1, PIK3R3, PLA2G12A, PLA2G3, PLA2G5, PLA2G6, PLCG1, PLCG2, PLD1, PLPP3, PRKCB, PTCH2, RAC1, RBPJ, S100B, SDC2, SEMA3C, SLC9A3R1, SNX10, SOS1, ST14, STAT5A, STAT5B, STK4, TDRD7, TFDP1, TGFB1I1, TGM2, TMEM17, TNF, TNNC1, TTC30B, TTLL1, UCHL1, UPK1A, UPK1B, UPK2, WASF2, WNT2, WNT8B, ZNF750* |
| Cadherin  *(CDH1,*  *CDH10,*  *CDH11,*  *CDH12,*  *CDH13,*  *CDH15,*  *CDH16,*  *CDH17,*  *CDH18,*  *CDH19,*  *CDH2,*  *CDH20,*  *CDH3,*  *CDH4,*  *CDH5,*  *CDH6,*  *CDH7,*  *CDH8,*  *CDH9)* | Group | *BDNF, CAV3, CDK5R1, CTNNB1, CTSB, DSE, GATA3, IL5, IL6, KRT17, MAPK3, PLA2G5, PLD1, TDRD7, TNF, TP53* | 3.03 x 10^-15^ | *ACADVL, ADIPOQ, AIMP2, AKT3, APOD, AQP1, ARPC2, AXIN2, CALB1, CAPZB, CDC42, CDC42EP2, CDH3, CITED2, CLTC, COL4A1, CTNNB1, DSE, E2F8, EDNRA, EFNA1, ENPP2, EXT2, FYN, FZD3, GAP43, GATA3, GJA1, GLI2, GNAS, HEY1, HEYL, HHIP, ID2, IFT27, IL5, INPP5D, IRF6, KCNJ2, KCNMA1, KIT, KRT17, KRT5, LMO4, MAFF, MAP2K4, MAP2K6, MAP2K7, MAPK12, MAPK9, MEF2A, MYH10, MYO10, NME2, PAFAH1B2, PDE2A, PFN2, PHACTR4, PIK3R1, PLA2G10, PLA2G5, PLCG1, PPARG, PSEN2, PTCH1, PTCH2, PTPDC1, RAC1, RAC2, RAF1, RDH10, SDC2, SLC35D1, SLC9A3R1, SMARCA4, ST14, STAT5A, STAT5B, STMN3, TCF7L2, TDRD7, TGFB1I1, TNF, TP53, TRAF6, WNT11, WNT2, WNT7A, WNT8B* |
| *CYP2A6* (includes others) | Enzyme | *CTNNB1, FRZB, FZD1, FZD6, HEY1, IL5, IL6, KITLG, TNF, TNNC1, TP53* | 3.12 x 10^-15^ | *AIMP2, AKT3, APOD, ARF1, ARPC2, AXIN2, BDNF, CAPZB, CHN1, CLTC, COL4A1, COL4A3BP, CTNNB1, DCN, DVL2, EDA, EDNRA, EMP2, ENPP2, EXT2, FGF2, FRZB, FYN, FZD3, GATA3, GHRL, GJA1, GNA11, GSK3B, GSN, HAND1, HEY1, HHIP, IFT27, IGF1R, IL6, ITIH4, ITIH5, KCNB2, KCNJ2, KITLG, KRT5, LMO4, LYVE1, MAFF, MAP2K4, MAP2K7, MAPK10, MAPK11, MAPK3, MYH10, NFIB, NME2, PADI1, PAFAH1B1, PFN2, PIK3R1, PLA2G5, PLCG1, PLPP3, PRKCB, PSEN2, PTCH1, PTCH2, RAB13, RAC2, RAF1, S100B, S1PR1, SEMA3C, SLC9A3R1, SNAP25, STAT5B, STMN1, TAGLN, TCF7L2, TGM2, TNF, TP53, TRAF6, WNT11, WNT16, WNT8B* |
| S-adenosylhomocysteine | Chemical - endogenous mammalian | *FGF2, GJA1, IL6, TNF, TP53* | 3.3 x 10^-15^ | *AIF1, APC, AQP1, ARFIP2, ATP6V0D1, BCCIP, BDNF, BMP4, CAV3, CDC42, CDC42EP2, CITED2, CPE, CTSB, DNM2, DPYSL2, EFNA1, ENPP2, FGF2, FST, FZD3, GATA3, GJA1, GLI2, GSK3B, GSN, HAS2, HEY1, IL5, INPP5D, IRF6, KCNMA1, KIT, KRT17, MAFF, MAP2K6, MAP2K7, MAPK11, MAPK12, MYH10, MYO10, PFN2, PRKCA, PTCH1, PTCH2, RAC1, RBPJ, RDH10, S100B, SEMA3C, SOS1, STAT5A, STAT5B, STK4, SUFU, TAGLN, TCF7L2, TGM2, TP53, TRAF6, WASF2* |
| Naproxen | Chemical drug | *FGF2, GATA3, GJA1, IL5, IRF6, KCNJ2, KRT17, MEF2C, PLA2G2A, TAGLN, TNNC1* | 3.85 x 10^-15^ | *ADIPOQ, ADM, AQP1, AXIN2, BDNF, BMP4, CALB1, CAV3, CCL11, COL4A1, CTNNB1, CTSB, DCN, EDNRA, EFNA1, EHF, ENPP2, FGF2, FST, FZD3, GATA3, GJA1, GLI2, GNAS, HAS2, HEY1, HHIP, ID2, IGF1R, KIT, KITLG, KRT17, MAPK9, PLD1, PTCH1, PTCH2, RAC1, RBPJ, RDH10, SDC2, SEMA3C, SNX10, STAT5A, TGM2, WNT11* |
| *SERPINA1* | Other | *APC, GATA3, GATA5, IL5, KITLG, MEF2A, PRKCB, S1PR1, SS18L1, TBX6, TNF* | 3.96 x 10^-15^ | *AIF1, APP, AXIN2, BCCIP, CALB1, CAV3, CDC42, CDC42EP1, CDH3, CITED2, COL4A1, CSK, CTSB, DCN, DOCK1, DPYSL2, DVL2, EFNA1, EHF, ENPP2, FGF2, FST, FYN, FZD3, GAP43, GNAS, GSK3B, HAS2, HEY1, ID2, IGF1R, IL5, KIT, KRT17, KRT5, MAFF, MAP2K6, MAPK3, PIK3R1, PLA2G5, PLD1, PRKCA, PTCH2, RAC2, RBPJ, S100B, S1PR1, SEMA3C, STAT5A, TFDP1, TGFB1I1, TGM2, TNF, TRAF6, VAV1* |
| *CRLF1* | Other | *ADM, APP, BDNF, CITED2, CTNNB1, IL6, KIT, PLA2G12B, PLA2G3, PPARG, PRKCA, TP53* | 4.01 x 10^-15^ | *AIF1, AIMP2, APC, AQP1, ARF1, ARPC2, BCCIP, CAPRIN1, CAPZB, CCL11, CDC42, CDC42EP1, CDC42EP2, CHN1, CSK, CTSH, DVL2, E2F8, EDA, EMP2, ENPP2, FOXP1, FST, FZD3, GAP43, GATA3, GJA1, HAS2, HEY1, HEYL, HHIP, ID2, IGF1R, IL5, IL6, INPP5D, IRF6, ITIH4, ITIH5, KCNJ2, KCNMB1, KCNQ3, KITLG, LGR4, LMO4, LYVE1, MAFF, MAP2K4, MEF2A, MEF2C, MYH10, MYO10, NFIB, PADI1, PAFAH1B1, PDE2A, PIK3R3, PLA2G2A, PLA2G5, PLA2G6, PLCG1, PPARG, PRKACA, PRKCA, PSEN2, PTCH2, RAC2, RBPJ, RDH10, S100B, S1PR1, SMARCA4, SNAP25, SNX10, SOS1, STAT5B, STMN3, SUFU, TAGLN, TFAP2A, TFDP1, TGFB1I1, TGM3, TRAF6, UPK1A, UPK1B, UPK2, WNT11* |
| *CLC* | Enzyme | *ADIPOQ, APP, ATP6V0D1, BDNF, CCL11, FCER1A, FST, GAP43, GJA1, GNAS, HAS2, HHIP, ID2, IGF1R, IL5, IL6, KITLG, MAPK3, NPY1R, PRKACA, SNAP25, TNF, TP53* | 4.09 x 10^-15^ | *AIF1, AIMP2, APC, AQP1, ARF1, ARPC2, BCCIP, CAPRIN1, CAPZB, CCL11, CDC42, CDC42EP1, CDC42EP2, CHN1, CSK, CTSH, DVL2, E2F8, EDA, EMP2, ENPP2, FOXP1, FST, FZD3, GAP43, GATA3, GJA1, HAS2, HEY1, HEYL, HHIP, ID2, IGF1R, IL5, IL6, INPP5D, IRF6, ITIH4, ITIH5, KCNJ2, KCNMB1, KCNQ3, KITLG, LGR4, LMO4, LYVE1, MAFF, MAP2K4, MEF2A, MEF2C, MYH10, MYO10, NFIB, PADI1, PAFAH1B1, PDE2A, PIK3R3, PLA2G2A, PLA2G5, PLA2G6, PLCG1, PPARG, PRKACA, PRKCA, PSEN2, PTCH2, RAC2, RBPJ, RDH10, S100B, S1PR1, SMARCA4, SNAP25, SNX10, SOS1, STAT5B, STMN3, SUFU, TAGLN, TFAP2A, TFDP1, TGFB1I1, TGM3, TRAF6, UPK1A, UPK1B, UPK2, WNT11* |
| Alpha catenin  *(CTNNA1, CTNNA2)* | Group | *CDK5R1, CTNNB1, GAP43, HAND1, HAS2, ID2, IL6, MAFF, PLPP3, SEMA3C, TNF, TP53* | 4.2 x 10^-15^ | *ACADVL, AIF1, AIMP2, APC, APP, ARF1, ARFIP2, ARPC2, ATP6V0D1, AXIN2, BDNF, BMP4, CALB1, CAPRIN1, CAV3, CCL11, CDC42, CDC42EP1, CDC42EP2, CHN1, CITED2, COL4A1, COL4A3BP, CPE, CTNNB1, CTSB, DNM2, DPYSL2, DSE, DVL2, EDA, EFNA1, EHF, ELF5, EXT2, FCER1A, FGF2, FLOT1, FOXP1, FST, FZD3, FZD6, GAP43, GATA3, GJA1, GLI2, GNA11, GNAS, GSN, HAS2, HHIP, ID2, IFT27, IGF1R, IL5, IL6, ITIH3, ITIH4, ITIH5, KCNB2, KCNJ2, KCNMA1, KCNMB1, KCNQ3, KIF4A, KIT, KITLG, KRT17, LGR4, MAFF, MAP2K7, MAPK10, MAPK9, MEF2A, MYO10, NME2, PADI1, PAFAH1B1, PAFAH1B2, PDE2A, PHACTR4, PLA2G10, PLA2G12A, PLA2G3, PLA2G5, PLCG1, PLCG2, PLD1, PPARG, PRKACA, PRKCB, PTCH1, PTCH2, PTPDC1, RAC2, RBPJ, S100B, SCN1B, SDC2, SLC35D1, SLC9A3R1, SMARCA4, SOS1, ST14, STAT5A, STAT5B, STK4, SUFU, TGM2, TMEM17, TNF, UCHL1, WASF2, WNT11, WNT2, WNT2B, ZNF750* |
| Aspirin | Chemical drug | *ADM, APP, ATP6V0D1, CCL11, FST, GNAS, IL5, IL6, S1PR1, ST14, TNF* | 4.21 x 10^-15^ | *ACADVL, AIF1, AKT3, APOD, ARPC2, AXIN2, BCCIP, BMP4, CALB1, CDC42, CDH3, CDK5R1, CITED2, COL4A1, COL4A3BP, CSK, DCN, DSE, DVL2, EDNRA, EFNA1, EHF, ENPP2, FRZB, FST, FYN, FZD3, GHRL, GSK3B, HEY1, HHIP, ID2, IFT27, IL6, IRF6, ITIH4, KCNJ2, KIT, KITLG, KYAT3, MAFF, MAP2K4, MAP2K6, MAPK9, PIK3R1, PLA2G2A, PLA2G5, PLA2G6, PLPP3, PRKCA, PTCH1, PTCH2, RBPJ, RDH10, SEMA3C, STAT5A, STMN1, TCF7L2, TDRD7, TFDP1, TGFB1I1, TNF, TNNC1, TP53, WNT11, WNT16, WNT2* |
| *CABIN1/ HDAC1/2/ MEF2D/ MITR/ SIN3* | Complex | *BMP4, CALB1, FGF2, FST, FZD1, HAS2, HEY1, ID2, MEF2C, PPARG, TAGLN, TGM2* | 4.58 x 10^-15^ | *ACADVL, ADIPOQ, APOD, AQP1, AXIN2, BDNF, BMP4, CAV3, CDC42, CDH3, CDK5R1, CITED2, COL4A3BP, CTSB, CTSH, EFNA1, ENPP2, FRZB, FST, FYN, GAP43, GATA3, GLI2, GSK3B, GSN, HAS2, ID2, IL6, IRF6, KCNMA1, KCNQ3, KRT17, MAP2K6, MAP2K7, MAPK12, MAPK3, MYH10, PAFAH1B2, PDE2A, PIK3CA, PIK3R3, PLD1, PRKCA, PSEN2, RAC2, RBPJ, RDH10, SLC35D1, ST14, STMN1, SUFU, TCF7L2, TFDP1, TGFB1I1, TNNC1, UPK1A, UPK1B, UPK2, WNT16, WNT2, WNT7A* |
| *CBP/ EP300* | Group | *APOD, AXIN2, BDNF, BMP4, CTNNB1, CTSB, FGF2, GJA1, GSN, PIK3R1, PRKCB* | 4.7 x 10^-15^ | *ADIPOQ, AKT3, APC, AQP1, ARF1, ARFIP2, ATP6V0D1, AXIN2, BCCIP, BDNF, BMP4, CALB1, CAV3, CCL11, CDC42, CDC42EP2, CDH3, CITED2, COL4A1, COL4A3BP, CPE, DNM2, EFNA1, ENPP2, FGF2, FZD3, GAP43, GJA1, GLI2, GNA11, GNAS, GSK3B, GSN, HAS2, HEY1, HHIP, ID2, INPP5D, KCNB2, KCNMA1, KRT17, MAFF, MAP2K6, MAP2K7, MAPK10, MAPK11, MEF2C, MYH10, PAFAH1B1, PFN2, PLA2G2A, PLD1, PRKCB, PTCH1, PTCH2, RAC1, S1PR1, SDC2, SLC9A3R1, SOS1, STAT5A, STAT5B, STK4, SUFU, TAGLN, TCF7L2, TGM2, TNF, TRAF6, WASF2, WNT11* |
| Methyl-methanesulfonate | Chemical toxicant | *ADIPOQ, IL6, TNF* | 4.98 x 10^-15^ | *AKT3, APC, APOD, APP, AQP1, BDNF, BMP4, CALB1, CAV3, CCL11, CDK5R1, CITED2, CTNNB1, EFNA1, EHF, FGF2, FRZB, FST, GAP43, GNAS, GSK3B, HAS2, IGF1R, IL6, KIT, KITLG, KRT17, MAP2K6, MAPK3, MAPK9, MEF2C, PLD1, PTCH1, RAC1, RBPJ, SDC2, ST14, STAT5A, STMN1, TAGLN, TGM2, TNF, TNNC1, TP53, WNT16, WNT7A* |
| *MARK3* | Kinase | *AXIN2, CTNNB1, DVL2* | 5.04 x 10^-15^ | *ADIPOQ, ADM, APC, APP, AQP1, ARFIP2, ARPC2, ATP6V0D1, AXIN2, BMP4, CALB1, CDC42, CDC42EP1, CDC42EP2, CDH3, CHN1, COL4A1, CPE, CSK, CTNNB1, CTSH, DCN, DNM2, DSE, EFNA1, EHF, ENPP2, FGF2, FZD1, FZD3, GAP43, GATA3, GJA1, GLI2, GSN, HEY1, HHIP, ID2, IL6, KCNMA1, KIF4A, MAFF, MAP2K6, MAP2K7, MAPK12, MAPK3, MYH10, PFN2, PIK3R1, PRKACA, PSEN2, PTCH1, PTCH2, RAC1, RBPJ, RDH10, SDC2, SOS1, STAT5B, STK4, SUFU, TAGLN, TCF7L2, TDRD7, TFAP2A, TFDP1, TGFB1I1, TNNC1, TP53, WASF2, WNT11* |
| *TRPS1* | Transcription regulator | *IL6, TNF, TOLLIP* | 5.05 x 10^-15^ | *ADIPOQ, ADM, APP, ARPC2, BDNF, CAPRIN1, CCL11, CDK5R1, CITED2, COL4A1, CTNNB1, CTSB, CTSH, DCN, EDNRA, ENPP6, EXT1, FCER1A, FGF2, FZD1, GATA3, GLI2, GNAS, GSN, HAS2, HEY1, ID2, IL5, IL6, INPP5D, ITIH3, KCNMA1, KCNQ3, KCNV1, KITLG, KRT17, LYVE1, MAPK3, MEF2C, MYO10, PFN2, PPARG, RAC1, STAT5A, STAT5B, TAGLN, TGFB1I1, TGM2, TMEM17, TNF, TP53, WNT11* |
| *HMGA1* | Transcription regulator | *IGF1R, KCNMA1, KCNMB1* | 5.25 x 10^-15^ | *ACADVL, ADIPOQ, AIF1, APP, ARPC2, AXIN2, CAV3, CCL11, CDC42, CDC42EP2, CDK5R1, CITED2, COL4A1, CPE, CSK, CTSH, EFNA1, ENPP2, ERCC3, F11R, FYN, GLI2, GSK3B, GSN, HAND1, HAS2, HEY1, IGF1R, IL5, IL6, KCNMA1, KIT, KITLG, MAFF, MAP2K6, MAP2K7, MAPK12, MYH10, PAFAH1B2, PDE2A, PIK3CA, PIK3R3, PPARG, PRKCA, PSEN2, PTCH1, RAC2, RBPJ, RDH10, SLC9A3R1, ST14, STMN1, SUFU, TAGLN, TCF7L2, TFDP1, TGFB1I1, UPK1A, UPK1B, UPK2, WNT2, WNT7A* |
| *PLK1* | Kinase | *BDNF, IL6, TNF* | 5.27 x 10^-15^ | *ACADVL, ADIPOQ, ADM, AKT3, APC, APP, AQP1, ARFIP2, ATP6V0D1, BCCIP, BDNF, BMP4, CALB1, CAV3, CCL11, CDC42, CDC42EP2, CDH3, CITED2, COL4A1, CPE, CSK, CTNNB1, CTSB, CTSH, DCN, DNM2, EHF, FCER1A, FGF2, FYN, FZD3, GATA3, GJA1, GLI2, GNAS, GSK3B, GSN, HHIP, IFT27, KCNMA1, KIT, KRT17, MAP2K7, MAPK11, PAFAH1B2, PDE2A, PFN2, PIK3CA, PIK3R1, PIK3R3, PLCG2, PLD1, PRKCA, PSEN2, PTCH1, PTCH2, RAC2, RBPJ, S1PR1, SDC2, SMARCA4, SOS1, ST14, STAT5B, STK4, STMN1, TAGLN, TDRD7, TFDP1, TGFB1I1, TGM2, UPK1A, UPK1B, UPK2, WASF2, WNT11, WNT2, WNT7A* |
| WWOX | Enzyme | *APP, IL6, TNF* | 5.36 x 10^-15^ | *AIF1, ARPC2, AXIN2, BDNF, CALB1, CAV3, CCL11, CDC42EP2, CDK5R1, CITED2, COL4A3BP, CTNNB1, EFNA1, EHF, ERCC3, FGF2, FYN, GAP43, GJA1, GLI2, GNAS, GSK3B, HAS2, HEY1, ID2, IFT27, IGF1R, IL5, IL6, KIT, KRT17, MAFF, MAPK11, MAPK3, PIK3R3, PLD1, PPARG, RAC1, RBPJ, STAT5A, STMN1, TCF7L2, TGM2, TP53, WNT2, WNT7A* |
| HSPD1 | Enzyme | *DOCK1, GSK3B, MAP2K6, PIK3R1, PRKACA, PRKCB, SOS1, TP53* | 5.67 x 10^-15^ | *ADM, APOD, APP, AQP1, ARPC2, BDNF, BMP4, CALB1, CAV3, CCL11, CDC42, CDK5R1, COL4A1, COL4A3BP, DCN, EFNA1, EHF, ENPP2, FGF2, FST, GAP43, GATA3, GLI2, GNAS, HAS2, IGF1R, IL6, IRF6, KCNJ2, KIT, KITLG, KRT17, KRT5, MAFF, MAP2K4, MAP2K6, MAPK3, MEF2C, PLA2G5, PLA2G6, PLD1, PRKCA, PTCH1, RAC2, RBPJ, S1PR1, SDC2, SEMA3C, SNX10, STMN1, TFDP1, TGM2, TNF, TNNC1* |
| Beta-estradiol | Chemical - endogenous mammalian | *GATA3, IL6, ITIH5, LMO4, NFIB, PPARG, RAF1, TGM2, TP53* | 5.82 x 10^-15^ | *ACADVL, ADIPOQ, ADM, APC, APOD, APP, BDNF, BLOC1S6, BMP4, CALB1, CDC42, CDK5R1, CITED2, CTNNB1, CTSB, CTSH, DCN, EFNA1, ELF5, ENPP2, EXT2, FGF2, FLOT1, FST, GJA1, GLI2, GSK3B, HAND1, IGF1R, IL6, IRF6, ITIH4, KIT, KITLG, MAP2K6, MEF2A, NPY1R, PADI1, PIK3R1, PIK3R2, PIK3R3, PLA2G10, PPARG, PRKCB, PSEN2, RAC1, RDH10, S1PR1, SDC2, SEMA3C, SLC9A3R1, SNAP25, SOS1, TNF, TNNC1, WNT11, WNT7A* |
| *GADD45A* | Other | *AIF1, CCL11, FGF2, GATA3, HEY1, IL5, IL6, KCNMA1, STMN1, TNF, TRAF6* | 5.85 x 10^-15^ | *ACADVL, ADIPOQ, ADM, APOD, AQP1, AXIN2, BDNF, BMP4, CAPRIN1, CAV3, CDH3, CDK5R1, COL4A1, CTSH, EFNA1, ENPP2, ERCC3, FGF2, FRZB, FST, FYN, GAP43, GATA3, GJA1, GLI2, GSK3B, GSN, HEY1, IFT27, IL6, KCNMA1, KIT, KRT17, MAP2K4, MAP2K6, MAP2K7, MAPK12, MAPK3, MAPK9, MEF2C, MYH10, PAFAH1B2, PDE2A, PRKCA, PSEN2, PTCH1, RDH10, S1PR1, ST14, STAT5A, STAT5B, STMN1, TAGLN, TCF7L2, TFDP1, TGFB1I1, TGM2, UCHL1, WNT16, WNT2, WNT7A* |
| Nocodazole | Chemical reagent | *AIF1, GAP43, GJA1, IL5, IL6, PPARG, TNF* | 5.88 x 10^-15^ | *ACADVL, AIF1, AKT3, APOD, APP, AQP1, ARPC2, AXIN2, BCCIP, BMP4, CALB1, CDC42EP2, CDH3, CDK5R1, CSK, EFNA1, EHF, ERCC3, FZD1, GAP43, GATA3, GLI2, GNAS, GSK3B, KCNMA1, KIT, KITLG, KRT17, MAFF, MAP2K6, MAP2K7, MAPK11, MAPK12, MAPK3, MEF2A, MEF2C, MYH10, PAFAH1B2, PDE2A, PIK3R1, PIK3R3, PLA2G2A, PLD1, PLPP3, PPARG, PRKCA, PRKCB, PSEN2, PTCH1, RAC1, RAF1, RBPJ, SDC2, SLC35D1, SNAP25, SNX10, ST14, STAT5A, STK4, STMN1, TCF7L2, TFDP1, TGFB1I1, TGM2, TNNC1, TP53, WNT2, WNT7A* |
| *FCGR2A* | Trans-membrane receptor | *AQP1, GAP43, IL5, IL6, PPARG, TNF* | 6.12 x 10^-15^ | *ADIPOQ, ADM, APP, ARPC2, BDNF, CALB1, CDK5R1, CITED2, CTNNB1, DSE, EFNA1, EHF, FGF2, FOXP1, FYN, FZD1, GAP43, GJA1, GLI2, GNAS, GSN, HAS2, HEY1, ID2, IGF1R, IL5, IL6, IRF6, KCNJ2, KIT, KITLG, MAFF, MAP2K4, MAP2K6, MEF2C, PIK3CA, PIK3R1, PLA2G5, PLA2G6, PLCG1, PLD1, PRKCA, RBPJ, SDC2, STAT5A, STAT5B, STMN1, TAGLN, TDRD7, TGM2, TNF, TNNC1* |
| *KlRA7* (includes others) | Trans-membrane receptor | *ADIPOQ, APP, AQP1, FGF2, GJA1, KIT* | 6.18 x 10^-15^ | *BDNF, BMP4, CAV3, CCL11, CITED2, CTSB, FGF2, FOXP1, FST, FZD1, GATA3, GJA1, GLI2, HAS2, HEYL, IGF1R, IL6, MAP2K4, MAPK3, MEF2C, NME2, PAFAH1B1, PIK3CA, PLA2G5, PLD1, RAC1, S100B, STMN1, SUFU, TGM2, TNF, TP53, UPK1B, UPK2* |
| Aleplasinin | Chemical drug | *APOD, APP, BDNF, CALB1, CCL11, EDNRA, FST, FYN, GJA1, IL5, IL6, LYVE1, MYH10, PRKCA, STAT5A, TAGLN, TNF, TP53* | 6.23 x 10^-15^ | *AIF1, AKT3, APP, ARF1, ARPC2, AXIN2, BDNF, BMP4, CALB1, CDK5R1, CLTC, COL4A1, DNM2, DOCK1, EFNA1, EHF, FRZB, FST, FYN, FZD1, FZD3, GATA3, GLI2, GNAS, GSK3B, HAS2, HEY1, HHIP, ID2, IGF1R, KIT, KITLG, KRT17, MAFF, MAPK11, MYH10, PLA2G5, PLD1, PTCH1, PTCH2, RAC1, RAC2, RBPJ, S1PR1, SDC2, SNAP25, STAT5A, STMN1, TAGLN, TGM2, TNF, TNNC1, VAV1, WNT11, WNT16* |
| *ADRA1* | Group | *ADIPOQ, APP, CTNNB1, CTSB, ENPP2, FGF2, IGF1R, IL6, KIT, KITLG, LGR4, PPARG, PRKCSH, RAC1, STMN1, TAGLN, TGM2, TNF, TP53* | 7.17 x 10^-15^ | *ADM, AIF1, APOD, APP, AQP1, ARPC2, BDNF, BMP4, CCL11, CDK5R1, CTNNB1, CTSB, FGF2, FZD1, GAP43, GJA1, GLI2, HAS2, HEY1, HEYL, IL6, MAFF, MAPK3, MEF2A, NME2, PAFAH1B1, PLD1, PPARG, PTCH1, RAC1, RDH10, S100B, S1PR1, SUFU, TGM2, TNF, TP53, UPK1B, UPK2* |
| *UBE4B* | Enzyme | *APP, CTNNB1, GJA1, IL6, TP53* | 7.18 x 10^-15^ | *ACADVL, ADM, AKT3, APP, AXIN2, CDC42, CDH3, CITED2, CTNNB1, CTSB, CTSH, EFNA1, ENPP2, FST, FYN, GSK3B, GSN, HAS2, ID2, IGF1R, IL5, IL6, KCNMA1, MAP2K6, MAP2K7, MAPK12, MYH10, PAFAH1B2, PDE2A, PIK3CA, PIK3R3, PPARG, PRKCA, PSEN2, PTCH1, RAC2, RBPJ, ST14, STMN1, SUFU, TAGLN, TCF7L2, TFDP1, TGFB1I1, TGM2, TNF, TP53, UPK1A, UPK1B, UPK2, WNT2, WNT7A* |
| *MTA1* | Transcription regulator | *BDNF, IL6, NPY1R, TNF, TP53* | 1.06 x 10^-15^ | *ADIPOQ, APC, APP, AQP1, ARFIP2, ARPC2, ATP6V0D1, AXIN2, BCCIP, BDNF, CAV3, CCL11, CDC42, CDC42EP2, CDK5R1, COL4A1, CPE, CTNNB1, CTSB, DNM2, EFNA1, ENPP2, FGF2, FZD1, FZD3, GAP43, GATA3, GLI2, GNAS, GSK3B, GSN, HAS2, HEY1, HHIP, ID2, IFT27, IL6, KCNMA1, MAFF, MAP2K6, MAP2K7, MAPK11, MAPK12, MAPK3, MYH10, PFN2, PIK3R1, PPARG, SDC2, SEMA3C, SOS1, STAT5A, STAT5B, STK4, SUFU, WASF2, WNT11* |
| *NCOR2* | Transcription regulator | *GNAS, IL6, MAPK3, PLA2G6, TNF* | 1.24 x 10^-15^ | *ADIPOQ, APC, APP, ARFIP2, ATP6V0D1, BCCIP, BDNF, CALB1, CAV3, CCL11, CDC42, CDC42EP2, CPE, CTSB, DNM2, DPYSL2, EDNRA, EFNA1, ELF5, F11R, FGF2, FLOT1, FST, FZD1, FZD6, GAP43, GATA3, GLI2, GNAS, HAS2, IRF6, ITIH3, KCNJ2, KCNMA1, KIT, KITLG, KRT17, MAFF, MAP2K6, MAP2K7, MAPK11, MAPK12, MAPK3, MEF2C, MYH10, MYO10, NME2, NPY1R, PLA2G2A, PLCG1, PRKCA, PTCH2, RDH10, SCN1B, SEMA3C, SLC9A3R1, SOS1, STK4, SUFU, TCF7L2, TFAP2A, TMEM17, TRAF6, UCHL1, WASF2, WNT16, WNT2, WNT2B, WNT7A, WNT8B* |
| *IKB*  *(IKBKG,*  *NFKBIA,*  *NFKBIB,*  *NFKBID,*  *NFKBIE)* | Group | *GATA3, IGF1R, IL5, IL6, TNF* | 1.25 x 10^-15^ | *APP, BCCIP, BDNF, CALB1, CAV3, CCL11, CDH3, CITED2, COL4A3BP, CSK, CTSB, DCN, EFNA1, EHF, ENPP2, FGF2, FST, GAP43, GJA1, GLI2, GNAS, HAS2, IGF1R, IL5, IL6, KIT, KRT17, PIK3R1, PLD1, PPARG, PRKACA, RBPJ, SEMA3C, STAT5A, TFDP1, TGFB1I1, TGM2, TNF, TP53, TRAF6* |
| Linsidomine | Chemical drug | *BDNF, ENPP2, KIT, KRT5, S1PR1, SEMA3C, TAGLN* | 1.28 x 10^-15^ | *AIF1, APOD, APP, AQP1, ARPC2, BDNF, BMP4, CAV3, CCL11, CDH3, CDK5R1, CITED2, CTSB, DCN, EFNA1, FGF2, FST, FYN, FZD1, GJA1, GNAS, GSK3B, HAS2, HEY1, IFT27, IGF1R, KCNB2, KITLG, MAFF, MAP2K6, MAPK10, MAPK11, MAPK3, MEF2C, PPARG, PTCH1, RAC1, RAC2, TAGLN, TCF7L2, TGM2, TNF* |
| *CYCLIN A*  *(CCNA1, CCNA2)* | Group | *ADIPOQ, BDNF, CAV3, CCL11, FGF2, FST, GJA1, IL5, IL6, IRF6, MEF2C, PPARG, SDC2, TNF, TNNC1, TP53* | 1.39 x 10^-15^ | *ACADVL, AKT3, APP, BDNF, BMP4, CDH3, CDK5R1, CITED2, COL4A3BP, CTNNB1, CTSB, CTSH, DCN, DPYSL2, EDA, EFNA1, ELF5, ENPP2, F11R, FST, FYN, FZD1, FZD3, GAP43, GJA1, GLI2, GNAS, GSN, HAS2, HHIP, IGF1R, IRF6, KCNMA1, KCNQ3, KIT, LMO4, MAP2K6, MAP2K7, MAPK3, MEF2C, MYH10, MYO10, PADI1, PAFAH1B2, PDE2A, PFN2, PIK3R1, PRKCA, PSEN2, PTCH1, PTCH2, RAC2, RBPJ, SDC2, SLC9A3R1, SNAP25, ST14, STAT5A, STAT5B, STMN1, TCF7L2, TFDP1, TGFB1I1, TGM2, TNNC1, WNT11, WNT2, WNT7A* |
| *PEPCK*  *(PCK1, PCK2)* | Group | *APP, IL6, TNF, WNT7A* | 1.42 x 10^-14^ | *ACADVL, ADM, AKT3, APP, AQP1, ARPC2, AXIN2, CCL11, CDC42EP1, CDH3, CDK5R1, CITED2, CLTC, COL4A1, CTSH, DVL2, ENPP2, FGF2, FST, FYN, GJA1, GLI2, GSK3B, GSN, HAS2, HEY1, ID2, IGF1R, IL5, IL6, INPP5D, ITIH5, KCNMA1, KYAT3, LMO4, MAFF, MAP2K6, MAP2K7, MAPK12, MAPK3, MEF2C, MYH10, NFIB, PAFAH1B2, PDE2A, PLA2G3, PSEN2, PTCH1, RBPJ, RDH10, S1PR1, SEMA3C, ST14, STAT5A, STAT5B, STMN1, TAGLN, TCF7L2, TFDP1, TGFB1I1, TNF, TNNC1, TP53, TRAF6, WNT16, WNT2, WNT7A* |
| *VRK2* | Kinase | *ATP6V0D1, COL4A1, FGF2, IRF6, KRT17, LYVE1, MEF2C, SEMA3C, TAGLN, TNNC1, WNT2* | 1.74 x 10^-14^ | *ACADVL, ADIPOQ, AKT3, APOD, AQP1, ARPC2, AXIN2, BDNF, BMP4, CAV3, CCL11, CDH3, CDK5R1, CITED2, COL4A1, CTNNB1, CTSB, CTSH, EFNA1, FGF2, FST, FYN, FZD1, GAP43, GATA3, GSK3B, GSN, HEY1, ID2, IL6, KCNMA1, LMO4, MAFF, MAP2K6, MAP2K7, MAPK12, MAPK3, MYH10, PAFAH1B2, PDE2A, PLA2G2A, PLD1, PPARG, PRKCA, PSEN2, RAC1, RBPJ, ST14, STAT5A, STMN1, TCF7L2, TFDP1, TGFB1I1, TNF, WNT2, WNT7A* |
| *MNAT1* | Other | *BMP4, DPYSL2, HAS2, IL6, PPARG, TAGLN, TP53* | 1.74 x 10^-14^ | *APC, AQP1, ARFIP2, ATP6V0D1, BCCIP, BMP4, CDC42, CDC42EP2, CDK5R1, COL4A3BP, CPE, CTNNB1, DNM2, E2F8, EDNRA, EFNA1, ENPP2, EXT2, FGF2, FRZB, FST, GJA1, GNAS, GSN, HAND1, IL5, IRF6, KCNMA1, KITLG, KRT17, MAFF, MAP2K6, MAP2K7, MAPK11, MAPK12, MAPK3, MEF2A, MYH10, PFN2, PLA2G2A, PSEN2, PTCH1, PTCH2, SEMA3C, SOS1, STAT5A, STAT5B, STK4, SUFU, TCF7L2, TDRD7, TFAP2A, TGM2, TNF, TNNC1, TP53, WASF2, WNT7A, WNT8B* |
| 1-chloro-2-(2,2,2-trichloro-1-(4-chlorophenylethyl)  benzene | Chemical toxicant | *ADIPOQ, FGF2, IL6, TNF, TP53* | 1.81 x 10^-14^ | *ADIPOQ, ADM, AKT3, APC, APP, AQP1, ARFIP2, ARPC2, ATP6V0D1, BCCIP, BMP4, CAV3, CCL11, CDC42, CDC42EP2, CDH3, CDK5R1, COL4A1, CPE, DNM2, EFNA1, ENPP2, F11R, FGF2, FST, GAP43, GHRL, GLI2, GNAS, GSN, HAS2, HEY1, IL5, IL6, KCNMA1, KRT17, MAFF, MAP2K6, MAP2K7, MAPK11, MAPK12, MYH10, PFN2, PLD1, PPARG, PTCH1, PTCH2, RDH10, SEMA3C, SLC9A3R1, SOS1, STAT5A, STAT5B, STK4, SUFU, TCF7L2, TGM2, WASF2* |
| *CSNK1D* | Kinase | *ADIPOQ, IL6, PPARG, TNF, TP53* | 1.92 x 10^-14^ | *ACADVL, AIF1, AKT3, APP, BDNF, CDC42, CITED2, COL4A1, CTSB, CTSH, EFNA1, FGF2, FYN, FZD3, GAP43, GATA3, GLI2, GSK3B, GSN, HAND1, HAS2, HHIP, IL5, IL6, KCNMA1, KCNQ3, LMO4, MAP2K6, MAP2K7, MAPK11, MAPK12, MAPK3, MYH10, PAFAH1B2, PDE2A, PLA2G10, PLA2G2A, PLD1, PRKCA, PSEN2, PTCH1, PTCH2, RBPJ, S100B, SDC2, SNAP25, ST14, STAT5A, STMN1, TAGLN, TCF7L2, TFDP1, TGFB1I1, TGM2, UCHL1, WNT11, WNT2, WNT7A* |
| *HDAC1/2* | Group | *ADM, GATA3, IL5, IL6, IRF6, KIT, KRT17, MYO10, PPARG, STAT5A, TNF, WNT11* | 1.95 x 10^-14^ | *ACADVL, ADM, AKT3, APP, AXIN2, CCL11, CDC42, CDH3, CITED2, COL4A3BP, CTNNB1, CTSB, CTSH, EFNA1, ENPP2, FGF2, FST, FYN, GLI2, GSK3B, GSN, HAS2, ID2, KCNMA1, MAP2K6, MAP2K7, MAPK12, MEF2C, MYH10, PAFAH1B2, PDE2A, PIK3CA, PIK3R3, PLD1, PPARG, PRKCA, PSEN2, PTCH1, RAC2, RBPJ, SLC35D1, ST14, STMN1, SUFU, TAGLN, TCF7L2, TFDP1, TGFB1I1, TP53, UPK1A, UPK1B, UPK2, WNT2, WNT7A* |
| *MIR-199a-5p* (and other miRNAs w/seed CCAGUGU) | Mature microRNA | *BMP4, CDH3, EHF, FCER1A, IL6, KIT, RBPJ, STAT5B* | 2.21 x 10^-14^ | *ACADVL, AIF1, AKT3, APP, AXIN2, BDNF, CDH3, CITED2, COL4A1, CTNNB1, CTSB, CTSH, DCN, EFNA1, ENPP2, FYN, GAP43, GATA3, GJA1, GSK3B, GSN, ID2, IL5, IL6, KCNMA1, KRT17, LIN7C, LMO4, MAP2K6, MAP2K7, MAPK12, MYH10, PAFAH1B2, PDE2A, PLA2G2A, PLD1, PPARG, PRKCA, PSEN2, PTCH1, RBPJ, ST14, STAT5A, STMN1, TAGLN, TCF7L2, TFDP1, TGFB1I1, TGM2, TNF, TNNC1, WNT2, WNT7A* |
| *SOX2* | Transcription regulator | *CCL11, FYN, GATA3, GNAS, IL5, IL6, KIT, MAPK3, PLD1, PRKACA, RAC2, RBPJ, S1PR1, SOS1, TNF, VDAC3* | 2.26 x 10^-14^ | *APC, AXIN2, BMP4, CITED2, CTNNB1, EXT2, FRZB, FST, GATA3, GJA1, GLI2, GSK3B, HAND1, HEY1, ID2, IRF6, KITLG, KRT17, MEF2C, PIK3R1, PPARG, RBPJ, TBX6, WNT8B* |
| *LGALS1* | Other | *APP, CTNNB1, ENPP2, GJA1, HAND1, IGF1R, PSEN2, TP53* | 2.45 x 10^-14^ | *ACADVL, ADIPOQ, ADM, APOD, APP, AQP1, ARPC2, BMP4, CCL11, CDC42EP1, CDK5R1, CHN1, CLTC, CTNNB1, CTSB, CTSH, EDA, ELF5, FGF2, FOXP1, FZD1, GAP43, GATA3, GATA5, GJA1, GLI2, HEY1, HHIP, ID2, KCNQ3, KIT, MAFF, MAPK11, MEF2C, PADI1, PIK3CA, PIK3R2, PLA2G12A, PLA2G5, PRKCA, PTCH1, PTCH2, RAC1, RDH10, S1PR1, SDC2, SNAP25, TGM2, TMEM100, TNNC1, WNT2B* |
| *IGF2R* | Trans-membrane receptor | *ADIPOQ, CITED2, CPE, FRZB, GAP43, IGF1R, ITIH4, KIF4A, LMO4, MAPK3, STAT5A, WNT7A* | 2.45 x 10^-14^ | *ADIPOQ, ADM, AIF1, APP, ARPC2, BDNF, CAPRIN1, CAV3, CCL11, CDK5R1, CITED2, COL4A1, CTNNB1, CTSB, CTSH, DCN, DOCK1, EDNRA, EXT1, FCER1A, FGF2, FYN, FZD1, GATA3, GJA1, GLI2, GNAS, GSN, HAS2, HEY1, ID2, IGF1R, IL5, INPP5D, ITIH3, KCNQ3, KCNV1, KITLG, KRT17, LYVE1, MAPK3, MEF2C, MYO10, PFN2, PLA2G12A, PLA2G5, RAC2, RBPJ, STAT5A, STAT5B, STMN1, TAGLN, TGFB1I1, TMEM17, TP53, TRAF6, VAV1, WNT11* |
| *PTCH / SHH* | Complex | *CITED2, ENPP2, GATA3, IL5, LMO4, PLA2G2A, PLD1, PPARG, STAT5A, TNF* | 2.74 x 10^-14^ | *ACADVL, ADM, APP, ARPC2, BDNF, BMP4, CALB1, CCL11, CDK5R1, CITED2, CLTC, COL4A1, COL4A3BP, DCN, FYN, FZD1, GAP43, GATA3, GATA5, GJA1, GLI2, GSN, HAS2, HEY1, ID2, IRF6, KCNJ2, KIT, KITLG, MAFF, MAP2K4, MAPK11, MAPK3, MEF2A, MEF2C, PIK3CA, PIK3R1, PLA2G5, PLA2G6, PLD1, PRKACA, PTCH1, PTCH2, RAC1, RDH10, S1PR1, SDC2, SOS1, STAT5B, STMN1, STMN3, TMEM100, TRAF6, VAV2, WNT16, WNT2B* |
| *CHRM5* | G-protein coupled receptor | *APP, CCL11, CTSB, EHF, ENPP2, GATA3, GJA1, HAS2, IL5, IL6, PRKACA, TNF, TP53* | 2.77 x 10^-14^ | *ACADVL, ADIPOQ, AIF1, AKT3, APOD, AQP1, ARPC2, BDNF, BMP4, CALB1, CDK5R1, CLTC, COL4A1, DCN, DSE, EFNA1, EHF, FGF2, FOXP1, FRZB, FST, GNA11, GNAS, GSK3B, GSN, HEYL, IGF1R, IL6, IRF6, KCNQ3, KIT, KITLG, KRT5, MAFF, MAP2K4, MAP2K6, MAPK11, MEF2A, MEF2C, NFIB, NME2, PAFAH1B1, PLA2G5, PLA2G6, PLCG1, PLD1, PLPP3, PRKCA, RAC1, RBPJ, RDH10, S100B, STAT5A, STK4, SUFU, TDRD7, TFDP1, TNF, UPK1B, UPK2, WNT16, WNT7A* |
| *MGEA5* | Enzyme | *AXIN2, CCL11, CTNNB1, FZD1, IL6, PTCH1, PTCH2, STMN1, TP53* | 2.86 x 10^-14^ | *APP, AXIN2, BMP4, CALB1, COL4A1, CPE, CTNNB1, CTSB, FZD10, FZD3, FZD6, GAP43, GATA3, GJA1, GLI2, GSN, HHIP, ID2, IFT27, IGF1R, IL6, MYO10, PIK3R1, PLCG1, PLPP3, PPARG, PRKCA, PTCH1, PTCH2, RAC1, S1PR1, SDC2, ST14, STAT5A, TCF7L2, TFDP1, TNF, TP53, WNT11* |
| Baze-doxifene | Chemical drug | *HAS2, ID2, IGF1R, MAFF, PLPP3, SEMA3C, TAGLN* | 3.26 x 10^-14^ | *ADIPOQ, AKT3, APC, AQP1, ARFIP2, ATP6V0D1, BCCIP, BDNF, BMP4, CAV3, CCL11, CDC42, CDC42EP2, CDH3, COL4A1, CPE, CTSB, DNM2, EFNA1, ENPP2, F11R, FGF2, FST, FZD1, GAP43, GNAS, GSN, HAS2, IL6, KCNMA1, MAP2K6, MAP2K7, MAPK11, MAPK12, MAPK3, MYH10, PFN2, PPARG, PTCH1, PTCH2, RAC1, SEMA3C, SLC9A3R1, SOS1, STAT5A, STAT5B, STK4, SUFU, TCF7L2, TGM2, TNF, TP53, WASF2* |
| *DKK1* | Growth factor | *AXIN2, BMP4, CDH3, COL4A1, CTNNB1, EFNA1, GJA1, GLI2, GSN, IGF1R, IL6, MAPK12, NME2, PLD1, PPARG, STAT5A, TNF, TP53* | 3.38 x 10^-14^ | *ADIPOQ, AIF1, APP, AXIN2, BDNF, BMP4, CAV3, CITED2, COL4A1, CTNNB1, FGF2, FYN, FZD3, GAP43, GATA3, GJA1, GSK3B, HAS2, HHIP, ID2, IFT27, IL5, IL6, MAPK11, MAPK3, PPARG, PTCH1, PTCH2, RAC1, RDH10, SDC2, SMARCA4, STAT5A, STAT5B, TCF7L2, TNF, TNNC1, TP53, WNT11, WNT7A* |
| Indole-3-carbinol | Chemical drug | *APP, ARFIP2, BMP4, ENPP2, FRZB, GATA3, GSN, ID2, IL5, IL6, KIT, PAFAH1B1, PPARG, SDC2, SEMA3C, SMARCA4, STMN1, TAGLN, TGM2, TNF, TP53* | 3.4 x 10^-14^ | *ADM, APC, APP, AQP1, ARFIP2, ATP6V0D1, BCCIP, BDNF, BMP4, CALB1, CAV3, CCL11, CDC42, CDC42EP2, CDK5R1, COL4A1, COL4A3BP, CPE, CTSB, DCN, DNM2, E2F8, EHF, ENPP2, FGF2, FST, GATA3, GJA1, GNAS, GSN, HAS2, IGF1R, KCNMA1, KRT17, LGR4, MAP2K6, MAP2K7, MAPK11, MAPK12, MAPK3, MEF2C, MYH10, PFN2, PLA2G12A, PLD1, PSEN2, PTCH1, PTCH2, RBPJ, SEMA3C, SOS1, STAT5A, STAT5B, STK4, STMN1, SUFU, TCF7L2, TGM2, TNNC1, TP53, WASF2* |
| Trans-nabilone | Chemical drug | *COL4A1, IL6, MAPK3, RAB13, TGM2, TNF* | 3.55 x 10^-14^ | *ACADVL, ADIPOQ, AIF1, AKT3, APP, ARPC2, BDNF, BMP4, CAV3, CCL11, CDK5R1, CTSB, DSE, FGF2, FRZB, FST, FZD1, GAP43, GATA3, GSK3B, GSN, HAS2, HEYL, ID2, IGF1R, IL6, KCNJ2, KRT17, MAFF, NME2, PAFAH1B1, PLD1, PPARG, RAC1, RBPJ, S100B, S1PR1, SDC2, STMN1, SUFU, TAGLN, TDRD7, TFDP1, TGM2, TNF, UPK1B, UPK2, WNT16* |
| Bay 38-7271 | Chemical drug | *CDC42, GJA1, IL6, PLPP3, RAC1, TGM2, TNF* | 3.55 x 10^-14^ | *ACADVL, ADIPOQ, AIF1, AKT3, APP, ARPC2, BDNF, BMP4, CAV3, CCL11, CDK5R1, CTSB, DSE, FGF2, FRZB, FST, FZD1, GAP43, GATA3, GSK3B, GSN, HAS2, HEYL, ID2, IGF1R, IL6, KCNJ2, KRT17, MAFF, NME2, PAFAH1B1, PLD1, PPARG, RAC1, RBPJ, S100B, S1PR1, SDC2, STMN1, SUFU, TAGLN, TDRD7, TFDP1, TGM2, TNF, UPK1B, UPK2, WNT16* |
| *DUSP6* | Phosphatase | *AXIN2, IL6, TNF* | 3.65 x 10^-14^ | *ADIPOQ, AIF1, APP, AQP1, ARPC2, BDNF, CCL11, CDK5R1, CITED2, CTNNB1, CTSB, CTSH, DPYSL2, DSE, EDNRA, EFNA1, ELF5, FGF2, FST, FZD1, GAP43, GJA1, GNAS, HAS2, HEY1, IGF1R, KITLG, KRT17, MAFF, MAP2K6, MEF2A, MEF2C, MYO10, PFN2, PLD1, PPARG, PTCH1, RAC1, SDC2, SNAP25, STAT5A, STAT5B, TAGLN, TDRD7, TGM2, TNNC1, TRAF6, WNT16, WNT7A* |
| 3,5-L-diiodo-thyronine | Chemical - endogenous mammalian | *APP, IL6, TNF* | 3.68 x 10^-14^ | *ADM, APP, BDNF, CALB1, CAV3, CCL11, CPE, CTSB, EFNA1, EHF, FGF2, FST, FZD3, FZD6, GAP43, GHRL, GJA1, GNAS, HAS2, ID2, IGF1R, IL5, IRF6, KCNJ2, KIT, KRT17, MAPK3, PLA2G2A, PLA2G5, PLA2G6, PLD1, PRKCA, RBPJ, SEMA3C, ST14, STAT5A, TFDP1, TGM2, TP53* |
| *FCER2* | Trans-membrane receptor | *APP, IL6, TNF* | 3.68 x 10^-14^ | *ADIPOQ, BCCIP, BDNF, CALB1, CAV3, CDH3, CITED2, COL4A1, COL4A3BP, CSK, CTNNB1, CTSB, DCN, EFNA1, EHF, ENPP2, FGF2, FST, GAP43, GJA1, GLI2, GNAS, HAS2, HEY1, IGF1R, IL5, IL6, ITIH4, KIT, KRT17, PIK3R1, PLD1, PPARG, PRKACA, RBPJ, SEMA3C, SNX10, STAT5A, TFDP1, TGFB1I1, TGM2, TNF, TNNC1* |
| *HDAC7* | Transcription regulator | *APP, BDNF, CDC42, FGF2, IGF1R, IL6, MEF2A, PADI1, PLA2G2F, PLCG1, PPARG, TAGLN, TNF* | 4.01 x 10^-14^ | *APC, AQP1, ARFIP2, ATP6V0D1, AXIN2, BCCIP, BDNF, BMP4, CDC42, CDC42EP2, CDK5R1, COL4A1, CPE, CTNNB1, DNM2, EFNA1, ENPP2, FST, FZD3, GAP43, GATA3, GJA1, GLI2, GNAS, GSN, HHIP, ID2, IGF1R, IL6, KCNMA1, KIF4A, KIT, MAP2K6, MAP2K7, MAPK12, MYH10, PFN2, PPARG, PSEN2, PTCH1, PTCH2, SDC2, SOS1, STAT5A, STAT5B, STK4, SUFU, TCF7L2, TDRD7, TFAP2A, TGM2, TNF, TNNC1, WASF2, WNT11* |
| *UBE3A* | Enzyme | *BDNF, CTNNB1, EXT2, FST, GJA1, GLI2, IL5, IL6, NFIB, TAGLN, TNF* | 4.15 x 10^-14^ | *ACADVL, ADIPOQ, ADM, AKT3, APC, APP, AQP1, ARFIP2, ATP6V0D1, AXIN2, BCCIP, BDNF, BMP4, CAV3, CDC42, CDC42EP2, CDH3, CPE, CTSB, CTSH, DCN, DNM2, DPYSL2, EDNRA, ELF5, ENPP2, FGF2, FST, FYN, GATA3, GJA1, GLI2, GNAS, GSK3B, GSN, ID2, IGF1R, IL5, IL6, KCNJ2, KCNMA1, KIT, MAP2K7, MAPK11, MAPK3, MYO10, PAFAH1B2, PDE2A, PPARG, PRKCA, PSEN2, PTCH1, PTCH2, RBPJ, RDH10, SEMA3C, SNAP25, SOS1, ST14, STK4, STMN1, SUFU, TBX6, TFDP1, TGFB1I1, TP53, WASF2, WNT2, WNT7A* |
| *EDA* | Cytokine | *AQP1, BMP4, IL5, IL6, NME2, PPARG, PTCH1, RAC1, TNF, TP53* | 4.26 x 10^-14^ | *AKT3, APOD, APP, AQP1, BCCIP, BDNF, BMP4, CALB1, CAV3, CDH3, CDK5R1, CSK, CTNNB1, CTSB, DCN, EFNA1, EHF, ENPP2, FGF2, FRZB, FST, GAP43, GJA1, GNAS, GSK3B, HAS2, IGF1R, IL6, KIT, KRT17, MAPK3, PIK3R1, PLD1, PTCH1, RAC1, RBPJ, SEMA3C, STAT5A, STMN1, TFDP1, TGFB1I1, TGM2, WNT16* |
| *CUL1* | Enzyme | *AXIN2, CTNNB1, GHRL, ID2, IL6, PLA2G10, PRKCA, PRKCB, TNF* | 4.27 x 10^-14^ | *AXIN2, BCCIP, BMP4, CAV3, CCL11, CITED2, CSK, DCN, E2F8, EDNRA, ENPP2, ERCC3, FGF2, FST, GAP43, GATA3, GLI2, HAS2, HEY1, ID2, IGF1R, IL5, IL6, INPP5D, KRT17, LGR4, MAFF, MEF2C, PIK3R1, PPARG, RAC1, S100B, S1PR1, SEMA3C, STAT5A, STAT5B, STMN1, TAGLN, TFDP1, TGFB1I1, TP53, WNT7A* |
| *TRAF* | Group | *BMP4, CDH3, CITED2, CTNNB1, FST, GJA1, HAS2, ID2, INPP5D, RAC1, TBX6, TNF* | 4.65 x 10^-14^ | *ADM, APP, ARPC2, BCCIP, BDNF, CALB1, CAV3, CDH3, CDK5R1, CSK, CTNNB1, CTSB, DCN, EFNA1, EHF, ENPP2, FGF2, FST, GAP43, GATA3, GJA1, GLI2, GNAS, HAS2, HEY1, IGF1R, KIT, KRT17, MAFF, MAP2K4, MEF2C, PIK3R1, PLA2G5, PLD1, PPARG, PRKACA, RBPJ, SEMA3C, STAT5A, STMN1, TFDP1, TGFB1I1, TGM2, TNF* |
| *ACTN2* | Transcription regulator | *ADIPOQ, BDNF, BMP4, CTNNB1, EFNA1, GATA3, HAND1, ID2, IGF1R, IL5, IL6, PPARG, PTCD2, PTCH1, TAGLN, TBX6, WNT8B* | 4.82 x 10^-14^ | *ADIPOQ, APC, AQP1, ARFIP2, ATP6V0D1, BCCIP, BDNF, BMP4, CDC42, CDC42EP2, CPE, CTNNB1, DNM2, EFNA1, ENPP2, FST, FZD1, FZD6, GJA1, GNAS, GSN, IL6, KCNJ2, KCNMA1, MAP2K6, MAP2K7, MAPK11, MAPK12, MAPK3, MEF2C, MYH10, PFN2, PPARG, PRKCA, PTCH1, PTCH2, SEMA3C, SOS1, STAT5A, STAT5B, STK4, SUFU, TCF7L2, TGM2, TP53, WASF2, WNT11, WNT16, WNT2, WNT2B, WNT7A, WNT8B* |
| *TGFB1* | Growth factor | *GAP43, MAP2K4, MAP2K7, MAPK9, PRKCA, TP53* | 4.87 x 10^-14^ | *ADIPOQ, ADM, APP, ARPC2, BDNF, CAPRIN1, CCL11, CDK5R1, CITED2, COL4A1, CTNNB1, CTSB, CTSH, DCN, EDNRA, EXT1, FCER1A, FGF2, FZD1, GATA3, GLI2, GNAS, GSN, HAS2, HEY1, ID2, IL5, IL6, INPP5D, ITIH3, KCNQ3, KCNV1, KITLG, KRT17, LYVE1, MAPK3, MEF2C, MYO10, PFN2, PPARG, RAC1, STAT5A, STAT5B, TAGLN, TGFB1I1, TGM2, TMEM17, TNF, TP53, WNT11* |
| Cyanoco-balamin | Chemical - endogenous mammalian | *CTNNB1, IL6, TNF, TRAF6* | 5.33 x 10^-14^ | *AIF1, APC, APP, AXIN2, BDNF, CITED2, CTNNB1, CTSB, DPYSL2, ENPP2, FGF2, FYN, FZD1, FZD3, FZD6, GSK3B, HEY1, IL6, MAFF, MAPK11, MAPK3, PRKCA, PTCH1, PTCH2, RBPJ, S100B, S1PR1, TNF, TP53, TRAF6* |
| *SPEN* | Transcription regulator | *ADIPOQ, AXIN2, DOCK1, GATA3, HAND1, ID2, KIF4A, KIT, LMO4, MEF2C, PLCG2, TNF* | 5.6 x 10^-14^ | *ADIPOQ, APC, AQP1, ARFIP2, ATP6V0D1, BCCIP, BMP4, CDC42, CDC42EP2, CPE, CTNNB1, DNM2, EFNA1, ENPP2, FGF2, FST, GATA3, GJA1, GLI2, GNAS, GSN, HEY1, HEYL, ID2, IGF1R, IL6, KCNMA1, LYVE1, MAP2K6, MAP2K7, MAPK11, MAPK12, MYH10, PFN2, PPARG, PTCH1, PTCH2, SEMA3C, SOS1, STAT5A, STAT5B, STK4, SUFU, TAGLN, TCF7L2, TGM2, TP53, WASF2* |
| R5020 | Chemical reagent | *ADIPOQ, AQP1, CITED2, GJA1, ID2, KITLG, MEF2C, PPARG, TNF* | 6.54 x 10^-14^ | *ADIPOQ, ADM, AKT3, APOD, AQP1, ARPC2, BDNF, BMP4, CCL11, CDH3, CDK5R1, COL4A1, COL4A3BP, CTNNB1, CTSB, DPYSL2, DSE, DVL2, EDNRA, EFNA1, ELF5, F11R, FZD1, GATA3, GLI2, GNAS, GSN, HAS2, HEY1, KITLG, MAFF, MAP2K6, MAP2K7, MAPK12, MEF2A, MEF2C, MYO10, NFIB, PFN2, PLCG1, PLD1, PSEN2, PTCH1, RAC1, RDH10, S1PR1, SLC9A3R1, SNAP25, STAT5A, STAT5B, TAGLN, TDRD7, TGM2, TNNC1* |
| *YWHAG* | Other | *GLI2, HEY1, HEYL, TAGLN, TP53* | 7.05 x 10^-14^ | *ACADVL, ADIPOQ, ADM, AKT3, APP, AXIN2, BDNF, CAV3, CDH3, CDK5R1, CITED2, COL4A1, CTSH, DSE, EFNA1, ENPP2, FGF2, FYN, GJA1, GLI2, GNAS, GSK3B, GSN, HAS2, ID2, KCNMA1, KITLG, KRT17, MAP2K4, MAP2K6, MAP2K7, MAPK12, MAPK3, MEF2C, MYH10, PAFAH1B2, PDE2A, PLA2G5, PLD1, PRKCA, PSEN2, PTCH1, RBPJ, RDH10, ST14, STAT5B, STMN1, TAGLN, TCF7L2, TDRD7, TFDP1, TGFB1I1, TGM2, TNNC1, TP53, TRAF6, WNT16, WNT2, WNT7A* |
| *FLT3* | Kinase | *APC, BDNF, FGF2, GJA1, IGF1R* | 7.07 x 10^-14^ | *ADIPOQ, AIF1, ANXA7, APP, BDNF, CTNNB1, FST, FYN, GAP43, GATA3, GJA1, GLI2, ID2, IGF1R, IL5, IL6, IRF6, KRT17, MAP2K4, MAPK11, MAPK3, MEF2A, MEF2C, PLA2G5, PPARG, S1PR1, STAT5B, STMN1, TGM2, TNF, TNNC1* |
| *SYK/ZAP* | Group | *CTNNB1, IGF1R, IL6, PPARG, TBX6, TNF, TP53* | 7.48 x 10^-14^ | *ADIPOQ, BDNF, CCL11, CDK5R1, CITED2, CTNNB1, CTSB, FGF2, FOXP1, FST, FYN, FZD1, GATA3, GJA1, GLI2, HAS2, ID2, IGF1R, IL6, INPP5D, KIT, MAFF, MAP2K4, MAPK3, MEF2A, MEF2C, MYH10, PIK3CA, PLA2G5, PLCG1, PPARG, RAC1, S1PR1, SDC2, STAT5A, STAT5B, STMN1, TGM2, TNF, TNNC1, TP53, VAV1* |
| *TESPA1* | Other | *ADIPOQ, BMP4, GJA1, ID2, IGF1R, IL6, MAPK3, PIK3R1, PPARG* | 7.97 x 10^-14^ | *AIF1, AKT3, APOD, AQP1, ARF1, ARPC2, BCCIP, CALB1, CDC42, CDC42EP1, CDC42EP2, CDH3, CHN1, COL4A3BP, CSK, CTSH, DCN, DSE, DVL2, E2F8, EFNA1, EHF, ENPP2, FOXP1, FRZB, GNAS, GSK3B, GSN, HAND1, HEYL, IL6, IRF6, KCNJ2, KIT, KITLG, KRT17, MAFF, MAP2K4, MAPK12, MEF2A, MEF2C, MYH10, MYO10, PFN2, PIK3CA, PIK3R1, PLA2G5, PLA2G6, PLCG1, PRKCB, PTCH1, RAC1, RBPJ, RDH10, S100B, S1PR1, SDC2, SEMA3C, STMN1, STMN3, TDRD7, TFDP1, TGFB1I1, TNNC1, WNT16* |
| *DKK1* | Growth factor | *CCL11, FGF2, GATA3, IL5, IL6, TNF* | 8.76 x 10^-14^ | *APP, AXIN2, BDNF, BMP4, CTNNB1, FGF2, GATA3, GSK3B, PPARG, TNNC1, TP53, WNT11, WNT7A* |
| *ITGAM* | Trans-membrane receptor | *ADIPOQ, ADM, CAV3, CTNNB1, GLI2, GSK3B, IGF1R, IL5, IL6, PPARG, RDH10, TNF, TP53* | 9.03 x 10^-14^ | *ADM, APOD, APP, AQP1, BCCIP, BMP4, CALB1, CAV3, CDH3, CITED2, COL4A3BP, CSK, CTNNB1, CTSH, DCN, DOCK1, EFNA1, EHF, ENPP2, FOXP1, FYN, GLI2, GNAS, HEY1, ID2, IGF1R, IL6, KIT, KITLG, KRT17, MAP2K6, MEF2C, PIK3CA, PIK3R1, PLA2G5, PLCG2, PTCH1, RAC1, RAC2, RDH10, S1PR1, SDC2, SEMA3C, STAT5A, TAGLN, TDRD7, TGFB1I1, TGM2, TNNC1, TRAF6, VAV1* |
| *SPN* | Trans-membrane receptor | *COL4A1, DCN, KRT17, LIN7C, TAGLN, TGFB1I1, WNT7A* | 1.03 x 10^-13^ | *ACADVL, AKT3, APP, AXIN2, BDNF, CDH3, CDK5R1, CITED2, COL4A1, CTNNB1, CTSB, CTSH, DSE, EFNA1, ELF5, ENPP2, FOXP1, FST, FYN, GSK3B, GSN, HAS2, ID2, IGF1R, KCNMA1, KRT17, MAP2K6, MAP2K7, MAPK12, MAPK3, MYH10, PAFAH1B2, PDE2A, PIK3CA, PLA2G12A, PLA2G5, PLD1, PPARG, PRKCA, PSEN2, PTCH1, RBPJ, ST14, STAT5A, STAT5B, STMN1, TCF7L2, TDRD7, TFDP1, TGFB1I1, TGM2, TNF, TP53, WNT2, WNT7A* |
| *CHRD* | Other | *AIF1, GJA1, ID2, IGF1R, IL5, IL6, KIT, MAPK11, MEF2C, PRKCA, PRKCB, TNF* | 1.11 x 10^-13^ | *ADIPOQ, BDNF, BMP4, CAV3, CCL11, COL4A1, CTNNB1, DCN, DPYSL2, EFNA1, EHF, FGF2, FST, FZD1, GAP43, GATA3, GJA1, GLI2, GNAS, HAND1, HAS2, HEY1, ID2, IGF1R, IL5, IL6, INPP5D, KIT, KRT17, MAPK3, MEF2C, MYH10, PIK3R1, PLD1, PLPP3, PTCH1, RBPJ, STAT5A, TP53, WNT2* |
| *MAP3K4* | Kinase | *ADM, CCL11, GATA3, IL5, IL6, KITLG, MAFF, MAP2K6, PPARG, PRKCA, TNF, TP53* | 1.2 x 10^-13^ | *APOD, APP, AQP1, ARPC2, BDNF, BMP4, CAV3, CCL11, CDK5R1, CITED2, CTNNB1, EFNA1, FGF2, FST, GAP43, GNAS, HAS2, HEY1, IL6, KITLG, LMO4, MAFF, MAP2K6, MAPK9, MEF2C, PLA2G2A, PTCH1, RAC1, SDC2, STAT5A, STK4, TAGLN, TGM2, TNF, TNNC1, WNT7A* |
| *MAPK8IP1* | Other | *ACADVL, AQP1, BMP4, CTNNB1, CTSB, DOCK1, FYN, GAP43, HEYL, ID2, IGF1R, IL6, INPP5D, LMO4, MAPK9, PIK3CA, PRKCA, RAC1, STMN1, TFAP2A, TNF, TP53* | 1.34 x 10^-13^ | *ADIPOQ, AIF1, AKT3, APOD, APP, AQP1, AXIN2, BDNF, BMP4, CDK5R1, CITED2, COL4A1, CTSB, DPYSL2, ENPP2, FRZB, FZD3, GAP43, GATA3, GJA1, GLI2, GSK3B, HEYL, ID2, IL5, IL6, LYVE1, MAFF, MAP2K7, MAPK3, PRKCA, PTCH1, PTCH2, RBPJ, S100B, S1PR1, STMN1, TNF, TRAF6, WNT16* |
| *MIR-146* | MicroRNA | *ADIPOQ, IL6, PPARG, TNF* | 1.47 x 10^-13^ | *AGPS, APP, BCCIP, BDNF, CALB1, CAV3, CDH3, CITED2, COL4A3BP, CSK, CTNNB1, CTSB, DCN, EFNA1, EHF, ENPP2, FST, GAP43, GATA3, GJA1, GLI2, GNAS, HAS2, HEY1, IL5, IL6, IRF6, KIT, KRT17, PIK3CA, PIK3R1, PLD1, PRKACA, RBPJ, RDH10, SEMA3C, STAT5A, TFDP1, TGFB1I1, TGM2, TNF, TRAF6, TTC30B, TTLL1, WASF2* |
| *MEMO1* | Other | *GSK3B, MAP2K4, MAP2K7, MAPK10* | 1.54 x 10^-13^ | *ADIPOQ, ADM, APC, AQP1, ARFIP2, ATP6V0D1, BCCIP, BDNF, BMP4, CCL11, CDC42, CDC42EP2, CPE, CTNNB1, CTSB, DNM2, EFNA1, ENPP2, FGF2, FST, FZD1, GJA1, GLI2, GNAS, GSN, HAS2, IL5, IL6, KCNMA1, MAP2K6, MAP2K7, MAPK11, MAPK12, MAPK3, MYH10, PFN2, PPARG, PTCH1, PTCH2, RAC1, RDH10, SEMA3C, SOS1, STAT5A, STAT5B, STK4, SUFU, TCF7L2, TGM2, WASF2* |
| *IQGAP1* | Other | *CTNNB1, FZD3, FZD6, WNT11* | 1.63 x 10^-13^ | *ADIPOQ, APC, APP, AQP1, ARFIP2, ATP6V0D1, AXIN2, BCCIP, BDNF, CAV3, CCL11, CDC42, CDC42EP2, CDK5R1, CITED2, COL4A1, CPE, CTNNB1, CTSB, DNM2, DSE, EFNA1, FST, FZD1, FZD3, GAP43, GLI2, GNAS, GSN, HAS2, HHIP, ID2, IGF1R, IL5, IL6, KCNMA1, LMO4, MAP2K6, MAP2K7, MAPK11, MAPK12, MYH10, NFIB, PFN2, PLA2G2A, PLCG1, PRKCA, RAC1, SDC2, SEMA3C, SOS1, STAT5A, STAT5B, STK4, SUFU, TAGLN, TCF7L2, TDRD7, TGM2, WASF2, WNT11* |
| *CR2* | Trans-membrane receptor | *APP, HEY1, TNF, TP53* | 1.65 x 10^-13^ | *ADM, APP, AQP1, ARPC2, BDNF, BMP4, CAV3, CCL11, CDK5R1, CTNNB1, CTSB, FGF2, FST, FYN, GAP43, GJA1, HEY1, ID2, IL5, IRF6, KCNJ2, MAFF, MAPK3, PLA2G6, PPARG, PRKCA, PTCH1, RAC1, SDC2, STAT5A, STAT5B, TGM2, TNNC1, VAV1* |
| Cinacalcet | Chemical drug | *ACADVL, ADIPOQ, APP, BDNF, COL4A1, GAP43, GHRL, GNAS, GSK3B, IFT27, IL6, NPY1R, PPARG, RAC1, SNAP25, TNF, TP53* | 1.77 x 10^-13^ | *ADIPOQ, ADM, AKT3, APOD, ARPC2, AXIN2, CALB1, CCL11, CDH3, COL4A1, DSE, EFNA1, EHF, FGF2, FLOT1, FRZB, FST, FZD1, FZD3, GATA3, GLI2, GNAS, GSK3B, HEY1, HHIP, ID2, IL6, INPP5D, KIT, KRT17, MAPK3, NME2, PIK3CA, PIK3R2, PPARG, PRKCA, PTCH2, RBPJ, S1PR1, SCN1B, SDC2, SLC9A3R1, STAT5A, STMN1, TAGLN, TDRD7, TGM2, TNF, TP53, WNT11, WNT16* |
| Hydro-quinone | Chemical - endogenous mammalian | *BDNF, CCL11, IL5, IL6, PFN2, TNF, UCHL1* | 1.89 x 10^-13^ | *ADIPOQ, ADM, AIF1, AIMP2, ANXA7, APOD, APP, ARF1, ARFIP2, ARPC2, ATP6V0D1, BDNF, CALB1, CAPRIN1, CAV3, CCL11, CDC42EP1, CHN1, CLTC, CPE, CSK, DCN, DNM2, DPYSL2, DVL2, EDA, EFNA1, EHF, EMP2, ENPP2, EXT2, FCER1A, FGF2, FOXP1, FST, FYN, FZD3, GAP43, GLI2, GNA11, GNAS, HAS2, HEYL, HHIP, ID2, IFT27, IGF1R, IL5, IL6, IRF6, ITIH3, ITIH4, ITIH5, KCNB2, KCNJ2, KCNMB1, KCNQ3, KIT, KITLG, KRT17, KRT5, KYAT3, LGR4, LYVE1, MAP2K4, MAPK10, MAPK3, MAPK9, MYO10, NME2, NPY1R, PAFAH1B1, PIK3R1, PIK3R2, PLA2G12A, PLA2G3, PLA2G6, PLCG1, PLCG2, PLD1, PLPP3, PRKACA, PRKCA, PTCH2, RAC2, RAF1, RBPJ, RDH10, SDC2, SEMA3C, SLC9A3R1, SMARCA4, SNX10, SOS1, SS18L1, STAT5A, STK4, STMN3, SUFU, TFAP2A, TGM2, TMEM100, TMEM17, TNF, UCHL1, WASF2, WNT2B, WNT8B* |
| *MAP3K14* | Kinase | *BMP4, COL4A1, CPE, CTSH, FST, GJA1, HHIP, ID2, IL6* | 1.93 x 10^-13^ | *ADM, AGPS, APP, BCCIP, CALB1, CDH3, CITED2, COL4A3BP, CSK, DCN, EFNA1, EHF, ENPP2, FCER1A, FGF2, FST, FYN, FZD1, GAP43, GNAS, GSK3B, HAS2, ID2, IL5, IL6, IRF6, KIT, KRT17, MAP2K4, MAPK3, MEF2C, PIK3CA, PIK3R1, PLA2G5, PLCG2, PLD1, PPARG, PRKACA, RAC1, RBPJ, SEMA3C, SNX10, STAT5A, STMN1, TAGLN, TDRD7, TFDP1, TGFB1I1, TTC30B, TTLL1, VAV1* |
| *PLC GAMMA*  *(PLCG1, PLCG2)* | Group | *BDNF, IL6, MEF2A, MEF2C, TNF, TP53* | 1.95 x 10^-13^ | *ADIPOQ, ADM, ARPC2, BCCIP, CALB1, CDC42EP1, CDH3, CDK5R1, CHN1, CITED2, COL4A3BP, CSK, CTSH, DCN, EFNA1, EHF, ENPP2, FZD1, GATA3, GNAS, HEY1, ID2, IGF1R, IL5, IRF6, KCNJ2, KIT, KRT17, MAFF, MAP2K4, MAPK3, MEF2C, PIK3R1, PLA2G5, PLA2G6, PLCG2, PPARG, PRKACA, PRKCA, RAC1, RBPJ, SDC2, SEMA3C, STAT5A, STMN1, TDRD7, TFDP1, TGFB1I1, TNNC1* |
| *FGR* | Kinase | *GATA3, GJA1, ID2, IL6, MAPK3, RAF1, TNF, TOLLIP* | 1.95 x 10^-13^ | *ADM, APOD, APP, AQP1, BDNF, CCL11, CDK5R1, CITED2, COL4A1, CTSB, DPYSL2, DSE, EDNRA, EFNA1, ELF5, FOXP1, FZD1, GAP43, GLI2, GNAS, GSN, HAS2, HEY1, ID2, KITLG, KRT17, MAP2K4, MAP2K6, MEF2C, MYO10, PFN2, PIK3CA, PLA2G5, PLCG1, PLD1, PTCH1, RAC1, RBPJ, RDH10, S1PR1, SNAP25, STAT5A, STAT5B, STMN1, TDRD7, TFDP1, TGM2, TNF, VAV1* |
| *CDK7* | Kinase | *AXIN2, CTNNB1, DOCK1, EXT1, FYN, MYO10, PLPP3, TGFB1I1, WNT11, WNT2, WNT2B* | 2.09 x 10^-13^ | *APC, AQP1, ARFIP2, ATP6V0D1, BCCIP, BMP4, CDC42, CDC42EP2, CDK5R1, COL4A3BP, CPE, CTNNB1, DNM2, E2F8, EDNRA, EFNA1, ENPP2, EXT2, FGF2, FRZB, FST, GAP43, GATA3, GJA1, GNAS, GSN, HAND1, IL5, KCNJ2, KCNMA1, KITLG, KRT17, MAFF, MAP2K6, MAP2K7, MAPK11, MAPK12, MAPK3, MEF2A, MYH10, PFN2, PRKCA, PSEN2, PTCH1, PTCH2, SEMA3C, SOS1, STAT5A, STAT5B, STK4, SUFU, TCF7L2, TDRD7, TFAP2A, TGM2, TNNC1, WASF2, WNT7A, WNT8B* |
| *TNFRSF13C* | Trans-membrane receptor | *APP, CAV3, GATA3, GJA1, IL6, NCS1, PRKCA, RAC1, STMN1, TNF, TP53* | 2.12 x 10^-13^ | *ADIPOQ, ADM, AIF1, AIMP2, APC, APOD, ARF1, ARFIP2, ARPC2, ATP6V0D1, BDNF, CALB1, CAPRIN1, CAPZB, CAV3, CCL11, CDC42EP1, CDC42EP2, CLTC, CPE, CSK, CTNNB1, DCN, DNM2, DVL2, EDA, EFNA1, EHF, EMP2, EXT2, FCER1A, FOXP1, FST, FZD3, GAP43, GLI2, GNA11, GNAS, HAND1, HAS2, HHIP, IFT27, IGF1R, IL5, IL6, ITIH3, ITIH4, ITIH5, KCNB2, KCNJ2, KCNMB1, KCNQ3, KIT, KITLG, KRT17, KYAT3, LGR4, LMO4, LYVE1, MAP2K4, MAPK10, MAPK9, MEF2A, NFIB, NME2, PAFAH1B1, PIK3R1, PIK3R3, PLA2G12A, PLA2G2A, PLA2G3, PLA2G5, PLCG1, PLCG2, PLD1, PLPP3, PRKACA, PRKCB, PTCH2, RAC2, RBPJ, RDH10, S100B, SDC2, SEMA3C, SLC9A3R1, SMARCA4, SNX10, SOS1, STAT5A, STK4, SUFU, TBX6, TFAP2A, TGM2, TMEM17, TNF, UCHL1, WASF2, WNT8B* |
| *LRRFIP1* | Other | *CCL11, FCER1A, IL5, IL6, PPARG, TNF* | 2.15 x 10^-13^ | *ARPC2, AXIN2, BDNF, BMP4, CALB1, CAV3, CCL11, CDK5R1, COL4A1, CTNNB1, EFNA1, EHF, FGF2, FST, FZD3, GATA3, GJA1, GLI2, GNAS, HAS2, HEY1, HHIP, ID2, IL5, IL6, KIT, KRT17, MAFF, PLD1, PPARG, PTCH1, PTCH2, RBPJ, SDC2, STAT5A, TGM2, TNF, TP53, WNT11* |
| *ESR1*/ Estrogen/ *SP1* | Complex | *APP, CDC42, FGF2, MAPK3, RAF1, TP53* | 2.17 x 10^-13^ | *APC, AQP1, ARFIP2, ATP6V0D1, BCCIP, BDNF, BMP4, CALB1, CDC42, CDC42EP2, CITED2, CPE, CTNNB1, DNM2, EDA, EFNA1, ENPP2, FST, GAP43, GJA1, GNAS, GSN, HAS2, IL6, KCNH1, KCNMA1, KCNQ3, KIT, MAP2K6, MAP2K7, MAPK11, MAPK12, MYH10, PADI1, PFN2, PTCH1, PTCH2, RAF1, S1PR1, SEMA3C, SNAP25, SOS1, STAT5A, STAT5B, STK4, SUFU, TCF7L2, TGM2, TNF, TP53, WASF2* |
| G-actin  (*ACTA1,*  *ACTA2,*  *ACTB,*  *ACTC1,*  *ACTG2*) | Group | *AXIN2, BMP4, COL4A1, CTNNB1, EFNA1, FRZB, FZD1, GATA3, GATA5, IL5, IL6, PPARG, TNF, TP53* | 2.25 x 10^-13^ | *ACADVL, AKT3, APP, AXIN2, BDNF, CDH3, COL4A1, CTNNB1, CTSB, CTSH, EDNRA, EFNA1, EHF, ENPP2, FYN, GPM6A, GSK3B, GSN, HAS2, HEY1, HHIP, ID2, IGF1R, IL5, IL6, IRF6, KCNJ2, KCNMA1, KCNQ3, KITLG, MAFF, MAP2K6, MAP2K7, MAPK12, MEF2C, MYH10, PAFAH1B2, PDE2A, PLPP3, PRKCA, PSEN2, PTCH1, RAC2, RBPJ, SDC2, ST14, STMN1, TAGLN, TCF7L2, TFDP1, TGFB1I1, TGM2, TNF, WNT2, WNT7A* |
| *SERPINC1* | Enzyme | *ADM, AXIN2, BDNF, CPE, EFNA1, FGF2, FST, GNAS, IGF1R, IL6, KITLG, NPY1R, PLA2G10, SMARCA4, SNAP25, TP53* | 2.37 x 10^-13^ | *ADIPOQ, APP, BCCIP, CALB1, CDC42EP1, CDH3, COL4A1, CSK, CTNNB1, CTSB, DCN, EDNRA, EFNA1, EHF, ENPP2, FGF2, FST, GAP43, GNAS, HAS2, IGF1R, IL5, IL6, ITIH4, KIT, KRT17, MAPK3, PIK3R1, PLA2G2A, PLD1, PLPP3, PPARG, RAC1, RAC2, RBPJ, S100B, SDC2, SEMA3C, STAT5A, TFDP1, TGFB1I1, TGM2, TNF, TNNC1* |
| T-cell alpha/beta receptor  (members of *TCR*) | Complex | *ADM, ATP6V0D1, BDNF, CITED2, CTSB, CTSH, EXT1, ID2, IGF1R, IL6, KCNJ2, NME7, PIK3R1, PPARG, TNF, TP53* | 2.58 x 10^-13^ | *ADIPOQ, APP, AQP1, BCCIP, BMP4, CALB1, CCL11, CDH3, CITED2, COL4A3BP, CSK, DCN, EFNA1, EHF, ENPP2, FST, FZD1, GAP43, GATA3, GJA1, GLI2, GNAS, IGF1R, IL5, KIT, KITLG, KRT17, MAP2K6, MAPK3, MEF2C, PIK3R1, PLD1, PPARG, PTCH1, RAC1, RBPJ, SDC2, SEMA3C, STAT5A, TAGLN, TFDP1, TGFB1I1, TGM2, TNNC1, VAV1* |
| *MAP3K10* | Kinase | *BMP4, CDC42, CDH3, CTNNB1, IL5, PIK3R1, TNF* | 3.04 x 10^-13^ | *AKT3, APOD, APP, AQP1, BDNF, BMP4, CAV3, CCL11, CDK5R1, CTNNB1, CTSB, DSE, EFNA1, FGF2, FRZB, GAP43, GATA3, GNAS, GSK3B, HAS2, IGF1R, IL6, KITLG, KRT17, MAP2K4, MAP2K6, MAPK3, MAPK9, MEF2C, PLD1, PTCH1, RAC1, SDC2, STMN1, TAGLN, TDRD7, TGM2, TNF, TNNC1, WNT16, WNT7A* |
| *MAPK15* | Kinase | *ACADVL, BDNF, GSK3B, IL6, PPARG, TNF, TP53* | 3.12 x 10^-13^ | *AKT3, APC, APP, AQP1, ARFIP2, ATP6V0D1, BCCIP, BDNF, BMP4, CDC42, CDC42EP2, CDK5R1, CPE, CTNNB1, DNM2, EFNA1, ENPP2, FGF2, FRZB, FST, GAP43, GNAS, GSK3B, GSN, IL6, KCNMA1, MAP2K6, MAP2K7, MAPK11, MAPK12, MAPK3, MYH10, PFN2, PTCH1, PTCH2, SEMA3C, SOS1, STAT5A, STAT5B, STK4, STMN1, SUFU, TCF7L2, TGM2, TNF, TP53, WASF2, WNT16* |
| L-685,458 | Chemical - protease inhibitor | *AKT3, APP, BDNF, CDK5R1, FGF2, FRZB, GAP43, GJA1, GSK3B, ID2, IGF1R, IL6, MAPK3, STMN1, TNF, TP53, WNT16* | 3.21 x 10^-13^ | *ACADVL, ADIPOQ, AKT3, APP, AXIN2, BDNF, CAV3, CCL11, CDH3, COL4A1, CTNNB1, CTSH, EFNA1, ENPP2, FGF2, FYN, FZD1, GJA1, GSK3B, GSN, HAS2, HEY1, HEYL, ID2, IGF1R, IL5, KCNMA1, MAP2K6, MAP2K7, MAPK12, MAPK3, MYH10, PAFAH1B2, PDE2A, PPARG, PRKCA, PSEN2, PTCH1, RAC1, RBPJ, ST14, STMN1, TCF7L2, TFDP1, TGFB1I1, TGM2, TNF, TP53, WNT2, WNT7A* |
| *BMP7* | Growth factor | *ADIPOQ, APP, BDNF, PPARG, TNF* | 3.68 x 10^-13^ | *ADIPOQ, APOD, APP, AQP1, AXIN2, BDNF, BMP4, CAV3, CCL11, CDK5R1, COL4A1, COL4A3BP, CTNNB1, CTSB, EDNRA, EFNA1, FGF2, FST, FZD1, GAP43, GATA3, GJA1, HAS2, ID2, IGF1R, IL6, LYVE1, MAP2K7, MAPK3, MEF2C, PIK3R1, PLPP3, PPARG, PSEN2, PTCH1, S1PR1, SDC2, TNNC1, WNT11* |
| *STK25* | Kinase | *IL5, IL6, KIT, PPARG, TNF* | 4.21 x 10^-13^ | *ADIPOQ, ADM, AIF1, APP, ARPC2, AXIN2, BDNF, BMP4, CAV3, CDC42EP1, CDK5R1, CHN1, CITED2, COL4A1, CTSB, CTSH, FGF2, FST, FYN, FZD3, GAP43, GATA3, GJA1, GLI2, HEY1, HHIP, ID2, IGF1R, IL5, MAFF, MAPK11, MAPK3, PIK3CA, PLD1, PTCH1, PTCH2, RDH10, STAT5B, TGM2, TNNC1, TRAF6, WNT11, WNT16* |
| Integrin alpha 2 beta 1  *(ITGA1-6, ITGAL, ITGB1-3)* | Complex | *BDNF, CDH3, CITED2, CTSH, EDA, EFNA1, ENPP2, IL6, LGR4, LYVE1, MAPK9, NFIB, PRKACA, PTER, RAB13, RAF1, SULT1B1, TNF, TP53* | 4.3 x 10^-13^ | *ADM, APP, CTNNB1, CTSB, EFNA1, FGF2, GAP43, GNAS, HAS2, ID2, IL5, IRF6, KCNJ2, KITLG, MAP2K6, MAPK3, MEF2C, PLA2G6, PPARG, PRKCA, TAGLN, TGM2, TNF, TP53* |
| *BMP*  *(BMP1-8, BMP10, BMP15)* | Group | *ARPC2, CDK5R1, CTNNB1, DPYSL2, FGF2, FST, HEY1, IL6, MAFF, TGM2, TNF, TP53* | 4.61 x 10^-13^ | *ADIPOQ, BDNF, BMP4, CAV3, CCL11, COL4A1, CTNNB1, DCN, DPYSL2, EFNA1, EHF, FGF2, FST, FZD1, GAP43, GATA3, GJA1, GLI2, GNAS, HAND1, HAS2, HEY1, ID2, IGF1R, IL5, IL6, INPP5D, KIT, KRT17, MAPK3, MEF2C, MYH10, PIK3R1, PLD1, PLPP3, RBPJ, STAT5A, TP53, WNT2* |
| Tauro-lithocholic acid | Chemical - endogenous mammalian | *PRKCA, PRKCB, TNF* | 4.99 x 10^-13^ | *ADM, AIF1, BDNF, BMP4, CAV3, CDK5R1, COL4A1, COL4A3BP, CTNNB1, CTSH, DCN, EDNRA, FST, FYN, GATA3, GJA1, GLI2, GSN, HAS2, HEY1, HEYL, IGF1R, IL6, KCNJ2, KITLG, MAP2K7, MAPK11, MEF2C, NME2, PAFAH1B1, PIK3CA, PLD1, PLPP3, PRKACA, PRKCA, PSEN2, RAC1, RDH10, S100B, SUFU, TAGLN, TNF, TNNC1, TP53, UPK1B, UPK2* |
| Vitamin A | Chemical - endogenous mammalian | *IL6, TNF, TP53* | 5.07 x 10^-13^ | *ADIPOQ, AIF1, APP, ARPC2, AXIN2, BMP4, CAV3, CDK5R1, COL4A1, CTSB, FGF2, FST, FYN, FZD3, GATA3, GNA11, GNAS, HEY1, HHIP, ID2, IFT27, IL5, IL6, KITLG, MAFF, MAP2K4, MAP2K6, MAP2K7, MAPK11, MAPK3, MAPK9, NCS1, PRKACA, PRKCA, PTCH1, PTCH2, RAC1, RAC2, RDH10, SDC2, STMN1, TCF7L2, TFAP2A, TGM2, WNT11* |
| Anti-benzo(a)pyrene-diol-epoxide | Chemical toxicant | *ADIPOQ, AKT3, CTNNB1* | 5.44 x 10^-13^ | *ADIPOQ, ADM, AKT3, APP, ARPC2, BDNF, CAV3, CCL11, CDK5R1, CTNNB1, EFNA1, FGF2, FRZB, FST, GAP43, GATA3, GLI2, GNAS, GSK3B, HAS2, HEY1, IL6, KITLG, MAFF, MAP2K4, MAP2K6, MAPK3, MEF2C, PIK3CA, PLA2G5, PPARG, RDH10, TAGLN, TGM2, TNF, TP53, WNT16* |
| Bupropion | Chemical drug | *ID2, IL6, TNF* | 5.67 x 10^-13^ | *AIF1, APOD, AQP1, AXIN2, BDNF, BMP4, CCL11, CDK5R1, CITED2, COL4A1, CTSB, ENPP2, FLOT1, FOXP1, FST, FYN, FZD1, GATA3, GHRL, GJA1, HEY1, ID2, IFT27, INPP5D, KCNJ2, KCNMA1, KCNQ3, KIT, KITLG, MAFF, MAPK11, MAPK3, PLD1, PRKCA, PTCH1, RBPJ, S1PR1, SCN1B, SLC9A3R1, SMARCA4, SNAP25, STAT5A, STAT5B, TAGLN, TFDP1, TGM2, TNF, TP53* |
| *DNMT1* | Enzyme | *PLA2G2A, TNF, TP53* | 6.15 x 10^-13^ | *ADM, APC, AQP1, ARFIP2, ATP6V0D1, AXIN2, BCCIP, BMP4, CAV3, CDC42, CDC42EP2, CPE, CTNNB1, DNM2, EFNA1, ENPP2, FST, GATA3, GJA1, GLI2, GNAS, GSN, HAS2, HEY1, IL5, INPP5D, KCNMA1, MAP2K6, MAP2K7, MAPK11, MAPK12, MYH10, PFN2, PPARG, PTCH1, PTCH2, RAC1, RDH10, S1PR1, SEMA3C, SOS1, STAT5A, STAT5B, STK4, SUFU, TAGLN, TCF7L2, TGM2, WASF2* |
| Aquaporin | Group | *FGF2, IL5, IL6* | 6.17 x 10^-13^ | *APP, AXIN2, BMP4, COL4A1, CTNNB1, CTSB, CTSH, FGF2, FZD3, GAP43, GATA3, GJA1, GLI2, GSK3B, HHIP, ID2, IL6, PPARG, PRKCB, PTCH1, PTCH2, SDC2, STMN1, TNF, TP53, WNT11* |
| U46619 | Chemical reagent | *ADIPOQ, AXIN2, CTNNB1, PPARG* | 7 x 10^-13^ | *ADM, ARPC2, BDNF, CALB1, CAV3, CCL11, CDK5R1, CITED2, COL4A1, EFNA1, EHF, FOXP1, FZD1, GAP43, GATA3, GLI2, GNA11, GNAS, GSN, HEY1, ID2, IFT27, IL5, IRF6, KCNJ2, KIT, KRT17, MAFF, MAPK11, MAPK3, PIK3R1, PLA2G6, PLD1, PRKCA, PTCH1, RAC1, RBPJ, RDH10, SDC2, SOS1, STAT5A, TAGLN, TCF7L2, TGM2, TNNC1, VAV2* |
| Aldose reductase  (*ADH4,*  *AKR1A1,*  *AKR1B1,*  *Akr1b10,*  *AKR1B10,*  *Akr1b7,*  *AKR1C1, AKR1C2,*  *AKR1C3,*  *AKR7A2,*  *AKR7A3,*  *ALDR2-4*) | Group | *IL5, IL6, S100B, TNF* | 8.26 x 10^-13^ | *ADIPOQ, ADM, APP, AXIN2, BDNF, CALB1, CAV3, CCL11, CTNNB1, CTSB, EFNA1, EHF, FGF2, FST, GAP43, GNAS, HAS2, ID2, IL5, IL6, IRF6, KCNJ2, KIT, KRT17, MAPK3, PLA2G6, PLD1, PRKCA, RBPJ, STAT5A, TAGLN, TGM2, TNF, TP53* |
| *NCOA6* | Transcription regulator | *BMP4, GATA3, KIT, KITLG, STAT5B, WNT11* | 8.93 x 10^-13^ | *ADIPOQ, APC, AQP1, ARFIP2, ATP6V0D1, AXIN2, BCCIP, BMP4, CAV3, CDC42, CDC42EP2, CPE, CTNNB1, DNM2, EFNA1, ENPP2, FST, GJA1, GNAS, GSN, IGF1R, IL5, IL6, KCNMA1, KITLG, MAP2K6, MAP2K7, MAPK11, MAPK12, MYH10, PFN2, PPARG, PTCH1, PTCH2, SEMA3C, SOS1, STAT5A, STAT5B, STK4, SUFU, TCF7L2, TGM2, TNF, TP53, WASF2* |
| Cep-1347 | Chemical drug | *BDNF, CAV3, IL6, KITLG, TNF, TP53* | 9.28 x 10^-13^ | *ADM, AKT3, APOD, APP, AQP1, BDNF, BMP4, CAV3, CCL11, CDK5R1, CTNNB1, CTSB, FGF2, FRZB, GAP43, GHRL, GSK3B, HAS2, ID2, IGF1R, IL6, IRF6, KCNJ2, MAP2K4, PLA2G6, PLD1, PPARG, PRKCA, PTCH1, SDC2, STMN1, TGM2, TNF, TNNC1, WNT16* |
| *CXCL16* | Cytokine | *ADIPOQ, AKT3, BDNF, CALB1, CDH3, COL4A1, F11R, GATA3, IL6, MAPK12, SLC9A3R1, TP53* | 9.3 x 10^-13^ | *BCCIP, BDNF, CALB1, CAV3, CDH3, CSK, CTNNB1, CTSB, DCN, EFNA1, EHF, ENPP2, FST, GAP43, GATA3, GJA1, GLI2, GNAS, HAS2, IGF1R, IL5, IL6, KIT, KRT17, MAP2K4, MEF2C, PIK3R1, PLA2G5, PLD1, PPARG, RBPJ, SEMA3C, STAT5A, STMN1, TFDP1, TGFB1I1, TGM2, TNF* |
| *RBX1* | Enzyme | *CTNNB1, EXT2, FRZB, HAND1, IGF1R, IL6, IRF6, KRT17, MEF2A, MEF2C, TDRD7, TNF, TP53, WNT8B* | 9.66E-13 | *ADIPOQ, AXIN2, BCCIP, BMP4, CCL11, CITED2, CSK, CTSB, DCN, DVL2, ENPP2, ERCC3, EXT2, FGF2, FRZB, FST, GATA3, GLI2, HAND1, HAS2, HEY1, ID2, IGF1R, IL5, INPP5D, KIT, KRT17, MAFF, PIK3R1, PPARG, RAC1, S1PR1, SEMA3C, STAT5A, STAT5B, TAGLN, TFDP1, TGFB1I1, WNT8B* |
| *CYLD* | Transcription regulator | *ADIPOQ, ADM, APP, BDNF, BMP4, CTNNB1, GAP43, ID2, IGF1R, IL6, MAPK3, PLA2G2A, PPARG, S100B, TNF, TP53* | 9.79 x 10^-13^ | *ADM, AGPS, AIF1, APOD, AQP1, AXIN2, BCCIP, BDNF, BMP4, CALB1, CAV3, CDH3, COL4A3BP, CSK, EHF, FGF2, FST, FYN, GAP43, GATA3, GLI2, GNAS, HAS2, ID2, IGF1R, IL6, IRF6, KIT, KRT17, MAP2K4, PIK3CA, PIK3R1, PLCG2, PLD1, PRKACA, PTCH1, RBPJ, RDH10, STAT5A, TAGLN, TDRD7, TFDP1, TGFB1I1, TGM2, TTC30B, TTLL1* |
| *RGS19* | Other | *APP, GJA1, IL6, S1PR1, TNF, TRAF6* | 1.05 x 10^-12^ | *ADIPOQ, ADM, AIF1, APC, APOD, ARF1, ARFIP2, ATP6V0D1, AXIN2, BMP4, CALB1, CAV3, CDC42EP1, CDC42EP2, CPE, CSK, DCN, DNM2, DVL2, EDA, EHF, EMP2, EXT2, F11R, FCER1A, FGF2, FRZB, FZD1, FZD3, GAP43, GHRL, GJA1, GLI2, GNA11, GSN, HAND1, HHIP, IFT27, IGF1R, IL5, IRF6, ITIH5, KCNB2, KCNQ3, KITLG, KYAT3, LGR4, LMO4, LYVE1, MAP2K4, MAPK10, MAPK12, MAPK9, MEF2A, MEF2C, MYH10, PAFAH1B1, PAFAH1B2, PDE2A, PFN2, PIK3R1, PIK3R3, PLA2G3, PLA2G5, PLA2G6, PLCG2, PLPP3, PPARG, PRKACA, PRKCA, PRKCB, PTCH2, RAC2, RDH10, S100B, SDC2, SEMA3C, SLC9A3R1, SMARCA4, SNX10, SOS1, ST14, STK4, SUFU, TAGLN, TBX6, TCF7L2, TGFB1I1, WASF2, WNT11, WNT8B* |
| *NCOA4* | Transcription regulator | *CCL11, IL5, IL6, PPARG, TNF* | 1.12 x 10^-12^ | *ADM, APC, APP, AQP1, ARFIP2, ATP6V0D1, BCCIP, BMP4, CDC42, CDC42EP2, COL4A1, CPE, CTNNB1, DCN, DNM2, E2F8, EFNA1, EHF, ENPP2, FST, FYN, GJA1, GNAS, GSN, IGF1R, KCNJ2, KCNMA1, KIT, MAP2K6, MAP2K7, MAPK11, MAPK12, MEF2C, MYH10, PFN2, PLA2G12A, PPARG, PRKCA, PTCH1, PTCH2, SEMA3C, SOS1, ST14, STAT5A, STAT5B, STK4, SUFU, TCF7L2, TGM2, TP53, WASF2* |
| Hormone | Chemical drug | *APP, CTNNB1, GATA3, SDC2, TP53* | 1.24 x 10^-12^ | *ADIPOQ, APC, APP, AQP1, ARFIP2, ATP6V0D1, BCCIP, BDNF, BMP4, CAPZB, CDC42, CDC42EP2, CPE, CTNNB1, DNM2, DPYSL2, EDNRA, EFNA1, ELF5, ENPP2, FGF2, FST, GAP43, GATA3, GJA1, GNAS, GSN, IGF1R, IL6, KCNMA1, MAP2K6, MAP2K7, MAPK11, MAPK12, MAPK3, MYH10, MYO10, PFN2, PTCH1, PTCH2, RAF1, SNAP25, SOS1, STAT5A, STAT5B, STK4, SUFU, TCF7L2, TGM2, TNF, TP53, WASF2* |
| *DUSP4* | Phosphatase | *ADM, AIF1, APP, DPYSL2, EXT1, GATA3, IL5, IL6, MAFF, MEF2C, TCF7L2, TNF* | 1.27 x 10^-12^ | *ADIPOQ, AKT3, APOD, APP, AQP1, ARPC2, BMP4, CAV3, CDK5R1, CITED2, CTNNB1, CTSB, DSE, EFNA1, FGF2, FRZB, FST, FZD1, GAP43, GNAS, GSK3B, HAS2, HEY1, ID2, IGF1R, IL6, KITLG, KRT17, MAFF, MAP2K6, MEF2C, PLD1, PPARG, PTCH1, RAC1, SDC2, STMN1, TAGLN, TDRD7, TGM2, TNNC1, WNT16* |
| *SNW1* | Transcription regulator | *ARF1, BDNF, CALB1, GNA11, IL5, IL6, KCNB2, MAFF, MAPK10, MEF2C, PAFAH1B1, PFN2, PPARG, PRKCB, TNF, TP53* | 1.35 x 10^-12^ | *APC, AQP1, ARFIP2, ATP6V0D1, BCCIP, BDNF, BMP4, CALB1, CAV3, CCL11, CDC42, CDC42EP2, CPE, CTNNB1, DNM2, EHF, ENPP2, FST, GAP43, GJA1, GNAS, GSN, HAS2, IGF1R, IL5, IL6, KCNMA1, KIT, KRT17, MAP2K6, MAP2K7, MAPK11, MAPK12, MYH10, PFN2, PLD1, PPARG, PTCH1, PTCH2, RBPJ, SEMA3C, SOS1, STAT5A, STAT5B, STK4, SUFU, TCF7L2, TGM2, TP53, WASF2* |
| Histrelin | Chemical drug | *ADM, BMP4, DCN, FRZB, FZD1, GJA1, IGF1R, IL6, PFN2, PLCG2, PPARG, TAGLN, TGM2, TP53* | 1.35 x 10^-12^ | *ADIPOQ, AKT3, APC, APP, ARF1, AXIN2, CALB1, CAPZB, CITED2, COL4A3BP, CPE, CTSH, DCN, DPYSL2, DVL2, EDNRA, EHF, ELF5, FRZB, FST, GHRL, GLI2, GNA11, GSK3B, GSN, IRF6, KCNJ2, KCNQ3, KIT, KRT5, LYVE1, MAP2K6, MAPK11, MAPK3, MEF2C, MYO10, PAFAH1B1, PFN2, PLA2G10, PLA2G6, RAF1, RBPJ, RDH10, SEMA3C, SMARCA4, STAT5A, STAT5B, STMN1, TFDP1, WNT16* |
| *CCNH* | Transcription regulator | *ADIPOQ, BDNF, CTNNB1, IL6, S1PR1, TNF* | 1.43 x 10^-12^ | *APC, AQP1, ARFIP2, ATP6V0D1, BCCIP, BMP4, CDC42, CDC42EP2, CPE, CTNNB1, DNM2, E2F8, EFNA1, ENPP2, EXT2, FRZB, FST, GJA1, GNAS, GSN, HAND1, IRF6, KCNMA1, KITLG, KRT17, MAFF, MAP2K6, MAP2K7, MAPK11, MAPK12, MEF2A, MEF2C, MYH10, PFN2, PPARG, PTCH1, PTCH2, SEMA3C, SOS1, STAT5A, STAT5B, STK4, SUFU, TCF7L2, TDRD7, TGM2, TNF, TP53, WASF2, WNT7A, WNT8B* |
| *MYLK* | Kinase | *AXIN2, IL6, TNF, WNT7A* | 1.48 x 10^-12^ | *ADIPOQ, ADM, AIF1, AIMP2, APC, APOD, ARF1, ARFIP2, ARPC2, ATP6V0D1, BDNF, CALB1, CAPRIN1, CAV3, CCL11, CLTC, CPE, CSK, CTSB, CTSH, DCN, DNM2, DPYSL2, DSE, DVL2, EDA, EDNRA, EFNA1, EHF, ELF5, ENPP6, ERCC3, EXT2, FGF2, FOXP1, FST, FZD3, GAP43, GATA3, GHRL, GNA11, GNAS, GSK3B, HAS2, HEYL, HHIP, IFT27, IGF1R, IL5, ITIH3, ITIH4, ITIH5, KCNJ2, KCNMB1, KCNQ3, KIT, KITLG, KRT17, KRT5, LMO4, MAFF, MAPK9, MYO10, NFIB, PADI1, PIK3R2, PIK3R3, PLA2G12A, PLA2G3, PLA2G6, PLCG1, PLCG2, PLD1, PRKACA, PRKCA, PTCH2, RAC1, RAC2, RBPJ, RDH10, S100B, SEMA3C, SLC35D1, SLC9A3R1, SMARCA4, SNX10, SOS1, STAT5A, STAT5B, STMN1, STMN3, SUFU, TFAP2A, TGM2, TMEM17, UCHL1, VAV1, VAV2, WASF2, WNT8B* |
| Pd 169316 | Chemical - kinase inhibitor | *ANXA7, APP, IL6, TNF* | 1.51 x 10^-12^ | *ADM, AIF1, AKT3, APP, BDNF, BMP4, CCL11, CDK5R1, CTNNB1, CTSH, DVL2, EFNA1, FGF2, FRZB, GAP43, GATA3, GLI2, GNAS, GSK3B, HAS2, HEY1, ID2, INPP5D, KITLG, MAP2K4, MAP2K6, MAPK3, PLA2G5, RAC1, RDH10, S1PR1, SDC2, SNAP25, STMN1, TAGLN, TNNC1, WNT11, WNT16* |
| *RAPGEF1* | Other | *APP, FGF2, IL5, IL6, TNF* | 1.62 x 10^-12^ | *ADIPOQ, APOD, APP, AQP1, ARPC2, BCCIP, BDNF, BMP4, CAV3, CDH3, CDK5R1, CSK, CTNNB1, CTSB, DCN, ENPP2, FGF2, FST, FZD1, GJA1, HAND1, HAS2, HEY1, IGF1R, IL5, IL6, MAFF, MAPK3, PIK3R1, PRKCA, PTCH1, RAC1, SEMA3C, TAGLN, TFDP1, TGFB1I1* |
| *GPER1* | G-protein coupled receptor | *CCL11, GATA3, IL5, IL6, TNF* | 1.69 x 10^-12^ | *ADM, APC, APP, AQP1, ARFIP2, ARPC2, ATP6V0D1, BCCIP, BMP4, CAV3, CCL11, CDC42, CDC42EP2, CDK5R1, CPE, DNM2, DSE, EFNA1, ENPP2, FZD1, GLI2, GSK3B, GSN, HAS2, HEY1, IL5, KCNMA1, KRT17, MAFF, MAP2K4, MAP2K6, MAP2K7, MAPK11, MAPK12, MAPK3, MEF2C, MYH10, PFN2, PLA2G5, PLD1, PPARG, PRKCA, PTCH1, PTCH2, RAC1, RDH10, SEMA3C, SOS1, STAT5A, STAT5B, STK4, STMN1, SUFU, TCF7L2, TDRD7, WASF2* |
| *CHRM5* | G-protein coupled receptor | *ATL1, HEY1, ID2, IL6, KCNJ2, MAFF, NME7, PLD1, RAPGEF2, TNF* | 1.89 x 10^-12^ | *ADIPOQ, BDNF, BMP4, FGF2, GNA11, HEYL, IGF1R, IL6, MAPK11, NME2, PAFAH1B1, PLD1, RAC1, S100B, SUFU, TNF, UPK1B, UPK2* |
| *CCND2* | Other | *ADIPOQ, APP, FGF2, GJA1, IL5, IL6, PLD1, SNAP25, TNF* | 2.07 x 10^-12^ | *CDK5R1, COL4A3BP, CTNNB1, CTSB, EDNRA, EFNA1, FGF2, GATA3, GJA1, GNAS, GSK3B, HAS2, IGF1R, IL5, IRF6, KCNJ2, KITLG, KRT17, MAP2K6, MAP2K7, MAPK3, PLA2G2A, PRKCA, PSEN2, RAC1, RAF1, S1PR1, STAT5A, TAGLN, TGM2, TNF, TP53* |
| Nimodipine | Chemical drug | *BDNF, BMP4, CITED2, EDA, FGF2, GJA1, GNAS, HAS2, IGF1R, KCNQ3, KIT, PADI1, PRKCA, PRKCB, SNAP25, TAGLN, TNF, TNNC1, TP53* | 2.07 x 10^-12^ | *ADIPOQ, AIF1, AKT3, APP, BDNF, CAV3, CCL11, CDK5R1, CTNNB1, CTSB, CTSH, EFNA1, FGF2, FRZB, FZD1, GAP43, GNAS, GSK3B, HAS2, IGF1R, IL5, KCNJ2, KITLG, MAP2K6, MEF2C, RAC1, SNAP25, STMN1, TAGLN, TGM2, TNF, WNT16* |
| Chondroitin sulfate E | Chemical - endogenous mammalian | *CCL11, CTSB, DCN, ENPP2, HEY1, IL5, IL6, SEMA3C, SNX10, TNF, TP53* | 2.1 x 10^-12^ | *ADIPOQ, APP, BDNF, BMP4, CALB1, CCL11, CPE, CTSB, CTSH, EDNRA, EFNA1, EHF, FGF2, FZD1, FZD3, GNAS, HEY1, HHIP, ID2, IGF1R, IL5, IL6, INPP5D, KIT, KRT17, MAPK3, PLD1, PPARG, PTCH1, PTCH2, RAC1, RBPJ, S1PR1, SDC2, STAT5A, TAGLN, TGM2, TNF, TP53, WNT11* |
| Ambroxol | Chemical drug | *FGF2, GSN, RAF1, SLC9A3R1, TP53* | 2.16 x 10^-12^ | *ADM, APP, BDNF, CALB1, CAV3, CCL11, CTNNB1, CTSB, EFNA1, EHF, FGF2, FST, GAP43, GNAS, HAS2, ID2, IGF1R, IL5, IL6, IRF6, KCNJ2, KIT, KRT17, MAPK3, PLA2G6, PLD1, PRKCA, RBPJ, STAT5A, TGM2, TNF, TP53* |
| Oog 41841 | Chemical drug | *ADIPOQ, IL6, PIK3R1, PPARG, TP53* | 2.47 x 10^-12^ | *ADIPOQ, ADM, ARF1, BDNF, BMP4, CCL11, CTNNB1, CTSB, FGF2, FST, GATA3, GHRL, GJA1, GLI2, HAS2, HEY1, IGF1R, IL5, MAP2K4, MAPK12, MAPK3, MEF2C, MYH10, PIK3CA, PIK3R1, PLA2G5, PLD1, PRKACA, RAC1, SMARCA4, SNAP25, SOS1, STMN1, TGM2, TP53, VAV2* |
| *DDX54* | Transcription regulator | *BMP4, GLI2, HEY1, HHIP, IL6, MEF2C, PTCH1, PTCH2, STMN3, TFDP1* | 2.52 x 10^-12^ | *APC, AQP1, ARFIP2, ATP6V0D1, BCCIP, BMP4, CDC42, CDC42EP2, CPE, DNM2, DPYSL2, E2F8, EDNRA, EFNA1, ELF5, ENPP2, FST, GAP43, GATA3, GJA1, GNAS, GSN, IGF1R, IL5, IL6, IRF6, KCNMA1, KRT17, MAP2K6, MAP2K7, MAPK11, MAPK12, MYH10, MYO10, PFN2, PTCH1, PTCH2, SEMA3C, SNAP25, SOS1, STAT5A, STAT5B, STK4, SUFU, TCF7L2, TFAP2A, TGM2, TNF, TP53, WASF2* |
| *EZH2* | Transcription regulator | *ADIPOQ, BDNF, IL6, PLA2G5, PPARG, TNF* | 2.62 x 10^-12^ | *APP, BDNF, BMP4, CCL11, CDC42, CDH3, COL4A1, CTNNB1, ENPP2, ERCC3, FGF2, FRZB, FST, FYN, GAP43, GATA3, GJA1, HAS2, HEY1, ID2, IGF1R, IL5, KIT, KRT17, MEF2A, MEF2C, PPARG, PRKACA, RBPJ, S1PR1, STAT5A, STAT5B, TAGLN, TFDP1, TNF, TNNC1, TP53* |
| *CYCLIN B*  (*CCNB1-3*) | Group | *ADIPOQ, AIF1, APP, BDNF, CAV3, CTNNB1, HEY1, IL6, PPARG, PSEN2, PTCH1, TNF, TP53* | 2.72 x 10^-12^ | *ACADVL, AKT3, APP, AXIN2, BDNF, CAV3, CDC42EP2, CDH3, CDK5R1, COL4A1, COL4A3BP, CTNNB1, CTSB, CTSH, EDNRA, EFNA1, ENPP2, ERCC3, FST, FYN, GSK3B, GSN, HAS2, ID2, IGF1R, IL5, KCNMA1, KIT, KITLG, MAP2K6, MAP2K7, MAPK12, MAPK3, MEF2C, MYH10, PAFAH1B2, PDE2A, PIK3R3, PRKCA, PSEN2, PTCH1, RBPJ, ST14, TAGLN, TCF7L2, TFDP1, TGFB1I1, TGM2, TNF, TNNC1, WNT2, WNT7A* |
| Thyroid hormone | Chemical - endogenous mammalian | *CTNNB1, DVL2, ENPP2, TP53* | 2.76 x 10^-12^ | *ACADVL, ADM, CALB1, CAV3, CDK5R1, COL4A3BP, CPE, CTNNB1, CTSB, EDNRA, FZD3, FZD6, GAP43, GHRL, GJA1, GLI2, HAS2, ID2, IRF6, KCNJ2, KRT5, MAP2K7, MEF2C, PLA2G2A, PLA2G5, PLCG1, PRKCA, PSEN2, RAF1, RDH10, ST14, STAT5B, TFDP1, TNNC1, WNT11, WNT16, WNT2, WNT2B, WNT7A, WNT8B* |
| Pilocarpine | Chemical drug | *BDNF, IL6, TNF, TP53* | 3.11 x 10^-12^ | *ADIPOQ, APP, ARPC2, AXIN2, BDNF, BMP4, CCL11, CDH3, CDK5R1, COL4A1, CTSB, FGF2, FST, FZD1, FZD3, GAP43, GATA3, GLI2, HAS2, HEY1, HHIP, ID2, IGF1R, IL6, KCNQ3, MAFF, MAPK3, PPARG, PTCH1, PTCH2, RAC1, TGM2, TNF, TNNC1, WNT11* |
| Estriol | Chemical - endogenous mammalian | *ADIPOQ, CTNNB1, PIK3R1, TNF* | 3.25 x 10^-12^ | *APC, AQP1, ARFIP2, ARPC2, ATP6V0D1, BCCIP, BDNF, BMP4, CAV3, CCL11, CDC42, CDC42EP2, CDK5R1, COL4A1, CPE, CTSB, DNM2, EFNA1, ENPP2, FST, FZD1, GJA1, GNAS, HAS2, HEY1, KCNJ2, KCNMA1, MAFF, MAP2K6, MAP2K7, MAPK11, MAPK12, MAPK3, MEF2C, MYH10, PFN2, PIK3R1, PPARG, PRKCA, PTCH2, RAC1, SEMA3C, SOS1, STAT5A, STAT5B, STK4, SUFU, TCF7L2, TGM2, TNF, TP53, WASF2* |
| *RGL4* | Other | *CTNNB1, EXT2, FGF2, FRZB, HAND1, TP53, WNT8B* | 3.63 x 10^-12^ | *AKT3, APOD, APP, AQP1, BDNF, CDK5R1, CTNNB1, CTSB, DSE, EFNA1, FGF2, FRZB, GAP43, GATA3, GJA1, GNAS, GSK3B, HAS2, IGF1R, IL6, KITLG, KRT17, MAP2K6, MAPK3, MEF2C, PLD1, PPARG, PTCH1, RAC1, STMN1, TAGLN, TDRD7, TGM2, TNF, WNT16* |
| Ly-2510924 | Biologic drug | *APC, KCNJ2, TNF* | 3.66 x 10^-12^ | *ADIPOQ, ADM, APP, ARPC2, AXIN2, BMP4, CAV3, CDK5R1, COL4A1, CTNNB1, FGF2, FST, FZD3, GAP43, GATA3, GJA1, GLI2, HEY1, HHIP, IFT27, IGF1R, IL5, IL6, MAFF, PTCH1, PTCH2, RAC1, RDH10, SDC2, TCF7L2, TGM2, TNNC1, WNT11* |
| Dov-102,677 | Chemical drug | *IL6, MAPK3, TNF* | 4.63 x 10^-12^ | *ADIPOQ, ADM, AIF1, BDNF, CAV3, CCL11, CDK5R1, CTNNB1, FST, FYN, GAP43, GATA3, GHRL, GJA1, GLI2, HAS2, HEY1, ID2, IGF1R, IL6, KCNJ2, KCNMA1, MAPK11, MAPK3, PPARG, RBPJ, RDH10, S1PR1, TAGLN, TFDP1, TNF* |
| D-methyl-phenidate | Chemical drug | *CTNNB1, DVL2, GSK3B* | 4.63 x 10^-12^ | *ADIPOQ, ADM, AIF1, BDNF, CAV3, CCL11, CDK5R1, CTNNB1, FST, FYN, GAP43, GATA3, GHRL, GJA1, GLI2, HAS2, HEY1, ID2, IGF1R, IL6, KCNJ2, KCNMA1, MAPK11, MAPK3, PPARG, RBPJ, RDH10, S1PR1, TAGLN, TFDP1, TNF* |
| *TBP* | Transcription regulator | *APP, IL6, TNF* | 4.65 x 10^-12^ | *AKT3, APC, AQP1, ARFIP2, ATP6V0D1, BCCIP, BMP4, CDC42, CDC42EP2, CDH3, COL4A1, CPE, CTNNB1, DNM2, DPYSL2, EDNRA, EFNA1, ELF5, ENPP2, F11R, FST, GATA3, GJA1, GNAS, GSN, KCNMA1, MAP2K6, MAP2K7, MAPK11, MAPK12, MAPK3, MYH10, MYO10, PFN2, PLD1, PTCH1, PTCH2, SEMA3C, SLC9A3R1, SNAP25, SOS1, STAT5A, STAT5B, STK4, SUFU, TCF7L2, TFDP1, TGM2, WASF2* |
| Flecainide | Chemical drug | *AXIN2, CTNNB1, PPARG* | 4.69 x 10^-12^ | *ADIPOQ, ADM, APOD, APP, AQP1, BMP4, CAV3, CCL11, CDK5R1, CTSB, EDNRA, FGF2, FLOT1, FZD1, GAP43, GNA11, IGF1R, IL5, MAFF, MAP2K4, MAP2K6, MAP2K7, MAPK3, MAPK9, PLCG1, PRKACA, PRKCA, PTCH1, RAC1, SCN1B, SLC9A3R1, TAGLN, TMEM100* |
| *PAK3* | Kinase | *APP, PSEN2, TP53* | 4.76 x 10^-12^ | *APOD, APP, AQP1, BDNF, BMP4, CAV3, CCL11, CDC42EP1, CHN1, CITED2, COL4A1, CTNNB1, CTSB, CTSH, DCN, EFNA1, FGF2, GAP43, GLI2, GNAS, HAS2, IGF1R, IL6, KITLG, MAP2K6, MEF2C, PLD1, PPARG, PTCH1, RAC1, SDC2, TAGLN, TGM2, TNNC1, WNT7A* |
| Mazindol | Chemical drug | *HAS2, IL6, TNF* | 4.84 x 10^-12^ | *ADIPOQ, ADM, AIF1, BDNF, CAV3, CCL11, CDK5R1, CTNNB1, FST, FYN, GAP43, GATA3, GHRL, GJA1, GLI2, HAS2, HEY1, ID2, IGF1R, IL6, KCNJ2, KCNMA1, MAPK11, MAPK3, PPARG, RBPJ, RDH10, S1PR1, TAGLN, TFDP1, TNF* |
| *MIR-192* | MicroRNA | *APP, IGF1R, PRKCA, PRKCB, TNF* | 4.87 x 10^-12^ | *AXIN2, BMP4, COL4A1, CTSB, EDNRA, FCER1A, FZD3, GAP43, GATA3, GJA1, GLI2, HHIP, ID2, IGF1R, IL6, INPP5D, PLCG1, PLCG2, PPARG, PTCH1, PTCH2, SDC2, STMN1, TNF, TP53, WNT11* |
| *NPPC* | Other | *CITED2, FOXP1, FST, GATA3, IL6, PIK3CA, TNF* | 5.29 x 10^-12^ | *ADIPOQ, ADM, APOD, APP, AQP1, BDNF, BMP4, CAV3, CCL11, CDK5R1, CTNNB1, DSE, FGF2, FZD1, GAP43, GATA3, GJA1, HAS2, ID2, IGF1R, IL5, IL6, IRF6, KCNJ2, KRT17, PLA2G6, PLD1, PPARG, PRKCA, PTCH1, RAC1, TDRD7, TNF, TP53* |
| *CD72* | Trans-membrane receptor | *BDNF, FRZB, GJA1, KCNJ2, MEF2A, MEF2C, PLA2G2A, TNNC1* | 5.48 x 10^-12^ | *APOD, APP, AQP1, ARPC2, BDNF, BMP4, CCL11, CDK5R1, CITED2, CTNNB1, FGF2, FOXP1, FST, FYN, GAP43, GATA3, GJA1, GLI2, HEY1, IGF1R, IL5, MAFF, MAP2K4, MEF2C, PIK3CA, PLA2G5, PTCH1, STAT5A, STAT5B, STMN1* |
| *PNMT* | Enzyme | *ADM, APP, CDC42, DCN, FGF2, GJA1, IGF1R, IL6, KITLG, TNF* | 5.62 x 10^-12^ | *ADM, AQP1, ARPC2, AXIN2, BDNF, BMP4, CDK5R1, CTNNB1, CTSB, EFNA1, FGF2, FST, GAP43, GHRL, HEY1, ID2, IL5, IRF6, KCNJ2, MAFF, PAFAH1B1, PLA2G2A, PLA2G5, PLA2G6, PLD1, PRKCA, PRKCB, RAF1, SEMA3C, TGM2, TNF, TP53* |
| Denatonium benzoate | Chemical drug | *CTNNB1, EXT2, FRZB, HAND1, HEY1, IL6, IRF6, KRT17, MEF2C, TAGLN, TP53, WNT8B* | 5.88 x 10^-12^ | *ADIPOQ, AKT3, APOD, APP, AQP1, AXIN2, CAV3, CCL11, CDH3, CDK5R1, COL4A1, DSE, ENPP2, FRZB, FYN, FZD1, FZD3, GAP43, GATA3, GLI2, GSK3B, GSN, HEY1, HHIP, ID2, INPP5D, KIT, KRT17, MAPK3, NME2, PIK3CA, PIK3R2, PLD1, PPARG, PRKCA, PTCH2, S1PR1, SDC2, SEMA3C, SNX10, STMN1, TDRD7, WNT11, WNT16* |
| *LAMININ1*  *(LAMA1, LAMA5, LAMB1, LAMC1*) | Complex | *IL6, MAPK9, MEF2C, PPARG, TNF, TP53* | 6.15 x 10^-12^ | *ADIPOQ, AXIN2, CTNNB1, DOCK1, FST, FYN, GATA3, GLI2, HAS2, HEY1, IL5, IL6, INPP5D, KCNMA1, PLA2G5, RAC1, RAC2, S1PR1, TAGLN, TNF, TP53, VAV1* |
| *MDC1* | Other | *FCER1A, IL5, IL6, KITLG, PRKACA, PTCH1, TNF* | 6.24 x 10^-12^ | *ACADVL, AKT3, APP, AXIN2, CDH3, COL4A1, CTNNB1, CTSB, CTSH, EFNA1, ERCC3, FYN, GSK3B, GSN, HAS2, ID2, IGF1R, IL5, IL6, KCNMA1, KIT, KRT17, MAP2K6, MAP2K7, MAPK12, MYH10, PAFAH1B2, PDE2A, PPARG, PRKCA, PSEN2, PTCH1, RBPJ, ST14, STAT5A, STAT5B, STMN1, TCF7L2, TFDP1, TGFB1I1, TGM2, TNF, TP53, WNT2, WNT7A* |
| *HERC2* | Enzyme | *CTNNB1, GSK3B, IL6, MAPK3, TNF* | 6.24 x 10^-12^ | *ACADVL, AKT3, APP, AXIN2, CDH3, COL4A1, CTNNB1, CTSB, CTSH, EFNA1, ERCC3, FYN, GSK3B, GSN, HAS2, ID2, IGF1R, IL5, IL6, KCNMA1, KIT, KRT17, MAP2K6, MAP2K7, MAPK12, MYH10, PAFAH1B2, PDE2A, PPARG, PRKCA, PSEN2, PTCH1, RBPJ, ST14, STAT5A, STAT5B, STMN1, TCF7L2, TFDP1, TGFB1I1, TGM2, TNF, TP53, WNT2, WNT7A* |
| *SLIRP* | Other | *BDNF, IL6, MAP2K4, TNF* | 6.4 x 10^-12^ | *ACADVL, ADIPOQ, APC, APP, AQP1, ARFIP2, ATP6V0D1, BCCIP, BDNF, BMP4, CALB1, CDC42, CDC42EP2, CPE, CTNNB1, DNM2, EFNA1, ENPP2, FST, GAP43, GATA3, GJA1, GNAS, GSN, IGF1R, KCNJ2, KCNMA1, KYAT3, MAP2K6, MAP2K7, MAPK11, MAPK12, MAPK3, MEF2C, MYH10, PFN2, PLA2G2A, PLCG1, PPARG, PRKCA, PTCH1, PTCH2, RAF1, SOS1, STAT5A, STAT5B, STK4, SUFU, TCF7L2, TGM2, TP53, WASF2* |
| Chondroitin sulfate B | Chemical - endogenous mammalian | *FGF2, MAPK3, TNF, TP53* | 6.46 x 10^-12^ | *ADIPOQ, AIF1, BDNF, CALB1, CCL11, COL4A1, CPE, CTNNB1, CTSB, CTSH, EFNA1, EHF, FGF2, FST, FYN, FZD1, GAP43, GJA1, GNAS, HAS2, HHIP, IGF1R, IL5, KIT, KRT17, MAPK11, MAPK3, PLD1, PPARG, RAC1, RBPJ, STAT5A, TGM2, TNF, TP53* |
| *GATA2* | Transcription regulator | *CTNNB1, DVL2, IL6, TNF, TOLLIP, TP53, WNT7A* | 6.78 x 10^-12^ | *ADIPOQ, ADM, AKT3, APP, BDNF, CAV3, CDC42, CDK5R1, CTNNB1, CTSB, EDNRA, FCER1A, FGF2, FRZB, GAP43, GJA1, GLI2, GSK3B, IL5, IL6, KIT, MAPK3, PPARG, PRKCA, RDH10, S1PR1, STAT5A, STMN1, TGM2, TNF, TP53, WNT16, ZNF750* |
| Glabridin | Chemical - endogenous non-mammalian | *APP, ARPC2, CTSB, ID2, PLA2G6, PLD1, TNF, TP53* | 6.96 x 10^-12^ | *ADIPOQ, ADM, AGPS, APP, BDNF, CALB1, CAV3, CCL11, CTSB, EFNA1, EHF, FGF2, FST, GAP43, GNAS, HAS2, ID2, IL6, IRF6, KCNJ2, KIT, KRT17, MAPK3, PIK3CA, PLA2G6, PLD1, PRKCA, RBPJ, RDH10, STAT5A, TGM2, TNF, TP53, TTC30B, TTLL1* |
| *C8orf4* | Other | *GJA1, HAND1, KCNMB1, MEF2C, PLA2G2A, TAGLN, TNNC1* | 7.34 x 10^-12^ | *ADM, APP, CTNNB1, CTSB, FGF2, GAP43, GHRL, GJA1, HAS2, ID2, IGF1R, IL5, IL6, IRF6, KCNJ2, MAPK3, PLA2G6, PLD1, PPARG, PRKCA, TNF* |
| U0126 | Chemical - kinase inhibitor | *APP, BDNF, CTNNB1, IL6, NCS1, TCF7L2, TP53* | 7.37 x 10^-12^ | *ADIPOQ, AQP1, AXIN2, BDNF, CCL11, CDK5R1, COL4A1, CTNNB1, EHF, FGF2, GJA1, GSK3B, GSN, IL5, IL6, MAPK3, PLD1, PPARG, PRKCB, RAC1, TCF7L2, TGM2, TNF, UPK2* |
| Benfotiamine | Chemical drug | *APP, IL6, PPARG, RAC1, TNF* | 7.51 x 10^-12^ | *ADM, APP, BDNF, CALB1, CAV3, CCL11, CTNNB1, CTSB, EFNA1, EHF, FGF2, FST, GAP43, GNAS, HAS2, ID2, IL5, IL6, IRF6, KCNJ2, KIT, KRT17, MAPK3, PLA2G6, PLD1, PRKCA, RBPJ, STAT5A, TGM2, TNF, TP53* |
| Cadherin  (*CDH1-20)* | Group | *FGF2, IL6, PPARG, TNF, TP53* | 7.92 x 10^-12^ | *ADIPOQ, AKT3, AXIN2, CDC42, CDH3, COL4A1, CTNNB1, FZD3, GAP43, GATA3, GJA1, GLI2, HHIP, ID2, IL5, PIK3R1, PPARG, PTCH1, PTCH2, RAC1, SDC2, SLC9A3R1, TNF, TP53, WNT11* |
| *Salmonella enterica* serotype *abortus equi* lipo-polysaccharide | Chemical toxicant | *APP, BDNF, CDC42, FGF2, GAP43, GATA3, GJA1, IL5, IL6, PTCH1, TNF, TP53* | 7.96 x 10^-12^ | *AKT3, APOD, APP, AQP1, ATL1, BDNF, BMP4, CAV3, CCL11, CDK5R1, FGF2, FRZB, GAP43, GJA1, GSK3B, HEY1, ID2, IGF1R, IL5, IL6, KCNJ2, MAFF, MAPK3, NME7, PLD1, PPARG, PTCH1, RAPGEF2, SDC2, STMN1, TNF, TNNC1, WNT16* |
| Chloramine | Chemical toxicant | *APP, BDNF, CDK5R1, GAP43, GSK3B, PPARG, RAC1, SNAP25, TFAP2A, TNF* | 8.93 x 10^-12^ | *ADM, APP, BDNF, CALB1, CAV3, CCL11, CTNNB1, CTSB, EFNA1, EHF, FGF2, FST, GAP43, GNAS, HAS2, ID2, IL5, IL6, IRF6, KCNJ2, KIT, KRT17, MAPK3, PLA2G6, PLD1, PRKCA, RBPJ, STAT5A, TGM2, TNF, TP53* |
| *PRKX* | Kinase | *BDNF, CTNNB1, GAP43, GJA1, TP53, WNT2, WNT7A* | 8.99 x 10^-12^ | *APP, BDNF, BMP4, CDC42, COL4A1, CTNNB1, EFNA1, FST, GATA3, GHRL, GLI2, HAS2, HEY1, IGF1R, IL6, INPP5D, KITLG, MAP2K6, MEF2C, PLA2G2A, PLD1, PPARG, PRKACA, RAC1, S1PR1, TGM2, WNT11* |
| *ARHGEF1* | Other | *DNM2, FGF2, IL5, IL6, PPARG, TNF, TP53* | 9.12 x 10^-12^ | *AIF1, AKT3, APOD, APP, AQP1, BMP4, CALB1, CAV3, CCL11, CDK5R1, CTNNB1, EHF, FGF2, FRZB, FST, GJA1, GSK3B, GSN, IGF1R, KIT, KITLG, KRT17, MAP2K6, MAPK3, MEF2C, PIK3CA, PIK3R1, PLD1, PTCH1, RBPJ, SDC2, SOS1, STAT5A, STMN1, TAGLN, TNNC1, VAV2, WNT16* |
| Sibutramine | Chemical drug | *BDNF, CTNNB1, DPP6, FGF2, GSK3B, PRKCA, SCN1B* | 9.14 x 10^-12^ | *ADIPOQ, AIF1, APP, AQP1, BDNF, BMP4, CAV3, CCL11, CDK5R1, CTNNB1, FLOT1, FST, FYN, GATA3, GHRL, GJA1, GLI2, HEY1, ID2, IGF1R, KCNJ2, KCNMA1, MAFF, MAPK11, MAPK3, PPARG, RBPJ, RDH10, S1PR1, SCN1B, SLC9A3R1, TAGLN, TFDP1, TMEM100* |
| Nor-gestimate | Chemical drug | *APP, CAV3, CTNNB1, CTSB, DCN, FGF2, HAS2, IL6, PPARG, RAC1, STMN1, TNF, TP53* | 9.29 x 10^-12^ | *APC, AQP1, ARFIP2, ATP6V0D1, BCCIP, BMP4, CDC42, CDC42EP2, CPE, CTNNB1, DNM2, DPYSL2, EDNRA, EFNA1, ELF5, ENPP2, FST, GATA3, GJA1, GNAS, GSN, IGF1R, KCNMA1, MAP2K6, MAP2K7, MAPK11, MAPK12, MYH10, MYO10, PFN2, PTCH1, PTCH2, S100B, SEMA3C, SNAP25, SOS1, STAT5A, STAT5B, STK4, SUFU, TCF7L2, TGM2, TNF, TP53, WASF2* |
| *PKN1* | Kinase | *AXIN2, GATA3, HAS2, IL5, PTCH1, TP53* | 9.46 x 10^-12^ | *ADIPOQ, ADM, AIF1, APC, AQP1, ARFIP2, ATP6V0D1, BCCIP, BMP4, CDC42, CDC42EP2, COL4A1, CPE, CTSB, DNM2, EFNA1, ENPP2, FST, FYN, GJA1, GLI2, GNAS, GSN, KCNJ2, KCNMA1, MAP2K6, MAP2K7, MAPK12, MAPK3, MEF2C, MYH10, PFN2, PLD1, PPARG, PRKCA, PTCH1, PTCH2, RAC1, SEMA3C, SOS1, STAT5A, STAT5B, STK4, SUFU, TAGLN, TCF7L2, TGM2, TP53, WASF2* |
| *UBE2M* | Enzyme | *APP, IL6, MAPK3, TNF* | 9.56 x 10^-12^ | *APC, APP, AQP1, ARFIP2, ATP6V0D1, BCCIP, BMP4, CCL11, CDC42, CDC42EP2, CPE, CTNNB1, DNM2, EFNA1, ENPP2, FST, GAP43, GJA1, GNAS, GSN, IGF1R, IL5, IL6, KCNMA1, LGR4, MAP2K6, MAP2K7, MAPK11, MAPK12, MYH10, PFN2, PRKACA, PTCH1, PTCH2, SEMA3C, SOS1, STAT5A, STAT5B, STK4, SUFU, TCF7L2, TGM2, TNF, TP53, WASF2* |
| *TGFBR* | Group | *BDNF, IL6, TNF, TP53* | 1.01 x 10^-11^ | *APP, AXIN2, BDNF, BMP4, CCL11, CTNNB1, CTSB, DCN, EFNA1, FCER1A, FGF2, FOXP1, FZD1, GLI2, GNAS, HAS2, HEY1, ID2, IGF1R, INPP5D, KIT, KITLG, MAP2K4, MAP2K6, MEF2C, PLA2G5, PLPP3, PTCH1, RAC1, S1PR1, STMN1, TAGLN, TGM2, TP53, VAV1, WNT11* |
| *PDLIM1* | Transcription regulator | *IL5, IL6, KIT, KITLG* | 1.01 x 10^-11^ | *AXIN2, BDNF, BMP4, CALB1, CAV3, CCL11, COL4A1, CTNNB1, EFNA1, EHF, FST, FZD3, GAP43, GATA3, GJA1, GLI2, GNAS, HAS2, HHIP, ID2, IL5, IL6, KIT, KRT17, PLD1, PTCH1, PTCH2, RBPJ, SDC2, STAT5A, TGM2, TNF, WNT11* |
| *CNR1* | G-protein coupled receptor | *BDNF, CPE, FZD3, FZD6, MAPK3, PLA2G2A, PLA2G5, ST14, STAT5B, TFDP1* | 1.04 x 10^-11^ | *ADIPOQ, BDNF, BMP4, FGF2, HEYL, IGF1R, IL6, NME2, PAFAH1B1, PLD1, RAC1, S100B, SUFU, TNF, UPK1B, UPK2* |
| *RELN* | Peptidase | *APP, BDNF, PIK3R1, PRKCA, SOS1* | 1.05 x 10^-11^ | *ADM, AIF1, APP, BDNF, CAV3, CCL11, CITED2, COL4A1, CTSB, DNM2, EDA, FGF2, FYN, GATA3, HAS2, HEY1, HEYL, ID2, IFT27, IL5, KCNMA1, KCNQ3, KIT, LYVE1, MAP2K4, MAPK11, MEF2C, PADI1, PLA2G5, RAC1, RDH10, SNAP25, STMN1, TAGLN, TCF7L2, TGM2* |
| *MYZAP* | Other | *APP, CCL11, GATA3, IL5, IL6, PLCG1, TNF* | 1.1 x 10^-11^ | *APP, ARPC2, AXIN2, BDNF, BMP4, CAV3, CCL11, CDK5R1, COL4A1, FGF2, FST, FZD3, GAP43, GATA3, GJA1, GLI2, HEY1, HHIP, ID2, IFT27, IGF1R, IL6, MAFF, PPARG, PTCH1, PTCH2, RAC1, TCF7L2, TGM2, TNF, TNNC1, WNT11* |
| Allyl iso-thiocyanate | Chemical toxicant | *IL6, PPARG, TNF* | 1.11 x 10^-11^ | *ADIPOQ, ADM, AIF1, APOD, APP, AQP1, BDNF, BMP4, CAV3, CCL11, CTNNB1, CTSB, CTSH, FST, FZD1, GJA1, HAS2, ID2, IGF1R, IL5, IRF6, KCNJ2, PLA2G6, PLCG2, PPARG, PRKCA, PTCH1, RAC1, SNAP25, TDRD7, TP53* |
| *THRB* | Ligand-dependent nuclear receptor | *IL6, PLA2G5, TNF* | 1.3 x 10^-11^ | *CTNNB1, FZD1, FZD6, IGF1R, IL6, MAPK3, PPARG, STAT5B, TNF, TP53, WNT11, WNT16, WNT2, WNT2B, WNT7A, WNT8B* |
| Piracetam | Chemical drug | *IL6, NCS1, TNF* | 1.35 x 10^-11^ | *ADIPOQ, BDNF, CCL11, CTNNB1, CTSB, DOCK1, FGF2, FYN, GAP43, IL6, PLA2G5, PPARG, RAC1, RAC2, TNF, TP53, VAV1* |
| *TAOK2* | Kinase | *ADIPOQ, IL6, TNF* | 1.37 x 10^-11^ | *AIF1, APOD, APP, AQP1, BDNF, BMP4, CAV3, CCL11, CDK5R1, CTNNB1, EFNA1, FYN, GAP43, GNAS, HAND1, HAS2, IL6, KITLG, MAP2K4, MAP2K6, MAPK11, MAPK3, MAPK9, MEF2C, PTCH1, SDC2, STK4, TAGLN, TGM2, TNF, TNNC1* |
| *WNT2* | Cytokine | *AXIN2, BMP4, MEF2C* | 1.51 x 10^-11^ | *APP, AXIN2, BMP4, COL4A1, CTNNB1, DVL2, FZD3, GAP43, GATA3, GJA1, GLI2, GSK3B, HHIP, ID2, IFT27, IGF1R, PPARG, PTCH1, PTCH2, RAC1, SDC2, TCF7L2, TNF, WNT11* |
| Ro 32-0432 | Chemical - kinase inhibitor | *IL5, IL6, TNF* | 1.64 x 10^-11^ | *ADIPOQ, ADM, APOD, APP, AQP1, BDNF, BMP4, CDK5R1, CTNNB1, DSE, EFNA1, FGF2, GAP43, GATA3, GJA1, GNAS, ID2, IGF1R, IRF6, KCNJ2, KITLG, KRT17, MAP2K6, MEF2C, PLA2G6, PLD1, PPARG, PRKCA, PTCH1, TAGLN, TDRD7, TGM2* |
| *MARK1* | Kinase | *GJA1, HHIP, PTCH1* | 1.71 x 10^-11^ | *ADIPOQ, AIF1, APOD, APP, AQP1, ARPC2, AXIN2, BMP4, CAV3, CCL11, CDK5R1, COL4A1, CTSB, FGF2, FST, FYN, FZD3, GAP43, GATA3, GNAS, HEY1, HHIP, ID2, IFT27, IL5, MAFF, MAPK11, MAPK3, PRKCA, PTCH1, PTCH2, RAC1, RDH10, SDC2, TCF7L2, TGM2, WNT11* |
| *WWP1* | Enzyme | *ADM, IL6, TNF* | 1.76 x 10^-11^ | *ADM, AXIN2, BDNF, BMP4, CDC42EP2, COL4A1, CTNNB1, EFNA1, ERCC3, FGF2, FST, GAP43, GJA1, GNAS, HAS2, HEY1, ID2, INPP5D, KIT, KRT5, MYH10, PIK3R3, PPARG, RAC1, S1PR1, STMN1, TNF, TP53, WNT11, WNT2, WNT7A* |
| Poly-saccharide | Chemical - endogenous mammalian | *ADIPOQ, AQP1, BDNF, EDNRA, FGF2, FZD1, GJA1, HAS2, IL6, TNF* | 1.85 x 10^-11^ | *ADIPOQ, ADM, CAPRIN1, CAV3, CCL11, CITED2, COL4A1, CTNNB1, CTSB, DVL2, EFNA1, GATA3, GJA1, GLI2, GNAS, HAS2, ID2, IL5, IL6, KITLG, MAP2K4, MAP2K6, MEF2C, PLA2G5, RAC1, RDH10, ST14, STMN1, TAGLN, TGM2, TNF, TP53* |
| Benomyl | Chemical toxicant | *ADIPOQ, ADM, CCL11, GJA1, IL6, TNF, TP53* | 2.04 x 10^-11^ | *ACADVL, APC, AQP1, ARFIP2, ATP6V0D1, BCCIP, BDNF, BMP4, CDC42, CDC42EP2, CPE, CTNNB1, DNM2, EFNA1, ENPP2, FST, GJA1, GNAS, GSK3B, GSN, IGF1R, IL6, KCNMA1, MAP2K6, MAP2K7, MAPK11, MAPK12, MYH10, PFN2, PPARG, PTCH1, PTCH2, SEMA3C, SOS1, STAT5A, STAT5B, STK4, SUFU, TAGLN, TCF7L2, TGM2, WASF2* |
| Zolpidem | Chemical drug | *BDNF, GATA3, IL5, IL6, TNF, TP53* | 2.1 x 10^-11^ | *ADM, AIF1, APOD, APP, AQP1, BDNF, BMP4, CDK5R1, EDNRA, FGF2, FLOT1, FYN, GAP43, GJA1, GNA11, GSN, HAS2, IL5, KCNJ2, KITLG, MAFF, MAP2K4, MAP2K6, MAP2K7, MAPK11, MAPK3, MAPK9, MEF2C, PLCG1, PRKACA, PRKCA, PTCH1, RAC2, SCN1B, SLC9A3R1, TAGLN, TMEM100, TNF* |
| *MYOD1* | Transcription regulator | *ADIPOQ, APP, AQP1, BDNF, CTNNB1, FGF2, FYN, GNA11, GSK3B, IL6, MAPK3, PPARG, TNF, TP53* | 2.38 x 10^-11^ | *APOD, AQP1, BDNF, BMP4, CAV3, COL4A1, CTNNB1, E2F8, EFNA1, ENPP2, FYN, GJA1, GNAS, HAS2, ID2, IL6, KCNJ2, KITLG, MAFF, MAP2K6, MEF2A, MEF2C, PLA2G2A, PPARG, PRKACA, PTCH1, S100B, SCN1B, TAGLN, TGM2, TNF, TNNC1, WNT7A* |
| p-chloro-mercuri-benzene-sulfonate | Chemical toxicant | *COL4A1, CTNNB1, ENPP2, FYN, ID2, MEF2A, MEF2C, PRKACA, SDC2, TNNC1* | 2.55 x 10^-11^ | *ADIPOQ, ADM, APP, AQP1, BDNF, CAV3, CDK5R1, CTNNB1, CTSB, DSE, EFNA1, GATA3, GLI2, GNAS, HAS2, IFT27, IGF1R, IL5, IL6, KITLG, KRT17, MAP2K6, MAPK3, MEF2C, PLD1, PPARG, RAC1, RDH10, TAGLN, TCF7L2, TDRD7, TGM2* |
| Nystatin | Chemical drug | *BMP4, CTSB, FGF2, GJA1, IL6, INPP5D, MEF2C, NME2, PLPP3, PRKCA, TNF, TP53* | 3.1 x 10^-11^ | *AGPS, APP, ARPC2, CALB1, CAV3, CCL11, CDK5R1, CTNNB1, EFNA1, EHF, FGF2, FST, FYN, GAP43, GNAS, HAS2, HEY1, IL6, IRF6, KIT, KRT17, MAFF, PIK3CA, PLD1, PPARG, RBPJ, RDH10, STAT5B, TGM2, TNF, TP53, TTC30B, TTLL1* |
| Ethynodiol diacetate | Chemical drug | *CCL11, CTSB, IL6, TNF* | 3.15 x 10^-11^ | *APC, AQP1, ARFIP2, ATP6V0D1, BCCIP, BMP4, CDC42, CDC42EP2, CPE, CTNNB1, DNM2, DPYSL2, EDNRA, EFNA1, ELF5, ENPP2, FST, GATA3, GJA1, GNAS, GSN, IGF1R, KCNMA1, MAP2K6, MAP2K7, MAPK11, MAPK12, MYH10, MYO10, PFN2, PTCH1, PTCH2, SEMA3C, SNAP25, SOS1, STAT5A, STAT5B, STK4, SUFU, TCF7L2, TGM2, TNF, TP53, WASF2* |
| Etonogestrel | Chemical drug | *BDNF, ID2, S100B, TNF* | 3.15 x 10^-11^ | *APC, AQP1, ARFIP2, ATP6V0D1, BCCIP, BMP4, CDC42, CDC42EP2, CPE, CTNNB1, DNM2, DPYSL2, EDNRA, EFNA1, ELF5, ENPP2, FST, GATA3, GJA1, GNAS, GSN, IGF1R, KCNMA1, MAP2K6, MAP2K7, MAPK11, MAPK12, MYH10, MYO10, PFN2, PTCH1, PTCH2, SEMA3C, SNAP25, SOS1, STAT5A, STAT5B, STK4, SUFU, TCF7L2, TGM2, TNF, TP53, WASF2* |
| *ITGA9* | Other | *COL4A1, GATA3, IGF1R, IL5, KIT, KITLG, PDE2A, VAV1* | 3.28 x 10^-11^ | *ADIPOQ, AXIN2, BDNF, BMP4, CAV3, CCL11, COL4A1, CTNNB1, CTSB, FGF2, FZD1, FZD3, GAP43, GATA3, GLI2, HAS2, HHIP, ID2, IGF1R, IL6, MAPK3, PPARG, PTCH1, PTCH2, RAC1, SDC2, TNF, TP53, WNT11* |
| Acetoxy-acetyl-amino-fluorene | Chemical toxicant | *BDNF, CDH3, DCN, EHF, ID2, MAFF, TGM3* | 3.28 x 10^-11^ | *APOD, APP, AQP1, BMP4, CITED2, EFNA1, GATA3, GJA1, GLI2, GNAS, HAS2, IGF1R, IL6, KITLG, MAP2K4, MAP2K6, MEF2C, PLA2G5, PPARG, PTCH1, RAC1, STMN1, TAGLN, TGM2, TNF, WNT7A* |
| *DUSP9* | Phosphatase | *IGF1R, IL6, KIT, PPARG, TNF, TP53* | 3.32 x 10^-11^ | *APP, ARPC2, BDNF, BMP4, CDK5R1, CTNNB1, CTSB, DSE, EFNA1, FGF2, FST, GATA3, GNAS, HAS2, HEY1, IL5, IL6, KITLG, KRT17, MAFF, MAP2K6, MAPK3, MEF2C, PLD1, TAGLN, TDRD7, TGM2, TNF, TP53, WNT7A* |
| Desogestrel | Chemical drug | *AKT3, BMP4, DCN, EHF, EMP2, IL6, KITLG, LMO4, PDE2A, S1PR1, TGM2, TGM3, TNF* | 3.42 x 10^-11^ | *APC, AQP1, ARFIP2, ATP6V0D1, BCCIP, BMP4, CDC42, CDC42EP2, CPE, CTNNB1, DNM2, DPYSL2, EDNRA, EFNA1, ELF5, ENPP2, FST, GATA3, GJA1, GNAS, GSN, IGF1R, KCNMA1, MAP2K6, MAP2K7, MAPK11, MAPK12, MYH10, MYO10, PFN2, PTCH1, PTCH2, SEMA3C, SNAP25, SOS1, STAT5A, STAT5B, STK4, SUFU, TCF7L2, TGM2, TNF, TP53, WASF2* |
| *APPL* | Group | *CDC42, CITED2, KIT, RAC1, SLC9A3R1* | 3.46 x 10^-11^ | *ADM, APP, AXIN2, BMP4, CAV3, COL4A1, CTNNB1, EFNA1, FZD3, GAP43, GATA3, GJA1, GLI2, GNAS, HAS2, HHIP, ID2, IGF1R, IL5, KITLG, MAP2K6, MEF2C, PTCH1, PTCH2, RDH10, SDC2, SNAP25, TAGLN, TGM2, WNT11* |
| Edelfosine | Chemical drug | *APP, IL6, MAP2K6, TNF, TP53* | 3.5 x 10^-11^ | *APP, AXIN2, CCL11, CDK5R1, COL4A1, CTNNB1, CTSB, CTSH, EFNA1, FGF2, FST, GAP43, GATA3, GJA1, GLI2, HAS2, HEY1, IGF1R, IL5, INPP5D, PLD1, RAC1, S1PR1, TAGLN, TGM2* |
| *KLF9* | Transcription regulator | *AIMP2, COL4A1, CTNNB1, EDA, EHF, EMP2, IL5, IL6, KITLG, KRT17, LYVE1, PLPP3, RAC1, SLC9A3R1, ST14, TNF, TP53* | 3.61 x 10^-11^ | *APC, AQP1, ARFIP2, ATP6V0D1, BCCIP, BMP4, CDC42, CDC42EP2, CPE, CTNNB1, DNM2, DPYSL2, EDNRA, EFNA1, ELF5, ENPP2, FST, GATA3, GJA1, GNAS, GSN, IGF1R, KCNMA1, MAP2K6, MAP2K7, MAPK11, MAPK12, MYH10, MYO10, PFN2, PTCH1, PTCH2, SEMA3C, SNAP25, SOS1, STAT5A, STAT5B, STK4, SUFU, TCF7L2, TGM2, TNF, TP53, WASF2* |
| *ZNF217* | Transcription regulator | *APOD, FCER1A, IL6, MAFF, PIK3R3, PTCH1* | 3.67 x 10^-11^ | *ADM, ATL1, AXIN2, BDNF, BMP4, CDK5R1, COL4A1, COL4A3BP, DPP6, EDNRA, FGF2, FZD3, GAP43, GATA3, GJA1, GLI2, HHIP, ID2, IGF1R, MAP2K7, MAPK3, MEF2C, PSEN2, PTCH1, PTCH2, SDC2, TGM2, TNF, TNNC1, TP53, WNT11* |
| *IGA*  *(IGHA, IGHA1)* | Complex | *ADM, APP, BDNF, CCL11, CTNNB1, ID2, IGF1R, IL6, PPARG, TNF, TP53* | 3.92 x 10^-11^ | *AIF1, AXIN2, BDNF, CALB1, CAV3, CCL11, CITED2, CTNNB1, CTSB, DPYSL2, EFNA1, EHF, ENPP2, FST, FZD3, GAP43, GATA3, GNAS, GSK3B, HAS2, HEY1, IGF1R, IL5, KIT, KRT17, MAFF, PLD1, PPARG, PRKCA, PTCH2, S100B, S1PR1, STAT5A, TGM2, TNF, TRAF6* |
| *CDH5* | Other | *CITED2, IL6, TNF, TP53* | 4.03 x 10^-11^ | *ADIPOQ, ADM, AXIN2, BMP4, CAV3, CITED2, COL4A1, CTNNB1, FGF2, FST, FZD3, GAP43, GATA3, GJA1, GSN, HHIP, ID2, IGF1R, IL5, MEF2A, PPARG, PTCH1, PTCH2, RAC1, RAC2, RDH10, S1PR1, SDC2, STAT5B, TNNC1, TRAF6, WNT11, WNT16* |
| Calpain  (*CAPN1-11, CAPNS1*) | Complex | *APP, AQP1, IL6, SNAP25* | 4.04 x 10^-11^ | *ADM, AIF1, APP, CAV3, CCL11, CDK5R1, COL4A1, COL4A3BP, CTNNB1, CTSB, CTSH, DCN, EDNRA, FST, FYN, GATA3, GJA1, GLI2, HEY1, IGF1R, IL6, KITLG, MAP2K7, MAPK11, MEF2C, PIK3CA, PLPP3, PRKACA, PSEN2, PTCH1, RDH10, TAGLN, TNNC1, TP53* |
| ENG | Trans-membrane receptor | *APP, CTSH, FRZB, FZD9, IL6, PPARG, TNF, TP53* | 4.09 x 10^-11^ | *ADIPOQ, ADM, AXIN2, BMP4, COL4A1, CTNNB1, CTSB, EDNRA, EFNA1, FGF2, FST, GATA3, GLI2, HAS2, HEY1, IGF1R, IL5, INPP5D, LYVE1, PPARG, RAC1, RAC2, RDH10, S1PR1, SDC2, STMN1, TAGLN, WNT11* |
| *BDKR*  *(BDKRB1, BDKRB2)* | Group | *IL6, KIT, TNF, TRAF6, WASF2* | 4.28 x 10^-11^ | *ADM, APP, CTNNB1, CTSB, FGF2, GAP43, GNA11, ID2, IL6, IRF6, KCNJ2, MAPK11, MAPK3, PLA2G6, PPARG, PRKCA, RAC1, TAGLN, TNF* |
| *FGF23* | Growth factor | *ADIPOQ, APC, IL6, TNF, TP53* | 4.31 x 10^-11^ | *ADM, APP, BDNF, CTNNB1, CTSB, FGF2, GAP43, GHRL, GJA1, HAS2, ID2, IGF1R, IL5, IL6, IRF6, KCNJ2, MAPK3, PLA2G6, PLD1, PPARG, PRKCA, TNF, TP53* |
| *EID3* | Other | *AXIN2, CTNNB1, GATA3, PPARG, SDC2* | 4.65 x 10^-11^ | *APC, APP, AQP1, ARFIP2, ATP6V0D1, BCCIP, BDNF, BMP4, CDC42, CDC42EP2, CPE, CTNNB1, DNM2, EFNA1, ENPP2, FGF2, FST, GAP43, GATA3, GJA1, GNAS, GSN, HAS2, IGF1R, KCNMA1, MAP2K6, MAP2K7, MAPK11, MAPK12, MAPK3, MYH10, PFN2, PTCH1, PTCH2, RAF1, SOS1, STAT5A, STAT5B, STK4, SUFU, TAGLN, TCF7L2, TGM2, TP53, WASF2* |
| *AVPR1A* | G-protein coupled receptor | *CTSB, EDNRA, IL6, MAPK9, TNF* | 4.68 x 10^-11^ | *ADIPOQ, ADM, APP, BDNF, CAV3, CCL11, CTNNB1, FGF2, FZD1, GAP43, GJA1, HAS2, ID2, IGF1R, IL6, IRF6, KCNJ2, MAPK3, PLA2G6, PPARG, PRKCA, RAC1, TNF, TP53* |
| Rivastigmine | Chemical drug | *ADIPOQ, APP, CCL11, IL5, IL6, TNF* | 4.85 x 10^-11^ | *ADIPOQ, AIF1, APP, BDNF, CCL11, CTNNB1, CTSB, DOCK1, FGF2, FYN, GAP43, IGF1R, IL6, MAPK11, MAPK3, PLA2G5, PPARG, RAC1, RAC2, TP53, VAV1* |
| *SENP2* | Peptidase | *CDC42, FGF2, GSN, RAC1, RAF1, TP53* | 5 x 10^-11^ | *APOD, AQP1, BDNF, BMP4, CALB1, CAV3, CCL11, COL4A1, CTNNB1, EFNA1, EHF, FST, FZD3, GAP43, GATA3, GJA1, GLI2, GNAS, HAS2, HHIP, IGF1R, IL5, KIT, KRT17, PLA2G10, PLD1, PTCH1, PTCH2, RBPJ, SDC2, STAT5A, TFDP1, TGM2, WNT11* |
| *ERK 1/2* dimer | Complex | *AIF1, CTNNB1, FYN, IL6, IRF6, KIT, MAPK12, TNF, TNNC1, TP53* | 5.01 x 10^-11^ | *ADIPOQ, APC, APP, AQP1, ARFIP2, ATP6V0D1, BCCIP, BMP4, CDC42, CDC42EP2, CPE, CTNNB1, DNM2, EFNA1, ENPP2, F11R, FST, GJA1, GNAS, GSN, IGF1R, IL5, IL6, KCNMA1, MAP2K6, MAP2K7, MAPK11, MAPK12, MYH10, PFN2, PLA2G2A, PPARG, PRKCA, PTCH1, PTCH2, SEMA3C, SOS1, STAT5A, STAT5B, STK4, SUFU, TAGLN, TCF7L2, TGM2, TRAF6, WASF2* |
| *EBF1* | Transcription regulator | *APOD, APP, CALB1, GAP43, IL5, MYH10, STAT5A, TMEM100, TNF, WNT7A* | 5.08 x 10^-11^ | *AKT3, GATA3, GSK3B, IL6, INPP5D, MAPK10, PIK3CA, PIK3R1, PIK3R3, PPARG, PRKACA, RAF1, SOS1* |
| *PHB2* | Transcription regulator | *APP, CTSB, CTSH, DCN, ID2, IL6, KRT5, LYVE1, STAT5A, TDRD7, TNF, TP53* | 5.16 x 10^-11^ | *AKT3, APC, AQP1, ARFIP2, ATP6V0D1, BCCIP, BMP4, CDC42, CDC42EP2, CDH3, COL4A1, CPE, CTNNB1, DNM2, EFNA1, ENPP2, F11R, FST, GJA1, GNAS, GSN, IGF1R, KCNMA1, MAP2K6, MAP2K7, MAPK11, MAPK12, MYH10, PFN2, PTCH1, PTCH2, SEMA3C, SLC9A3R1, SOS1, STAT5A, STAT5B, STK4, SUFU, TCF7L2, TGM2, TNF, TP53, WASF2* |
| Desmo-pressin | Biologic drug | *ADIPOQ, ADM, CITED2, CTNNB1, CTSB, IFT27, IGF1R, IL6, PIK3R1, PPARG, PRKCB, SMARCA4, STAT5A, STAT5B, TNF, TP53* | 5.39 x 10^-11^ | *ADM, AQP1, AXIN2, BMP4, COL4A1, CTNNB1, FGF2, FZD3, GAP43, GATA3, GHRL, GLI2, HAS2, HHIP, ID2, IGF1R, IL5, IL6, PLD1, PPARG, PTCH1, PTCH2, SDC2, ST14, TP53, WNT11* |
| Trans-hydroxy-tamoxifen | Chemical drug | *APP, PSEN2, TP53* | 5.57 x 10^-11^ | *APC, AQP1, ARFIP2, ATP6V0D1, BCCIP, BMP4, CDC42, CDC42EP2, CPE, CTNNB1, CTSH, DNM2, EFNA1, ENPP2, FGF2, FST, GJA1, GNAS, GSN, IGF1R, IL6, IRF6, KCNMA1, MAP2K6, MAP2K7, MAPK11, MAPK12, MYH10, PFN2, PTCH1, PTCH2, SEMA3C, SOS1, STAT5A, STAT5B, STK4, SUFU, TCF7L2, TGM2, TNF, TP53, WASF2* |
| *GRIP1* | Transcription regulator | *CCL11, IL6, TNF* | 5.99 x 10^-11^ | *ACADVL, APC, APP, AQP1, ARFIP2, ATP6V0D1, AXIN2, BCCIP, BMP4, CDC42, CDC42EP2, CPE, CTSB, DNM2, ENPP2, FST, GJA1, GNAS, IGF1R, IL6, IRF6, ITIH3, KCNMA1, KRT17, MAP2K6, MAP2K7, MAPK11, MAPK12, MYH10, PFN2, PTCH1, PTCH2, SEMA3C, SMARCA4, SOS1, STAT5A, STAT5B, STK4, SUFU, TGM2, TMEM17, TNF, TP53, UCHL1, WASF2, WNT11* |
| *PELP1* | Other | *COL4A1, IL6, TNF* | 6.25 x 10^-11^ | *AKT3, APC, AQP1, ARFIP2, ATP6V0D1, BCCIP, BMP4, CDC42, CDC42EP2, CDH3, COL4A1, CPE, CTNNB1, DNM2, EFNA1, ENPP2, F11R, FST, GJA1, GNAS, GSN, IGF1R, KCNMA1, MAP2K6, MAP2K7, MAPK11, MAPK12, MYH10, PFN2, PTCH1, PTCH2, SEMA3C, SLC9A3R1, SOS1, STAT5A, STAT5B, STK4, SUFU, TCF7L2, TGM2, TNF, TP53, WASF2* |
| Sitosterol | Chemical - endogenous non-mammalian | *APP, IL6, TNF* | 6.72 x 10^-11^ | *ADIPOQ, APP, BLOC1S6, CCL11, CTNNB1, CTSB, DOCK1, ELF5, FGF2, FYN, GAP43, GNAS, IL6, MAP2K6, PLA2G12A, PPARG, RAC1, RAC2, TNF, TP53, VAV1* |
| *EPM2A* | Phosphatase | *CCL11, IL5, TNF* | 7.52 x 10^-11^ | *AIF1, APP, CTNNB1, FYN, GATA3, GSK3B, IFT27, IGF1R, IL6, ITIH5, KCNQ3, LMO4, MAPK11, MAPK3, NFIB, PPARG, RAC1, RAF1, TCF7L2, TGM2* |
| *SLC2A1* | Transporter | *APP, BDNF, SNAP25* | 7.74 x 10^-11^ | *ADIPOQ, ADM, APP, BDNF, CAV3, CDK5R1, CTNNB1, CTSB, DSE, EFNA1, GATA3, GLI2, GNAS, HAS2, IFT27, IGF1R, IL5, IL6, KITLG, KRT17, MAP2K6, MAPK3, MEF2C, PLD1, PPARG, RAC1, RDH10, TAGLN, TCF7L2, TDRD7, TGM2* |
| *PPARGC1B* | Transcription regulator | *GJA1, IL6, TNF* | 8.09 x 10^-11^ | *ACADVL, APC, APP, AQP1, ARFIP2, ATP6V0D1, AXIN2, BCCIP, BMP4, CDC42, CDC42EP2, CPE, CTSB, DNM2, ENPP2, FST, GJA1, GNAS, IGF1R, IL6, IRF6, ITIH3, KCNMA1, KRT17, MAP2K6, MAP2K7, MAPK11, MAPK12, MEF2C, MYH10, PFN2, PTCH1, PTCH2, SEMA3C, SMARCA4, SOS1, STAT5A, STAT5B, STK4, SUFU, TGM2, TMEM17, TNF, TP53, UCHL1, WASF2, WNT11* |
| *APBB1* | Transcription regulator | *ADIPOQ, AIF1, BDNF, ID2, IL6, PIK3R1, TP53* | 8.35 x 10^-11^ | *APC, APP, AQP1, ARFIP2, ATP6V0D1, BCCIP, BMP4, CDC42, CDC42EP2, CPE, CTNNB1, DNM2, EFNA1, ENPP2, FST, GJA1, GNAS, GSN, IGF1R, IL6, KCNMA1, MAP2K6, MAP2K7, MAPK11, MAPK12, MYH10, PFN2, PTCH1, PTCH2, SEMA3C, SOS1, STAT5A, STAT5B, STK4, SUFU, TAGLN, TCF7L2, TGM2, TP53, WASF2* |
| *TIMP1* | Cytokine | *ADIPOQ, BDNF, CDH3, CTNNB1, DCN, FZD1, GNAS, GSK3B, HAS2, IL5, IL6, PPARG, RAC2, TNF, TP53* | 8.36 x 10^-11^ | *ADIPOQ, ADM, CAPRIN1, CAV3, CCL11, CITED2, COL4A1, CTNNB1, CTSB, DVL2, EFNA1, GATA3, GJA1, GLI2, GNAS, HAS2, ID2, IL5, IL6, KITLG, MAP2K4, MAP2K6, MEF2C, PLA2G5, RAC1, RDH10, ST14, STMN1, TAGLN, TGM2, TP53* |
| Fluoxy-mesterone | Chemical drug | *TNF, TP53* | 8.59 x 10^-11^ | *APC, AQP1, ARFIP2, ATP6V0D1, BCCIP, BDNF, BMP4, CDC42, CDC42EP2, CPE, CTNNB1, DNM2, EFNA1, ENPP2, FGF2, FST, GJA1, GNAS, GSN, IGF1R, KCNMA1, MAP2K6, MAP2K7, MAPK11, MAPK12, MYH10, PFN2, PTCH1, PTCH2, SEMA3C, SOS1, STAT5A, STAT5B, STK4, SUFU, TCF7L2, TGM2, TNF, TP53, WASF2* |
| Swinholide A | Chemical toxicant | *APP, TNF* | 9.05E-11 | *ADIPOQ, BDNF, CAV3, CCL11, CTSB, EFNA1, FGF2, FZD1, GJA1, GNAS, HAS2, IGF1R, IL5, IL6, KITLG, MAP2K6, MAPK3, MEF2C, RAC1, TAGLN, TGM2, TNF, TP53* |
| *HLA-DR*  *(HLA-DRA,*  *HLA-DRB1,*  *HLA-DRB3,*  *HLA-DRB4,*  *HLA-DRB5)* | Complex | *FOXP1, TP53* | 1.01 x 10^-10^ | *ADIPOQ, BDNF, CAV3, CCL11, CDK5R1, CITED2, CTSB, DSE, EFNA1, FOXP1, FST, FYN, FZD1, GJA1, GNAS, HAS2, IGF1R, IL5, KITLG, KRT17, MAP2K6, MEF2C, PIK3CA, PLD1, RAC1, STAT5A, STAT5B, TAGLN, TDRD7, TGM2, TP53* |
| *FRAT1* | Other | *IL6, TNF* | 1.12 x 10^-10^ | *AIF1, APOD, APP, BMP4, COL4A1, CTNNB1, CTSB, ENPP2, FGF2, FYN, FZD3, GAP43, GATA3, GJA1, GLI2, GSN, ID2, IFT27, IGF1R, IL6, MAPK11, MAPK3, PHACTR4, PTCH1, PTCH2, PTPDC1, RAC1, SDC2, TCF7L2, TGM2, TP53, WNT11* |
| Toxaphene | Chemical toxicant | *IL6, TNF* | 1.13 x 10^-10^ | *ACADVL, APC, AQP1, ARFIP2, ATP6V0D1, BCCIP, BDNF, BMP4, CDC42, CDC42EP2, CPE, DNM2, EFNA1, ENPP2, FST, GJA1, GNAS, GSK3B, GSN, IGF1R, IL6, KCNMA1, KRT17, MAP2K6, MAP2K7, MAPK11, MAPK12, MYH10, PFN2, PPARG, PTCH1, PTCH2, SEMA3C, SOS1, STAT5A, STAT5B, STK4, SUFU, TCF7L2, TGM2, TNF, TP53, WASF2* |
| *MPO* | Enzyme | *IL6, TNF* | 1.13 x 10^-10^ | *ADIPOQ, BDNF, CCL11, CTNNB1, CTSB, DOCK1, FGF2, FYN, GAP43, IL6, PLA2G5, PPARG, RAC1, RAC2, TNF, VAV1* |
| Gamma-linolenic acid | Chemical - endogenous mammalian | *IL6, TNF* | 1.13 x 10^-10^ | *ADIPOQ, ADM, APP, AQP1, CTNNB1, CTSB, EDNRA, FGF2, GAP43, HAS2, ID2, IRF6, KCNJ2, MAPK3, MAPK9, PLA2G6, PRKCA, RAC1, TGM2, TP53* |
| *LAMC1* | Other | *IL6, TNF* | 1.16 x 10^-10^ | *ADIPOQ, ADM, APP, BDNF, CAV3, CCL11, CTNNB1, FGF2, FZD1, GAP43, GJA1, HAS2, ID2, IGF1R, IL6, IRF6, KCNJ2, MAPK3, PLA2G6, PPARG, PRKCA, RAC1, TNF, TP53* |
| *HSP90B1* | Other | *ADIPOQ, CPE, ID2, MAFF, MAPK11, PRKCA, STMN1, TP53* | 1.22 x 10^-10^ | *APC, APP, AQP1, ARFIP2, ATP6V0D1, BCCIP, BMP4, CDC42, CDC42EP2, COL4A3BP, CPE, CTNNB1, DNM2, ENPP2, FST, GATA3, GJA1, GNAS, GSN, IGF1R, IL5, IL6, KCNMA1, MAP2K6, MAP2K7, MAPK11, MAPK12, MYH10, PFN2, PPARG, PTCH1, PTCH2, SEMA3C, SOS1, STAT5A, STAT5B, STK4, SUFU, TCF7L2, TGM2, TNF, TP53, TRAF6, WASF2* |
| *FAM129B* | Other | *BMP4, GATA3, IL5, IL6, KRT71, TNF* | 1.33 x 10^-10^ | *ADM, ARF1, AXIN2, COL4A1, CTNNB1, CTSB, FGF2, FZD1, FZD3, GAP43, GATA3, GJA1, GLI2, HAS2, HEY1, HHIP, ID2, IGF1R, IL5, MAPK12, MYH10, PPARG, PTCH1, PTCH2, SDC2, TAGLN, TP53, WNT11* |
| *TFIIH*  *(CCNH,*  *CDK7,*  *ERCC2,*  *ERCC3,*  *GTF2H1,*  *GTF2H2,*  *GTF2H3,*  *GTF2H4,*  *GTF2H5,*  *MNAT1)* | Complex | *IL5, IL6, MAPK11, PIK3R1, STK4, TNF* | 1.34 x 10^-10^ | *ADIPOQ, APC, APP, AQP1, ARFIP2, ATP6V0D1, BCCIP, BMP4, CDC42, CDC42EP2, CPE, CTNNB1, DNM2, EFNA1, ENPP2, F11R, FST, GJA1, GNAS, GSN, HAND1, IGF1R, KCNMA1, KITLG, MAP2K6, MAP2K7, MAPK11, MAPK12, MYH10, PFN2, PLA2G2A, PPARG, PTCH1, PTCH2, SEMA3C, SOS1, STAT5A, STAT5B, STK4, SUFU, TAGLN, TCF7L2, TGM2, TRAF6, WASF2* |
| *ZNF335* | Other | *BDNF, CTSB, DPYSL2, KIT, PRKACA, TNF* | 1.37 x 10^-10^ | *APC, APP, AQP1, ARFIP2, ATP6V0D1, BCCIP, BDNF, BMP4, CDC42, CDC42EP2, CPE, CTNNB1, DNM2, E2F8, EFNA1, ENPP2, FST, GATA3, GJA1, GNAS, GSN, IGF1R, IL5, IRF6, KCNMA1, MAP2K6, MAP2K7, MAPK11, MAPK12, MAPK3, MEF2C, MYH10, PFN2, PTCH1, PTCH2, RAF1, SOS1, STAT5A, STAT5B, STK4, SUFU, TCF7L2, TFAP2A, TGM2, WASF2* |
| Dishevelled  (*DVL1-3*) | Group | *CTNNB1, KIT, MAPK3, PPARG, PRKCA, TCF7L2* | 1.69 x 10^-10^ | *AIF1, APOD, APP, AQP1, COL4A1, CTNNB1, FGF2, FYN, FZD3, GAP43, GATA3, GJA1, GLI2, GSN, HHIP, IFT27, IGF1R, IL5, IL6, MAPK11, MAPK3, PLA2G10, PTCH2, RAC1, TCF7L2, TP53, WNT11* |
| N-(6,6-dimethyl-5-((1-methylpiperidin-4-yl)carbonyl)-1,4,5,6-tetrahydropyrrolo(3,4-c)pyrazol-3-yl)-3-methylbutanamide | Chemical drug | *CCL11, GATA3, IL5, IL6, TNF* | 1.75 x 10^-10^ | *AIF1, AXIN2, BDNF, CITED2, CTNNB1, CTSB, DPYSL2, ENPP2, FGF2, FYN, FZD3, GSK3B, HEY1, IL6, MAFF, MAPK11, MAPK3, PRKCA, PTCH2, RBPJ, S100B, S1PR1, TNF, TP53, TRAF6* |
| Imidazole | Chemical - endogenous mammalian | *BMP4, GLI2, HHIP, PTCH1, WNT2B* | 1.75 x 10^-10^ | *ACADVL, AXIN2, BDNF, BMP4, COL4A1, EFNA1, FZD3, GAP43, GATA3, GJA1, GLI2, GNAS, GSK3B, HAS2, HHIP, ID2, IL5, KITLG, MAP2K6, MEF2C, PTCH1, PTCH2, SDC2, TAGLN, TGM2, WNT11* |
| *SIAH1* | Enzyme | *CTNNB1, IGF1R, PPARG, RAC1, TNF* | 1.84 x 10^-10^ | *ADIPOQ, APP, AXIN2, BMP4, CCL11, COL4A1, CTNNB1, ENPP6, FZD3, GAP43, GJA1, HAS2, HEY1, HHIP, ID2, IGF1R, IL5, IL6, KIT, KITLG, PDE2A, PLA2G10, PLA2G2A, PPARG, PRKCB, PTCH1, PTCH2, SDC2, TGM2, TNF, WNT11* |
| Racemic flurbiprofen | Chemical drug | *APP, CTSB, IL6, SUFU, TNF* | 1.86 x 10^-10^ | *APP, AQP1, ARPC2, AXIN2, BMP4, CDK5R1, COL4A1, EDNRA, FST, FZD3, GAP43, GATA3, GHRL, GJA1, GLI2, HAS2, HEY1, HHIP, ID2, MAFF, MAPK9, PLD1, PTCH1, PTCH2, RAC1, SDC2, TP53, WNT11* |
| Nisoldipine | Chemical drug | *BDNF, FGF2, IL6, TNF* | 1.88 x 10^-10^ | *ADM, APP, CTNNB1, CTSB, FGF2, GAP43, ID2, IL5, IL6, IRF6, KCNJ2, KIT, MAPK3, PLA2G6, PPARG, PRKCA, TNF* |
| Diflunisal | Chemical drug | *CCL11, IL6, TNF, TP53* | 1.93 x 10^-10^ | *APP, AQP1, AXIN2, BMP4, COL4A1, EDNRA, FGF2, FZD3, GAP43, GATA3, GJA1, GLI2, HHIP, ID2, IL6, MAPK9, PTCH1, PTCH2, RAC1, SDC2, TGM2, TNF, TP53, WNT11* |
| *PITX2* | Transcription regulator | *AGPS, APP, IL5, IL6, IRF6, PIK3CA, RDH10, STAT5B, TNF, TTC30B, TTLL1* | 1.94 x 10^-10^ | *APP, AXIN2, BMP4, COL4A1, FZD3, FZD9, GAP43, GATA3, GJA1, GLI2, HHIP, ID2, IL6, IRF6, PPARG, PTCH1, PTCH2, SDC2, TBX6, TNF, TP53, WNT11, WNT2* |
| *NCX*  *(SLC8A1-A3)* | Group | *FCER1A, IL6, PRKCA, PRKCB, TNF* | 2.04 x 10^-10^ | *ADIPOQ, ADM, APP, BDNF, CAV3, CCL11, CTNNB1, FGF2, FZD1, GAP43, GJA1, HAS2, ID2, IGF1R, IL6, IRF6, KCNJ2, MAPK3, PLA2G6, PPARG, PRKCA, RAC1, TNF* |
| *ID3* | Transcription regulator | *CALB1, CPE, CTNNB1, FZD10, FZD6, GSN, MYO10, PIK3R1, PLCG1, PLPP3, ST14, TFDP1, TP53, WNT11* | 2.29 x 10^-10^ | *ADIPOQ, ADM, CAV3, COL4A1, CTNNB1, ENPP2, FYN, GATA3, GJA1, GLI2, ID2, IGF1R, IL5, IL6, IRF6, KIF4A, KIT, LMO4, MEF2A, MEF2C, PIK3R1, PPARG, PRKACA, RDH10, TNF, TNNC1, TP53* |
| *CRHR2* | G-protein coupled receptor | *APP, BDNF, CTNNB1, FGF2, HAS2, IL5, IL6, PPARG, PRKCA, TNF, TRAF6* | 2.3 x 10^-10^ | *AIF1, APP, BDNF, BMP4, CALB1, CAV3, CTNNB1, EFNA1, EHF, FST, FYN, GJA1, GNAS, HAS2, IFT27, IGF1R, IL5, IL6, KCNMB1, KIT, KRT17, MAPK11, MAPK3, PLD1, PPARG, RAC1, RBPJ, STAT5A, TCF7L2* |
| CACN | Group | *APP, IGF1R, PLD1, PLPP3, S100B, TNF* | 2.49 x 10^-10^ | *ADIPOQ, ADM, APP, AQP1, ARPC2, BMP4, CDK5R1, CTNNB1, CTSB, FGF2, FST, GJA1, GNAS, HEY1, ID2, IL5, IL6, KCNJ2, MAFF, PLA2G6, PLD1, PPARG, PRKCA, PTCH1, RAC1, TGM2, TP53* |
| *DGAT1* | Enzyme | *ADIPOQ, CCL11, ENPP2, GJA1, IL5, IL6, INPP5D, KIT, TNF* | 2.63 x 10^-10^ | *ADIPOQ, ADM, APOD, APP, AQP1, BMP4, CLTC, CTNNB1, CTSB, DVL2, FGF2, FST, GAP43, GATA3, ID2, IL5, IL6, IRF6, KCNJ2, MAPK3, PLA2G6, PPARG, PRKCA, PTCH1, S1PR1, TNF* |
| Cyclopamine | Chemical reagent | *AQP1, EDNRA, IL6, MAPK9, PPARG, RAC1, TGM2, TNF, TP53* | 2.71 x 10^-10^ | *ACADVL, ADIPOQ, ADM, BDNF, CAV3, GATA5, GLI2, HHIP, ID2, IGF1R, IL5, IL6, ITIH5, KIT, LMO4, MAPK11, MEF2C, NFIB, PPARG, PTCH1, PTCH2, RDH10, TGM2, TMEM100, TNF, TP53, WNT2B* |
| Mephenytoin | Chemical drug | *ADM, CITED2, CLTC, EFNA1, FST, GAP43, GATA3, IL6, PPARG, S1PR1, TNF, TP53* | 2.95 x 10^-10^ | *ADIPOQ, APOD, APP, AQP1, BDNF, BMP4, CAV3, CCL11, CDK5R1, CTSB, EDNRA, FZD1, GNA11, HAS2, IGF1R, IL5, MAP2K4, MAP2K6, MAP2K7, MAPK3, MAPK9, PLCG1, PRKACA, PRKCA, PTCH1, RAC1, TMEM100* |
| *DIO3* | Enzyme | *IL6, ITIH5, LMO4, NFIB, TGM2, TNF, TP53* | 3.08 x 10^-10^ | *ADIPOQ, ADM, APP, COL4A1, CPE, CTNNB1, CTSH, EFNA1, FZD3, FZD6, GHRL, ID2, IGF1R, IL6, ITIH3, KRT17, NFIB, PLA2G2A, PLA2G5, PPARG, SEMA3C, ST14, TFDP1, TNF, TP53* |
| Olmesartan | Chemical drug | *ADIPOQ, AXIN2, ID2, IL5, IL6, KIT, PPARG, STAT5A, STAT5B, TNF, TNNC1, TP53* | 3.3 x 10^-10^ | *ADIPOQ, APOD, AQP1, BDNF, BMP4, CDK5R1, CTNNB1, CTSB, DSE, DVL2, EFNA1, GATA3, GNAS, HAS2, ID2, IL6, KITLG, KRT17, MAP2K6, MAPK3, MEF2C, PLD1, PPARG, PTCH1, TAGLN, TDRD7, TGM2, TNF* |
| *LRP4* | Other | *BMP4, CTNNB1, MEF2C, PTCH1* | 3.34 x 10^-10^ | *ADM, APP, BDNF, BMP4, CAV3, CDK5R1, COL4A1, CTNNB1, CTSB, DCN, DSE, E2F8, GAP43, GATA3, GJA1, GLI2, ID2, IGF1R, INPP5D, KIT, KRT17, MAP2K4, MEF2C, MYH10, PLA2G12A, PLA2G5, PLD1, PPARG, STMN1, TDRD7, TGM2, WNT2* |
| Protein kinase C inhibitor | Chemical drug | *FGF2, IL6, PPARG, TNF* | 3.36 x 10^-10^ | *ADM, APP, BDNF, CTNNB1, CTSB, FGF2, GAP43, ID2, IL6, IRF6, KCNJ2, MAPK3, PLA2G6, PPARG, PRKCA, TNF* |
| Palmitoleic acid | Chemical - endogenous mammalian | *DCN, PPARG, RAC1, TNF* | 3.43 x 10^-10^ | *ADIPOQ, APOD, APP, AQP1, BDNF, BMP4, CAV3, CCL11, CITED2, CTNNB1, CTSB, FGF2, FZD1, GJA1, HAS2, IGF1R, IL5, IL6, MAPK3, PLD1, PPARG, PTCH1, RAC1, STAT5B, TNF, TNNC1, TRAF6, WNT16* |
| *ZNF703* | Other | *GATA3, IL6, RAC1, RAC2, TNF* | 3.43 x 10^-10^ | *APP, AXIN2, BDNF, BMP4, CTNNB1, DCN, FZD1, GAP43, GSK3B, HAS2, IL6, KIT, KITLG, MEF2C, PLD1, PPARG, SDC2, TNF, WNT11* |
| *GNB5* | Enzyme | *ADIPOQ, APP, IL6, PPARG, TNF* | 3.54 x 10^-10^ | *ADIPOQ, ADM, AIF1, APP, ARPC2, BMP4, CAV3, CDK5R1, CTSB, DSE, EFNA1, FYN, GLI2, GNAS, GSK3B, GSN, IRF6, KCNJ2, KCNMA1, KITLG, KRT17, MAFF, MAP2K6, MAPK11, MAPK3, MEF2C, PLA2G6, PPARG, PRKCA, RAC1, RDH10, TDRD7, TGM2* |
| *CSNK1G2* | Kinase | *APC, CTSB, FST, FYN, GATA3, TFDP1* | 3.55 x 10^-10^ | *APC, AQP1, ARFIP2, ATP6V0D1, BCCIP, BMP4, CDC42, CDC42EP2, CPE, DNM2, EFNA1, ENPP2, FST, GJA1, GNAS, GSK3B, GSN, IGF1R, KCNMA1, MAP2K6, MAP2K7, MAPK11, MAPK12, MYH10, PFN2, PIK3R1, PTCH1, PTCH2, SEMA3C, SOS1, STAT5A, STAT5B, STK4, SUFU, TCF7L2, TGM2, TNF, TP53, WASF2* |
| *ESRRB* | Ligand-dependent nuclear receptor | *ACADVL, ANXA7, APP, IL6, PPARG, TNF* | 3.86 x 10^-10^ | *APC, AQP1, ARFIP2, ATP6V0D1, BCCIP, BMP4, CDC42, CDC42EP2, CPE, CTNNB1, DNM2, EFNA1, ENPP2, FST, GJA1, GNAS, GSN, IGF1R, KCNMA1, MAP2K6, MAP2K7, MAPK11, MAPK12, MEF2C, MYH10, PFN2, PTCH1, PTCH2, SEMA3C, SOS1, STAT5A, STAT5B, STK4, SUFU, TCF7L2, TGM2, TNF, TP53, WASF2* |
| MIR-199a-5p (and other miRNAs w/seed CCAGUGU) | Mature microRNA | *COL4A1, FGF2, GJA1, HEY1, PIK3R3, PPARG, TP53* | 3.88 x 10^-10^ | *CITED2, COL4A1, DCN, ENPP2, GATA3, IL5, KRT17, LIN7C, LMO4, PLA2G2A, PLD1, PPARG, STAT5A, TAGLN, TGFB1I1, TNF, WNT7A* |
| *Kt 5926* | Chemical - kinase inhibitor | *CTNNB1, CTSB, IGF1R, IL5, IL6, TNF, TP53* | 4 x 10^-10^ | *ADM, APP, BDNF, CTNNB1, CTSB, FGF2, GAP43, ID2, IL6, IRF6, KCNJ2, MAPK3, PLA2G6, PPARG, PRKCA, TNF* |
| *ICAM3* | Trans-membrane receptor | *ADIPOQ, IL6, TNF* | 4.2 x 10^-10^ | *ADIPOQ, ADM, APP, CAV3, CTNNB1, CTSB, FGF2, GAP43, GLI2, ID2, IGF1R, IL5, IL6, IRF6, KCNJ2, MAPK3, PLA2G6, PPARG, PRKCA, RDH10, TNF, TP53* |
| *LY117018* | Chemical drug | *BMP4, GLI2, MEF2C* | 4.31 x 10^-10^ | *ADIPOQ, APC, AQP1, ARFIP2, ATP6V0D1, BCCIP, BMP4, CDC42, CDC42EP2, CPE, CTNNB1, DNM2, EFNA1, ENPP2, FST, GJA1, GNAS, GSN, IGF1R, IL6, KCNMA1, MAP2K6, MAP2K7, MAPK11, MAPK12, MYH10, PFN2, PTCH1, PTCH2, SEMA3C, SOS1, STAT5A, STAT5B, STK4, SUFU, TCF7L2, TGM2, TP53, WASF2* |
| *MALT1* | Peptidase | *APP, CCL11, MAPK3* | 4.31 x 10^-10^ | *AGPS, APP, AXIN2, BCCIP, BDNF, CALB1, CAV3, CDH3, CSK, CTNNB1, EFNA1, EHF, FST, GAP43, GNAS, HAS2, IL6, IRF6, KIT, KRT17, PIK3CA, PIK3R1, PLD1, RBPJ, RDH10, STAT5A, TFDP1, TGFB1I1, TGM2, TNF, TRAF6, TTC30B, TTLL1* |
| *RGN* | Enzyme | *APP, BDNF, LYVE1* | 4.54 x 10^-10^ | *ADM, APP, BDNF, CTNNB1, CTSB, FGF2, GAP43, ID2, IL5, IL6, IRF6, KCNJ2, MAPK3, PLA2G6, PPARG, PRKCA, TNF* |
| Jwh-015 | Chemical reagent | *CCL11, IL5, IL6* | 4.78 x 10^-10^ | *ADIPOQ, ADM, APP, BDNF, BMP4, CAV3, CCL11, CDK5R1, CTNNB1, CTSB, DSE, GAP43, GATA3, GJA1, GLI2, ID2, IGF1R, IL5, IL6, KRT17, MAPK3, PLD1, PPARG, RDH10, SDC2, TDRD7, TGM2, TNNC1, TP53* |
| AKT inhibitor VII | Chemical reagent | *CCL11, IL5, TNF* | 4.93 x 10^-10^ | *APP, AXIN2, BMP4, COL4A1, CTNNB1, FZD3, GAP43, GATA3, GJA1, GLI2, HHIP, ID2, IFT27, IGF1R, PPARG, PTCH1, PTCH2, RAC1, SDC2, TCF7L2, TNF, WNT11* |
| *LYPD6* | Other | *APP, GATA3, IL6* | 5.19 x 10^-10^ | *APP, AXIN2, BMP4, COL4A1, CTNNB1, FZD3, GAP43, GATA3, GJA1, GLI2, HHIP, ID2, IFT27, IGF1R, PPARG, PTCH1, PTCH2, RAC1, SDC2, TCF7L2, TNF, WNT11* |
| Delavirdine | Chemical drug | *APP, FGF2, TP53* | 5.28 x 10^-10^ | *ADM, APOD, APP, AQP1, BDNF, BMP4, CDK5R1, EDNRA, FLOT1, GAP43, GJA1, GNA11, HAS2, IL5, MAFF, MAP2K4, MAP2K6, MAP2K7, MAPK9, PLCG1, PRKACA, PRKCA, PTCH1, SCN1B, SLC9A3R1, TAGLN, TMEM100* |
| *PDIA3* | Peptidase | *APP, CDC42, IL6, RAC1, TNF* | 5.48 x 10^-10^ | *ADM, APP, CDC42, CTNNB1, CTSB, FGF2, GAP43, GJA1, HAS2, ID2, IL5, IL6, IRF6, KCNJ2, MAPK3, PLA2G6, PLPP3, PPARG, PRKCA, TGM2, TNF, TP53* |
| Ba^2+^ | Chemical reagent | *ADIPOQ, BDNF, ID2, IL5, MYH10* | 5.51 x 10^-10^ | *ADM, APOD, APP, AQP1, BMP4, CTNNB1, CTSB, FGF2, GAP43, ID2, IL5, IRF6, KCNJ2, MAPK3, MAPK9, MEF2C, PLA2G6, PPARG, PRKCA, PTCH1* |
| *FSCN1* | Other | *BDNF, CAPRIN1, FGF2, IGF1R, IL6, KITLG, MAP2K4, MAPK3, PAFAH1B2, RAF1* | 5.51 x 10^-10^ | *ADM, APP, ARPC2, CDK5R1, CTNNB1, CTSB, FGF2, FST, GAP43, HEY1, ID2, IL6, IRF6, KCNJ2, MAFF, MAPK3, PLA2G6, PPARG, PRKCA, TGM2, TNF* |
| *HPRT1* | Enzyme | *DPYSL2, HAND1, ID2, IL6* | 5.73 x 10^-10^ | *AXIN2, BDNF, BMP4, CDK5R1, COL4A1, CTNNB1, CTSB, DSE, FZD3, GAP43, GATA3, GJA1, GLI2, HHIP, ID2, IL5, IL6, KRT17, MAPK3, PLD1, PPARG, PTCH1, PTCH2, SDC2, TDRD7, TNF, TP53, WNT11* |
| Sew 2871 | Chemical reagent | *ADIPOQ, BDNF, IL6, TNF* | 5.99 x 10^-10^ | *ADIPOQ, ARPC2, BDNF, CAV3, CCL11, CDK5R1, CTNNB1, CTSB, FGF2, FST, FZD1, GATA3, GJA1, HAS2, HEY1, IGF1R, IL6, MAFF, MAPK3, RAC1, S1PR1, TGM2, TNF* |
| *BMP15* | Growth factor | *BDNF, FGF2, TAGLN, TP53* | 6.46 x 10^-10^ | *ADIPOQ, BMP4, COL4A1, GJA1, HAS2, HEY1, ID2, IGF1R, IL6, INPP5D, KIT, KITLG, MAPK3, PIK3R1, PPARG, RAC1, S1PR1, TGM2* |
| Camostat | Chemical drug | *IL6, KCNQ3, RAC1, TNF* | 6.59 x 10^-10^ | *ADIPOQ, BDNF, CCL11, CTNNB1, DCN, DOCK1, FGF2, FST, FYN, GATA3, HEY1, IL5, IL6, PLA2G5, PLPP3, RAC1, RAC2, TAGLN, VAV1* |
| Pyri-methamine | Chemical drug | *AKT3, ID2, IL5, IL6, MAPK9, TNF, TRAF6* | 6.73 x 10^-10^ | *ADM, APOD, APP, AQP1, BMP4, CDK5R1, EDNRA, FGF2, FLOT1, GAP43, GJA1, GNA11, HAS2, IL5, MAFF, MAP2K4, MAP2K6, MAP2K7, MAPK9, PLCG1, PRKACA, PRKCA, PTCH1, SCN1B, SLC9A3R1, TAGLN, TMEM100* |
| Estradiol valerate | Chemical drug | *APP, COL4A1, CTNNB1, IL6, TNF, TP53* | 7.12 x 10^-10^ | *AKT3, APC, APP, AQP1, ARFIP2, ATP6V0D1, BCCIP, BDNF, BMP4, CDC42, CDC42EP2, CDH3, COL4A1, CPE, DNM2, EFNA1, ENPP2, F11R, FST, GNAS, GSN, IGF1R, KCNMA1, KRT17, MAP2K6, MAP2K7, MAPK11, MAPK12, MYH10, PFN2, PTCH1, PTCH2, SEMA3C, SLC9A3R1, SOS1, STAT5A, STAT5B, STK4, SUFU, TCF7L2, TGM2, TNF, TP53, WASF2* |
| *ESR1* | Ligand-dependent nuclear receptor | *ADIPOQ, APC, AXIN2, COL4A1, CTNNB1, DCN, IL5, IL6, PPARG, TNF, TP53, UPK1A, UPK1B, UPK2* | 7.19 x 10^-10^ | *APC, AQP1, ARFIP2, ATP6V0D1, BCCIP, BMP4, CDC42, CDC42EP2, CPE, CTNNB1, DNM2, EFNA1, ENPP2, FST, GJA1, GNAS, GSN, IGF1R, KCNMA1, MAP2K6, MAP2K7, MAPK11, MAPK12, MYH10, PFN2, PTCH1, PTCH2, SEMA3C, SOS1, STAT5A, STAT5B, STK4, SUFU, TCF7L2, TGM2, TNF, TP53, WASF2* |
| Ketorolac | Chemical drug | *DSE, HEY1, MAPK12, SNAP25, TNF* | 7.43 x 10^-10^ | *AQP1, AXIN2, BMP4, COL4A1, EDNRA, FGF2, FZD3, GAP43, GATA3, GJA1, GLI2, HHIP, ID2, IL5, IL6, MAPK9, PTCH1, PTCH2, RAC1, SDC2, TGM2, TNF, TP53, WNT11* |
| *TERT* | Enzyme | *ADIPOQ, COL4A1, GAP43, ID2, IL6, PPARG, SNAP25, TCF7L2, TNF* | 8.1 x 10^-10^ | *AXIN2, BDNF, BMP4, CDK5R1, COL4A1, COL4A3BP, EDNRA, FGF2, FZD3, GAP43, GATA3, GJA1, GLI2, HHIP, ID2, IGF1R, MAP2K7, MAPK3, MEF2C, PSEN2, PTCH1, PTCH2, SDC2, TGM2, TNF, TNNC1, TP53, WNT11* |
| Pyrilamine | Chemical drug | *APC, GAP43, PAFAH1B1, SNAP25, TAGLN, TFDP1, TNF* | 8.42 x 10^-10^ | *AXIN2, BDNF, BMP4, CDK5R1, COL4A1, CTNNB1, CTSB, DSE, FZD3, GAP43, GATA3, GJA1, GLI2, HHIP, ID2, IL5, IL6, KRT17, MAPK3, PLD1, PPARG, PTCH1, PTCH2, SDC2, TDRD7, TNF, TP53, WNT11* |
| *WNT3A* | Cytokine | *DOCK1, IL5, IL6, INPP5D, S1PR1, TNF, TP53* | 9.09 x 10^-10^ | *APP, AXIN2, BDNF, BMP4, CTNNB1, DCN, FZD1, GAP43, GSK3B, HAS2, IL6, KIT, KITLG, MEF2C, PLD1, PPARG, TNF, WNT11* |
| *NPR3* | G-protein coupled receptor | *ADM, FGF2, GAP43, GNAS, IL6, PPARG, STAT5A, TP53* | 9.19 x 10^-10^ | *ADM, APP, BDNF, CTNNB1, CTSB, FGF2, GAP43, GJA1, ID2, IRF6, KCNJ2, MAPK3, PLA2G6, PPARG, PRKCA, TP53* |
| Catechol | Chemical - endogenous mammalian | *CTNNB1, GAP43, IL6, MAPK3, PLD1, PRKCA, TNF* | 9.19 x 10^-10^ | *ADM, APP, CTNNB1, CTSB, FGF2, GAP43, ID2, IL5, IL6, IRF6, KCNJ2, MAPK3, PLA2G6, PPARG, PRKCA, TNF* |
| *GATA6* | Transcription regulator | *APP, IL6, S100B, TNF* | 9.19 x 10^-10^ | *ATP6V0D1, COL4A1, EFNA1, FGF2, GNAS, HAS2, IL5, INPP5D, IRF6, KITLG, KRT17, LYVE1, MAP2K6, MEF2C, RAC1, S1PR1, SEMA3C, TAGLN, TGM2, TNNC1, TP53, WNT2* |
| *MIR-133* | MicroRNA | *ACADVL, IL6, PPARG, TNF* | 9.35 x 10^-10^ | *ADIPOQ, BDNF, CAV3, CCL11, CDC42, CTSB, FCER1A, FGF2, FZD1, GJA1, HAS2, IGF1R, IL6, INPP5D, MAPK3, PIK3R1, PLCG1, PLCG2, PPARG, RAF1, S1PR1, TAGLN, TNF, TP53* |
| *IgG2b*  *(IGG2)* | Complex | *GATA3, HEY1, IL5, LYVE1* | 9.44 x 10^-10^ | *ADIPOQ, APP, BDNF, DOCK1, FYN, IL6, PLA2G5, RAC1, RAC2, TNF, VAV1* |
| *CD79B* | Trans-membrane receptor | *BDNF, GSK3B, IL6, TNF* | 1 x 10^-9^ | *BDNF, BMP4, CDK5R1, CITED2, CTNNB1, CTSB, DSE, FOXP1, FST, GJA1, GLI2, IGF1R, IL5, IL6, KRT17, MAP2K4, MAPK3, MEF2C, PIK3CA, PLA2G5, PLCG1, PLD1, STMN1, TDRD7, TGM2, TNF, TP53* |
| Nabumetone | Chemical drug | *IL5, IL6, TNF, TP53* | 1.02 x 10^-9^ | *AQP1, AXIN2, BMP4, COL4A1, EDNRA, FGF2, FZD3, GAP43, GATA3, GJA1, GLI2, HHIP, ID2, IL6, MAPK9, PTCH1, PTCH2, RAC1, SDC2, TGM2, TNF, TP53, WNT11* |
| Tolmetin | Chemical drug | *APC, APP, FZD1, FZD6, TP53* | 1.02 x 10^-9^ | *AQP1, AXIN2, BMP4, COL4A1, EDNRA, FGF2, FZD3, GAP43, GATA3, GJA1, GLI2, HHIP, ID2, IL6, MAPK9, PTCH1, PTCH2, RAC1, SDC2, TGM2, TNF, TP53, WNT11* |
| NADP | Chemical - endogenous mammalian | *ACADVL, BDNF, CCL11, CTSB, CTSH, ENPP2, FLOT1, GATA3, GSN, IL6, KITLG, PPARG, TGM2, TNF* | 1.11 x 10^-9^ | *ADM, APP, BDNF, CTNNB1, CTSB, FGF2, GAP43, GJA1, ID2, IL6, IRF6, KCNJ2, MAPK3, PLA2G6, PPARG, PRKCA, TNF* |
| *IGHE* | Other | *FGF2, IGF1R, IL6, RAC1, TP53, VAV1* | 1.12 x 10^-9^ | *ADIPOQ, ARPC2, BDNF, CAV3, CCL11, CDK5R1, CTNNB1, CTSB, FST, FZD1, GATA3, GJA1, HAS2, HEY1, ID2, IGF1R, IL5, IL6, MAFF, MAPK3, PLD1, RAC1, TGM2, TNF* |
| Salsalate | Chemical drug | *ADIPOQ, CTNNB1, GSN, IL6, TNF, TP53* | 1.12 x 10^-9^ | *AQP1, AXIN2, BMP4, COL4A1, EDNRA, FGF2, FZD3, GAP43, GATA3, GJA1, GLI2, HHIP, ID2, IL6, MAPK9, PLPP3, PTCH1, PTCH2, RAC1, SDC2, TGM2, TNF, TP53, WNT11* |
| Pinosylvin | Chemical - endogenous non-mammalian | *APP, BDNF, FGF2, GJA1, RAC1, TP53* | 1.17 x 10^-9^ | *ADM, APP, CTNNB1, CTSB, FGF2, GAP43, ID2, IL6, IRF6, KCNJ2, MAPK3, PLA2G6, PPARG, PRKCA, TNF, TP53* |
| *CBLL1* | Enzyme | *GHRL, GJA1, HAS2, IGF1R, IL5, IL6, PLD1, TNF* | 1.25 x 10^-9^ | *AXIN2, BMP4, CDC42, CDH3, COL4A1, CTNNB1, FZD3, GAP43, GATA3, GJA1, GLI2, HHIP, ID2, IL5, PPARG, PTCH1, PTCH2, SDC2, TNF, TP53, WNT11* |
| *AZU1* | Peptidase | *CALB1, CAPRIN1, CTNNB1, FST, GPM6A, GSN, MAPK10, PAFAH1B1, PDE2A, RAF1, SNX2, TAGLN, TNNC1* | 1.26 x 10^-9^ | *ADM, APP, CTNNB1, CTSB, FGF2, GAP43, ID2, IL6, IRF6, KCNJ2, MAPK3, PLA2G6, PPARG, PRKCA, TNF, TP53* |
| *WNT11* | Other | *BDNF, GATA3, IL5, IL6, KCNJ2, PPARG, RBPJ, STAT5B, TNF* | 1.27 x 10^-9^ | *ADM, APP, BDNF, CTNNB1, CTSB, FGF2, GAP43, HAS2, ID2, IL6, IRF6, KCNJ2, MAPK3, PLA2G6, PRKCA, TNF, TNNC1, TP53* |
| Budesonide | Chemical drug | *BDNF, PRKACA, TNF* | 1.28 x 10^-9^ | *CCL11, CDK5R1, GNA11, IL5, IL6, MAP2K4, MAP2K6, MAP2K7, PRKACA, PRKCA, TNF* |
| *C1QTNF2* | Other | *IL5, IL6, TNF* | 1.32 x 10^-9^ | *ADIPOQ, BDNF, CAV3, CCL11, CTSB, FGF2, FZD1, GATA3, GJA1, HAS2, IGF1R, ITIH5, LMO4, MAPK3, NFIB, PPARG, RAC1, RAF1, TGM2, TNF, TP53* |
| *PLGRKT* | Other | *IL6, TNF, TRAF6* | 1.54 x 10^-9^ | *ADIPOQ, BDNF, CCL11, CTNNB1, CTSB, DOCK1, FYN, IL6, PLA2G5, RAC1, RAC2, TNF, VAV1* |
| Pentosan polysulfate | Chemical drug | *FYN, IL6, TNF* | 1.56 x 10^-9^ | *AIF1, APP, AXIN2, BDNF, BMP4, CALB1, CTNNB1, ENPP2, FGF2, FYN, FZD1, GAP43, GJA1, IGF1R, IL6, LYVE1, MAPK11, MAPK3, PPARG, TAGLN, TGM2, TNF, TP53* |
| *GAP*  *(RASA1-A3)* | Group | *CCL11, IL5, IL6* | 1.59 x 10^-9^ | *ADM, APP, BDNF, CTNNB1, CTSB, FGF2, GAP43, GJA1, ID2, IL6, IRF6, KCNJ2, MAPK3, PLA2G6, PPARG, PRKCA, TNF* |
| *NFAT* complex  *(NFATC1-C4)* | Complex | *FGF2, PADI1, PRKCA* | 1.7 x 10^-9^ | *ADIPOQ, AIF1, AQP1, BDNF, CITED2, CTNNB1, ENPP2, GAP43, GATA3, IL5, IL6, LMO4, PLA2G2A, PLD1, PPARG, STAT5A, TNF* |
| *RTN4* | Other | *BDNF, IL6, TNF* | 1.8 x 10^-9^ | *ADM, AIF1, APP, BDNF, CTNNB1, CTSB, CTSH, DPYSL2, FGF2, GAP43, ID2, IL6, IRF6, KCNJ2, MAPK3, PLA2G6, PPARG, PRKCA, SDC2, SNAP25, TNF* |
| *PKC*(s)  *(PRKCA,*  *PRKCB,*  *PRKCD,*  *PRKCE,*  *PRKCG,*  *PRKCH,*  *PRKCI,*  *PRKCQ,*  *PRKCZ,*  *PRKD1,*  *PRKD3)* | Group | *GNAS, IL6, TNF* | 1.82 x 10^-9^ | *ADM, APP, CTNNB1, CTSB, FGF2, GAP43, ID2, IL6, IRF6, KCNJ2, MAPK3, PLA2G6, PPARG, PRKCA, TNF* |
| Meclo-fenamic acid | Chemical drug | *CALB1, COL4A1, GHRL, GNAS, IGF1R, IL6, KIT, PPARG, S100B, TNF* | 1.97 x 10^-9^ | *APP, AQP1, AXIN2, BMP4, COL4A1, EDNRA, FGF2, FZD3, GAP43, GATA3, GJA1, GLI2, HHIP, ID2, IL6, MAPK9, PTCH1, PTCH2, RAC1, SDC2, TGM2, TP53, WNT11* |
| *PAK5* | Kinase | *BDNF, BMP4, COL19A1, FCER1A, HAS2, PFN2, TAGLN, TGM2, TNNC1, TP53, VAV1* | 2.16 x 10^-9^ | *ADIPOQ, AIF1, APOD, APP, AQP1, BMP4, CDH3, CTNNB1, FYN, IL5, IL6, LMO4, MAPK11, MAPK3, PTCH1, TP53* |
| *CD69* | Trans-membrane receptor | *APOD, BMP4, CDC42, DPYSL2, ID2, IGF1R, MAPK3, MAPK9, PRKCA, TFDP1, WNT7A* | 2.24 x 10^-9^ | *ADIPOQ, BDNF, CAV3, CCL11, CITED2, CTSB, FGF2, FOXP1, FST, FZD1, GATA3, GJA1, HAS2, ID2, IGF1R, IL5, MAPK3, PIK3CA, RAC1, S1PR1, TNF, TP53* |
| *miR-141-3p* (and other miRNAs w/seed AACACUG) | Mature microRNA | *CCL11, HEY1, HEYL, IL6, MEF2A, MEF2C, TNF* | 2.24 x 10^-9^ | *AXIN2, CTNNB1, FST, GATA3, GLI2, HAS2, HEY1, IL5, IL6, INPP5D, IRF6, MAP2K4, RAC1, S1PR1, STAT5B, TAGLN, TNF* |
| *PLK4* | Kinase | *COL4A3BP, F11R, FST, HAS2, KIT, KITLG, PIK3R1* | 2.36 x 10^-9^ | *ADIPOQ, BDNF, CDC42, CITED2, DOCK1, FYN, IL5, IL6, PLA2G5, RAC1, RAC2, TNF, VAV1* |
| *APBA2* | Transporter | *ADIPOQ, IGF1R, IL6, PPARG, TNF* | 2.82 x 10^-9^ | *AIF1, APP, AXIN2, BDNF, CITED2, CTNNB1, CTSB, DPYSL2, ENPP2, FGF2, FZD3, GSK3B, HEY1, IL6, MAFF, PRKCA, PTCH2, RBPJ, S100B, S1PR1, TNF, TP53, TRAF6* |
| *SLC6A3* | Transporter | *ADIPOQ, IL6, PIK3R1, PPARG, TNF* | 2.97 x 10^-9^ | *ADIPOQ, ADM, AIF1, BDNF, CAV3, CDK5R1, CTNNB1, FYN, GLI2, IGF1R, IL5, IL6, KCNMA1, MAPK11, MAPK3, PPARG, RDH10, TNF, TP53* |
| *GRK4* | Kinase | *CDC42EP1, CHN1, CTNNB1, CTSH, IGF1R, IL6, PLD1, RAC1, TGM2, TNF* | 3.46 x 10^-9^ | *ADM, APP, AQP1, ARPC2, CAV3, CCL11, CDK5R1, CTNNB1, CTSB, FST, FZD1, GJA1, GNAS, HAS2, HEY1, IGF1R, IL5, KITLG, MAFF, MAPK3, MEF2C, RAC1, TGM2, TP53* |
| Rosuvastatin | Chemical drug | *CTNNB1, DPYSL2, FGF2, GSN, IL5, IL6, RAC1* | 3.8 x 10^-9^ | *ADIPOQ, BDNF, CCL11, CTNNB1, CTSB, DOCK1, FYN, IL6, KIT, PLA2G5, RAC1, RAC2, TNF, VAV1* |
| *FBXL2* | Enzyme | *IL6, PPARG, TNF, TP53* | 4.01 x 10^-9^ | *AIF1, APP, AXIN2, BDNF, CITED2, CTNNB1, CTSB, DPYSL2, ENPP2, FGF2, FZD3, GSK3B, HEY1, IL6, MAFF, PRKCA, PTCH2, RBPJ, S100B, S1PR1, TNF, TP53, TRAF6* |
| *APC/APC2* | Group | *APP, CTSB, RAC1, TNF* | 4.03 x 10^-9^ | *AXIN2, BMP4, COL4A1, CTNNB1, FZD3, GAP43, GATA3, GJA1, GLI2, HHIP, ID2, IGF1R, IL6, PLA2G10, PPARG, PTCH1, PTCH2, SDC2, TNF, TP53, WNT11* |
| *DSP* | Other | *CDC42EP1, COL4A1, FGF2, HAS2, IGF1R, IL6, MAPK3, RAC1, RAC2, S100B, TNF* | 4.03 x 10^-9^ | *ADIPOQ, APP, AQP1, AXIN2, CTNNB1, CTSB, EFNA1, GATA3, GSN, HAS2, IL5, IL6, IRF6, ITIH3, KRT17, PFN2, PPARG, TCF7L2, TMEM17, UCHL1, WNT11* |
| 8-epi-prosta-glandin F2alpha | Chemical - endogenous non-mammalian | *CTSB, ENPP2, GSK3B, ITIH4, NFIB, PRKCB, STMN1, TP53* | 4.47 x 10^-9^ | *ADIPOQ, CCL11, CDC42, COL4A1, CTSB, FGF2, FST, GJA1, HAND1, HEY1, IL6, PRKCA, PRKCB, TGM2, TNF, TP53* |
| *CD47* | Trans-membrane receptor | *ADM, FGF2, IL6, PPARG, TNF* | 4.66 x 10^-9^ | *CITED2, CTSB, CTSH, ELF5, FGF2, FOXP1, FST, FYN, GATA3, GSK3B, IL6, PIK3CA, PLA2G12A, PLA2G5, STAT5A, STAT5B, STMN1, TNF, TP53* |
| *KRT1* | Other | *ARPC2, FGF2, IGF1R, IL6, SEMA3C, TNF, TP53* | 5.23 x 10^-9^ | *ADIPOQ, BDNF, DOCK1, EDNRA, FYN, IL6, PLA2G5, RAC1, RAC2, TNF, VAV1* |
| *HOXA10* | Transcription regulator | *CTNNB1, DVL2, IL6, TAGLN, TNF, TP53* | 5.8 x 10^-9^ | *ADIPOQ, ADM, AIMP2, AQP1, ENPP2, FGF2, FST, FZD1, GJA1, HAS2, ID2, IL6, MAPK12, PIK3R1, S100B, SOS1, TAGLN, TNF, TP53* |
| *VCAN* | Other | *APP, DCN, FGF2, FST, GJA1, HAS2, IGF1R, IL5, IL6, PPARG, TNF, TP53* | 6.4 x 10^-9^ | *ADM, BDNF, CPE, DCN, ENPP2, FST, HAS2, MEF2C, MYH10, PLA2G2A, RBPJ, TCF7L2, TP53* |
| *GLI3* | Transcription regulator | *AIF1, CTSH, GAP43, IL6, SNAP25, TNF, TP53* | 7.7 x 10^-9^ | *ADIPOQ, APP, AXIN2, BDNF, BMP4, CCL11, CTSH, DCN, GJA1, GLI2, GNAS, HHIP, ID2, IL6, KRT5, LYVE1, MEF2C, PPARG, PRKCA, PTCH1, STAT5A, TDRD7, TNF, TP53, VAV1* |
| *FBN1* | Other | *BDNF, GATA5, KIT, PTCH1, TP53* | 8.13 x 10^-9^ | *AXIN2, CTNNB1, FST, GATA3, GLI2, HAS2, HEY1, IL5, IL6, INPP5D, PPARG, RAC1, S1PR1, TAGLN, TNF* |
| NFATC | Group | *ADIPOQ, AXIN2, CTNNB1, GATA3, PPARG* | 9.42 x 10^-9^ | *ADIPOQ, AIF1, BDNF, CITED2, ENPP2, GAP43, GATA3, IL5, IL6, LMO4, PLA2G2A, PLD1, PPARG, STAT5A, TNF* |
| *SMAD3/ SMAD4/ TOB* | Complex | *BMP4, IGF1R, MEF2C, PIK3CA, PIK3R1* | 9.95 x 10^-9^ | *AXIN2, BMP4, CDH3, CITED2, FST, GATA3, GLI2, HAS2, HEY1, ID2, IL5, IL6, INPP5D, RAC1, S1PR1, TAGLN, TNF, TP53* |
| *PSMD14* | Peptidase | *DCN, FST, IL6, TNF* | 1.07 x 10^-8^ | *AKT3, APP, BDNF, CDK5R1, CTSB, FGF2, FRZB, GAP43, GJA1, GSK3B, IGF1R, IL6, MAPK3, PRKCA, S1PR1, STAT5A, STMN1, TNF, TP53, WNT16* |
| *PLG* | Peptidase | *IL6, MAP2K4, TNF, TP53* | 1.09 x 10^-8^ | *ADIPOQ, BDNF, DOCK1, FYN, IL6, PLA2G5, RAC1, RAC2, TNF, VAV1* |
| *PARD3* | Other | *ENPP6, IL6, MAP2K4, TNF* | 1.09 x 10^-8^ | *AIF1, APP, CCL11, CTNNB1, FGF2, FYN, GSN, IFT27, IGF1R, IL5, IL6, MAPK11, MAPK3, RAC1, TCF7L2* |
| *EDN1* | Cytokine | *APP, RBPJ, TFDP1, TRAF6* | 1.23 x 10^-8^ | *ADIPOQ, CDC42, COL4A1, CTNNB1, FGF2, FST, GJA1, HAND1, HEY1, IL6, PRKCA, PRKCB, TGM2, TP53* |
| *SUFU* | Transcription regulator | *APP, IL6, PRKCA, TNF* | 1.28 x 10^-8^ | *ACADVL, BDNF, BMP4, GATA5, GLI2, HEY1, HHIP, ID2, IL6, KIT, KRT17, MAPK11, MEF2C, PTCH1, PTCH2, STMN3, TMEM100, TP53, WNT2B* |
| Rp-8-Br-cAMPS | Chemical - kinase inhibitor | *FGF2, FST, IL6, TP53* | 1.34 x 10^-8^ | *ADIPOQ, ADM, BDNF, CTNNB1, CTSB, DVL2, GHRL, GJA1, GNAS, HAS2, IGF1R, IL5, MAPK3, PLD1, PPARG, PRKACA, PRKCA, TNF, TP53* |
| *APP* | Other | *ADIPOQ, ADM, ARPC2, AXIN2, CITED2, FYN, GJA1, ID2, IL6, MAFF, MEF2C, PRKCA, TNF, TP53* | 1.36 x 10^-8^ | *AIF1, AXIN2, BDNF, CITED2, CTNNB1, CTSB, DPYSL2, ENPP2, FGF2, FZD3, GSK3B, HEY1, IL6, MAFF, PRKCA, PTCH2, RBPJ, S100B, S1PR1, TNF, TP53, TRAF6* |
| *FAM20B* | Enzyme | *FZD3, GLI2, HHIP, KRT5, PTCH1, PTCH2* | 1.42 x 10^-8^ | *EFNA1, FGF2, FST, IGF1R, IL6, KIT, S1PR1, TNF, TP53* |
| *CRHR*  *(CRHR1, CRHR2)* | Group | *AGPS, CTNNB1, FZD10, NFIB, TCF7L2, TNF, WNT11, WNT2B, WNT8B* | 1.5 x 10^-8^ | *ACADVL, BDNF, GATA5, GLI2, GNA11, HHIP, ID2, IL6, KCNMB1, MAPK11, MEF2C, PPARG, PTCH1, PTCH2, TMEM100, TP53, WNT2B* |
| *MGP* | Other | *AXIN2, CTNNB1, NME2* | 1.63 x 10^-8^ | *AXIN2, BMP4, CALB1, DPYSL2, FST, FZD1, GNAS, HAND1, HAS2, HEY1, ID2, IL6, MEF2C, PPARG, TAGLN, TGM2, TNF, TP53* |
| *DOCK3* | Other | *ADIPOQ, IL6, TNF* | 1.68 x 10^-8^ | *AIF1, APP, CCL11, CTNNB1, FGF2, FYN, GAP43, GJA1, GSN, IGF1R, IL5, MAPK11, MAPK3, PPARG, TGM2, TNF, TP53* |
| *GRB2/ SHC1/ SOS* | Complex | *APP, IL6, TNF* | 1.73 x 10^-8^ | *ADM, ARF1, BMP4, CTNNB1, CTSB, FGF2, FZD1, GJA1, HAS2, HEY1, IGF1R, IL6, MAPK12, MAPK3, MYH10, PLD1, TAGLN, TNF, TP53* |
| *TAOK1* | Kinase | *AXIN2, TNF, WNT11* | 2 x 10^-8^ | *ADIPOQ, AIF1, APOD, AQP1, BMP4, FYN, IL5, IL6, LMO4, MAPK11, MAPK3, MAPK9, MEF2C, PPARG, PTCH1, STK4, TNF* |
| *AQP1* | Transporter | *AQP1, GJA1, IL6* | 2.01 x 10^-8^ | *APP, AXIN2, BMP4, COL4A1, CTNNB1, FZD3, GAP43, GATA3, GJA1, GLI2, HHIP, ID2, PPARG, PTCH1, PTCH2, SDC2, TNF, TP53, WNT11* |
| Hymeni-aldisine | Chemical - endogenous non-mammalian | *IL6, TNF, TP53* | 2.43 x 10^-8^ | *AIF1, APP, AXIN2, FYN, IFT27, IGF1R, IL6, MAPK11, MAPK3, RAC1, TCF7L2, TNF, TP53* |
| *HOXA10* | Transcription regulator | *ADIPOQ, ADM, BDNF, CTSB, GJA1, GNAS, HAS2, IL6, LMO4, PPARG, PRKCA, TNF* | 2.45 x 10^-8^ | *ADIPOQ, ADM, AIMP2, AQP1, ENPP2, FST, FZD1, GJA1, ID2, MAPK12, PIK3R1, S100B, SOS1, TP53* |
| *LGALS3* | Other | *APP, CDC42, COL4A1, CTNNB1, ID2, IGF1R, IL6, KRT5, RAC1, TFDP1, TP53* | 2.76 x 10^-8^ | *ADIPOQ, COL4A1, ENPP2, IL5, IL6, PPARG, PRKCSH, RAC1, TNF* |
| *LTB4R* | G-protein coupled receptor | *APP, CALB1, CTSH, HAS2, IL5, IL6, PLA2G2A, PLD1, PPARG, TFAP2A, TNF* | 2.86 x 10^-8^ | *CITED2, CTSB, CTSH, FGF2, FOXP1, FST, GATA3, GJA1, GSK3B, GSN, IL5, IL6, PIK3CA, STMN1, TP53* |
| *GATA*  *(GATA1-6)* | Group | *BDNF, IL6, MYO10, PAFAH1B2, PPARG, TNF, TP53* | 2.87 x 10^-8^ | *ADM, ATP6V0D1, CDC42, COL4A1, EDNRA, FCER1A, FGF2, GATA3, GJA1, IL5, IL6, IRF6, KCNJ2, KRT17, LYVE1, MEF2C, MYO10, PLA2G2A, PPARG, SEMA3C, TAGLN, TGFB1I1, TGM2, TNF, TNNC1, WNT2, ZNF750* |
| *RIT2* | Enzyme | *IL6, PIK3CA, PIK3R1, PIK3R2, PPARG, TNF* | 2.96 x 10^-8^ | *CTSB, CTSH, EFNA1, GJA1, GNAS, GSK3B, HAS2, IL5, KITLG, MAP2K6, MEF2C, PLA2G2A, STMN1, TAGLN, TGM2, TP53* |
| *RIN1* | Other | *FGF2, IL6, KCNMA1, KCNMB1, PPARG, TNF* | 3.22 x 10^-8^ | *ADM, ARF1, BMP4, CTNNB1, CTSB, FGF2, FZD1, GJA1, HAS2, HEY1, IGF1R, IL6, MAPK12, MYH10, RAC1, TAGLN, TNF, TP53* |
| *HRAS* | Enzyme | *BDNF, GJA1, ID2, TAGLN, TCF7L2* | 3.49 x 10^-8^ | *ADM, ARF1, BMP4, CTNNB1, CTSB, FGF2, FZD1, GJA1, HAS2, HEY1, IGF1R, IL6, MAPK12, MYH10, TAGLN, TNF, TP53* |
| *SMAD3* | Transcription regulator | *CAV3, IGF1R, IL6, MEF2A, MEF2C* | 3.53 x 10^-8^ | *AXIN2, CTNNB1, FST, GATA3, GLI2, HAS2, HEY1, IL5, IL6, INPP5D, RAC1, S1PR1, TAGLN, TNF* |
| *ARFGEF2* | Other | *APP, BDNF, CALB1, FGF2, TNF* | 3.75 x 10^-8^ | *AXIN2, BMP4, COL4A1, FZD3, GAP43, GATA3, GJA1, GLI2, HHIP, ID2, PLA2G6, PPARG, PTCH1, PTCH2, SDC2, TNF, TP53, WNT11* |
| *NDP* | Growth factor | *ACADVL, GHRL, IGF1R, IL6, PPARG, RDH10, TNF, TP53* | 4.36 x 10^-8^ | *AXIN2, BDNF, BMP4, COL4A1, FGF2, FZD3, GAP43, GATA3, GJA1, GLI2, HHIP, ID2, PPARG, PTCH1, PTCH2, SDC2, TP53, WNT11* |
| *MFAP2* | Other | *APP, BDNF, CLTC, FGF2, GAP43, KCNQ3, TNF, TP53* | 4.51 x 10^-8^ | *CTNNB1, FGF2, GATA3, GLI2, HEY1, HEYL, ID2, IGF1R, IL6, LYVE1, PPARG, PTCH1, TAGLN, TNF, TP53* |
| *JUP* | Other | *APP, CTNNB1, GATA3, IL6* | 4.58 x 10^-8^ | *AXIN2, BMP4, COL4A1, CTNNB1, FZD3, GAP43, GATA3, GJA1, GLI2, HHIP, ID2, NME2, PPARG, PTCH1, PTCH2, SDC2, TP53, WNT11* |
| *STIM2* | Transporter | *FCER1A, INPP5D, PLCG1, PLCG2* | 5.43 x 10^-8^ | *BDNF, CITED2, ENPP2, GATA3, HAS2, IL5, LMO4, PLA2G2A, PLD1, PPARG, STAT5A, TNF, TP53* |
| *RLIM* | Enzyme | *CTNNB1, IL6, PPARG, TNF* | 6.17 x 10^-8^ | *CITED2, DCN, FRZB, FST, GATA3, HAS2, ID2, IL6, INPP5D, KIT, RAC1, S1PR1, TAGLN, TNF* |
| *ANTXR2* | Trans-membrane receptor | *IGF1R, IL6, MEF2A, MEF2C* | 6.31 x 10^-8^ | *ANXA7, CTNNB1, FGF2, GAP43, GJA1, IL6, TNF, TP53* |
| *CTNN beta/ LEF1* | Complex | *BMP4, CTNNB1, GJA1, ID2, IGF1R, PPARG, RAC1, SLC9A3R1, STMN1, TNF, TP53* | 6.76 x 10^-8^ | *AXIN2, BMP4, COL4A1, FZD3, GAP43, GATA3, GJA1, GLI2, HAS2, HHIP, ID2, IL5, PPARG, PTCH1, PTCH2, SDC2, TP53, WNT11* |
| Integrin beta | Group | *AXIN2, FOXP1, PPARG, TAGLN, TP53* | 7.07 x 10^-8^ | *CTNNB1, DCN, DVL2, ENPP2, FST, HAS2, IL6, MEF2A, PPARG, RAC1, RAC2, S1PR1, TP53, TRAF6* |
| *SKP1* | Transcription regulator | *APOD, HEY1, SDC2, TAGLN, TP53* | 7.07 x 10^-8^ | *ADIPOQ, CTNNB1, DVL2, ENPP2, ERCC3, ID2, IGF1R, KIT, KRT17, PPARG, STAT5A, STAT5B, TNF, TP53* |
| Alster-paullone | Chemical reagent | *APP, BDNF, DPYSL2, GAP43, IL6* | 7.09 x 10^-8^ | *AIF1, BDNF, CDK5R1, COL4A3BP, CTNNB1, EDNRA, FGF2, FYN, GSK3B, IL6, KCNMA1, MAP2K7, MAPK11, MAPK3, MEF2C, PPARG, PSEN2, TNNC1* |
| *PSMD10* | Transcription regulator | *CTSB, CTSH, IL6, TGM2, TNF* | 7.29 x 10^-8^ | *GNAS, IL6, PIK3CA, PIK3R1, PLD1, TP53* |
| Riboflavin | Chemical - endogenous mammalian | *APOD, APP, CCL11, FGF2, IL5, IL6, KIT, KITLG, PPARG, TNF* | 7.29 x 10^-8^ | *APC, FZD1, FZD6, PTCH1, SUFU, TNF* |
| *DCN* | Other | *CCL11, CTNNB1, CTSB, DCN, ENPP2, FYN, IL5, IL6, SEMA3C, TNF, TP53* | 1.07 x 10^-7^ | *FGF2, FST, IGF1R, IL6, KIT, S1PR1, TNF, TP53* |
| *KITLG* | Growth factor | *BDNF, FGF2, GNAS, IL6, NME2, TAGLN* | 1.19 x 10^-7^ | *AIF1, GJA1, ID2, IGF1R, IL5, IL6, KIT, MAPK11, MEF2C, PRKCA, PRKCB, TNF* |
| *LEMD3* | Other | *ADM, CCL11, CTSB, DCN, EHF, IL5, IL6, INPP5D, KITLG, PLA2G2A, S100B, TNF, TRAF6* | 1.37 x 10^-7^ | *COL4A1, DPYSL2, GLI2, HAND1, HAS2, HEY1, ID2, IL6, INPP5D, RAC1, S1PR1* |
| Nocodazole | Chemical reagent | *APP, S100B* | 1.53 x 10^-7^ | *GSK3B, MAP2K6, MAP2K7, MAPK12, MAPK3, PRKCB, RAF1, STK4, TP53* |
| Ar-a014418 | Chemical reagent | *PTCH1, PTCH2* | 1.74 x 10^-7^ | *AIF1, APP, CTNNB1, FYN, IFT27, IGF1R, IL6, MAPK11, MAPK3, PPARG, RAC1, TCF7L2, TP53* |
| *MAPKAPK2* | Kinase | *IL6, TNF* | 1.75 x 10^-7^ | *CCL11, CITED2, IL5, IL6, LMO4, STAT5A, TNF, TP53* |
| MEDIATOR  *(MED1-30, CCNC, CDK19, CDK8)* | Complex | *IL6, TNF* | 1.79 x 10^-7^ | *APP, AQP1, AXIN2, CTNNB1, CTSB, EFNA1, GSN, HAS2, IL6, IRF6, ITIH3, KRT17, PFN2, TAGLN, TCF7L2, TMEM17, TNNC1, TP53, UCHL1, WNT11* |
| *SPHK2* | Kinase | *IL6, TNF* | 1.90 x 10^-7^ | *APP, FYN, IL6, TNF, TP53* |
| Paroxetine | Chemical drug | *IL6, TNF* | 1.94 x 10^-7^ | *ADM, APP, BDNF, ID2, TNF, TP53* |
| *GDF9* | Growth factor | *IL6, TNF* | 2.09 x 10^-7^ | *ADIPOQ, BMP4, GJA1, HAS2, ID2, IL6, INPP5D, KIT, KITLG, MAPK3, PIK3R1, PPARG, RAC1, S1PR1, TGM2* |
| Pro-carbazine | Chemical drug | *IL6, TNF* | 2.13 x 10^-7^ | *APC, CALB1, FGF2, GATA3, IL6, ITIH5, LMO4, MAP2K4, NFIB, PPARG, RAF1, TGM2, TNF* |
| Lithium chloride | Chemical drug | *IL6, TNF* | 2.21 x 10^-7^ | *APP, AXIN2, BMP4, CTNNB1, GATA3, GSK3B, MAPK10, PLD1, PPARG, TP53* |
| *ACVRL1* | Kinase | *IL5, TNF* | 2.32 x 10^-7^ | *ANXA7, CTNNB1, EFNA1, GAP43, HAS2, ID2, INPP5D, LYVE1, RAC1, S1PR1, TAGLN* |
| *KLF15* | Transcription regulator | *IL6, TNF* | 2.36 x 10^-7^ | *ACADVL, CLTC, FST, GAP43, GATA3, GJA1, HAND1, IL6, KCNMB1, MEF2C, PLA2G2A, PPARG, S1PR1, TAGLN, TNF, TNNC1, TP53* |
| *TP63* | Transcription regulator | *IL6, TNF* | 2.49 x 10^-7^ | *ADM, CDC42, CITED2, COL4A1, CTNNB1, FST, IL6, PIK3CA, PIK3R3, RAC2, SUFU, TAGLN, TP53, UPK1A, UPK1B, UPK2* |
| *MMP3* | Peptidase | *IL6, TNF* | 2.53 x 10^-7^ | *AIF1, APP, BDNF, CAPRIN1, CCL11, CTSB, FGF2, FYN, GAP43, GJA1, IL6, IRF6, KIT, MAPK12, RAC1, TAGLN, TNF, TNNC1, TP53* |
| Morphine | Chemical drug | *IL6, TNF* | 2.53 x 10^-7^ | *ADM, AIF1, BDNF, CALB1, GATA3, GNAS, IL6, MAPK3, TNF, TP53, TRAF6* |
| Cyano-cobalamin | Chemical - endogenous mammalian | *PIK3R1, TP53* | 2.60 x 10^-7^ | *APC, APP, FZD1, FZD6, PTCH1, TNF* |
| *RRAD* | Enzyme | *IL6, TNF* | 2.75 x 10^-7^ | *BDNF, CDK5R1, COL4A3BP, EDNRA, FGF2, FZD1, HAS2, IL6, MAP2K7, MAPK3, MEF2C, PPARG, PSEN2, RAC1, TNNC1, TP53* |
| Ganglioside gq1b | Chemical - endogenous mammalian | *IL6, TNF* | 3.44 x 10^-7^ | *FGF2, GJA1, IL5, IL6, TNF, TP53* |
| *ATP2A3* | Transporter | *APP, PSEN2* | 3.61 x 10^-7^ | *APP, CAV3, CTSB, ID2, IL5, IL6, PLA2G6, PLD1, TNF, TP53* |
| *DTNBP1* | Other | *IL6, TNF* | 4.25 x 10^-7^ | *ADIPOQ, ADM, BDNF, BMP4, CTNNB1, CTSB, ENPP2, GJA1, GNAS, IL5, IL6, PPARG, PRKCA, SNAP25, TAGLN, TNF* |
| Cisapride | Chemical drug | *IL6, TNF* | 4.27 x 10^-7^ | *BDNF, FGF2, GJA1, IL6, KCNJ2, PPARG, TNF, TP53* |
| Glycol-sphingolipid | Chemical - other | *IL6, TNF* | 4.48 x 10^-7^ | *APP, BDNF, FYN, HEY1, HEYL, IL6, STAT5A, STAT5B, TNF* |
| *CSF3* | Cytokine | *IL6, TNF* | 4.56 x 10^-7^ | *ENPP2, GJA1, IL6, KIT, KITLG, NME2, PPARG, PRKCA, PRKCB, TFDP1, TNF, TP53* |
| Nor-epinephrine | Chemical - endogenous mammalian | *IL6, TNF* | 4.89 x 10^-7^ | *APP, BDNF, DNM2, FCER1A, FGF2, FST, HHIP, IGF1R, IL6, NPY1R, PPARG, PTCH1* |
| *TSHR* | G-protein coupled receptor | *IL6, TNF* | 5.27 x 10^-7^ | *CAV3, FGF2, GJA1, IL6, PIK3CA, PIK3R1, SOS1, TAGLN, TNF, TP53, VAV2* |
| *FSH*  *(CGA, FSHB)* | Complex | *BDNF, TNF* | 5.89 x 10^-7^ | *ADM, AXIN2, BDNF, CPE, FGF2, FST, IGF1R, IL6, KITLG, PLA2G10, SMARCA4, SNAP25, TP53* |
| 5'-guany-lylimidodi-phosphate | Chemical reagent | *IL6, TNF* | 6.15 x 10^-7^ | *APC, APP, CTSB, FGF2, FST, FYN, GATA3, GJA1, IL6, TFDP1, TP53* |
| Crt-0066101 | Chemical - kinase inhibitor | *CTNNB1, CTSB, GJA1, HAS2, IGF1R, IL6, PRKCA* | 6.15 x 10^-7^ | *ADIPOQ, GATA3, IL6, ITIH5, LMO4, NFIB, PPARG, RAF1, TGM2, TNF, TP53* |
| *SIK3* | Kinase | *GATA3, IL6, INPP5D, MAPK9, PRKCA, RAC1, SMARCA4, TNF* | 6.89 x 10^-7^ | *ADIPOQ, BDNF, CTNNB1, FST, IL6, MEF2C, SMARCA4, TAGLN, TNF* |
| *SALL4* | Transcription regulator | *APC, APP, CTNNB1, GJA1, IGF1R, IL6, PRKCA, TNF, TOLLIP, TP53* | 7.25 x 10^-7^ | *CTNNB1, EXT2, FRZB, HAND1, ID2, IGF1R, IL6, IRF6, KRT17, MEF2A, MEF2C, TDRD7, TNF, TP53, WNT8B* |
| Kpt-9274 | Chemical drug | *ADIPOQ, APP, CAV3, COL4A1, GJA1, IL6, ITIH4, TNF, TNNC1* | 7.33 x 10^-7^ | *BDNF, CTSB, IL6, NPY1R, TNF, TP53* |
| *GRM2* | G-protein coupled receptor | *BDNF, FGF2, GATA3, GHRL, GJA1, HAND1, IL6, TNF, TP53* | 8.31 x 10^-7^ | *BDNF, FGF2, GHRL, GJA1, HAS2, IGF1R, IL5, KCNJ2, PLD1, TNF, TP53* |
| Opioid | Chemical drug | *HAS2, IL5, IL6, TNF* | 9.67 x 10^-7^ | *ADM, AIF1, APP, BDNF, FYN, GJA1, IL6, MAPK11, MAPK3, TNF* |
| Cdki at7519 | Chemical drug | *ADIPOQ, IL6, TNF, TP53* | 1.05 x 10^-6^ | *APP, CTNNB1, IFT27, IGF1R, IL6, KITLG, RAC1, TCF7L2, TNF, TP53* |
| *A4GALT* | Enzyme | *IL6, TNF, TP53, TRAF6* | 1.12 x 10^-6^ | *ADIPOQ, BDNF, ID2, IL5, IL6, MYH10, TNF* |
| *NR6A1* | Ligand-dependent nuclear receptor | *CTNNB1, GSK3B, IL6, TNF* | 1.17 x 10^-6^ | *CTNNB1, EXT2, FRZB, GATA3, HAND1, IGF1R, IL6, IRF6, KRT17, MEF2A, MEF2C, TDRD7, TNF, TP53, WNT8B* |
| *MAPK8IP1* | Other | *CAV3, IL5, IL6, TNF* | 1.41 x 10^-6^ | *APP, BDNF, IL6, MAP2K7, TNF* |
| *SIRT6* | Enzyme | *APP, IL6, TNF, TP53* | 1.43 x 10^-6^ | *AKT3, GSK3B, IGF1R, IL6, MAPK3, TNF* |
| Nalbuphine | Chemical drug | *CCL11, IL5, IL6, TNF* | 1.43 x 10^-6^ | *ADM, FGF2, GJA1, IL6, TNF, TP53* |
| *ACVR2A* | Kinase | *IL6, TFDP1, TNF, TP53* | 1.46 x 10^-6^ | *BDNF, COL4A1, FST, HAS2, HEY1, ID2, IL6, INPP5D, RAC1, S1PR1* |
| *NHLRC1* | Enzyme | *CAV3, IL6, PRKCA, TP53* | 1.64 x 10^-6^ | *CTSB, GATA3, GSK3B, IL6, ITIH5, LMO4, NFIB, PPARG, PRKCA, RAF1, S1PR1, STAT5A, TGM2* |
| *SATB1* | Transcription regulator | *AIF1, GJA1, TNF, TP53* | 1.64 x 10^-6^ | *APC, GATA3, GATA5, IL5, IL6, KITLG, MEF2A, PRKCB, S1PR1, SS18L1, TBX6, TNF, TP53* |
| Sertindole | Chemical drug | *BDNF, FYN, IL6, STAT5A, STAT5B, TNF* | 2.01 x 10^-6^ | *ADM, APP, BDNF, IGF1R, IL5, IL6, KCNJ2, PPARG, TP53* |
| *Cdc42* | Enzyme | *BMP4, CTNNB1, TP53* | 2.13 x 10^-6^ | *CTSB, CTSH, FGF2, GSK3B, STMN1, TNF* |
| Clorgyline | Chemical drug | *IL6, PLA2G2A, PLA2G5* | 2.13 x 10^-6^ | *FGF2, GJA1, GNAS, IL6, TNF, TP53* |
| *CD34* | Other | *IL5, IL6, TNF* | 2.15 x 10^-6^ | *BDNF, CITED2, FOXP1, FST, FYN, GATA3, PIK3CA, STAT5A, STAT5B, TNF* |
| *GLI*  *(GLI1-3, GLIS1, HKR1)* | Group | *APP, IL6, KIT* | 2.31 x 10^-6^ | *ACADVL, BMP4, GATA5, HHIP, ID2, IL6, KRT17, MAPK11, MEF2C, PTCH1, PTCH2, TMEM100, TP53, WNT2B* |
| *CER1* | Cytokine | *CDC42, IL5, RAC1* | 2.37 x 10^-6^ | *CTNNB1, DPYSL2, HAND1, ID2, IL6, PPARG, SDC2, TNF* |
| Pergolide | Chemical drug | *IL6, PPARG, TNF* | 2.37 x 10^-6^ | *BDNF, FGF2, GJA1, IL6, KCNJ2, PPARG, TNF, TP53* |
| *PDGF/CC* | Complex | *GATA3, IL5, TNF* | 2.50 x 10^-6^ | *APP, CTNNB1, FGF2, GSK3B, IFT27, IGF1R, RAC1, TCF7L2, TNF, TP53* |
| *WNT10B* | Other | *IL5, IL6, TNF* | 2.87 x 10^-6^ | *APP, CTNNB1, IFT27, IGF1R, PPARG, RAC1, TCF7L2, TNF, TP53* |
| *PPP1R2* | Phosphatase | *GJA1, TNF, TP53* | 3.05 x 10^-6^ | *BDNF, ENPP2, ERCC3, IGF1R, IL6, KIT, KRT17, STAT5A, STAT5B, TNF, TP53* |
| *GLI1* | Transcription regulator | *ADM, APOD, APP, IL5, IL6, ITIH1, ITIH4, PPARG, TGM2, TNF* | 3.14 x 10^-6^ | *ACADVL, GATA5, GLI2, HHIP, ID2, MAPK11, MEF2C, PTCH1, PTCH2, TMEM100, TP53, WNT2B* |
| *GATA4* | Transcription regulator | *FGF2, GATA3, GLI2, IL5, IL6, PLA2G2A, TNF, TP53* | 3.24 x 10^-6^ | *CBY1, GATA3, GJA1, IL5, IRF6, KCNJ2, KRT17, MEF2C, PLA2G2A, TAGLN, TNNC1* |
| *ADCY*  *(ADCY1-10)* | Group | *ACADVL, ADM, APP, ARF1, DPYSL2, GAP43, GATA3, ID2, IGF1R, IL6, MAPK12, PIK3R1, PPARG, PRKCA, STMN1, TNF* | 3.43 x 10^-6^ | *FGF2, GJA1, IL6, TNF, TP53* |
| *SATB1* | Transcription regulator | *CTNNB1, MEF2C, PPARG, TAGLN, TNF* | 3.45 x 10^-6^ | *APC, GATA3, GATA5, IL5, KITLG, MEF2A, PRKCB, S1PR1, SS18L1, TBX6, TNF* |
| Ioperamide | Chemical drug | *APP, BDNF, CAPRIN1, CCL11, CTNNB1, DCN, ENPP2, FGF2, GAP43, ID2, IL5, IL6, KIT, PLA2G2A, PPARG, SNX10, TGM2, TNF, TP53, TRAF6* | 3.49 x 10^-6^ | *ADM, BDNF, CTNNB1, GJA1, IL6, KCNJ2, TP53* |
| *IGFBP3* | Other | *APC, APP, CDK5R1, CTSB, EDNRA, IL6, SDC2, TGM2, TNF, TP53* | 3.70 x 10^-6^ | *ADIPOQ, CCL11, IL5, LYVE1, SDC2, TNF* |
| *NOTCH2* | Transcription regulator | *ADIPOQ, HAS2, IL6, MEF2C, PPARG, TAGLN, TBX6, TNF* | 3.70 x 10^-6^ | *HEY1, IL6, SDC2, TAGLN, TNF, TP53* |
| Vincristine | Chemical drug | *ENPP2, IL5, PTCH1, TNF* | 3.70 x 10^-6^ | *GAP43, MAP2K4, MAP2K7, MAPK9, PRKCA, TP53* |
| Rp 73401 | Chemical toxicant | *APP, IL5, IL6, TNF* | 3.94 x 10^-6^ | *ADIPOQ, ADM, BDNF, CCL11, CTNNB1, CTSB, GJA1, GNAS, IL5, IL6, PPARG, PRKCA, TNF* |
| Nitrendipine | Chemical drug | *IL6, KIT, RAC1, TNF* | 3.97 x 10^-6^ | *APP, BDNF, CTNNB1, GJA1, IL6, KCNJ2, TP53* |
| *GH1* | Growth factor | *APC, GJA1, IGF1R, IL6, TNF, TP53* | 4.15 x 10^-6^ | *APC, BMP4, CTNNB1, ERCC3, GHRL, ID2, IGF1R, IL6, PPARG, SMARCA4, TFAP2A* |
| *MGMT* | Enzyme | *ADIPOQ, GJA1, IL6, TNF, TP53* | 4.44 x 10^-6^ | *APC, GATA3, IL6, ITIH5, LMO4, NFIB, PPARG, RAF1, TGM2, TP53* |
| *GATA6* | Transcription regulator | *ADIPOQ, APP, ASAP1, CTNNB1, F11R, FST, FZD1, IL6, PLA2G2A, PLA2G3, PPARG, TAGLN, TNF, TP53, TRAF6* | 4.68 x 10^-6^ | *ATP6V0D1, COL4A1, FGF2, IRF6, KRT17, LYVE1, MEF2C, SEMA3C, TAGLN, TNNC1, WNT2* |
| *NFATC2* | Transcription regulator | *ADIPOQ, CTNNB1, GJA1, IL6, PPARG, TNF, TP53* | 5.09 x 10^-6^ | *CITED2, ENPP2, GATA3, IL5, LMO4, PLA2G2A, PLD1, PPARG, STAT5A, TNF* |
| *CLEC1B* | Trans-membrane receptor | *FGF2, IL6, MYH10, PLA2G2A, TAGLN, TNF* | 5.12 x 10^-6^ | *CITED2, FGF2, FOXP1, FST, GATA3, IL6, PIK3CA, PLCG2, TNF* |
| *PLA2G10* | Enzyme | *APC, CAPRIN1, COL4A1, CTNNB1, EFNA1, EXT2, IGF1R, RBPJ, SOS1, TFAP2A* | 5.71 x 10^-6^ | *BMP4, GATA3, IL5, IL6, TNF* |
| *RYR2* | Ion channel | *DPYSL2, EDNRA, ELF5, GATA3, MYO10, PFN2, SLC9A3R1, SNAP25, STAT5A, STAT5B, TNF* | 5.98 x 10^-6^ | *ADIPOQ, ADM, BDNF, CTNNB1, CTSB, GJA1, GNAS, IL6, PPARG, PRKCA, TNF, TP53* |
| *PRKAA2* | Kinase | *AXIN2, ID2, LMO4* | 5.99 x 10^-6^ | *GATA3, IL6, ITIH5, LMO4, NFIB, PPARG, RAF1, TGM2, TP53* |
| *BBS12* | Other | *CTSB, IL6, TNF* | 7.07 x 10^-6^ | *ADIPOQ, IL6, TNF* |
| *CTDNEP1* | Phosphatase | *IL5, IL6, TNF* | 7.07 x 10^-6^ | *AXIN2, CTNNB1, DVL2* |
| *CYP11B2* | Enzyme | *FGF2, IL6, TNF* | 7.07 x 10^-6^ | *IGF1R, KCNMA1, KCNMB1* |
| *PPP1CC* | Phosphatase | *CTNNB1, TNF, TP53* | 7.07 x 10^-6^ | *BDNF, IL6, TNF* |
| Cromolyn | Chemical drug | *APP, GSN, IL6, TNF* | 7.07 x 10^-6^ | *APP, IL6, TNF* |
| *SOST* | Other | *ANXA7, CTNNB1, GAP43, IL6* | 7.08 x 10^-6^ | *BMP4, GLI2, ID2, IL6, INPP5D, MYH10, TAGLN, WNT2* |
| *HMGA1* | Transcription regulator | *CCL11, GATA3, IL5, TNF* | 7.10 x 10^-6^ | *COL4A1, CPE, CSK, CTSH, GSN, HAND1, IL6, KITLG, PSEN2, TAGLN* |
| Dimethyl-nitrosamine | Chemical toxicant | *GATA3, IL5, IL6, PPARG* | 7.15 x 10^-6^ | *COL4A1, IL6, MAPK3, RAB13, TGM2, TNF* |
| *FRAT2* | Other | *APP, CTNNB1, FGF2, IL6, PRKCB* | 7.15 x 10^-6^ | *AIF1, CTNNB1, FYN, IL6, MAPK11, MAPK3* |
| *NAMPT* | Cytokine | *BDNF, BLOC1S6, DNM2, DVL2, F11R, GSK3B, GSN, MYO10, RDH10, TRAF6* | 7.23 x 10^-6^ | *BDNF, IL6, NPY1R, TNF, TP53* |
| Azoxy-methane | Chemical toxicant | *FZD3, FZD9, IRF6, TBX6, WNT2, WNT2B* | 7.23 x 10^-6^ | *CTNNB1, GSK3B, IGF1R, IL6, PRKCA* |
| *PRNP* | Other | *BDNF, ENPP2, FST, GAP43, GATA3, IL6, MAP2K7, NPY1R, SNAP25, TNF* | 8.13 x 10^-6^ | *FST, GJA1, IGF1R, IL6, PLPP3, SNAP25, TP53* |
| *GATA4* | Transcription regulator | *CTNNB1, IL6, TNF, TP53, TRAF6* | 9.16 x 10^-6^ | *GATA3, GJA1, IL5, IRF6, KCNJ2, KRT17, MEF2C, PLA2G2A, TAGLN, TNNC1* |
| *MIR-15* | MicroRNA | *BDNF, CALB1, GAP43, GJA1, IGF1R, IL6, KCNH1, RAF1, S1PR1, TNF, TP53* | 9.65 x 10^-6^ | *APP, CDC42, FGF2, MAPK3, RAF1, TP53* |
| *MAPK10* | Kinase | *ADIPOQ, ADM, BDNF, BMP4, GNAS, GPM6A, HEY1, IL6, MAP2K4, PPARG, STAT5B, TNF, TP53* | 9.83 x 10^-6^ | *APP, IL6, TNF, WNT7A* |
| Rimonabant | Chemical drug | *ADIPOQ, IL6, INPP5D, TNF* | 1.12 x 10^-5^ | *ADIPOQ, FGF2, IL6, TNF, TP53* |
| Alpha-tocopherol | Chemical drug | *BDNF, IL6, TNF, TP53* | 1.12 x 10^-5^ | *ADIPOQ, IL6, PPARG, TNF, TP53* |
| NUMB/NUMBL | Group | *EDNRA, ID2, IL6, TNF* | 1.20 x 10^-5^ | *APP, GATA3, HEY1, ID2, IL6, TAGLN, TNF, TP53* |
| Romidepsin | Biologic drug | *GAP43, GJA1, IL6, MEF2C* | 1.28 x 10^-5^ | *CDC42, FGF2, GSN, RAC1, RAF1, TP53* |
| *SOSTDC1* | Other | *BDNF, CTNNB1, EDNRA, GATA3, GLI2, IL6, SEMA3C, TNF* | 1.28 x 10^-5^ | *DPYSL2, GLI2, HAND1, ID2, IL6, INPP5D* |
| *TWSG1* | Other | *CDC42EP2, CDK5R1, COL4A1, EFNA1, IL6, INPP5D, MAP2K4, MEF2C, PIK3CA, PLD1, PRKCB, TGM3, TNF* | 1.30 x 10^-5^ | *COL4A1, HAS2, HEY1, ID2, IL6, INPP5D, RAC1, S1PR1* |
| *LGR4* | Trans-membrane receptor | *APP, IL6, KIT, RAF1, STAT5A, TNF* | 1.41 x 10^-5^ | *AXIN2, IL6, TNF, WNT7A* |
| *IL25* | Cytokine | *APP, BDNF, DNM2, SNAP25, TNF* | 1.47 x 10^-5^ | *CCL11, FGF2, GATA3, IL5, IL6, TNF* |
| Rhodamine-123 | Chemical reagent | *ARFIP2, CTNNB1, IL6, INPP5D, MYO10, PIK3R1, TCF7L2, TNF* | 1.47 x 10^-5^ | *APP, CCL11, IL5, PPARG, TNF, TP53* |
| *JAG1* | Growth factor | *FGF2, MAPK3, PPARG* | 1.67 x 10^-5^ | *GLI2, HEY1, HEYL, TAGLN, TP53* |
| *RSPO3* | Kinase | *APP, IL6, TNF* | 1.75 x 10^-5^ | *AXIN2, IL6, TNF* |
| *IgG2B (IGG2)* | Complex | *IL5, IL6, TNF* | 1.75 x 10^-5^ | *APP, IL6, TNF* |
| *ROR2* | Kinase | *ADIPOQ, IL6, TNF* | 1.75 x 10^-5^ | *AXIN2, CTNNB1, PPARG* |
| *CDH13* | Other | *IL6, PLCG2, TNF* | 1.75 x 10^-5^ | *ADIPOQ, AKT3, CTNNB1* |
| *Ppp1cc* | Phosphatase | *IL6, PPARG, TNF* | 1.75 x 10^-5^ | *ID2, IL6, TNF* |
| *PSENEN* | Peptidase | *ITIH1, PPARG, TNF* | 1.75 x 10^-5^ | *APP, PSEN2, TP53* |
| Magnesium chloride | Chemical drug | *GATA3, IL5, TNF* | 1.75 x 10^-5^ | *IL5, IL6, TNF* |
| Formestane | Chemical drug | *APC, FZD1, FZD6* | 1.80 x 10^-5^ | *ACADVL, BDNF, GSK3B, IL6, PLD1, PPARG, TNF, TP53* |
| *CYP19A1* | Enzyme | *FGF2, IL6, TNF* | 1.88 x 10^-5^ | *ACADVL, BDNF, GSK3B, IL6, PPARG, TNF, TP53* |
| *LRP8* | Trans-membrane receptor | *CCL11, CTNNB1, E2F8, IGF1R, IL5, IL6, S1PR1, STMN1, TGM2, TNF* | 1.91 x 10^-5^ | *AIF1, FYN, IL6, MAPK11, MAPK3, TNF* |
| *ENPP2* | Enzyme | *ADM, APP, COL4A1, CTNNB1, DCN, E2F8, IL6, KIT, PLA2G12A, PPARG, TNF, TP53* | 1.95 x 10^-5^ | *ADIPOQ, IL6, PPARG, TNF* |
| Gamma-tocotrienol | Chemical drug | *IL5, IL6, KCNJ2, MAFF, NME7, PLD1, PLPP3, PPARG, TNF* | 1.95 x 10^-5^ | *CTNNB1, IL6, TNF, TRAF6* |
| 9z-tetra-decenoic acid | Chemical - endogenous mammalian | *ADIPOQ, GAP43, GATA3, IL5, IL6, PIK3R3, TNF* | 1.95 x 10^-5^ | *ADIPOQ, IL6, TNF, TP53* |
| Apo-morphine | Chemical drug | *EFNA1, ID2, LYVE1, TAGLN* | 2.01 x 10^-5^ | *APC, BDNF, FGF2, GJA1, IGF1R* |
| *EFHD2* | Other | *AIF1, APP, BDNF, TNF* | 2.11 x 10^-5^ | *CITED2, FGF2, FOXP1, FST, GATA3, IL6, PIK3CA, TNF* |
| *EPHB4* | Kinase | *APP, IL6, TMEM100, TNF* | 2.11 x 10^-5^ | *BMP4, GATA3, IL6, KIT, KITLG, STAT5B, TNF, WNT11* |
| MIR-199a-5p (and other miRNAs w/seed CCAGUGU) | Mature microRNA | *APOD, AQP1, BMP4, IL5, IL6, PTCH1, TNF, TP53* | 2.28 x 10^-5^ | *COL4A1, DCN, KRT17, LIN7C, TAGLN, TGFB1I1, WNT7A* |
| *LRP2* | Transporter | *APC, CTNNB1, EFNA1, FGF2, IL6, TNF* | 2.40 x 10^-5^ | *BMP4, CTSB, GLI2, ID2, INPP5D* |
| *CHRD* | Other | *ADIPOQ, AXIN2, CAV3, GATA3, GSK3B, ID2, IGF1R, IL6, IRF6, KCNIP3, MEF2C, TNF, TP53, WNT8B* | 2.40 x 10^-5^ | *DPYSL2, HAND1, ID2, IL6, PTCH1* |
| *GFI1* | Transcription regulator | *ADIPOQ, APP, BDNF, DPYSL2, IL6, PIK3R1, RAF1, TNF* | 2.50 x 10^-5^ | *GJA1, ID2, IL6, MAPK3, RAF1, TNF, TOLLIP* |
| *SELENOS* | Other | *CDC42EP2, CTNNB1, EDA, ERCC3, FGF2, FST, ID2, IL6, KIT, PIK3R3, STMN1, TP53* | 2.63 x 10^-5^ | *ADIPOQ, IL6, PPARG, TNF* |
| *NFATC4* | Transcription regulator | *ADIPOQ, BDNF, CTNNB1, GATA5, GLI2, IGF1R, IL6, MEF2C, TNF, TP53* | 2.63 x 10^-5^ | *ADIPOQ, BDNF, PPARG, TNF* |
| *EREG* | Growth factor | *CCL11, CTNNB1, CTSB, IL6, TNF* | 2.63 x 10^-5^ | *FGF2, HAS2, IL6, TNF* |
| *SPINT1* | Other | *CCL11, IL6, PPARG, TAGLN, TNF, TP53* | 2.63 x 10^-5^ | *CTNNB1, FZD3, FZD6, WNT11* |
| L-685,458 | Chemical - protease inhibitor | *ADIPOQ, ASAP1, AXIN2, FZD1, PLA2G3, TBX6* | 2.63 x 10^-5^ | *APP, HEY1, TNF, TP53* |
| Cerdulatinib | Chemical drug | *AXIN2, GJA1, IL6, PPARG, TNF, WNT7A* | 2.65 x 10^-5^ | *CITED2, FOXP1, FST, GATA3, IL6, PIK3CA, STAT5A, TNF* |
| Anandamide | Chemical - endogenous mammalian | *ADIPOQ, APP, IL6, PPARG, TNF, UPK2* | 2.86 x 10^-5^ | *FGF2, IL6, PPARG, TNF, TP53* |
| Tauro-lithocholic acid | Chemical - endogenous mammalian | *APP, IL5, IL6, PRKCA, PRKCB, TNF* | 2.86 x 10^-5^ | *CTNNB1, GJA1, IL6, TNF, TP53* |
| *GHR* | Trans-membrane receptor | *ADM, CITED2, GJA1, IL6, ITIH5, MAFF, PIK3CA, PPARG, PRKCA* | 3.37 x 10^-5^ | *ADIPOQ, APC, IL6, TNF, TP53* |
| Thalidomide | Chemical drug | *BMP4, CTNNB1, PPARG, WNT7A* | 3.37 x 10^-5^ | *APP, FGF2, IL5, IL6, TNF* |
| *PHLPP2* | Enzyme | *IGF1R, IL5, IL6, TNF* | 3.47 x 10^-5^ | *PRKCA, PRKCB, TNF* |
| *WNT2* | Cytokine | *CCL11, CDH3, CTSH, FGF2, GAP43, ID2, IL5, IL6, KRT17, MYH10, PPARG, STK4, TNF, TNNC1, TP53, UPK1A* | 3.47 x 10^-5^ | *CTNNB1, DVL2, GSK3B* |
| *DUSP10* | Phosphatase | *AIF1, BDNF, CTSB, GSK3B, GSN, TNF, TP53, UCHL1* | 3.47 x 10^-5^ | *IL6, TNF, TP53* |
| *DHH* | Peptidase | *ADIPOQ, CSK, IL6, MEF2C, TGM2, TMEM100, TNF, TP53* | 3.47 x 10^-5^ | *GJA1, HHIP, PTCH1* |
| Latrunculin A | Chemical toxicant | *APP, CCL11, GAP43, GJA1, IL5, IL6, TGM2, TNF, TP53* | 3.47 x 10^-5^ | *HAS2, IL6, TNF* |
| Propyl-gallate | Chemical toxicant | *ADIPOQ, CAPZB, IL6, MEF2C, PIK3R3, PLA2G3, PPARG, TNF, TP53* | 3.47 x 10^-5^ | *PLA2G2A, TNF, TP53* |
| *DSP* | Other | *ADM, FGF2, IGF1R, IL5, IL6, PSEN2, RAF1, TNF, TP53* | 3.48 x 10^-5^ | *ADIPOQ, AXIN2, CTNNB1, PPARG* |
| Cyclo-piazonic acid | Chemical - endogenous non-mammalian | *APOD, BMP4, GJA1, KRT5, PTCH1, SEMA3C* | 3.48 x 10^-5^ | *IL5, IL6, S100B, TNF* |
| *EPHB4* | Kinase | *IL6, TNF* | 3.49 x 10^-5^ | *BMP4, GATA3, KIT, KITLG, STAT5B, WNT11* |
| Eft508 | Chemical drug | *IL5, IL6* | 3.54 x 10^-5^ | *APC, GAP43, PAFAH1B1, SDC2, SNAP25, TAGLN, TFDP1, TNF* |
| *GLI2* | Transcription regulator | *APP, PPARG* | 3.91 x 10^-5^ | *BMP4, HHIP, IL6, KRT17, MEF2C, PTCH1* |
| 1-methyl-4-phenyl-1,2,3,6-tetrahydropyridine | Chemical toxicant | *IL6, TNF* | 3.91 x 10^-5^ | *CALB1, FGF2, IL6, MAP2K4, TNF, TP53* |
| *HOXB9* | Transcription regulator | *IL6, TNF* | 3.91 x 10^-5^ | *ADIPOQ, FGF2, IGF1R, IL6, TGM2, TP53* |
| Green tea polyphenol | Chemical drug | *IL6, TNF* | 3.96 x 10^-5^ | *ADIPOQ, IL6, PIK3R1, PPARG, TP53* |
| Ibuprofen | Chemical drug | *IL6, TNF* | 3.96 x 10^-5^ | *APP, IL6, PPARG, RAC1, TNF* |
| *UBE2K* | Transcription regulator | *BDNF, FGF2* | 4.45 x 10^-5^ | *BDNF, COL4A1, DCN, KCNJ2, MEF2A, MEF2C, TBX6, TGM2, TP53* |
| *NPM1* | Transcription regulator | *AXIN2, DVL2* | 4.51 x 10^-5^ | *CTNNB1, IL6, TNF, TP53* |
| Alpha-tocopherol succinate | Chemical drug | *APP, PSEN2* | 4.51 x 10^-5^ | *FGF2, MAPK3, TNF, TP53* |
| Histone deacetylase inhibitor | Chemical drug | *BDNF, TP53* | 4.62 x 10^-5^ | *FGF2, GSN, RAF1, SLC9A3R1, TP53* |
| Pravastatin | Chemical drug | *IL6, TNF* | 4.62 x 10^-5^ | *ADIPOQ, GJA1, IL6, TNF, TP53* |
| Uridine triacetate | Chemical drug | *BDNF, GAP43* | 4.62 x 10^-5^ | *AIF1, BDNF, FYN, MAPK11, MAPK3* |
| *NTN1* | Other | *HHIP, PTCH1* | 5.74 x 10^-5^ | *CCL11, CTSB, IL6, TNF* |
| *ITGA6* | Trans-membrane receptor | *IL6, TNF* | 5.74 x 10^-5^ | *CTNNB1, DVL2, ENPP2, TP53* |
| Sertraline | Chemical drug | *IL6, TNF* | 5.74 x 10^-5^ | *BDNF, IL6, TNF, TP53* |
| *TIMD4* | Other | *IL6, TNF* | 5.74 x 10^-5^ | *GATA3, IL5, IL6, TNF* |
| *ATP7A* | Transporter | *IL6, TNF* | 5.74 x 10^-5^ | *APP, IL6, PLA2G2A, PLA2G5* |
| *MYOCD* | Transcription regulator | *IL6, TNF* | 5.89 x 10^-5^ | *GJA1, HAND1, KCNMB1, MEF2C, PLA2G2A, TAGLN, TNNC1* |
| *SYK* | Kinase | *IL6, TNF* | 5.89 x 10^-5^ | *CITED2, FOXP1, FST, GATA3, IL6, PIK3CA, TNF* |
| *SERPINC1* | Enzyme | *IL6, TNF* | 6.02 x 10^-5^ | *IL6, MAPK3, TNF* |
| *MGLL* | Enzyme | *IL6, TNF* | 6.02 x 10^-5^ | *APP, IL6, TNF* |
| Lidocaine | Chemical drug | *PPARG, TNF* | 6.02 x 10^-5^ | *APP, GATA3, IL6* |
| *TTR* | Transporter | *IL6, TNF* | 6.19 x 10^-5^ | *AIF1, APP, FYN, MAPK11, MAPK3* |
| Benzo-diazepines | Chemical drug | *GSK3B, TP53* | 6.19 x 10^-5^ | *APP, BDNF, HEY1, HEYL, KCNJ2* |
| Epi-allopreg-nanolone | Chemical - endogenous mammalian | *IL6, TNF* | 6.19 x 10^-5^ | *AIF1, FYN, MAPK11, MAPK3, TNF* |
| *EHF* | Transcription regulator | *KITLG, PTCH2* | 6.89 x 10^-5^ | *BMP4, CDH3, FCER1A, IL6, KIT, RBPJ, STAT5B* |
| *APH-1* | Group | *IL6, TNF* | 7.12 x 10^-5^ | *APP, HEY1, HEYL, PSEN2, TP53* |
| Chloral hydrate | Chemical drug | *IL6, TNF* | 7.12 x 10^-5^ | *AIF1, FYN, MAPK11, MAPK3, PTCH1* |
| Cep-1347 | Chemical drug | *IL6, TNF* | 7.20 x 10^-5^ | *BDNF, IL6, MAP2K4, TNF* |
| *REST* | Transcription regulator | *GSN, RAC2* | 7.31 x 10^-5^ | *BDNF, GAP43, KCNQ3, S100B, SNAP25, UCHL1* |
| Pano-binostat | Chemical drug | *IL6, TNF* | 8.15 x 10^-5^ | *CDC42, CITED2, KIT, RAC1, SLC9A3R1* |
| *AKT2* | Kinase | *IL6, TNF* | 8.15 x 10^-5^ | *CTNNB1, IGF1R, PPARG, RAC1, TNF* |
| *HDAC4* | Transcription regulator | *FGF2, IL6* | 8.15 x 10^-5^ | *BDNF, FST, IL6, MEF2C, TAGLN* |
| Calpain  (*CAPN1-11, CAPNS1*) | Complex | *IL6, TNF* | 8.91 x 10^-5^ | *CTNNB1, GJA1, IL6, TP53* |
| *LRPAP1* | Other | *IL6, TNF* | 8.91 x 10^-5^ | *APP, IL6, MAPK3, TNF* |
| Acetyl-choline | Chemical - endogenous mammalian | *IL6, TNF* | 8.91 x 10^-5^ | *IL6, KCNQ3, RAC1, TNF* |
| *ERG* | Transcription regulator | *IL5, TNF* | 9.40 x 10^-5^ | *CTNNB1, DOCK1, FYN, MYO10, PLPP3, TGFB1I1, WNT11, WNT2* |
| 12(S)-hydroxy-eicosate-traenoic acid | Chemical - endogenous non-mammalian | *IL6, TNF* | 9.54 x 10^-5^ | *IL6, PPARG, TNF* |
| 2',3'-dialdehyde ATP | Chemical reagent | *IL6, TNF* | 9.54 x 10^-5^ | *IL6, PLA2G5, TNF* |
| Ibudilast | Chemical drug | *IL6, TNF* | 9.54 x 10^-5^ | *IL6, NCS1, TNF* |
| *APH1A* | Peptidase | *IL6, TNF* | 9.54 x 10^-5^ | *APP, PSEN2, TP53* |
| *PFKFB3* | Kinase | *IL6, TNF* | 9.54 x 10^-5^ | *ADIPOQ, IL6, TNF* |
| *RGS19* | Other | *BDNF, BMP4, FGF2, GATA3, IL6, KIT, NFIB, PRKCA, PRKCB, TNF, TP53* | 9.54 x 10^-5^ | *AXIN2, BMP4, MEF2C* |
| *RALBP1* | Enzyme | *BDNF, CTNNB1, GATA3, GSK3B, IL6* | 9.54 x 10^-5^ | *COL4A1, IL6, TNF* |
| *MEOX1* | Transcription regulator | *ADIPOQ, BDNF, EDNRA, GJA1, IL6, MAPK3, PPARG, S100B, TMEM17* | 9.54 x 10^-5^ | *BMP4, GLI2, MEF2C* |
| *IL18R1* | Trans-membrane receptor | *GLI2, HEY1, RBPJ* | 9.54 x 10^-5^ | *IL5, IL6, TNF* |
| Magnesium sulfate | Chemical drug | *ADIPOQ, IL6, TNF* | 9.54 x 10^-5^ | *ADM, IL6, TNF* |
| Itf3056 | Chemical reagent | *IL5, IL6, TNF* | 9.54 x 10^-5^ | *IL6, TNF, TP53* |
| *SOX9* | Transcription regulator | *IL5, IL6, TNF* | 9.73 x 10^-5^ | *CTNNB1, KIT, MAPK3, PPARG, PRKCA, TCF7L2* |
| Flurazepam | Chemical drug | *KIT, PIK3R1, STAT5A* | 1.06 x 10^-4^ | *AIF1, FYN, MAPK11, MAPK3, TNF* |
| *MYB* | Transcription regulator | *BDNF, HEY1, IL6* | 1.08 x 10^-4^ | *COL4A1, GATA3, IGF1R, IL5, KIT, KITLG, PDE2A* |
| Daidzein | Chemical drug | *ID2, IL6, TNF* | 1.15 x 10^-4^ | *ADIPOQ, AIF1, BDNF, ID2, IL6, PIK3R1, TP53* |
| 4-hydroxy-nonenal | Chemical toxicant | *ENPP2, IL6, TP53* | 1.19 x 10^-4^ | *APP, CTSB, IL6, SUFU, TNF* |
| *CX3CL1* | Cytokine | *GJA1, IL6, TNF* | 1.32 x 10^-4^ | *FGF2, IL6, PPARG, TNF* |
| *TCF7* | Transcription regulator | *BDNF, MAPK3, RAF1* | 1.32 x 10^-4^ | *AXIN2, CTNNB1, GATA3, SDC2* |
| Desipramine | Chemical drug | *APP, BDNF, KCNQ3* | 1.32 x 10^-4^ | *BDNF, FGF2, IL6, TNF* |
| *PRKACA* | Kinase | *CTSB, DCN, ENPP2, IL6, PPARG, SEMA3C, TNF* | 1.35 x 10^-4^ | *ADIPOQ, BDNF, ID2, IL5, MYH10* |
| *CMKLR1* | G-protein coupled receptor | *KCNB2, MAPK10, PPARG, PTCH1* | 1.42 x 10^-4^ | *ADIPOQ, IL6, TNF* |
| *UNC5B* | Trans-membrane receptor | *IGF1R, PIK3CA, RAC1, SOS1* | 1.42 x 10^-4^ | *CCL11, IL6, TNF* |
| *IGHG2B* | Other | *ADIPOQ, COL4A1, CTNNB1, FGF2, IGF1R, IL6, PIK3R1, PPARG, TNF, TP53* | 1.42 x 10^-4^ | *APP, IL6, TNF* |
| *CD48* | Other | *BDNF, DCN, FGF2, IL5, IL6, TNF, WNT16* | 1.42 x 10^-4^ | *CCL11, IL5, TNF* |
| *MZF1* | Transcription regulator | *GJA1, IL6, TAGLN, TGM2, TP53* | 1.42 x 10^-4^ | *FGF2, PADI1, PRKCA* |
| *SLC30A3* | Transporter | *BMP4, HAS2, IL6, TNF, WNT11* | 1.42 x 10^-4^ | *APP, BDNF, SNAP25* |
| Montelukast | Chemical drug | *GJA1, IL6, PPARG, TNF, TP53* | 1.42 x 10^-4^ | *CCL11, IL5, IL6* |
| Bq 123 | Chemical drug | *ACADVL, ADIPOQ, ADM, APC, APP, AQP1, AXIN2, BDNF, BMP4, CCL11, CDC42, CDH3, CDK5R1, CITED2, COL4A3BP, CTNNB1, CTSB, DCN, EFNA1, EHF, EMP2, ENPP2, EXT1, F11R, FGF2, FRZB, FST, FYN, GHRL, GJA1, GSK3B, HAS2, IGF1R, IL5, IL6, INPP5D, KCNJ2, KIT, KITLG, LYVE1, MAFF, MAP2K4, MAP2K6, MEF2C, MYH10, PDE2A, PLA2G2A, PLA2G3, PLA2G5, PPARG, PRKCA, RAC1, SDC2, SEMA3C, STAT5A, STMN1, TAGLN, TDRD7, TFAP2A, TGM2, TNF, TNNC1, TP53, TRAF6, WNT7A, ZNF750* | 1.42 x 10^-4^ | *GJA1, IL6, TNF* |
| *ADNP* | Transcription regulator | *ACADVL, ADIPOQ, ADM, AIF1, APC, APOD, APP, AQP1, BDNF, BLOC1S6, BMP4, CALB1, CDC42, CDK5R1, CITED2, CTNNB1, CTSB, CTSH, DCN, EFNA1, ELF5, ENPP2, EXT2, FGF2, FLOT1, FST, GJA1, GLI2, GSK3B, HAND1, HAS2, ID2, IGF1R, IL6, IRF6, ITIH4, KIT, KITLG, KRT17, KRT5, MAP2K6, MEF2A, NPY1R, PADI1, PIK3R1, PIK3R2, PIK3R3, PLA2G10, PPARG, PRKCB, PSEN2, RAC1, RDH10, S1PR1, SDC2, SEMA3C, SLC9A3R1, SNAP25, SOS1, TNF, TNNC1, TP53, WNT11, WNT7A* | 1.48 x 10^-4^ | *TNF, TP53* |
| *DHCR24* | Enzyme | *ACADVL, AKT3, APC, APP, AXIN2, CDH3, CITED2, COL4A1, CSK, CTNNB1, CTSB, CTSH, DNM2, E2F8, EFNA1, ENPP2, ERCC3, F11R, FGF2, FYN, GSK3B, GSN, HAS2, ID2, IGF1R, IL5, IL6, KCNMA1, KIT, KITLG, MAP2K4, MAP2K6, MAP2K7, MAPK12, MAPK3, MYH10, MYO10, PAFAH1B2, PDE2A, PIK3R1, PIK3R3, PPARG, PRKCA, PRKCB, PSEN2, PTCH1, RAF1, RBPJ, S100B, SEMA3C, ST14, STMN1, TCF7L2, TFDP1, TGFB1I1, TGM2, TNF, TP53, WNT2, WNT7A* | 1.48 x 10^-4^ | *APP, TNF* |
| *MIR-504* | MicroRNA | *AKT3, CTNNB1, CTSH, DCN, FRZB, FZD1, FZD6, FZD9, GSN, IGF1R, IL6, MAPK3, PLA2G2A, PPARG, STAT5B, TNF, TP53, WNT11, WNT16, WNT2, WNT2B, WNT7A, WNT8B* | 1.48 x 10^-4^ | *FOXP1, TP53* |
| *IL1RAPL1* | Trans-membrane receptor | *AIF1, APP, AXIN2, BDNF, BMP4, CALB1, CDC42, CDH3, CDK5R1, CITED2, CLTC, CTNNB1, CTSB, DCN, DPYSL2, ENPP2, EXT1, FGF2, FYN, FZD3, GAP43, GSK3B, HEY1, IGF1R, IL6, LMO4, MAFF, MEF2C, PAFAH1B2, PIK3R1, PLCG1, PPARG, PRKACA, PRKCA, PRKCB, PTCH2, RBPJ, S100B, S1PR1, SMARCA4, SNAP25, STMN1, TAGLN, TNF, TP53, TRAF6, UCHL1* | 1.48 x 10^-4^ | *IL6, TNF* |
| *STK38* | Kinase | *APP, AXIN2, BDNF, BMP4, CTNNB1, FGF2, GATA3, GSK3B, PPARG, TAGLN, TNNC1, TP53, WNT11, WNT7A* | 1.48 x 10^-4^ | *IL6, TNF* |
| *TRIM30A/ TRIM30D* | Other | *ADIPOQ, APOD, APP, AXIN2, BMP4, CAPZB, COL4A1, CTNNB1, ENPP2, FST, FZD3, GAP43, GATA3, GJA1, GLI2, HHIP, ID2, IFT57, KIT, KRT5, PIK3R1, PPARG, PRKCSH, PTCH1, PTCH2, SDC2, SEMA3C, STAT5A, STAT5B, TCF7L2, TGM2, TNF, TP53, VAV1, WNT11, WNT16, WNT2* | 1.48 x 10^-4^ | *IL6, TNF* |
| *CASP10* | Peptidase | *ADIPOQ, ADM, ANXA7, APP, AQP1, ASAP1, ATP6V0D1, BDNF, BMP4, CCL11, CDC42EP2, CDK5R1, CITED2, COL4A1, CTNNB1, CTSB, DCN, DVL2, EHF, ENPP2, FGF2, FST, FYN, GAP43, GHRL, GJA1, GSN, HEY1, ID2, IL5, IL6, INPP5D, ITIH4, KIT, MAFF, MAP2K7, MAPK9, PIK3R1, PLA2G2A, PLA2G2F, PLA2G5, PLD1, PPARG, PRKACA, PRKCA, PTCH1, S1PR1, STAT5A, STMN1, TCF7L2, TFDP1, TGM2, TNF, TNNC1, TOLLIP, TP53, TRAF6, UCHL1, VAV1* | 1.48 x 10^-4^ | *IL6, TNF* |
| Poly-saccharide TA-1 | Chemical reagent | *ADIPOQ, ADM, APP, AQP1, ARPC2, BDNF, BMP4, CAPRIN1, CCL11, CDK5R1, CITED2, COL4A1, CTNNB1, CTSB, CTSH, DCN, EDNRA, EXT1, EXT2, FCER1A, FGF2, FYN, FZD1, GATA3, GJA1, GLI2, GNAS, GSN, HAS2, HEY1, ID2, IL5, IL6, INPP5D, ITIH3, KCNMB1, KCNQ3, KCNV1, KIT, KITLG, KRT17, LYVE1, MAPK3, MEF2C, MYO10, NFIB, PFN2, PPARG, PRKCA, RAC1, STAT5A, STAT5B, TAGLN, TGFB1I1, TGM2, TMEM17, TNF, TP53, WNT11* | 1.48 x 10^-4^ | *IL6, TNF* |
| 12(S)-Hpete | Chemical - endogenous mammalian | *APC, AQP1, ARFIP2, ATP6V0D1, AXIN2, BCCIP, BMP4, CALB1, CDC42, CDC42EP2, CDK5R1, CPE, CTNNB1, CTSB, DNM2, EFNA1, ENPP2, FST, GATA3, GJA1, GLI2, GNAS, GSN, IGF1R, IL5, IL6, KCNMA1, MAP2K6, MAP2K7, MAPK11, MAPK12, MYH10, NFIB, PFN2, PTCH1, PTCH2, SCN1B, SEMA3C, SLC9A3R1, SOS1, STAT5A, STAT5B, STK4, SUFU, TCF7L2, TGM2, TNF, TP53, WASF2, WNT11* | 1.48 x 10^-4^ | *IL6, TNF* |
| Folic acid | Chemical - endogenous mammalian | *APC, AXIN2, BMP4, CITED2, CTNNB1, EXT2, FRZB, FST, GATA3, GJA1, GLI2, GSK3B, HAND1, HEY1, ID2, IRF6, KITLG, KRT17, MEF2C, PIK3R1, PPARG, RBPJ, TBX6, WNT8B* | 1.51 x 10^-4^ | *APC, APP, FZD1, FZD6, TP53* |
| *BMP*  *(BMP1-8, BMP10, BMP15)* | Group | *ADM, APOD, APP, AQP1, BMP4, CALB1, CDC42, CDK5R1, CITED2, CLTC, COL4A1, CPE, CTNNB1, CTSB, DCN, ENPP2, ERCC3, FGF2, FST, FZD10, GAP43, GJA1, GLI2, GNAS, HAS2, HEY1, ID2, IGF1R, IL5, IL6, KCNJ2, KIT, KITLG, KRT5, MAPK10, MAPK9, MEF2C, NME2, PIK3R1, PLD1, PPARG, PRKCA, PRKCB, PTCH1, RBPJ, S100B, SMARCA4, TGM2, TNF, TP53, VAV1, VAV2, WNT2B, WNT8B* | 1.58 x 10^-4^ | *DPYSL2, HAND1, ID2, IL6* |
| *ZEB2* | Transcription regulator | *ADIPOQ, AQP1, AXIN2, BDNF, CCL11, CDK5R1, COL4A1, CTNNB1, EHF, ENPP2, FGF2, GJA1, GSK3B, GSN, IL5, IL6, MAPK3, PLD1, PPARG, PRKCB, RAC1, RAC2, SCN1B, TCF7L2, TGM2, TNF, TP53, UPK2* | 1.58 x 10^-4^ | *FCER1A, INPP5D, PLCG1, PLCG2* |
| *LTF* | Peptidase | *ADIPOQ, BDNF, BMP4, FGF2, HEYL, IGF1R, IL6, NME2, PAFAH1B1, PLD1, RAC1, S100B, SUFU, TNF, UPK1B, UPK2* | 1.58 x 10^-4^ | *IL5, IL6, TNF, TP53* |
| Calcifediol | Chemical - endogenous mammalian | *ADM, APP, ARF1, ASAP1, BMP4, CAV3, CDH3, COL4A1, CTNNB1, CTSB, FGF2, FZD1, GJA1, GSN, HAS2, HEY1, ID2, IGF1R, IL6, MAPK12, MAPK3, MYH10, PRKCA, PRKCB, RAF1, TAGLN, TGFB1I1, TGM2, TNF, TP53* | 1.58 x 10^-4^ | *CCL11, IL6, TNF, TP53* |
| *RTN4* | Other | *ADIPOQ, AKT3, GATA3, GSK3B, IL6, INPP5D, MAPK10, PIK3CA, PIK3R1, PIK3R2, PIK3R3, PPARG, PRKACA, RAF1, SOS1* | 1.70 x 10^-4^ | *APP, BDNF, DPYSL2, GAP43, IL6* |
| *NEK2* | Kinase | *ADIPOQ, BDNF, CAV3, CCL11, CDC42EP1, CDH3, CDK5R1, CHN1, COL4A1, CTNNB1, CTSB, CTSH, FGF2, GAP43, GHRL, GJA1, GSK3B, HAS2, IGF1R, IL5, IL6, MAPK3, PIK3R1, PPARG, STAT5A, TNF, TP53, UPK2* | 1.70 x 10^-4^ | *ANXA7, CTNNB1, GAP43, ID2, IL6* |
| *MSX2* | Transcription regulator | *CCL11, CDK5R1, FYN, GNA11, IL5, IL6, MAP2K4, MAP2K6, MAP2K7, PRKACA, PRKCA, TNF* | 1.88 x 10^-4^ | *BMP4, CTNNB1, PPARG, WNT7A* |
| *SFRP1* | Trans-membrane receptor | *APC, CTNNB1, DVL2, FZD1, FZD3, GSK3B, MEF2A, SLC9A3R1, WNT16, WNT2B, WNT7A, WNT8B* | 1.88 x 10^-4^ | *APP, CTNNB1, GATA3, SDC2* |
| *ACVRL1* | Kinase | *APP, AXIN2, BDNF, BMP4, CTNNB1, DCN, DVL2, FZD1, GAP43, GSK3B, HAS2, IL6, KIT, KITLG, MEF2C, PLD1, PPARG, TNF, WNT11* | 1.88 x 10^-4^ | *EFNA1, ID2, LYVE1, TAGLN* |
| *NOTCH3* | Transcription regulator | *ADIPOQ, ADM, AIF1, APP, AQP1, BDNF, CALB1, CCL11, CDK5R1, CSK, CTNNB1, CTSB, CTSH, EDNRA, EHF, F11R, FGF2, FZD1, GATA3, GJA1, GNAS, HAS2, IGF1R, IL5, IL6, KCNMA1, KITLG, KRT17, MAFF, MYH10, PLA2G2A, PLA2G5, PLCG1, PLD1, PPARG, PRKCA, PSEN2, RAC2, SNAP25, TCF7L2, TFDP1, TNF, TP53, TRAF6* | 1.89 x 10^-4^ | *APOD, HEY1, SDC2, TAGLN, TP53* |
| *ANXA7* | Ion channel | *CSK, FYN, GSK3B, MAP2K4, MAP2K6, MAP2K7, MAPK12, MAPK3, PRKCB, RAF1, STK4, TP53* | 1.94 x 10^-4^ | *APC, CTSB, FST, FYN, GATA3, TFDP1* |
| *IDH1* | Enzyme | *APP, BDNF, CALB1, CAV3, CCL11, CTNNB1, EFNA1, EHF, FGF2, FST, GAP43, GATA3, GLI2, GNAS, HAS2, IL5, IL6, KIT, KRT17, PLD1, PPARG, PRKACA, RBPJ, STAT5A, TGM2, TNF, TOLLIP, TP53* | 2.01 x 10^-4^ | *KCNB2, MAPK10, PPARG* |
| *PAWR* | Transcription regulator | *ADM, AIF1, APP, BMP4, CALB1, CCL11, CTNNB1, CTSB, DCN, EFNA1, EHF, ENPP2, FGF2, FST, GATA3, GJA1, GNAS, GSK3B, HAS2, IL6, MAP2K6, MEF2C, PLA2G2A, PLA2G3, PLA2G5, PLD1, PPARG, RAC2, S100B, SNAP25, STAT5A, TGM2, TNF, TRAF6* | 2.01 x 10^-4^ | *APP, CCL11, MAPK3* |
| *RELN* | Peptidase | *BDNF, CALB1, CHN1, FST, GPM6A, IL6, MAPK10, MEF2C, PRKCA, PRKCB, SNAP25, TAGLN, TNF* | 2.01 x 10^-4^ | *APP, BDNF, LYVE1* |
| *GREM1* | Other | *ACADVL, ADIPOQ, ADM, APOD, APP, AQP1, AXIN2, COL4A1, CTNNB1, E2F8, EFNA1, FGF2, FST, FZD6, GAP43, GSN, ID2, IGF1R, IL6, KRT5, MAFF, PPARG, STAT5A, TGM2, TNF, TP53* | 2.01 x 10^-4^ | *AXIN2, TNF, WNT11* |
| Ic87114 | Chemical - kinase inhibitor | *ADIPOQ, APOD, APP, BMP4, CTNNB1, CTSB, GATA3, IGF1R, IL5, IL6, MAP2K7, PLPP3, PPARG, PRKCB, TNF, TRAF6, WNT2* | 2.01 x 10^-4^ | *CCL11, IL5, TNF* |
| Metoprolol | Chemical drug | *ADM, BMP4, COL4A1, EFNA1, IL6, KITLG, MAPK9, MYH10, PLPP3, PPARG, S1PR1, TNF, TRAF6, VAV1* | 2.01 x 10^-4^ | *AQP1, GJA1, IL6* |
| E64d | Chemical - protease inhibitor | *ADIPOQ, ADM, BDNF, CAV3, COL4A1, EDNRA, FGF2, GAP43, GJA1, GSK3B, HAS2, HEY1, IGF1R, IL6, KITLG, MAP2K7, MYH10, PIK3R1, PLA2G10, PPARG, RAC1, TNF, TP53* | 2.01 x 10^-4^ | *APP, FGF2, TP53* |
| *MIR-16-5p* (and other miRNAs w/seed AGCAGCA) | Mature microRNA | *APP, AXIN2, BMP4, CTNNB1, DCN, GATA3, GSK3B, HEYL, MAPK10, PLD1, PPARG, TP53* | 2.18 x 10^-4^ | *BDNF, CAPRIN1, FGF2, IGF1R, IL6, KITLG, MAP2K4, MAPK3, PAFAH1B2, RAF1* |
| *DLL4* | Other | *ADM, BDNF, CPE, DCN, ENPP2, FST, HAS2, MEF2C, MYH10, PLA2G2A, RBPJ, TCF7L2, TP53* | 2.22 x 10^-4^ | *GATA3, HEY1, IL5, LYVE1* |
| Amitriptyline | Chemical drug | *ADM, APP, BDNF, CTNNB1, CTSB, FGF2, GAP43, ID2, IL6, IRF6, KCNJ2, MAPK3, PLA2G6, PPARG, PRKCA, TNF* | 2.22 x 10^-4^ | *BDNF, FGF2, TAGLN, TP53* |
| Atenolol | Chemical drug | *ADIPOQ, AQP1, BMP4, CALB1, CDK5R1, COL4A1, CTSB, DCN, FGF2, FST, GHRL, GJA1, IGF1R, IL6, NPY1R, PICK1, PLA2G6, PLCG1, PPARG, PRKCB, RDH10, SNAP25, TGM2, TNF, TP53* | 2.22 x 10^-4^ | *APP, BDNF, GNAS, KCNJ2* |
| *NRL* | Transcription regulator | *ADIPOQ, BDNF, DOCK1, FYN, IL6, PLA2G5, RAC1, RAC2, TNF, VAV1* | 2.34 x 10^-4^ | *BMP4, IGF1R, MEF2C, PIK3CA, PIK3R1* |
| Epicatechin | Chemical drug | *ADIPOQ, CDC42, COL4A1, CTNNB1, EDNRA, FGF2, FST, GJA1, HAND1, HEY1, IL6, PRKCA, PRKCB, TGM2, TP53* | 2.34 x 10^-4^ | *DSE, HEY1, MAPK12, SNAP25, TNF* |
| *MKNK1* | Kinase | *ADIPOQ, COL4A1, DVL2, GATA3, GJA1, ID2, MAPK12, MEF2C, PIK3R1, PLPP3, PPARG, TNNC1* | 2.37 x 10^-4^ | *APC, GAP43, PAFAH1B1, SNAP25, TAGLN, TFDP1, TNF* |
| Lgk-974 | Chemical drug | *ADIPOQ, ADM, CCL11, CTSB, FGF2, FST, GAP43, GATA3, GJA1, HAS2, HEY1, ID2, IGF1R, IL5, IL6, KRT17, PLA2G10, PLA2G2A, RBPJ, S1PR1, TAGLN, TFDP1, TNF, TP53* | 2.60 x 10^-4^ | *APP, AXIN2, CTNNB1, GATA3* |
| *KCNMA1* | Ion channel | *CTNNB1, FZD1, FZD3, GSK3B, SLC9A3R1, WNT16, WNT2B, WNT8B* | 2.60 x 10^-4^ | *CCL11, GLI2, KCNMB1, PTCH1* |
| Rp-8-Br-cAMPS | Chemical - kinase inhibitor | *ADIPOQ, ADM, AIMP2, AQP1, ENPP2, FST, FZD1, GJA1, ID2, MAPK12, PIK3R1, S100B, SOS1, TP53* | 2.74 x 10^-4^ | *BDNF, PRKACA, TNF* |
| *IL17RB* | Trans-membrane receptor | *EFNA1, GJA1, GNAS, HAS2, IL5, IL6, KITLG, MAP2K6, MEF2C, PLA2G5, TAGLN, TGM2, TNF, TP53* | 2.74 x 10^-4^ | *CCL11, IL5, IL6* |
| Albuterol | Chemical drug | *APP, BDNF, CDK5R1, CLTC, CTNNB1, DPYSL2, ENPP2, GJA1, HAND1, PAFAH1B2, PIK3R1, PPARG, PSEN2, RBPJ, S100B, SNAP25, STMN1, TNF, TP53, UCHL1* | 2.74 x 10^-4^ | *GNAS, IL6, TNF* |
| Lena-lidomide | Chemical drug | *BDNF, CDK5R1, CTNNB1, FGF2, GJA1, GSK3B, HAS2, IGF1R, IL6, MAP2K4, MEF2A, MEF2C, PIK3R1, PLA2G2A, PPARG, S1PR1, SEMA3C, SOS1, TAGLN, TNF, TP53, UPK2, WNT7A* | 2.91 x 10^-4^ | *AGPS, CTNNB1, FZD10, NFIB, TCF7L2, TNF, WNT11, WNT2B, WNT8B* |
| *CABP1* | Other | *APC, FZD1, FZD6, PTCH1, SUFU, TNF* | 3.02 x 10^-4^ | *HAS2, IL6, MAPK3, TP53* |
| *GMNN* | Transcription regulator | *ADIPOQ, ADM, APOD, AQP1, BDNF, CITED2, EDNRA, EFNA1, ENPP2, FST, GAP43, GJA1, HEY1, IL5, IL6, KRT5, MYO10, NME2, NPY1R, PIK3R1, PLPP3, STAT5A, STAT5B, TNF, TP53* | 3.29 x 10^-4^ | *CTNNB1, EXT2, FGF2, FRZB, HAND1, WNT8B* |
| *PAX3* | Transcription regulator | *ADIPOQ, ADM, APP, COL4A1, CTNNB1, CTSH, EFNA1, ENPP2, GHRL, ID2, IGF1R, IL6, ITIH3, KRT17, NFIB, PLA2G2A, PPARG, TNF, TP53* | 3.49 x 10^-4^ | *BMP4, CDH3, ID2, TP53* |
| *HAVCR1* | Other | *ADIPOQ, ADM, APOD, APP, AQP1, AXIN2, BDNF, CALB1, COL4A1, CPE, CTNNB1, CTSB, DCN, EDA, EDNRA, EFNA1, EHF, FGF2, FST, GAP43, GATA3, GHRL, GJA1, ID2, IGF1R, IL5, IL6, KIF4A, KIT, KRT17, LMO4, LYVE1, MAPK3, PAFAH1B1, PIK3R1, PLD1, PPARG, PRKCA, PRKCB, SLC35D1, STAT5A, SULT1B1, TAGLN, TGM2, TNF, TP53, ZNF750* | 3.62 x 10^-4^ | *GATA3, IL5, TNF* |
| *MIB1* | Enzyme | *ADIPOQ, BMP4, CTSB, CTSH, EDNRA, EHF, GPM6A, HEY1, HHIP, IL6, IRF6, KCNJ2, KCNQ3, KIT, MAFF, MEF2C, PLPP3, PPARG, PTCH1, RAC2, SDC2, SMARCA4, TAGLN, TP53, WNT7A* | 3.62 x 10^-4^ | *HEY1, HEYL, TP53* |
| *MIR-30c-5p* (and other miRNAs w/seed GUAAACA) | Mature microRNA | *ADIPOQ, AQP1, BMP4, CAV3, CCL11, CDK5R1, EFNA1, EHF, EMP2, GJA1, IL5, IL6, MAP2K6, MAPK12, PPARG, TAGLN, TGM2, TNF, TP53* | 3.79 x 10^-4^ | *BDNF, IL6, MYO10, PAFAH1B2, PPARG, TNF, TP53* |
| Maslinic acid | Chemical - endogenous non-mammalian | *APP, BDNF, DNM2, FCER1A, FGF2, FST, HHIP, IGF1R, IL6, NPY1R, PPARG, PTCH1, TNF* | 4.01 x 10^-4^ | *GSK3B, PRKACA, SOS1, TP53* |
| Arundic acid | Chemical drug | *AIF1, APP, BDNF, CDK5R1, CLTC, DPYSL2, FYN, IL6, MAPK11, MAPK3, PAFAH1B2, S100B, SNAP25, STMN1, TNF, UCHL1* | 4.39 x 10^-4^ | *APP, S100B* |
| *PTCHD4* | Other | *AQP1, CALB1, CDC42, COL4A1, DPP6, FYN, GATA3, GATA5, GSN, IL6, INPP5D, KIT, PLCG1, PPARG, PRKACA, PRKCB, RAF1, STAT5B, TGM2, TNF, TP53* | 4.39 x 10^-4^ | *PTCH1, PTCH2* |
| *SPON2* | Other | *ADIPOQ, COL4A1, CTSH, ENPP2, IL5, IL6, PPARG, PRKCSH, RAC1, TNF* | 4.39 x 10^-4^ | *IL6, TNF* |
| *LILRA4* | Other | *ADM, APP, BDNF, CDC42, CDK5R1, CPE, CTNNB1, CTSB, FGF2, FOXP1, FST, FZD1, GAP43, GATA3, GJA1, HAS2, ID2, IL5, IL6, KIT, KRT71, LYVE1, MAPK9, NME2, PLD1, PPARG, PRKACA, PRKCA, PRKCB, RBPJ, SNAP25, STAT5B, TFDP1, TNF, TP53* | 4.39 x 10^-4^ | *IL6, TNF* |
| *SCARB2* | Other | *AXIN2, CTNNB1, FST, GATA3, GLI2, HAS2, HEY1, ID2, IL5, IL6, INPP5D, RAC1, S1PR1, TAGLN, TNF* | 4.39 x 10^-4^ | *IL6, TNF* |
| *SBDS* | Other | *APP, BDNF, CTSB, DCN, FST, GJA1, IGF1R, IL5, IL6, KIT, KITLG, MAP2K6, PLA2G2A, PLD1, PPARG, TGM2, TNF, TP53* | 4.39 x 10^-4^ | *RAC1, RAC2* |
| *GCA* | Other | *APP, FST, GJA1, ID2, IGF1R, IL6, PLPP3, SNAP25, TP53* | 4.39 x 10^-4^ | *IL6, TNF* |
| *CFH* | Other | *ADM, APC, CDC42, CITED2, COL4A1, CTNNB1, DNM2, FST, IL6, MAFF, PIK3CA, PIK3R3, RAC2, SUFU, TAGLN, TP53, UPK1A, UPK1B, UPK2* | 4.39 x 10^-4^ | *PPARG, TNF* |
| *RPS7* | Other | *FGF2, FST, IGF1R, IL6, KIT, S1PR1, TNF, TP53* | 4.39 x 10^-4^ | *PIK3R1, TP53* |
| *DHCR7* | Enzyme | *ADIPOQ, ADM, APOD, AXIN2, CAV3, CTNNB1, EHF, IL5, IL6, KCNIP3, KITLG, PPARG, TNF* | 4.39 x 10^-4^ | *IL6, TNF* |
| *SNRNP70* | Other | *CCL11, CITED2, IL5, IL6, LMO4, STAT5A, TNF, TP53* | 4.39 x 10^-4^ | *APP, PSEN2* |
| Ketotifen | Chemical drug | *CDC42EP1, DCN, DVL2, FGF2, FST, GJA1, GSK3B, HAS2, ID2, IGF1R, IL6, KRT5, MAPK11, MAPK3, PLPP3, PPARG, RAC1, SNAP25, TFAP2A, TFDP1, TGM2, TP53* | 4.39 x 10^-4^ | *IL6, TNF* |
| Paxilline | Chemical - endogenous non-mammalian | *ADM, APP, BDNF, ID2, TNF, TP53* | 4.39 x 10^-4^ | *IL6, TNF* |
| Aripiprazole | Chemical drug | *BDNF, CDK5R1, DNM2, FCER1A, FST, GAP43, HHIP, IGF1R, IL5, IL6, KIT, KITLG, PTCH1, S100B, SLC9A3R1, SNAP25, TNF* | 4.39 x 10^-4^ | *BDNF, TNF* |
| Micro-  cystin-LR | Chemical toxicant | *COL4A1, CPE, CSK, CTSH, GSN, HAND1, IL6, KIT, KITLG, PPARG, PSEN2, TAGLN* | 4.39 x 10^-4^ | *IL6, TNF* |
| Sr 31747 | Chemical drug | *ACADVL, CCL11, COL4A1, GAP43, GSN, IL6, ITIH4, KRT71, MAP2K6, PFN2, PRKCB, RAC1, STAT5B, TNF, TP53* | 4.39 x 10^-4^ | *IL6, TNF* |
| *PSEN2* | Peptidase | *BMP4, CCL11, GATA3, GLI2, HHIP, IL6, KRT17, MEF2C, PTCH1, TNF* | 4.64 x 10^-4^ | *APP, CTNNB1, ENPP2, GJA1, HAND1, TP53* |
| *CaMKII* | Complex | *BCCIP, CCL11, CDH3, CSK, CTNNB1, CTSB, DCN, ENPP2, FGF2, HEY1, IL5, IL6, PIK3R1, RAC1, SEMA3C, TFDP1, TGFB1I1, TNF, TOLLIP, TP53* | 4.66 x 10^-4^ | *BDNF, HAS2, TP53* |
| *FZD8* | G-protein coupled receptor | *AXIN2, BMP4, CTNNB1, ENPP2, IGF1R, KIT, KRT5, PPARG, SEMA3C, STAT5A, TP53* | 4.66 x 10^-4^ | *BMP4, CTNNB1, TP53* |
| *ELF1* | Transcription regulator | *CCL11, CDC42, CDC42EP2, CDH3, COL4A1, CTNNB1, CTSB, E2F8, EHF, GATA3, GJA1, GSK3B, HAS2, HEYL, ID2, IGF1R, IL6, IRF6, KIT, MYO10, NFIB, PFN2, PPARG, STMN3, TAGLN, TNF, TP53* | 4.66 x 10^-4^ | *CITED2, FCER1A, IL5* |
| *FCER1A* | Trans-membrane receptor | *APP, FYN, IL6, TNF, TP53* | 4.66 x 10^-4^ | *IL6, PLCG2, TNF* |
| Na^+^ | Chemical - endogenous mammalian | *ADIPOQ, AXIN2, BDNF, GNAS, GSK3B, IL5, IL6, MEF2C, PPARG, SLC35D1, TAGLN, TBX6, TGM2, TNF, TP53* | 4.66 x 10^-4^ | *CTNNB1, TNF, TP53* |
| Rasagiline | Chemical drug | *APP, AQP1, ATP2C1, BDNF, CITED2, COL4A1, CTNNB1, DCN, ENPP2, ERCC3, GAP43, GSN, KCNJ2, NCS1, NFIB, PFN2, PIK3CA, PPARG, PRKCB, SNAP25, STMN1, TAGLN, TBX6, TGM2, TP53, UCHL1* | 5.21 x 10^-4^ | *APP, BDNF, PRKCA, SOS1* |
| *SPRY1* | Other | *GNAS, IL6, PIK3CA, PIK3R1, PLD1, TP53* | 5.88 x 10^-4^ | *AXIN2, ID2, LMO4* |
| Pyridoxine | Chemical - endogenous mammalian | *APC, APP, FZD1, FZD6, PTCH1, TNF* | 5.88 x 10^-4^ | *APC, FZD1, FZD6* |
| Pilocarpine | Chemical drug | *ADM, APP, BDNF, CCL11, CITED2, CTNNB1, FGF2, FST, GAP43, GJA1, GNAS, IL5, IL6, PLA2G2A, PPARG, PSEN2, TNF, TP53* | 5.88 x 10^-4^ | *APP, BDNF, KCNQ3* |
| Cobalt | Chemical toxicant | *ADIPOQ, ENPP2, IL6, PPARG, TNF* | 5.88 x 10^-4^ | *FGF2, IL6, TNF* |
| *MIR-124-3p* (and other miRNAs w/seed AAGGCAC) | Mature microRNA | *CTNNB1, GSK3B, IGF1R, IL6, PRKCA, PRKCB, TP53* | 5.99 x 10^-4^ | *BDNF, BLOC1S6, DNM2, DVL2, F11R, GSK3B, GSN, MYO10, RDH10, TRAF6* |
| Thiazolidine-dione | Chemical drug | *ENPP2, FGF2, GAP43, HAS2, IL6, PLA2G2A, S1PR1, TAGLN, TNF* | 7.28 x 10^-4^ | *ADIPOQ, CTNNB1, PIK3R1* |
| *FBXO2* | Enzyme | *AGPS, ARF1, ASAP1, ATP2C1, BDNF, CTSB, CTSH, DOCK1, FGF2, FRZB, FZD3, FZD6, GJA1, GSK3B, HAS2, IL6, KITLG, PLD1, STMN1, TNF, TP53, WNT11* | 7.28 x 10^-4^ | *APP, IL6, TNF* |
| *FcGR2*  *(FCGR1, FCGR2)* | Group | *ADM, AIF1, BDNF, CALB1, GATA3, GNAS, IL6, MAPK3, TNF, TP53, TRAF6* | 8.71 x 10^-4^ | *IL5, IL6* |
| *SNX27* | Other | *BMP4, CCL11, GJA1, HEY1, ID2, IL6, KIT, PTCH1, TAGLN, TGM2, TP53* | 8.71 x 10^-4^ | *APP, PPARG* |
| Enecadin | Chemical drug | *ADM, AQP1, BDNF, BMP4, CPE, FLOT1, GAP43, HAS2, IL6, MAFF, SCN1B, SLC9A3R1, TAGLN, TNF, TP53* | 8.71 x 10^-4^ | *BDNF, FGF2* |
| *DACT3* | Other | *CALB1, CTSB, DCN, DPYSL2, GJA1, IL5, IL6, TNF, TP53, WNT11* | 8.71 x 10^-4^ | *AXIN2, DVL2* |
| *APH-1*  *(APH1A, APH1B, APH1C)* | Group | *ACADVL, GATA5, GLI2, HHIP, ID2, IL6, MAPK11, MEF2C, PTCH1, PTCH2, TMEM100, TP53, WNT2B* | 8.71 x 10^-4^ | *APP, PSEN2* |
| *UNG* | Enzyme | *CDK5R1, CITED2, COL4A3BP, EDNRA, FGF2, FST, IGF1R, IL6, KIT, MAP2K7, MAPK3, MEF2C, PPARG, PSEN2, TFDP1, TNF, TNNC1, TP53* | 8.71 x 10^-4^ | *BDNF, TP53* |
| *GALNS* | Enzyme | *ENPP2, GJA1, IL6, KIT, KITLG, NME2, PPARG, PRKCA, PRKCB, TFDP1, TNF, TP53* | 8.71 x 10^-4^ | *BDNF, GAP43* |
| *SUFU* | Transcription regulator | *APP, CALB1, CDK5R1, COL4A1, EHF, GATA3, ID2, IGF1R, IL5, IL6, KCNH1, PADI1, PLCG1, PLD1, PPARG, PRKCA, PRKCB, STMN1, TMEM100, TNF, TP53, WNT11* | 8.71 x 10^-4^ | *HHIP, PTCH1* |
| *HAMP* | Other | *CALB1, FGF2, HAS2, IL6, TNF* | 8.71 x 10^-4^ | *IL6, TNF* |
| *DDIT4* | Other | *CCL11, CTNNB1, DCN, FGF2, FST, GATA3, GLI2, HEY1, IL5, IL6, INPP5D, PLPP3, TAGLN, TNF* | 8.71 x 10^-4^ | *TNF, TP53* |
| *DVL3* | Other | *CTNNB1, FGF2, GATA3, GLI2, HEY1, HEYL, ID2, IGF1R, IL6, LYVE1, PPARG, TAGLN, TNF, TP53* | 8.71 x 10^-4^ | *CTNNB1, GSK3B* |
| *SCIN* | Other | *CDH3, CTNNB1, ENPP2, ERCC3, IGF1R, KIT, KRT17, KRT5, STAT5A, STAT5B, TNF, TP53* | 8.71 x 10^-4^ | *GSN, RAC2* |
| *CD300LD* | Other | *ADIPOQ, ARF1, BDNF, FGF2, GATA3, IGF1R, IL6, MAPK3, PIK3R1, PIK3R2, PPARG, PRKCA, PRKCB, SNAP25, STAT5A, TNF* | 8.71 x 10^-4^ | *IL6, TNF* |
| Fluticasone | Chemical drug | *APP, BDNF, CALB1, FGF2, IL6, MAP2K4, TNF, TP53* | 8.71 x 10^-4^ | *CCL11, IL6* |
| Verlukast | Chemical drug | *APP, CCL11, CITED2, COL4A3BP, CTSB, EHF, FGF2, GLI2, HAS2, IGF1R, IL6, KIT, PLD1, PPARG, STAT5A, TGM2, TNF, TP53* | 8.71 x 10^-4^ | *IL5, TNF* |
| Scopoletin | Chemical - endogenous non-mammalian | *BDNF, BMP4, CALB1, CTNNB1, DCN, ENPP2, FGF2, GAP43, GJA1, HAS2, IGF1R, IL6, LYVE1, PPARG, TAGLN, TNF, TP53* | 8.71 x 10^-4^ | *IL5, IL6* |
| *NUMB* | Other | *ADM, BDNF, ENPP2, FST, FYN, FZD1, GHRL, GJA1, HAS2, IGF1R, IL6, MAPK3, PLPP3, PPARG, SNAP25, TNF, TP53* | 8.88 x 10^-4^ | *APP, HEY1, TP53* |
| *MIR-221* | MicroRNA | *CDK5R1, GAP43, GATA3, GJA1, GLI2, IGF1R, IL6, MAP2K4, MEF2C, PLA2G5, STMN1, TAGLN, TGM2, TNF, TP53* | 8.88 x 10^-4^ | *KIT, PIK3R1, STAT5A* |
| Nimesulide | Chemical drug | *ADM, AQP1, CCL11, FGF2, FST, GJA1, HAS2, IL6, KIT, NME2, NPY1R, SNAP25, STAT5A, TNF, TP53* | 8.88 x 10^-4^ | *APP, CTNNB1, EDNRA* |
| Phenytoin | Chemical drug | *ADM, APP, AQP1, ARPC2, BDNF, EFNA1, GAP43, GATA3, IGF1R, IL6, KRT17, KRT5, LMO4, MAPK3, MAPK9, PIK3R1, PIK3R3, PPARG, PRKCB, RAF1, SEMA3C, STAT5A, TNF, TP53* | 8.88 x 10^-4^ | *BDNF, MAPK3, RAF1* |
| *MIR-182-5p* (and other miRNAs w/seed UUGGCAA) | Mature microRNA | *APP, BDNF, IL6, MAP2K7, TNF* | 9.30 x 10^-4^ | *IGF1R, PIK3CA, RAC1, SOS1* |
| *HOXA9* | Transcription regulator | *APC, BMP4, CALB1, CTNNB1, ERCC3, GHRL, ID2, IGF1R, IL6, PPARG, SMARCA4, STAT5A, TFAP2A* | 9.45 x 10^-4^ | *EDNRA, FZD1, ID2, KIT, PIK3R2* |

^1^Master regulators are regulators that are indirectly connected to genes in the dataset through upstream regulators

^2^Molecule type of the master regulator as defined by the Ingenuity Pathway Analysis

^3^Upstream regulators controlled by each master regulator

^4^P-value with fisher’s exact test

^5^List of leading edge genes and positional candidate genes regulated by each upstream regulator
